# Algorithm-driven, phenotype-directed bioactive molecular discovery

**DOI:** 10.1038/s42004-026-02066-8

**Published:** 2026-05-16

**Authors:** Amalia-Sofia Piticari, Samuel D. Griggs, Laura Crawford, Joss Whittle, Brian S Mantilla, Sonja Sievers, Megan H. Wright, Stephen P. Marsden, Mark Basham, Adam Nelson

**Affiliations:** 1 Rosalind Franklin Institute, Harwell Campus, Didcot, Oxfordshire OX11 0QX UK; 2 Astbury Centre for Structural Molecular Biology, University of Leeds, Leeds, LS2 9JT UK; 3 School of Chemistry, University of Leeds, Leeds, LS2 9JT UK; 4 Department of Chemical Biology, Max Planck Institute of Molecular Physiology, Otto-Hahn-Strasse 11, 44227 Dortmund, Germany; 5 Compound Management and Screening Center, Otto-Hahn-Strasse 11, 44227 Dortmund, Germany

**Keywords:** Combinatorial libraries, Screening, Combinatorial libraries, Cheminformatics

## Abstract

The discovery of bioactive small molecules is dominated by iterative design-make-purify-test cycles focused on specific protein targets. In contrast, phenotype-driven discovery can yield bioactive molecules with unexpected mechanisms of action, and open paths to first-in-class drugs. Here, we present a fully closed-loop, algorithm-driven workflow for phenotypic-driven molecular discovery. Initially, a large virtual reaction space is constructed from pairs of potential substrates and co-substrates. Batches of reactions are then algorithmically-designed and automatically executed, and the products screened in a phenotypic assay; based on observed hits, the algorithm then directs subsequent round(s) of discovery and optimisation until a user-defined end-point is reached. The approach was exemplified using Rh-catalysed annulations of hydoxamate esters with alkene/alkyne co-substrates, coupled with the cell painting assay, and enabled the discovery and structural evolution of a series of tubulin modulators. Because the workflow is agnostic to both the chemistry and the assay modality, the approach may be generalisable for the automated function-directed exploration of synthetically-accessible chemical space. The approach has the potential to accelerate the discovery of chemical probes and to unlock opportunities for drug discovery.

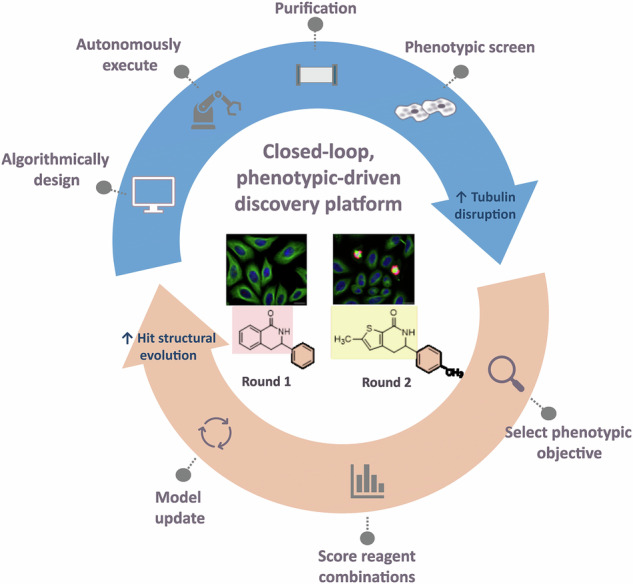

## Abstract (web summary)

Drug discovery typically relies on iterative design-make-purify-test cycles focused on specific, pre-selected protein targets. Here, the authors introduce an algorithm-driven, closed-loop workflow for phenotype-driven discovery which was exemplified through the discovery and structural evolution of a series of tubulin modulators.

## Introduction

Functional molecules are central to many scientific domains including medicine, biotechnology and materials science^1–7^. In the context of drug discovery, target-centric approaches (i.e. those that focus on specific proteins) have traditionally dominated, and can be driven by structural and biophysical approaches^1,8,9^. However, such reductionist approaches are inherently biased towards previously identified protein targets. They also overlook the possibility of polypharmacology (i.e. action on multiple proteins) and the complexity of target biology within native contexts^10,11^. Furthermore, target-centric approaches have been a declining source of late-stage clinical candidates^12,13^. In contrast, phenotypic screens of pre-synthesised compound collections harness cellular, tissue or organismal models, and can potentially uncover bioactive molecules that have distinctive mechanisms^14,15^. Indeed, over the past 30 years, phenotypic approaches have yielded more first-in-class small-molecule drugs than target-based approaches^13^.

**Fig. 1:**
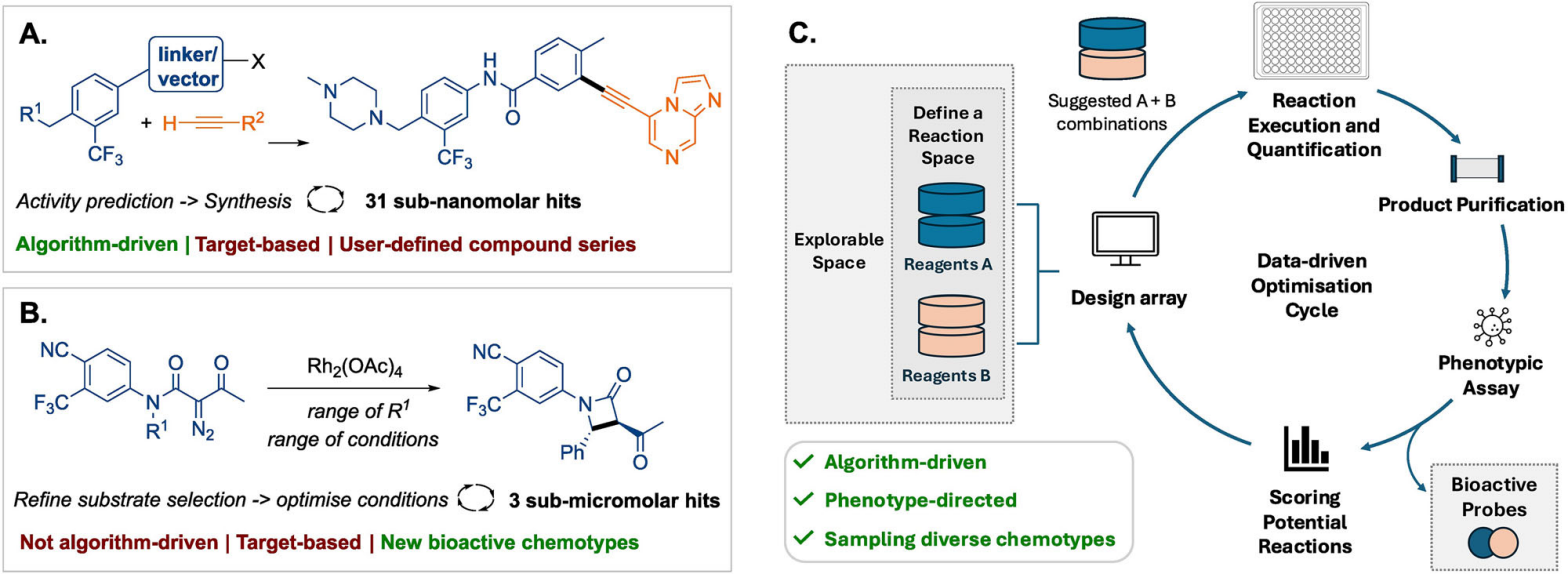
High-throughput experimental approaches for the discovery of bioactive small molecules. **A** Closed-loop discovery of ABL1 kinase inhibitors. **B** Activity-driven synthesis of androgen receptor agonists. **C** This work: algorithm-driven, phenotype-directed molecular discovery in which bioactive molecules are structurally elucidated after identification of hit reactions.

The discovery of bioactive small molecules, such as drugs and chemical probes, is generally driven via iterative design–make–purify–test cycles. Molecular discovery is dominated by a small number of robust synthetic reactions^16,17^, which has contributed to the uneven historic exploration of chemical space and the limited diversity of exemplified bioactive scaffolds^18^. To expedite discovery, automation is widely used at individual stages within discovery cycles. Furthermore, platforms that integrate multiple automated stages have emerged^19–29^, and include examples that support closed-loop discovery cycles^30^. For example, active learning has been integrated with microfluidic-based synthesis, purification and biochemical evaluation to explore the structure-activity relationships of a known series of ABL1 kinase inhibitors (Fig. 1A)^31^. In addition, other platforms have fully integrated flow synthesis with in-line biological assays, whilst using activity data to drive molecular optimisation^32–34^. To date, iterative closed-loop approaches have focused exclusively on target-based molecular discovery in which the structures of prepared molecules are determined in advance.

Several high-throughput molecular discovery approaches are differentiated from workflows that embed iterative design–make–purify–test cycles. By harnessing reactions with inherently unpredictable outcomes, activity-directed synthesis has enabled the structure-blind, function-driven discovery of series of androgen receptor modulators (Fig. 1B)^35^. Affinity-selection mass spectrometry also enabled molecular function to be determined within discovery cycles without prior purification of crude reaction mixtures^36^. Dynamic combinatorial chemistry has been used to triage mixtures of alternative products on the basis of their affinity for a specific target protein^37^.

In this paper, we describe, for the first time, an algorithm-driven, phenotype-directed molecular discovery platform (Fig. 1C and Supplementary Fig. 1). Initially, a large virtual reaction space would be defined in terms of possible combinations of substrates (Reagents A) and co-substrates (Reagents B) (~10^4^-10^5^ reactions based on all possible combinations). A diverse subset of possible substrate/co-substrate combinations would then be algorithmically designed for experimental investigation. Leveraging integrated automation capabilities, these reactions would then be executed in parallel, and the observed products quantified and purified. The purified products, whose structures would not have been elucidated at this stage, would then be screened in a phenotypic assay, enabling scoring of the most promising substrate/co-substrate combinations (i.e. those yielding products with a desirable function). By avoiding structural elucidation of all products, this approach would save time and avoid premature assumptions about structure–activity relationships until the functional relevance of specific chemotypes had been established. Propagation of these scores to related substrate/co-substrate combinations within the virtual reaction space would then enable the algorithm-driven design of further sets of reactions for execution. Our overall goal was to realise a data-driven, automated closed-loop approach in which the discovery and optimisation of bioactive molecular series was driven by phenotypic effects alone.

## Results

### Design and development of a general workflow

**Fig. 2:**
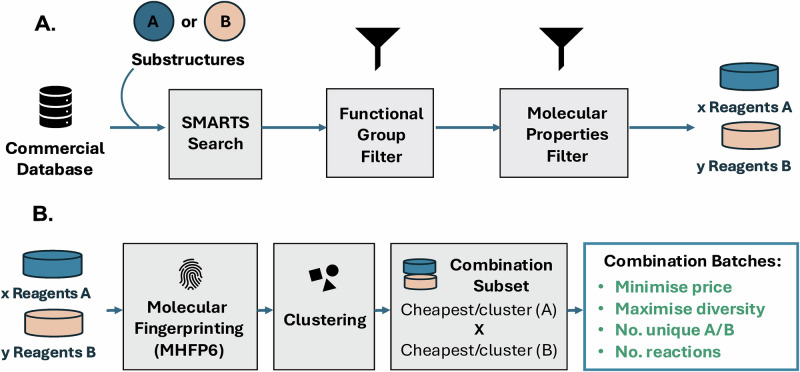
Overview of the definition of virtual reaction spaces and the design of batches of reactions for execution. **A** Customisable pipeline for filtering commercially available compounds to yield virtual reagent sets. **B** Python-based workflow for generating potential batches of reactions for execution.

Initially, sets of substrates (Reagents A) and co-substrates (Reagents B) were identified, whose combinations would define the virtual reaction space. These substrate/co-substrate pairs could be combined using a single-step, automatable transformation that would ideally have the potential to form unexpected and/or multiple alternative products. A filtering process was devised to identify readily-available compounds with appropriate structural features and molecular properties (Fig. 2A). Initially, information on commercially available compounds was pooled from our internal procurement system. A SMARTS query was used to select compounds with specific substructures, necessary for the selected reaction class, and which also lacked undesirable functionality^38^. Thresholds for molecular properties such as heavy (non-hydrogen) atom count (HA), lipophilicity (XlogP), aromatic atom count (AA), and rotatable bond count (nRotB) ensured that the reagents had fragment-like molecular properties; the thresholds were informed by the need to explore chemical space efficiently and for the products to align with established developability metrics for bioactive molecules^39–44^. Overall, the workflow enabled the definition of large virtual reaction spaces based on all pairwise combinations of the filtered reagent sets.

A Python-based algorithm was developed to generate multiple potential batches of reactions (i.e. substrate/co-substrate combinations) for execution (Fig. 2B). To navigate the structural landscape of the pre-filtered reagents, the reagents were firstly encoded using MinHashed MHFP6 molecular fingerprints^45^, a high-performing probabilistic method that captures many pharmacologically-relevant structural features^46^. After individually clustering reagent sets (A and B) based on their MHFP6 fingerprints, the cheapest representative from each cluster was selected using parsed pricing data. This yielded a reduced **A** × **B** combination subset that retains most of the diversity of the full virtual reaction space. Finally, a constrained MaxMin algorithm was used to generate options for reaction batches for which, within user-defined specifications, the structural diversity was maximised^47^. These user-defined specifications could include limitations on the number of unique reagents and the total number of reactions in each batch, to align with automation capabilities (for example, physical space/positions on the robot deck). A bespoke KNIME workflow (Supplementary Information) then converted information on the selected batch into input instructions for automated synthesis, an approach that would be suitable for many different chemistries and platforms. Alternatively, it would be possible for the batch to be assembled manually using pipettes as in many target-focused, “direct-to-biology” workflows^48^. To promote sustainability and reproducibility, all chemical and biological data, including reagent selection and reaction outcomes, were stored with version control using GitHub to enable traceable, updatable, and reusable analyses.

### First round of molecular discovery

**Fig. 3:**
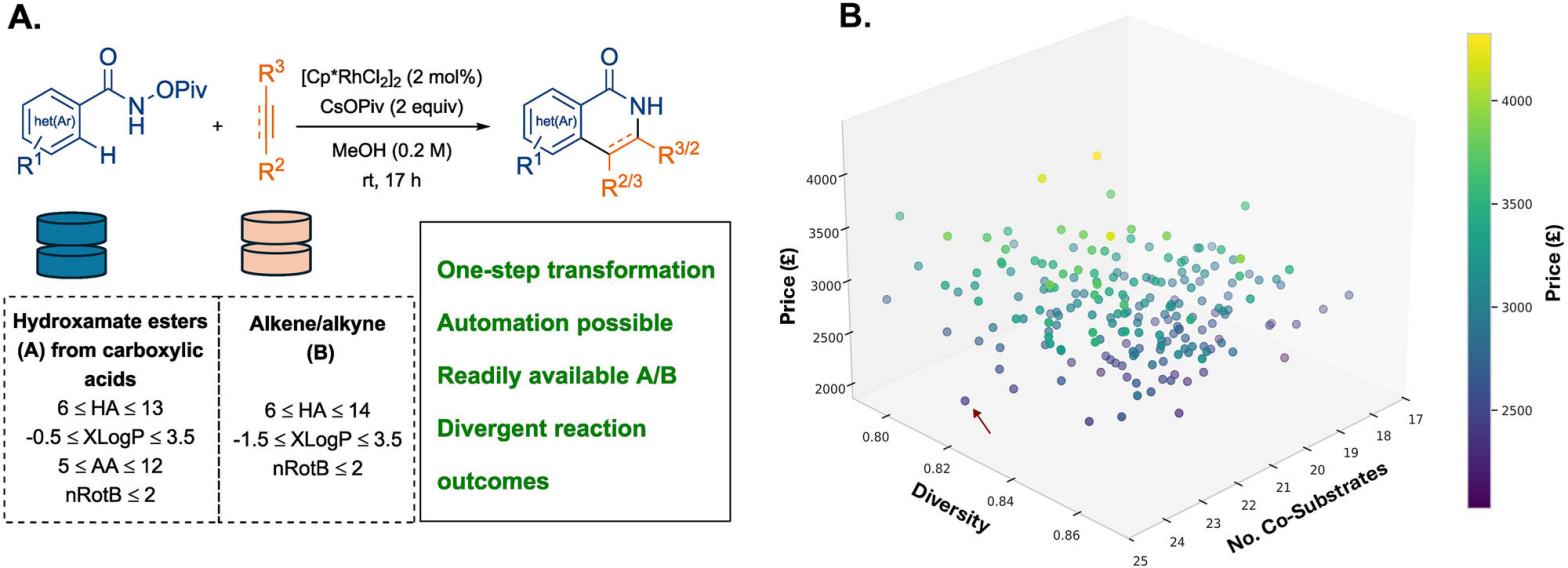
Definition of a specific virtual reaction space and design of alternative batches of reactions for execution. **A** Chosen Rh-catalysed annulation in which aromatic hydroxamate ester substrates would be combined with alkene/alkyne co-substrates. **B** Metrics for the 100 algorithmically generated batches of reactions.

We exemplified our discovery approach using the cell painting assay which enables morphological effects of compounds to be profiled^49–51^; like other assays^52^, high content images may be transformed into low-dimensional representations (morphological fingerprints). To enable the discovery of biologically relevant, yet synthetically accessible compounds, we recognised the value of connective reactions that may yield more than one possible product. Metal-catalysed C–H functionalisation reactions have potential for defining and exploring biologically relevant reaction spaces because such chemistries frequently enable modification of bioactive fragments such as (het)aromatic or saturated rings^53–55^. To exemplify our workflow, we chose the Rh-catalysed annulation of hydroxamate esters (Reagents A) with alkenes or alkynes (Reagents B) (Fig. 3A)^56,57^. This one-step transformation is feasible for automation, and uses readily-prepared/available reagents to yield annulated products. Alternative products could potentially be formed, given possible variations in the C–H activation site, annulation mode (e.g. head-to-head or head-to-tail), saturation (alkene *vs* alkyne) and/or diastereochemical outcome.

Using the workflow (Fig. 2) and filters shown in Fig. 3A, 850 (het)aromatic carboxylic acids (which may be readily converted into hydroxamate esters) and 354 alkene/alkynes were filtered from commercial databases. The diversity of products that may be potentially accessible from these reagent sets was compared with that of the ChEMBL database, and was shown to be high (Supplementary Fig. 2). We found that batches of reactions chosen from all 850 × 254 combinations of substrates **A** and co-substrates **B** were prohibitively expensive (Supplementary Fig. 3A). It has previously been shown that implementation of hard price cut-offs can adversely affect the diversity of molecular properties of commercially available building blocks^58^. Instead of using hard price cut-offs, we clustered the sets into 128 clusters, using kmeans and based on MHFP molecular fingerprints, from which the cheapest representatives were chosen. Batches of reactions were then chosen from the 128 × 128 pairwise combinations of the substrates **A** and co-substrates **B**. Here, the constrained MaxMin algorithm was used to design alternative batches of 80 reactions, involving, at most, 11 unique substrates and 25 unique co-substrates, whilst maximising diversity (see Supplementary Fig. 4 for comparison with uniform sampling). The 100 generated batches utilised substrates/co-substrates with total costs less than £4000, with diversity scores between 0.80 and 0.86 (Fig. 3B); these batches were much cheaper, but had comparable diversity, to those chosen from the full combination space (Supplementary Fig. 3). Notably, our approach enables balancing of multiple considerations to guide the choice of a specific batch of reactions for execution. We selected the highlighted batch, which harnessed the maximum number (25) of co-substrates at an overall cost of less the £2500.

**Fig. 4:**
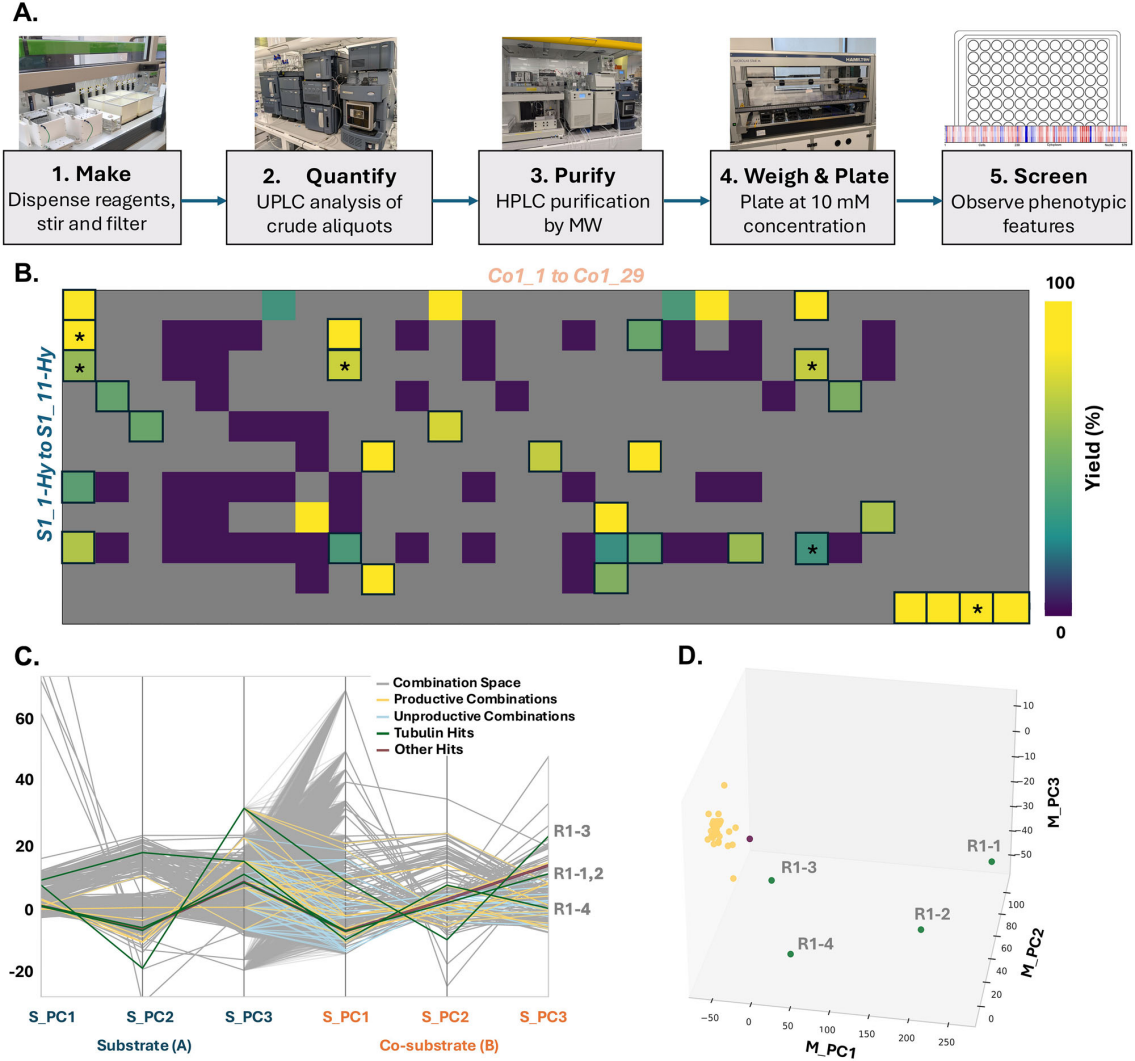
First round of phenotype-directed molecular discovery. **A** High-throughput experimental workflow with images of the automation equipment used. **B** Heat map of analytical ELS-determined yields for the executed reactions (80 algorithmically suggested combinations and 4 controls): grey, not executed; boxes = successful product isolation; asterisk = multiple products. **C** PCA of structural MHFP fingerprints (S_PC1-3) for substrate/co-substrate combinations shown on a parallel coordinate plot, with a focused view (−35 < PC < 70) of the first three principal components for each reagent (**A** and **B**): unexecuted reactions (grey); and executed reactions (coloured, with: light blue, unproductive; yellow, productive but not hits; green, tubulin hits; purple, other hits). **D** PCA of morphological fingerprints (M_PC1-3) of screened products at 10 µM in the cell painting assay: yellow, inactive; green, hits with >70% similarity to tubulin cluster; and purple, hit with <70% similarity to any defined functional cluster.

Following an integrated experimental pipeline (Fig. 4A), the selected reactions (250 µL scale) were robotically executed in vials in 96-well format via sequential addition of methanolic stock solutions of catalyst (34 µL of 0.03 M solution), CsOPiv (66 µL of 1.5 M solution), alkene/alkyne co-substrate (75 µL of 0.67 M solution; limiting reagent) and hydroxamate ester substrate (75 µL of 0.73 M solution), followed by stirring for 17 h. Four control reactions, involving *N*-(pivaloyloxy)benzamide (**S1_11-Hy**) and four known co-substrates (**Co1_26**-**Co1_29**, see Supplementary Information), were also executed on the same plate. The reaction outcomes were then analysed using an Ultra-Performance Liquid Chromatography (UPLC) system equipped with an evaporative light-scattering (ELS) detector, enabling estimation of the yield of each product (Fig. 4B)^59,60^. We used 15% estimated yield as a threshold for mass-directed purification based on our previous experience^60^ of obtaining sufficient material (ideally ≥0.5 mg) for gravimetric quantification; a higher threshold may have excluded identified hits and affected batch design in the second round (see Supplementary Fig. 6). The dried isolated products were weighed using an automated balance. Of the 80 executed reactions, 28 (35%) were identified as productive (i.e. leading to products in >15% estimated yield). All selected hydroxamate ester substrates reacted productively with at least one co-substrate; however, whilst some co-substrates participated in several successful reactions, others (such as **Co1_4**-**Co1_6**) did not. Finally, the products were plated as 10 mM solutions in DMSO using a liquid handler ready for phenotypic screening. To capture the structural diversity of the experimentally executed reactions, a parallel coordinate plot was generated using the high-dimensional molecular fingerprinting data (Fig. 4C). We found that this representation preserved similarity/dissimilarity more effectively than alternative representations such as two-dimensional UMAP (Uniform Manifold Approximation and Projection for Dimension Reduction) and t-SNE (t-distributed Stochastic Neighbour embedding)^61,62^. The plot focuses on the first three principal components (S_PC1-S_PC3) of both reagents (A and B). The lines represent the structural variation in reagent A (first three axes) and reagent **B** (final three axes), with the full trajectory capturing each pairwise combination (i.e. reaction). The 80 sampled combinations, highlighted in colour, illustrate the broad coverage of the 128 × 128 combination space.

The isolated products were screened at 10, 30, and 50 µM in the cell painting assay performed on U2OS cells which employs six dyes to stain various cellular compartments, and uses high-content imaging and automated analysis to extract 579 morphological features^49,50^. Changes in these features (with median absolute deviation >3) relative to DMSO control were used to generate morphological fingerprints for the screened compounds. At 10 µM, five compounds displayed substantial changes in >5% of the features (i.e. induction value > 5%) and were considered hits (Fig. 4C, D and Supplementary Information). All hits were non-toxic, as assessed by cell viability counts in the cell painting assay. The morphological fingerprints of these hits were compared to the profiles of mechanism-of-action (MoA) clusters based on defined subsets of the morphological features^63^. Four of the hits (**R1-1** to **R1-4**; green, Fig. 4C) were >70% similar to the defined tubulin cluster, while one had <70% similarity to any of the defined functional clusters (purple, Fig. 4C). Notably, **R1-1** and **R1-2** track each other closely in the parallel coordinate plot (Fig. 4C), reflecting the similarity of the corresponding substrates and co-substrates.

### Subsequent round(s) of molecular discovery

A scoring system was developed to enable the phenotype-driven design of further batches of reactions for execution. Our approach drew on both the activity and functional cluster similarity of all previously tested combinations. To drive the development of hit series with similarity to the tubulin functional cluster, we assigned scores, for all three screened concentrations, to substrate/co-substrate combinations:

- Induction <5% or <70% tubulin cluster similarity: score 0.
- Induction >5% and >80% tubulin cluster similarity: score 1.
- Induction >5% and 70–80% tubulin cluster similarity: score between 0 and 1 (in 0.1 steps).

These criteria were informed by previously-established practices with the cell painting assay^15,50,51,60,63,64^ in which induction ≥5% has been used to identify compounds with significant morphological effects; in addition, a hard cut-off for cluster similarity was deliberately avoided, with the criteria informed by the establishment^63^ of morphological subprofile analysis, and its exploitation^64^ in identifying tubulin-targeting compounds (see also Supplementary Fig. 20 for comparison with alternative scoring schemes). These scores were summed then batch-normalised to a 0–1 scale to give a Combination Score. Nine of the tested combinations were assigned non-zero scores. These scores were then propagated to substrate/co-substrate combinations within an expanded virtual subset of 134 × 134 combinations; the expanded subset incorporated, in a cost-effective manner, additional reagents **A** and **B** that were similar^65,66^ to those that had been used in the reactions that had yielded hits (see Supplementary Information). The formula used the Jaccard similarity (*J*(*x,y*)) to quantify, on a 0-1 scale, the structural similarity of reagent pairs based on their MHFP6 fingerprints (*A*_*x*_*B*_*x*_ for pair x; *A*_*y*_*B*_*y*_ for pair y) (Eq. (1)):

1 $$J\left(x,\,y\right)=\frac{|{A}_{x}{B}_{x}\bigcap {A}_{y}{B}_{y}|}{|{A}_{x}{B}_{x}\bigcup {A}_{y}{B}_{y}|}$$

Here we used the concatenated fingerprints of the reagent pairs, but the user could choose to use product fingerprints if a reaction class with a predictable outcome had been used. However, applying MHFP6 to fully enumerated products would cause a dramatic expansion in molecular shingles, significantly increasing computational time per iteration (estimated at >12 h GPU in this case). We also chose not to use fast bit‑based product fingerprints (e.g., Morgan) which lose the substructure‑level resolution that motivated the use of MHFP6, opting therefore for maximising information while maintaining tractability.

The propagated score for each combination was obtained by summing the contributions from all hits (Eq. (2)). The choice of the exponent (here, power 3) is user-definable, and reflects the extent to which structural similarity to previously identified hits is prioritised; for example, a higher exponent (e.g. 4) would further accentuate high similarities.

2 $${{Score}}_{x}= {\sum }_{n=1\,}^{n={no}.\,{hits}}{{Score}}_{{hit}{\_}n}\times {J\left(x,\,{hi}{t}_{n}\right)}^{3}$$

**Fig. 5:**
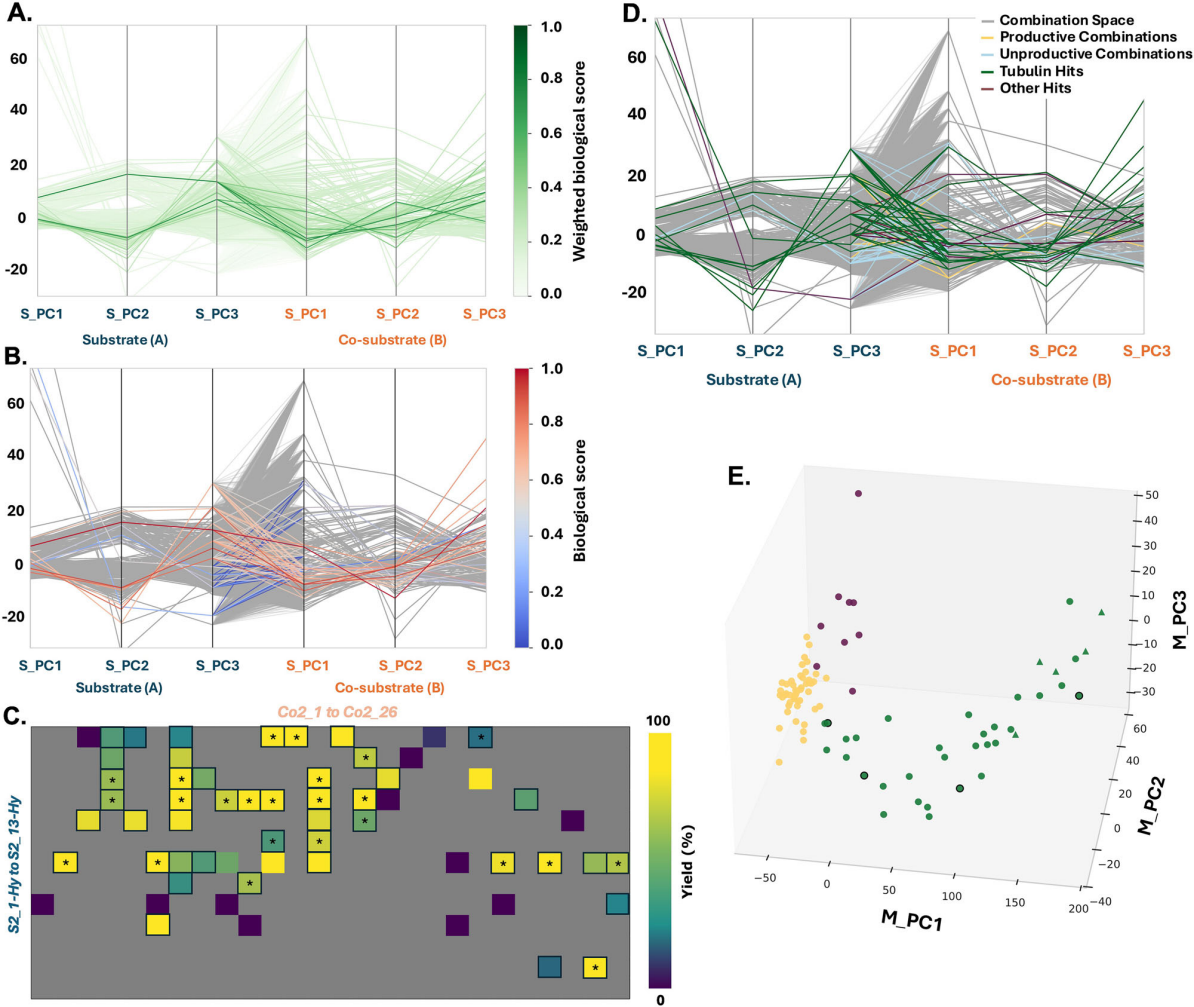
Second round of molecular discovery. **A** Propagation of scores (i.e. based on similarity to round 1 combinations affording hits) to the expanded 134 × 134 virtual subset visualised on a parallel coordinate plot (Y-axis focused on −35 to 70 for clarity). Line colour indicates propagated score; the first three principal component contributions of each reagent (**A** and **B**) are shown sequentially. **B** Selected combinations, colour-coded with a cool-to-warm gradient based on propagated scores (see above), showing a balance between exploitation and exploration. **C** Heat map of analytical ELS-determined yields for the executed reactions: grey, not executed; boxes = successful product isolation; asterisk = multiple products. **D** PCA of structural MHFP fingerprints (S_PC1-3) for substrate/co-substrate combinations shown on a parallel coordinate plot, with a focused view (−35 < PC < 70) of first three components for each reagent (**A** and **B**): unexecuted reactions (grey); and executed reactions (coloured, with: light blue, unproductive; yellow, productive but not hits; green, tubulin hits; purple, other hits). **E** PCA of morphological fingerprints (M_PC1-3) of screened products at 10 µM in the cell painting assay: yellow, inactive; green, hits with >70% similarity to tubulin cluster; and purple, hits with <70% similarity to any functional cluster; outlined green circles indicate validated tubulin hits from round one.

Although the approach was applied using cell painting assay data, it could, in principle, be adapted for other phenotypic, or indeed target-based, assays. The propagated biological scores enable identification of promising substrate/co-substrate combinations for investigation; on the parallel coordinate plot, highly-scored combinations track those that had yielded tubulin hits in the first round (compare Fig. 4C with Fig. 5A). To navigate the combination space, and design potential batches of reactions for execution, a compound algorithm was used. First, a bounce sampling algorithm alternated the selection of reagents **A** and reagents **B** with the highest scores based on the previous selection. Then, a MaxMin algorithm was applied to select diverse combinations to complete the batch. This balanced the exploitation of the identified promising regions with the exploration of previously unexplored regions. Here, 100 batches, each containing 80 potential reactions, were designed, with a 50:50 balance between exploitation and exploration, and minimisation of costs. From these, the batch with the lowest overall estimated cost was selected for execution. Notably, combinations within this chosen batch had both high propagated scores (red) that align with previously identified regions, and low propagated scores corresponding with novel regions (blue) (Fig. 5B).

The execution of this batch was accompanied by a significant increase in reaction productivity, with 67% of the reactions leading to products in >15% yield (compared with 35% reaction productivity in the first round) (Fig. 5C). The productive reactions tended to reside in regions with higher propagated scores (Fig. 5D), and 60% of these yielded hits with >70% tubulin cluster similarity. Overall, 32 additional tubulin hits were discovered in round 2, along with the 4 tubulin hits from round 1 that had been validated through resynthesis and rescreening (Supplementary Information and Fig. 5E). Interestingly, five of the hits with >70% tubulin cluster similarity displayed an even higher similarity to the HDAC functional cluster. Finally, there were 9 products stemming from combinations that were active (induction >5%) but with <70% similarity to all of the defined functional clusters (Fig. 5D, E).

### Hit validation

**Fig. 6:**
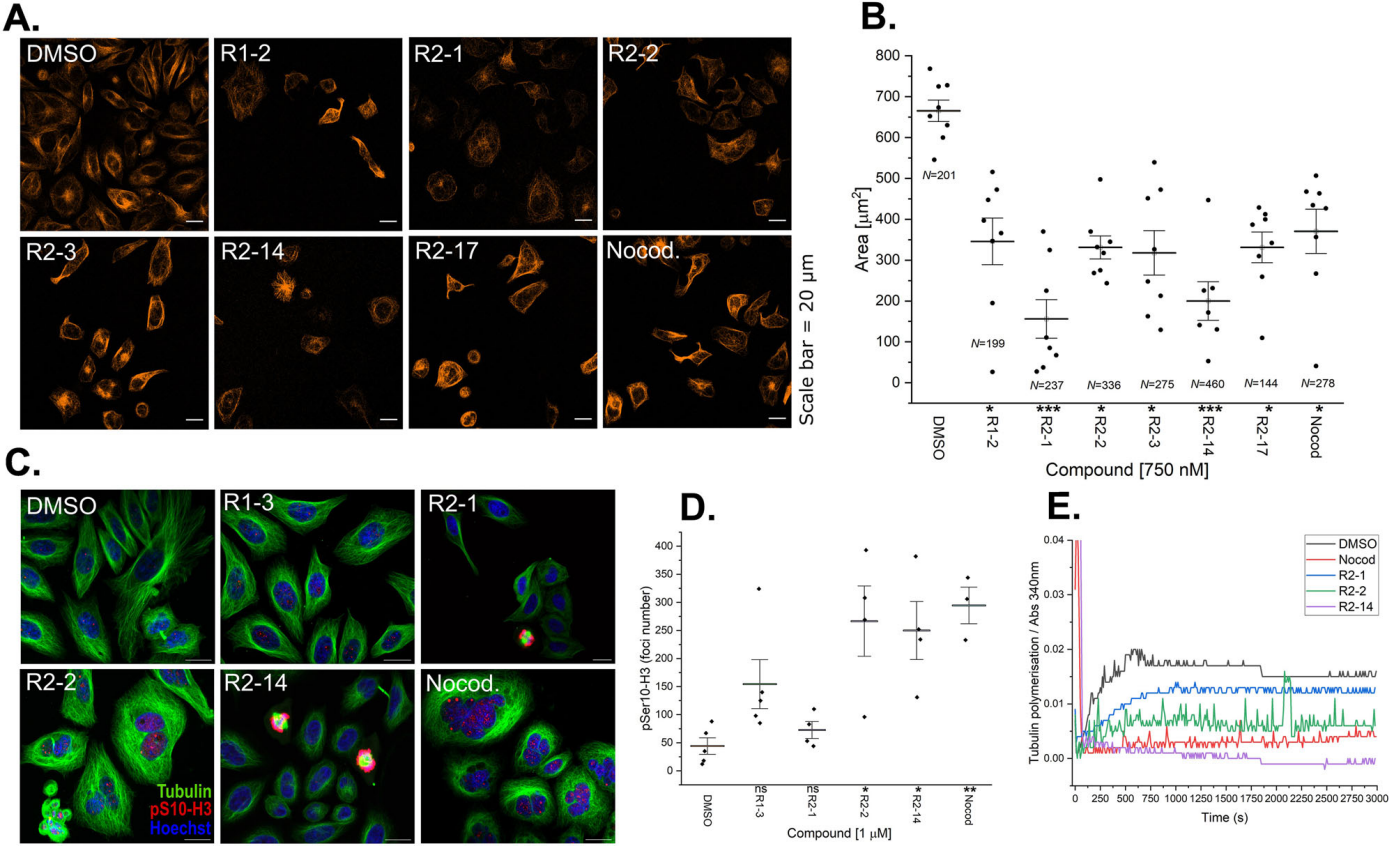
Validation of identified tubulin hits. **A** Fluorescence microscopy for microtubule (MT) labelling using the SPY555-tubulin dye on live HeLa cells following a 24-h treatment with DMSO (negative control), 750 nM nocodazole (positive control) or 750 nM selected products. **B** Median stained area per cell after image segmentation (4 fields/well, *n* = 2 biologically-independent wells); bars depict the mean and standard errors of these medians, and *N* indicates the number of cells analysed. Significance compared with DMSO control was assessed by one-way ANOVA and Tukey’s test (*P < 0.05, **P < 0.01, ***P < 0.001). **C** Immunofluorescence images showing α-tubulin (green), phosphorylated histone H3 (pSer10-H3, red), and DNA (blue) staining after 24-h compound treatment (1.0 μM). **D** Mitotic arrest quantified by nuclear pSer10-H3 foci number. Individual points represent the number of foci in each imaged field (≥4 fields per compound); bars indicate the mean and standard error with statistical significance determined using one-way ANOVA (*P* values depicted as above). **E** Tubulin polymerisation was monitored spectrophotometrically in vitro (Abs 340 nm, 1.5 μM compound), with nocodazole used as reference.

To validate the tubulin-targeting activity of our round 2 hits, we examined their effects on cytoskeletal integrity using immunofluorescence and morphological profiling (Fig. 6). Before testing, compound purity was confirmed by re-analysing the original stocks of isolated products by ¹H NMR spectroscopy; this enabled identification of 19 compounds with excellent purity (>90%) for downstream analysis. We stained HeLa cells with the live-cell α-tubulin probe SPY555-tubulin to assess the effects of compounds at lower concentration (750 nM) than the original screen (10, 30 and 50 μM) (see Supplementary Fig. 13 for assay optimisation). To quantify the effects (Fig. 6 and Supplementary Figs. 14–16), we measured stained areas across four fields in duplicate; this metric was selected as a sensitive indicator of changes in cell shape and spreading. Within each field, individual cell measurements were collected, and the median area was used for plotting and statistical evaluation. Statistically significant effects, compared to the DMSO control, were used to shortlist compounds for further validation studies. Notably, two hits from round two (**R2-1** and **R2-14**), unlike **R1-2** from round one, still had a statistically-significant effect on stained area at lower concentration (150 nM) (Supplementary Figs. 17, 18).

The four shortlisted compounds (**R1-2,** **R2-1,** **R2-2,** **R2-14**) were then tested for their ability to induce mitotic arrest in HeLa cells. It has been previously noted that HeLa cells are better suited to immunofluorescence studies of mitotic arrest than the U2OS cells that were used in the cell painting assay^64,67^. We quantified the labelling of the mitotic biomarker phospho-histone H3 (pSer10-H3) on fixed cells^68^. HeLa monolayers were treated with our hit compounds (1.0 μM) for 24 h and then labelled. The selected compounds induced phenotypes consistent with mitotic arrest that are similar to well-characterised mitotic arrest agent nocodazole^64^. Quantification of pSer10 H3 labelling revealed that **R2-2** and **R2-14** significantly increased mitotic arrest compared to DMSO (Fig. 6D). Finally, to demonstate target engagement, we evaluated selected compounds in an in vitro tubulin polymerisation assay; **R2-2** and **R2-14** were found to exhibit similar effects to the nocodazole control (Fig. 6E).

## Discussion

**Fig. 7:**
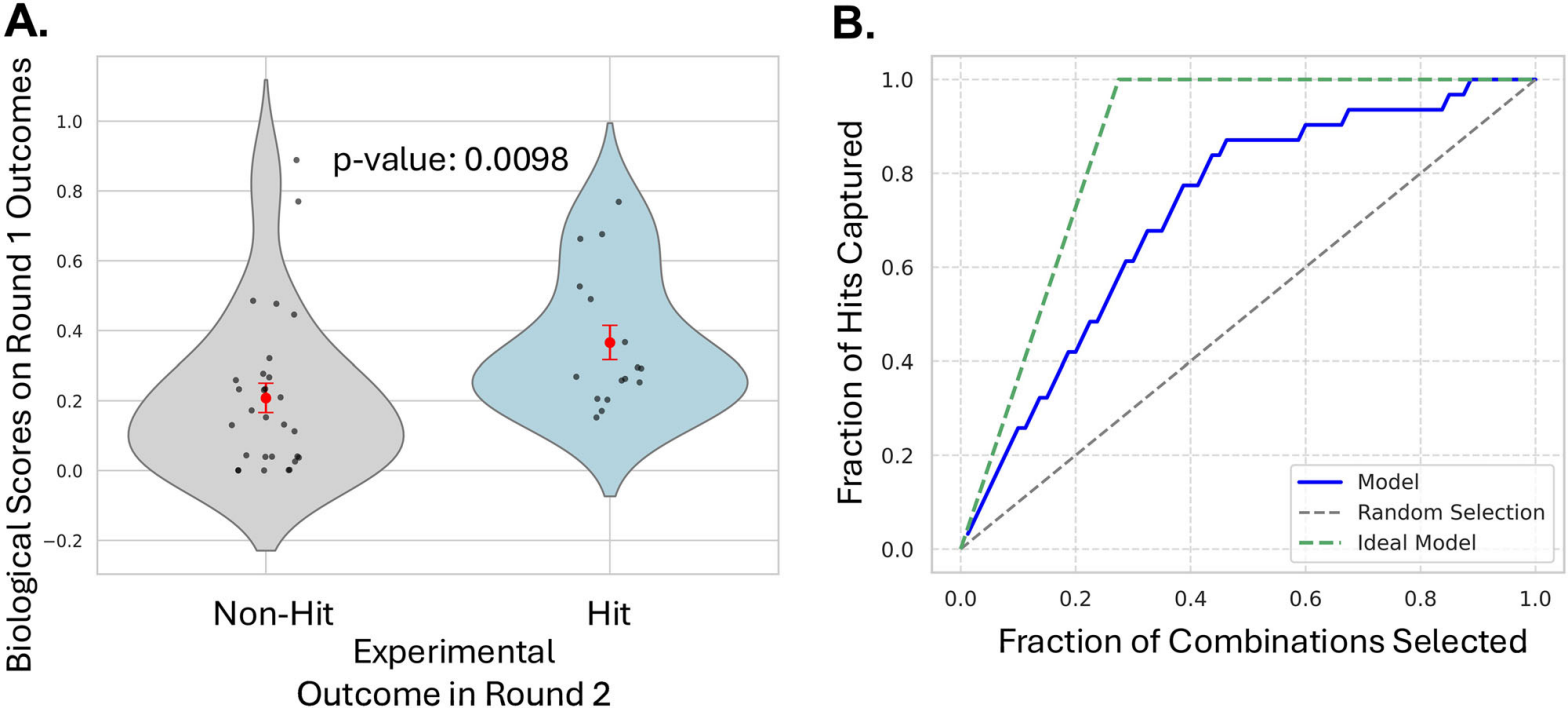
Analysis of the effectiveness of scoring based on round 1 hits. **A** Violin plots showing the distribution of propagated scores (i.e. based on similarity to hit combinations identified in round 1) against experimentally determined round 2 outcomes (tubulin hit *vs.* non-hit). Red bars indicate means and standard errors. Statistical significance was assessed by an independent two-sample *t*-test (see text). **B** Cumulative gain curve showing the enrichment of combinations that yield tubulin hits as a function of the fraction of top-scored combinations.

In this study, we had aimed to realise a data-driven, automated closed-loop approach in which bioactive molecular discovery was driven by phenotypic effects alone. The approach was expected to enable identification of synthetically accessible, biologically relevant space. The design of batches for execution in round 2 was driven by scores that were based on the activity of products from round 1. To assess the performance of the scoring, propagation and reaction design, we compared the propagated biological scores assigned after the first round of discovery, with the experimental results subsequently obtained in the second round (Fig. 7A). The difference between the propagated scores for combinations that did, and did not, yield hits in round 2 was statistically significant, as shown by an independent two-sample t-test (t = 2.45, one-tailed p = 0.0098). This finding validated the ability of our scoring scheme to guide the selection of combinations for investigation. Hit compounds from round 1 had necessarily stemmed from substrate/co-substrate combinations that yielded products. We therefore wondered whether our scoring would prioritise combinations that correspond to productive reactions. The scores for combinations found to be productive in round 2 were indeed significantly higher than for those that did not yield products (Supplementary Fig. 22). Nonetheless, the proportion of productive reactions in round one, under one set of reaction conditions, was quite low (35%). It has been shown, in a discovery programme driven by aminations of aryl bromides^36^, that the use of alternative reaction conditions can significantly increase reaction productivity and, hence the chemical space that may be explored. The incorporation of alternative reaction conditions, or even reaction optimisation, may be a fruitful future direction for expanding accessible chemical space for phenotype-directed molecular discovery. Moreover, the diversity of readily-explorable chemical space could also be expanded through development of a broader range of operationally simple connective reactions that enable diverse chemotypes to be prepared.

We determined the proportion of the hit combinations that were captured in the top-ranked combinations that were investigated in round 2 (Fig. 7B). Our approach clearly outperforms the random selection baseline, and compares well with the ideal model in which all of the combinations that yielded hits have higher scores than those that did not. Notably, 80% of all round 2 hits are found in the top 42% of the ranked combinations (compared with 80% for random selection and 22% for an ideal model). This indicated that the scoring prioritised promising combinations, enabling our algorithm to drive the design of reaction batches that explored biologically relevant chemical space.

The effectiveness of the approach was showcased through the expansion of hits with >70% similarity to tubulin cluster in the cell painting assay from 4 hits (in round 1) to at least 19 validated hits (in round 2). Of these hits, 25% had, significant effects on the median stained microtubule area per cell at 750 nM. In addition, by assaying at lower concentration (150 nM), we showed that two hits from round 2 were significantly more potent than those obtained in from round 1. Selected hit compounds were shown to induce mitotic arrest and, through a polymerisation assay, to directly engage with tubulin.

**Fig. 8:**
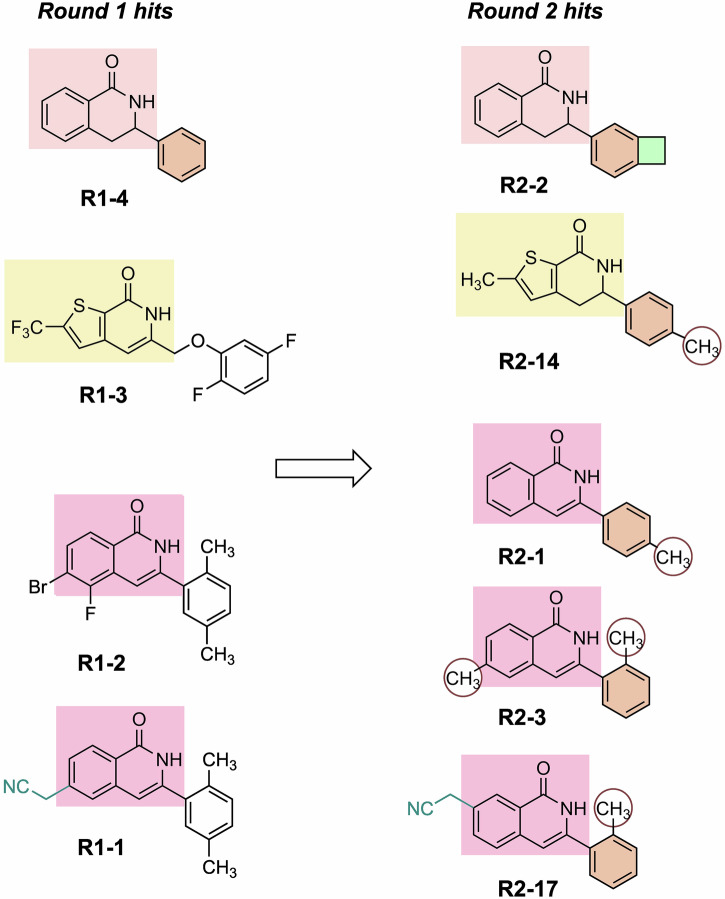
Structural elucidation of hits with validated tubulin-modulating behaviour. The evolution of hits between the discovery rounds is shown. Round 2 hits incorporate scaffolds and motifs from round 1 hits, refined, repositioned or linked in different ways.

We retrospectively analysed the structural evolution of hits between round 1 and round 2 (Fig. 8). Most hits contained either an isoquinolinone or dihydroisoquinolinone core, highlighting their importance for modulation of tubulin; these cores were present in **R1-1,** **R1-2** and **R1-4** (from round 1) and were preserved in **R2-1,** **R2-2,** **R2-3**, and **R2-17** (from round 2). Whilst known tubulin modulators include isoquinolinones^69^ and natural product-inspired isoquinolines^70^, the 19 validated hits have expanded the structural and skeletal diversity of this class of functional compounds. Round 2 compounds had been refined from round 1, for example through substituent introduction (e.g. methylation) and repositioning (e.g. of a cyanomethyl group; **R1-1** → **R2-17**). Notably, the hit **R2-14** merged structural features from both **R1-3** (its thiophene-fused scaffold) and **R1-4** (its side chain), illustrating how additive scoring drove the merging of fragments found in more than one parent compounds; in this case, this resulted in a non-trivial scaffold swap.

Our approach requires a basis for scoring hits, and we scored on the basis of similarity to a reference phenotype (the tubulin functional cluster). We did, however, also discover some hits that were dissimilar to all defined functional clusters and which may be of particular interest: the application of our approach to discover such compounds explicitly would require an appropriate scoring scheme, for example that rewarded dissimilarity to all defined functional clusters or reference compounds.

### Summary

We have demonstrated, for the first time, the feasibility of data-driven, automated closed-loop molecular discovery and optimisation driven by phenotypic effects alone. Our approach has the potential to be general: it could be adapted to exploit any reaction class that connects building blocks, and be applied to any high-throughput assay (either phenotypic or target based). To exemplify the approach, we exploited Rh-catalysed annulation reactions (which could potentially yield multiple isomeric products) and the cell painting assay (which can identify compounds that induce morphological effects). The design of the reaction batches in both rounds of discovery was algorithmically driven. Subsequently, all aspects of the execution of selected reaction batches (i.e. set-up, quantitation, purification and plating) were fully automated. Iteration of the discovery loop was demonstrated, and enabled expansion of the number, and improvement of the potency of, hit compounds; here, structural optimisation was achieved through (algorithm-driven) modification of the substitution, and merging of the structural features, of hits obtained in round 1. Overall, we have established a framework for the discovery of synthetically accessible bioactive compounds that induce specific phenotypic effects. We envisage that our approach, driven by other phenotypic assays with appropriate scoring criteria, may enable the discovery of classes of chemical probes of biological systems that have the potential to reveal mechanistic insights and, ultimately, open opportunities for drug discovery.

## Methods

### Automated reaction array assembly

Reaction screening was conducted on a Zinsser liquid handling platform using algorithmically generated worklists of non-exhaustive hydroxamate/alkene–alkyne combinations. Stock solutions of catalysts, bases, and substrates were prepared in methanol at appropriate concentrations to ensure dissolution and allow uniform dispensing across 96-well glass vials. Each reaction was carried out on a 0.05 mmol scale of hydroxamate substrate (limiting reagent) in a total volume of 250 µL, with [Cp*RhCl₂]₂ (2 mol%), CsOPiv (2 equiv), and alkene/alkyne coupling partners (1.1 equiv). The corresponding solutions were dispensed sequentially by the robot into individual 1 mL glass vials positioned in a Zinsser 96-well metal block. The vials were sealed with PTFE-lined caps and agitated at ambient temperature for 17 h. Following reaction, the solvent was evaporated under reduced pressure, and crude residues were redissolved in DMSO for analysis. Full stock preparation protocols and dispensing orders are provided in the Supplementary Information.

### Calibration and quantification

Analytical quantification was performed by UPLC coupled with photodiode array (PDA) and ELS detection. Calibration curves were constructed from a multi-analyte standard set spanning log concentration ranges, with three-dimensional calibration surfaces derived from retention time and ELS detector response (*R*² = 0.998). This calibration strategy ensured quantification accuracy within 10% for test compounds of 250–400 Da. Crude reactions were sampled using a Hamilton liquid handler, diluted five-fold in DMSO, and analysed under gradient elution (MeCN/H₂O with 0.1% formic acid). Product yields were estimated using the calibration model. Instrument parameters and calibration surfaces are described in the Supplementary Information.

### Product purification and normalisation

Reactions with higher than 15% estimated product yield were purified by mass-directed preparative HPLC under standard acidic gradients. Isolated fractions were tracked and processed using an HTZ Selecta robotic workstation equipped with integrated balances and barcode tracking. Dried fractions were reweighed, and plated as 10 mM solutions in DMSO for biological assays. Excess material was archived in deep-well plates. Full HPLC conditions and robotic handling workflows are described in the Supplementary Information.

### Cell painting assay

Phenotypic profiling of U2OS cells was performed as described by Bray et al.^49^, with adaptation for automated screening. Cells were seeded into 384-well plates, treated with test compounds at 1–10 µM for 20 h, and subjected to multiplexed staining with fluorescent dyes marking nuclei, mitochondria, actin, and membranes. Plates were imaged on a Molecular Devices Micro XL high-content imaging system (20× objective, 9 sites/well, five channels). Images were analysed with CellProfiler (v3.0.0), extracting 1716 features per site, subsequently aggregated at the well and replicate level. Robustness filtering, following Woehrmann et al.^71^, identified 579 features consistently reproducible across reference replicates. Phenotypic signatures were expressed as *Z*-scores relative to DMSO controls, calculated using median absolute deviation. Induction values, defined as the percentage of significantly perturbed features, were computed per compound. Similarities between profiles were quantified using correlation distance. An illustration of phenotypic similarity between representative compounds is shown in Supplementary Fig. 10. Full staining formulations, dye sources, and imaging settings are provided in the Supplementary Information.

### Computational workflows

A KNIME pipeline was developed to curate commercially available compound libraries according to user-defined filters for functional group content, substructural alerts, and physicochemical developability. Input structures were imported in SMILES format, filtered through rule-based descriptors, and exported as curated libraries for subsequent screening. To design reaction arrays, a custom Python algorithm was developed to design hydroxamate + alkene/alkyne combinations under constraints of diversity and cost. The algorithm accepts substrate inventories as input, enumerates potential combinations, and applies a non-exhaustive selection strategy optimised for chemical space coverage. Worklists were output in CSV format for direct upload to the Zinsser platform. All workflows and scripts are described in detail in the Data availability section.

### In-cell microtubule imaging

HeLa cells were seeded in 96-well plates, treated with compounds for 24 h, and stained with a live-cell tubulin probe and Hoechst nuclear dye. Imaging was performed using a Zeiss LSM 980 confocal microscope (20× objective), with four fields acquired per well. Images were segmented using ZEN Intellesis, with features aggregated per replicate.

For immunofluorescence assays, HeLa cells were seeded on coverslips, treated with compounds (1 µM), fixed, permeabilised, and stained with anti-α-tubulin and anti-H3S10p primary antibodies, visualised with fluorescent secondaries, and counterstained with DAPI. Imaging was performed under confocal microscopy (40× objective), and foci were quantified. Detailed staining formulations and antibody dilutions are provided in the Supplementary Information.

### Tubulin polymerisation assay

Tubulin polymerisation was monitored spectrophotometrically at 340 nm in BRB80 buffer with GTP induction, using purified tubulin. Test compounds were added at 10 µM, and turbidity changes were monitored for 1 h. Experimental details on tubulin preparation and assay setup are provided in the Supplementary Information.

### Statistical analysis

All experiments were performed with at least 3 independent biological replicates, and representative data are shown unless otherwise stated. Data are presented as mean ± s.e.m. (or s.d. where indicated in figure legends). For comparisons among more than two groups (e.g., compound treatments vs. controls), one-way ANOVA followed by Tukey’s *post hoc* test was applied. For direct comparisons between hit and non-hit compounds, two-tailed unpaired *t*-tests were used to calculate *P* values. Exact *P* values are reported in figure legends where possible; significance thresholds were set as *P* < 0.05 (*), *P* < 0.01 (**), *P* < 0.001 (***). No samples or data points were excluded, and variances were similar between groups being compared.

### Reporting summary

Further information on research design is available in the Nature Portfolio Reporting Summary linked to this article.

## Supplementary information

Transparent Peer Review file

Supplementary Information

Life sciences reporting summary

## Data Availability

All data supporting the findings of this study are available within the article and its Supplementary Information files. Raw and processed datasets, together with all analysis workflows, filtering scripts and Jupyter notebooks for data visualisation are openly available at: 10.5281/zenodo.19736513. The PlatePicker Python package enabling array design guided by phenotypic data is also available at: 10.5281/zenodo.19735945. Datasets for the cell painting assay, imaging experiments (and imaging analysis) and tubulin polymerisation are available on Zenodo: 10.5281/zenodo.17405960.

## Code availability

All code used in this study is available at: 10.5281/zenodo.19735945 and 10.5281/zenodo.19736513.
