# Supplementary material for: Algorithm-driven, phenotype-directed bioactive molecular discovery
Source: Commun Chem. 2026 May 16;9:267. doi: 10.1038/s42004-026-02066-8 (PMC13439441; doi:10.1038/s42004-026-02066-8)
Supplement: Supplementary file 1 [file 42004_2026_2066_MOESM1_ESM.pdf]

nature portfolio

Peer Review File

Algorithm-Driven, Phenotype-Directed Bioactive Molecular Discovery

Corresponding Author: Professor Adam Nelson

This file contains all reviewer reports in order by version, followed by all author rebuttals in order by version.

Attachments originally included by the reviewers as part of their assessment can be found at the end of this file.

Version 0:

Reviewer comments:

Reviewer #1

(Remarks to the Author)
In this manuscript, the authors describe an iterative, algorithm-driven parallel medicinal chemistry approach to ligand discovery based on a phenotypic assay readout. Although such approaches are well-precedented in target-based drug discovery, applications in phenotypic drug discovery are not well represented in the literature.
In the present study, the authors describe the use of a Rh-catalyzed C-H annulation reaction to synthesize diverse libraries of (dihydro)quinolones that were then tested in a cell painting assay. Those compounds that exhibited notable changes in morphology were then clustered based on a fingerprint analysis and compared to literature compounds with known mechanism-of-action. One cluster mapped with high similarity to the activity of known tubulin modulators and was explored via a second round of library synthesis in which compound selection was algorithmically driven based on molecular similarity to hits from the first round. This second set of compounds was found to be enriched in hits that displayed a similar tubulin inhibitory phenotype. Selected hits were then validated in orthogonal biochemical and cell-based assays for tubulin polymerization inhibition and cell cycle arrest. The result was an increased efficiency in identification of isoquinolone-based tubulin modulators.
Overall, this is a solidly designed study, and the authors clearly and logically lay out the rationale of the workflow, the results obtained and the learnings. The Supporting Information is sufficiently detailed from both an experimental and computational perspective, and there is adequate characterization data for key compounds. Having said that, there are a few aspects of the study that could benefit from further explanation and/or development:
1. Although it is laudable that the authors are using an uncommon C-H annulation reaction to construct their library, the enumerated virtual design space (850 x 354 coupling partners) is rather small, perhaps due to the relatively limited commercial availability of alkynes and alkenes as building blocks. By comparison, standard high-throughput phenotypic screening campaigns using pre-synthesized compound collections often number in the tens to hundreds of thousands. This relative lack of chemical diversity could ultimately limit the hit rate and utility of a phenotypic screen. A visualization of the virtual design space as well as the compounds actually synthesized in comparison to a standard collection of drug-like compounds like CheMBL would be helpful here.
2. This reviewer also found it questionable that building block cost was used as a selection criterion to pick representatives from each cluster for synthesis. Acknowledging the practical limitations of conducting library synthesis in an academic environment and on a limited budget, the potential bias this selection criterion could introduce into the library should be considered. For example, one could imagine that building block cost might correlate with complexity and drug-like physicochemical properties. Similar to the previous comment, a visualization of the targets selected for synthesis versus those in the total virtual design space of drug-like property space would provide some context here.
3. Given the somewhat small size of the library (80 compounds, 0.4% of the 128 x 128 clustered matrix) and the rather low synthetic success rate of the Rh-catalyzed annulation reaction (35%), the productive combinations (green and yellow lines in Figure 4C) represent only a small fraction of the combination space, with significant regions of the Substrate A PC1 and PC2 and Substrate B PC1 property space being poorly sampled. While one could make the counter-argument that the screen did yield hits with a phenotype of interest (albeit via a well-known mechanism), if the point of a phenotypic screen is to uncover new biology, maximizing chemical diversity should be one of the goals. In this light, it is surprising that the authors did not attempt to optimize failed reactions from either round of the library synthesis. As it stands, it seems fair to say that the chemical space coverage is highly biased / limited by the synthetic scope of the one set of reaction conditions that were applied.

4. An analysis of the cell-painting assay morphological fingerprints of the five hits from the round 1 screen showed that four mapped with high similarity to the tubulin cluster, while the fifth seemed to elicit a distinct phenotype. In light of the statement that “hits that have <70% similarity are likely to have mechanisms that are distinct from all of the defined functional clusters, and may be of particular interest”, it is puzzling that the authors chose in round 2 of the library to bias designs toward the tubulin cluster, which is well-known biology. This choice seems to undercut the primary premise of phenotypic screening, since if the goal were to screen for tubulin modulators that would be a much easier task in a target-directed screening approach. A fuller explanation of this decision in the manuscript is warranted.
5. Along the lines of the previous comment, no mention is made in the manuscript of any follow-up work done on the functionally active hits from Round 1 and 2 that did not map to the tubulin cluster. This is indeed puzzling given the statement in the introduction that one advantage of phenotypic screening is to avoid being “biased towards previously-identified protein targets” which in this case appears to be tubulin.
Overall, this is a solid study, but given the limitations described above, this reviewer does not see this article as being highly influential to the field.

Reviewer #2

(Remarks to the Author)
I co-reviewed this manuscript with one of the reviewers who provided the listed reports. This is part of the Communications Chemistry initiative to facilitate training in peer review and to provide appropriate recognition for Early Career Researchers who co-review manuscripts.

Formatting of the review has been lost in this text box: I upload the Word doc

Overall Assessment
We were deeply impressed by the content of the manuscript and found it a genuine pleasure to read. The work is conceptually exciting, thorough, well-structured, and both scientifically interesting and sound. The manuscript outlines an elegant experimental design where the integration of automated synthesis, phenotypic screening and iterative algorithmic re-design are beautifully exemplified, in a closed-loop manner. From the experimental section it is clear that the experimental execution of this beautiful proof-of-concept is excellent.
The authors comment that as molecular diversity is dominated by a small number of robust synthetic reactions, and that this has contributed to uneven historic exploration of chemical space. We strongly agree with this assessment. Whilst the work outlined in this manuscript enables a level of chemical space scoping (as beautifully illustrated in figure 3) hard to reach chemical space still remains unexplored. It would be good for the authors to iterate this in their conclusion, and emphasise the volume of synthetic discovery research that still remains paramount.
Major Comments
None, though the actual diversity of chemical space is not so wide, and the authors must mention this in the conclusions. It is an exciting starting point for this concept, but there is much work required by a community to enable fully diverse chemical space to be accessed.

Minor Comments
1. The presentation of the statistical rigor could be improved, (for example see points A, B, and C), however we believe that the authors probably have these data and that these points should be readily addressed.
A. The yield threshold (15%, on page 6) is simply stated, and as such seems arbitrary. We are sure that the authors will have thought carefully as to why to set this at 15%. They should justify this in the text (why not 5%, why not 20%, or 40%). The setting of the threshold will promote bias toward certain chemotypes. If the threshold is set too high, low-yielding but potent products are excluded (again diminishing access to diverse and relevant chemical space). It would be good to also discuss the bias that selection, and threshold setting can introduce.
The rational behind the setting of the following thresholds should also be discussed, with appropriate detail, and its impact on potential outcomes/bias briefly outlined:
Thresholds for “hit” definition. Page 8
Induction >5% and >80% tubulin cluster similarity: score 1.
Induction >5% and 70-80% tubulin cluster similarity: score between 0 and 1 (in 0.1 steps).
Thresholds 80% 70-80% . Sensitivity to parameter choice should be discussed.
"Hits that have <70% similarity are likely to have mechanisms that are distinct from all of the defined functional clusters": please justify this statement in the manuscript.

B. Small error: Filters at Figure 3A and Supplementary Table 11 do not match, moreover the table filter is not full.
Property Filter 1
Figure 3A (group A) Filter 1
Figure 3A (group B) Filter 2
Supplementary Table 11
Filter Condition | Include if
Heavy atoms 6≤HA≤13 6≤HA≤14 <6 | TRUE
>14 | TRUE
XLogP -0.5≤XLogP≤3.5; -1.5≤XLogP≤3.5; < −1.5 | TRUE
>3.5 | TRUE
Rotatable bonds
(non-terminal) ≤2 ≤2 >2 | TRUE
Aromatic atoms 5≤AA≤12 Not constrained Not constrained

These filters are logically opposite.
Again the selection of filters for group A and B, in figure 3A (and throughout the experimental design) should be discussed.
C. Algorithm development. Was the approach compared with a more simplistic algorithm? Is it possible that your algorithm is more complex than it need be? It would be really good to exemplify how your approach out-performs the standard simple models, where random, yet chemical sensible selection is implemented. Showing this as a baseline would be really great. You probably have this work already, and just need to embed it into the manuscript.
You have shown that the propagated scores correlate with round 2 hits and a statistical significance (t-test). However, this is a test of internal consistency. It does not compare the algorithm against alternative strategies.
Algorithm-guided round 2 was stated to have generated more hits, and higher productivity. Please include comparative data that you have of the performance of your algorithm vs the obvious alternatives that are commonly used. For example, the scoring/propagation scheme is not compared against: random sampling with yield bias, * or simple nearest-neighbour selection, ** or "Greedy similarity search".
2. A narrow chemical space is accessed and explored, despite the original positioning within the abstract .
Jaccard similarity on concatenated reagent fingerprints.
Using similarity of (A,B) pairs rather than predicted products: assumes a single reaction outcome. In the authors approach, similarity is set to track with the reagent similarity. The authors' hypothesis/assumption here isn't tested, validated or discussed. It is really important to reflect on this aspect.
Similarity(Reaction 1, Reaction 2) = Jaccard(Fingerprint(A1 + B1), Fingerprint(A2 + B2))
The use of Jaccard similarity on concatenated reagent fingerprints implicitly assumes that similarity at the reagent-pair level correlates with similarity of reaction products and thus similarity of biological phenotypes. This assumption is not discussed or validated and may not hold for reactions with multiple possible outcomes.
But same reagents ≠ same products, (unless there is only one way in which they can combine with a single regiochemistry/stereochemistry etc) Minor substituent changes can of course redirect C–H activation.

Other very minor points, that would help with expectation setting and clarity:

1. (from the abstract)
"phenotype-driven discovery can yield bioactive molecules with unexpected mechanisms of action, and open paths to first-in-class drugs"
a. Having this point in the abstract makes the reader anticipate that a new "first in class" drug will be identified: The tubulin mechanism is well known, strongly represented in Cell Painting reference clusters, rapidly inferred by similarity metrics
No truly unexpected MoA is shown.
b. If the sentiment were correct, the sentence should still be written as "reveal" not yield.

(from summary)
"may reveal new mechanistic insights"
Again, in this section, it should be explained that without appropriate threshold setting, the likelihood of finding a new mechanism of action is low.
2. Due to the very mixed readership that this manuscript will rightly attract, it would be helpful to be very clear on definitions. Often particular chemical scaffolds are referred to as targets. In the introductory sentences, elaborate: target centric-approaches (that focus on small molecule binding to a particular biomolecular target)

3. Red and green are used frequently in the figures, avoiding colour combinations associated with colour blindness will be helpful to some of the readership.

4. Potency claims are modest. No IC_{50}, EC_{50}, or selectivity data, no comparison to known tubulin binders beyond nocodazole. "Improved potency" claim is qualitative.

These are all minor points that we are sure that the authors will have considered and can readily address. Once these modifications are incorporated this will be a superb and timely contribution to the literature.

Reviewer #3

(Remarks to the Author)
This manuscript presents an end-to-end, closed-loop platform that couples algorithmic reaction selection, automated synthesis/purification, and phenotype-based screening (cell painting) to drive iterative discovery and optimization without requiring up-front structure elucidation. Specifically I have some major concerns as below:
1) A central concern is that the "phenotype-directed, structure-blind" framing is not fully realized in the current implementation. While the workflow avoids early structural elucidation of all products, the algorithmic propagation is performed on reagent-pair fingerprints rather than product fingerprints, and the readout heavily leverages similarity to a predefined tubulin morphological cluster. This makes the optimization loop partially dependent on prior knowledge embedded in the reference clusters and raises the question of whether the system is discovering novel phenotypes or mostly enriching toward a known mechanism-of-action signature.
2) Another major issue is the limited mechanistic depth around the discovered series. The tubulin conclusions are supported by follow-up imaging, mitotic arrest, and a polymerization assay for a subset of compounds, which is helpful. However, the screening concentrations in cell painting (10–50 μM) are high, and only a fraction of compounds show effects at sub-micromolar concentrations in the follow-up assay. More quantitative pharmacology may help strengthen the work.
3) From a methods and statistics standpoint, the manuscript would benefit from tighter clarity on replication and reproducibility across the closed-loop. Many steps involve multiple potential sources of variance, reaction yield estimation

via ELSD, automated purification and weighing, compound plating, and phenotypic profiling.
4) A key technical concern is whether the closed-loop is learning biology or learning chemistry productivity. The authors acknowledge that scores correlate with reaction productivity, which is practically useful, but it also risks conflating “high-yielding chemistry regions” with “bioactive chemistry regions.” The platform may preferentially select combinations that produce isolable products and thus bias discovery toward chemistries that work well under the chosen conditions. This does not invalidate the approach, but it should be made explicit and ideally addressed by incorporating productivity-aware controls.
5) Generalizability claims are promising but currently under-supported.
6) Finally, presentation could be improved by sharpening the definition of “closed loop” and the decision points that truly depend on the algorithm. It would help to provide an explicit flow diagram showing the inputs and outputs at each stage, which data are used to update the model, and what parameters were fixed versus tuned between rounds. Moreover, I suggest the authors to include discussion of related tools (https://www.nature.com/articles/s41467-025-67479-w) in this study.

Reviewer #4

(Remarks to the Author)
I co-reviewed this manuscript with one of the reviewers who provided the listed reports. This is part of the Communications Chemistry initiative to facilitate training in peer review and to provide appropriate recognition for Early Career Researchers who co-review manuscripts.

Version 1:

Reviewer comments:

Reviewer #1

(Remarks to the Author)
The revisions to the manuscript, the additions to the Supporting Information, and the responses of the authors to the reviewer's comments have satisfactorily addressed this reviewer's concerns. This reviewer recommends publication without any additional revisions.

Reviewer #2

(Remarks to the Author)
The manuscript is significantly improved through the authors’ responses to the comments.
I recommend publication.

Reviewer #3

(Remarks to the Author)
The authors have addressed my concerns.

Reviewer #4

(Remarks to the Author)
I co-reviewed this manuscript with one of the reviewers who provided the listed reports. This is part of the Communications Chemistry initiative to facilitate training in peer review and to provide appropriate recognition for Early Career Researchers who co-review manuscripts.

Open Access This Peer Review File is licensed under a Creative Commons Attribution 4.0 International License, which permits use, sharing, adaptation, distribution and reproduction in any medium or format, as long as you give appropriate credit to the original author(s) and the source, provide a link to the Creative Commons license, and indicate if changes were made.
In cases where reviewers are anonymous, credit should be given to 'Anonymous Referee' and the source.
The images or other third party material in this Peer Review File are included in the article's Creative Commons license, unless indicated otherwise in a credit line to the material. If material is not included in the article's Creative Commons license and your intended use is not permitted by statutory regulation or exceeds the permitted use, you will need to obtain permission directly from the copyright holder.
To view a copy of this license, visit https://creativecommons.org/licenses/by/4.0/

Astbury Centre for Structural Molecular Biology

University of Leeds
Leeds LS2 9JT

Telephone: 0113 343 6502
Fax: 0113 343 6401
E-mail: a.s.nelson@leeds.ac.uk
http://www.astbury.leeds.ac.uk
www.asn.leeds.ac.uk

11th March 2026

"Algorithm-Driven, Phenotype-Directed Bioactive Molecular Discovery"

Dear Dr Guo,

We thank the reviewers for their extensive comments on our manuscript. The reviewers have noted that our approach is exciting, is clearly described and documented, and enables algorithm-driven phenotype-directed discovery of bioactive molecules. Our approach has been exemplified using a specific connective reaction and a specific phenotypic effect, but we believe that it has the potential to be a general approach for the discovery of bioactive small molecules. We have provided a point-by-point response to the specific comments that were raised by the reviewers below, but also summarise here the major changes that we have made:

- New section in the Supplementary Information (Data Flow in the Closed Loop Cycle), including a detailed overview of the inputs/outputs, experimental steps, decision points and algorithmic workflow across the closed loop (new Supplementary Figure 1).
- New section in the Supplementary Information (Algorithmic Design Considersations) including:
  - Comparison of chemical space populated by 2.9M ChEMBL compounds and the enumerated reaction space products (new Supplementary Figure 2)
  - Impact of cost-filtered sampling on the diversity and price of reaction batches (new Supplementary Figure 3)
  - Impact of batch sampling methods (MaxMin vs uniform sampling) on the diversity and price of reaction batches (new Supplementary Figure 4)
- New Supplementary Figure 6 illustrating the relationship between estimated yields and purified yields of products (with hits identified)
- New Supplementary Figures 17 and 18 concerning imaging of effects of compounds on cells at lower concentration (150 nM)
- New section in the Supplementary Information with notes on controlling reproducibility across the closed loop.

- New section in the Supplementary Information that analyses the effect of alternative schemes for propagating scores after the first round of discovery (including new Supplementary Figure 20)

We have also provided a point-by-point response to the specific comments that were raised by the reviewers:

Reviewer 1

1.1 "Although it is laudable that the authors are using an uncommon C-H annulation reaction to construct their library, the enumerated virtual design space (850 x 354 coupling partners) is rather small, perhaps due to the relatively limited commercial availability of alkynes and alkenes as building blocks. By comparison, standard high-throughput phenotypic screening campaigns using pre-synthesized compound collections often number in the tens to hundreds of thousands. This relative lack of chemical diversity could ultimately limit the hit rate and utility of a phenotypic screen. A visualization of the virtual design space as well as the compounds actually synthesized in comparison to a standard collection of drug-like compounds like CheMBL would be helpful here."

Response: Our approach embeds the execution of reaction arrays into the workflow, which contrasts with high-throughput screens of curated collections of pre-synthesised compounds (which is now noted in the opening paragraph). We have now enumerated a virtual library of ~700,000 products (including regioisomers) that may potentially be accessible from all combinations of coupling partners. We have compared the diversity of this virtual library with the ~2.9M compounds in the ChEMBL database. The UMAP representation in new Supplementary Figure 2 conveys the high diversity of our virtual library. We have noted in the manuscript that: "The diversity of products that may be potentially accessible from these reagent sets was compared with that of the ChEMBL database, and was shown to be high (Supplementary Figure 2)."

1.2 This reviewer also found it questionable that building block cost was used as a selection criterion to pick representatives from each cluster for synthesis. Acknowledging the practical limitations of conducting library synthesis in an academic environment and on a limited budget, the potential bias this selection criterion could introduce into the library should be considered. For example, one could imagine that building block cost might correlate with complexity and drug-like physicochemical properties. Similar to the previous comment, a visualization of the targets selected for synthesis versus those in the total virtual design space of drug-like property space would provide some context here.

Response: The impact on using hard price cut-offs on diversity and molecular properties has previously been considered for commercially-available building blocks: https://pubs.acs.org/doi/10.1021/acscombsci.5b00063 (which we have now cited: reference 58). Instead of using hard price cut-offs, we therefore clustered substrates/co-substrates into 128 clusters, and then choose the cheapest substrate from each cluster. We have explained this more clearly in Section B, and added in new data (Supplementary Figure 3) that demonstrates that the designed

batches were more than three-fold cheaper, but had comparable diversity, to those selected from the full combination set:

"We found that batches of reactions chosen from all 850 x 254 combinations of substrates A and co-substrates B were prohibitively expensive (Supplementary Figure 3A). It has previously been shown that implementation of hard price cut-offs can adversely affect the diversity of molecular properties of commercially-available building blocks. Instead of using hard price cut-offs, we therefore algorithmically clustered the sets into 128 clusters, using kmeans and based on MHFP molecular fingerprints, from which the cheapest representatives were chosen...The 100 generated batches utilised substrates/co-substrates with total costs less than £4,000, with diversity scores between 0.80 and 0.86. (Figure 3B); these batches were much cheaper, but had comparable diversity, to those chosen from the full combination space (Supplementary Figure 3B)"

1.3 *Given the somewhat small size of the library (80 compounds, 0.4% of the 128 x 128 clustered matrix) and the rather low synthetic success rate of the Rh-catalyzed annulation reaction (35%), the productive combinations (green and yellow lines in Figure 4C) represent only a small fraction of the combination space, with significant regions of the Substrate A PC1 and PC2 and Substrate B PC1 property space being poorly sampled. While one could make the counter-argument that the screen did yield hits with a phenotype of interest (albeit via a well-known mechanism), if the point of a phenotypic screen is to uncover new biology, maximizing chemical diversity should be one of the goals. In this light, it is surprising that the authors did not attempt to optimize failed reactions from either round of the library synthesis. As it stands, it seems fair to say that the chemical space coverage is highly biased / limited by the synthetic scope of the one set of reaction conditions that were applied.*

Response: The suggestion to integrate reaction optimisation into our workflow is interesting but would be a major undertaking. An alternative might be to explore a few alternative reaction conditions (rather than just a single reaction condition), which could well increase the proportion of successful reactions (as has been shown for high-through discovery driven by aminations of aryl halides; cited ref. 36). We have amended the discussion to note these possible future directions for autonomous phenotype-directed molecular discovery:

"Nonetheless, the proportion of productive reactions in round one, under one set of reaction conditions, was quite low (35%). It has been shown, in a discovery programme driven by aminations of aryl bromides, that the use of alternative reaction conditions can significantly increase reaction productivity and, hence the chemical space that may be explored. The incorporation of alternative reaction conditions, or even reaction optimisation, may be a fruitful future direction for expanding accessible chemical space for phenotype-directed molecular discovery."

See also response to comment 2.1 on maximizing chemical diversity.

1.4 *An analysis of the cell-painting assay morphological fingerprints of the five hits from the round 1 screen showed that four mapped with high similarity to the tubulin cluster, while the fifth seemed to elicit a distinct phenotype. In light of the statement that "hits that have <70% similarity are likely*

to have mechanisms that are distinct from all of the defined functional clusters, and may be of particular interest", it is puzzling that the authors chose in round 2 of the library to bias designs toward the tubulin cluster, which is well-known biology. This choice seems to undercut the primary premise of phenotypic screening, since if the goal were to screen for tubulin modulators that would be a much easier task in a target-directed screening approach. A fuller explanation of this decision in the manuscript is warranted..... Along the lines of the previous comment, no mention is made in the manuscript of any follow-up work done on the functionally active hits from Round 1 and 2 that did not map to the tubulin cluster. This is indeed puzzling given the statement in the introduction that one advantage of phenotypic screening is to avoid being "biased towards previously-identified protein targets" which in this case appears to be tubulin.

Response: As the reviewer suggests, it would indeed have been interesting to discover bioactive compounds that were not associated with a functional cluster. Our approach requires the formulation of a basis of scoring hits. We had only considered the possibility of scoring compounds on the basis of similarity to a pre-defined reference phenotype (here, the tubulin functional cluster). However, it could also be possible to score compounds on the basis of *dissimilarity* to functional clusters (or even all reference compounds). We have moved the comment about hits with <70% similarity to all clusters into an expanded section in the discussion section:

"Our approach requires a basis for scoring hits, and we scored on the basis of similarity to a reference phenotype (the tubulin functional cluster). We did, however, also discover some hits that were dissimilar to all defined functional clusters and which may be of particular interest: the application of our approach to discover such compounds explicitly would require an appropriate scoring scheme, for example that rewarded dissimilarity to all defined functional clusters or reference compounds."

Reviewer 2

2.1 *Whilst the work outlined in this manuscript enables a level of chemical space scoping (as beautifully illustrated in figure 3) hard to reach chemical space still remains unexplored. It would be good for the authors to iterate this in their conclusion, and emphasise the volume of synthetic discovery research that still remains paramount.*

Response: We agree that there is a need for more operationally-simple methods that enable diverse chemotypes to be prepared and evaluated. We have previously exploited reactions with multiple possible outcomes (e.g. metal carbenoid chemistry; cited references 35 and 60) to drive bioactive molecular discovery. Although such reactions can be low-yielding, we strongly advocate screening products before structural elucidation in order to focus efforts on functional molecules. We have expanded the discussion as follows:

"Moreover, the diversity of readily-explorable chemical space could also be expanded through development of a broader range of operationally-simple connective reactions that enable diverse chemotypes to be prepared."

2.2 [no major comments]…though the actual diversity of chemical space is not so wide, and the authors must mention this in the conclusions. It is an exciting starting point for this concept, but there is much work required by a community to enable fully diverse chemical space to be accessed.

Response: See response to comments 1.1 and 2.1 above. We have now captured the chemical diversity of the virtual reaction space in a new Supplementary Figure 2. The discussion also explicitly recognizes the need to explore diverse chemical space, and provides suggestions for how this might be addressed in future activities.

2.3 The presentation of the statistical rigor could be improved, (for example see points A, B, and C), however we believe that the authors probably have these data and that these points should be readily addressed.

2.3A The yield threshold (15%, on page 6) is simply stated, and as such seems arbitrary. We are sure that the authors will have thought carefully as to why to set this at 15%. They should justify this in the text (why not 5%, why not 20%, or 40%). The setting of the threshold will promote bias toward certain chemotypes. If the threshold is set too high, low-yielding but potent products are excluded (again diminishing access to diverse and relevant chemical space). It would be good to also discuss the bias that selection, and threshold setting can introduce. The rational behind the setting of the following thresholds should also be discussed, with appropriate detail, and its impact on potential outcomes/bias briefly outlined:

Thresholds for "hit" definition. Page 8

Induction >5% and >80% tubulin cluster similarity: score 1.

Induction >5% and 70-80% tubulin cluster similarity: score between 0 and 1 (in 0.1 steps).

Thresholds 80% 70-80% . Sensitivity to parameter choice should be discussed.

"Hits that have <70% similarity are likely to have mechanisms that are distinct from all of the defined functional clusters": please justify this statement in the manuscript.

Response:

- We used analytical HPLC with ELSD detection (~3 minute run) in order to prioritise samples for mass-directed purification (~20 minute run). The chosen threshold (15%) was informed by our practical requirements including the need for sufficient material to enable gravimetric quanitification (ideally ≥0.5 mg). We have now explicitly justified this choice of threshold in the manuscript, and included a new Supplementary Figure 6 to show the relationship between estimated yields and purified masses. Two active compounds (at 10 μM) from round one that were >70% similar to the tubulin functional cluster had were obtained from reactions with low estimated yields; a higher choice of threshold could have excluded one or both of these compounds, which would have had an effect on scoring of the round one hits.

  "We used 15% estimated yield as a threshold for mass-directed purification based on our previous experience of obtaining sufficient material (ideally ≥0.5 mg) for gravimetric quantification; a higher threshold may have excluded identified hits and affected batch design in the second round (see Supplementary Figure 6). The dried isolated products were weighed using an automated balance."

- The criteria for scoring hits were informed by previously established practices with the cell painting assay in which an induction (i.e. proportion of significantly-changed parameters) of >5% is widely used to identify compounds with significant phenotypic effects (cited references 15, 50, 51, 60, 63 and 64). We deliberately avoided a hard cut-off for similarity to the tubulin functional cluster; here, the criteria were chosen based on the establishment of morphological subprofile analysis for bioactivity annotation (cited ref 63), and its exploitation in identifying tubulin-targeting compounds (cited ref 64). This aspect is now specifically commented on in Section C:

  "These criteria were informed by previously-established practices with the cell painting assay in which induction ≥5% has been used to identify compounds with significant morphological effects; in addition, a hard cut-off for cluster similarity was deliberately avoided, with the criteria informed by the establishment of morphological subprofile analysis, and its exploitation in identifying tubulin-targeting compounds."

  We have also included a new Figure (Supplementary Figure 20) that analyses the effect of alternative schemes for propagating scores after the first round of discovery. In particular, we compared our scoring approach (induction >5% and >80% tubulin cluster similarity: score 1; induction >5% and 70-80% similarity: ramped score) with two alternatives: (1) induction >5% and >90% tubulin cluster similarity: score 1; induction >5% and 80-90% similarity: ramped score; (2) induction >5% and 90-100% similarity: ramped score. Here we present the propagated scores for all combinations (with the hits that identified in round 2 highlighted). We have noted in the legend that: "Thresholds of 70% and 80% produce similarly smooth score distributions, with more graded score separation at 70%. In contrast, the 90% threshold yields a more compressed distribution with uniformly lower scores. Notably, the experimentally-confirmed hits fall within the graded continuum at the 70% threshold, where they receive a spread of partial scores, as opposed to the clustering observed with a 80% threshold. With the 90% threshold, these same hits split into two distinct groups – one high-scoring and one low-scoring – such that only the closest structural analogues would have been prioritised at this more stringent cutoff."
- Also see response to comment 1.4 in relation to hits that have <70% similarity.

2.3B Small error: Filters at Figure 3A and Supplementary Table 11 do not match, moreover the table filter is not full.

Property	Filter 1<br>Figure 3A (group A)	Filter 1<br>Figure 3A (group B)	Filter 2<br>Supplementary Table 11<br>Filter Condition | Include if
Heavy atoms	6≤HA≤**13**	6≤HA≤**14**	<6 | TRUE<br>>14 | TRUE
XLogP	**-0.5**≤XLogP≤3.5;	**-1.5**≤XLogP≤3.5;	< −1.5 | TRUE<br>>3.5 | TRUE
Rotatable bonds (non-terminal)	≤2	≤2	>2 | TRUE
Aromatic atoms	5≤AA≤12	Not constrained	Not constrained

These filters are logically opposite.

Again the selection of filters for group A and B, in figure 3A (and throughout the experimental design) should be discussed.

Response: We thank the reviewer for pointing out this error. We have now corrected the details of the filter conditions in Supplementary Tables 10 and 11.

2.3C Algorithm development. Was the approach compared with a more simplistic algorithm? Is it possible that your algorithm is more complex than it need be? It would be really good to exemplify how your approach out-performs the standard simple models, where random, yet chemical sensible selection is implemented. Showing this as a baseline would be really great. You probably have this work already, and just need to embed it into the manuscript.

You have shown that the propagated scores correlate with round 2 hits and a statistical significance (t-test). However, this is a test of internal consistency. It does not compare the algorithm against alternative strategies.

*Algorithm-guided round 2 was stated to have generated more hits, and higher productivity. Please include comparative data that you have of the performance of your algorithm vs the obvious alternatives that are commonly used. For example, the scoring/propagation scheme is not compared against: random sampling with yield bias, * or simple nearest-neighbour selection, ** or "Greedy similarity search".*

Response: We developed an algorithm that enabled the design of diverse batches that could be executed within the constraints of our budget and our robotic infrastructure (in particular, number of unique reagents and reactions that could be accommodated). The alternative suggestions from the reviewer are helpful suggestions, but would still need to be implemented within these constraints. In addition, the suggestion to select nearest neighbours would require the exploitation of reaction(s) with predictable products. We note that we did use an expanded virtual reaction space in round two in which additional reagents were included that were similar to those used in the reactions that had yielded hits.

We have now compared the diversity and price of round one reaction batches designed using the approach described in the paper (MinMax) with uniform sampling (in which all combinations have the same probability of being selected). These data, presented in the new Supplementary Figure 4, show that our approach yields significantly more diverse batches which are comparable in price.

2.4A narrow chemical space is accessed and explored, despite the original positioning within the abstract .

Jaccard similarity on concatenated reagent fingerprints.

Using similarity of (A,B) pairs rather than predicted products: assumes a single reaction outcome. In the authors approach, similarity is set to track with the reagent similarity. The authors' hypothesis/assumption here isn't tested, validated or discussed. It is really important to reflect on this aspect.

Similarity(Reaction 1, Reaction 2) = Jaccard(Fingerprint(A1 + B1), Fingerprint(A2 + B2))

The use of Jaccard similarity on concatenated reagent fingerprints implicitly assumes that similarity at the reagent-pair level correlates with similarity of reaction products and thus similarity of biological phenotypes. This assumption is not discussed or validated and may not hold for reactions with multiple possible outcomes.

But same reagents ≠ same products, (unless there is only one way in which they can combine with a single regiochemistry/stereochemistry etc) Minor substituent changes can of course redirect C–H activation.

Response: See responses to comment 1.1 concerning chemical space. We chose to use the fingerprints of the reactants to compare reactions because our choice of chemistry meant that it was not possible to predict a single product for each combination.

It was found that using concatenated MHFP6 fingerprints for reagent pairs offered the best balance between computational feasibility and retaining high-quality fingerprint information. With MHFP6, application to the enumerated reaction products (if predictable), which are more structurally complex than the reagents, would lead to an explosion in shingle count, pushing fingerprint generation and Jaccard similarity calculations to exceed ~12 hours of GPU time per iteration. The alternative would be to fall back to fast bit-based fingerprints (e.g., Morgan), but these lose the substructure-level resolution that motivated the use of MHFP6 in the first place. We acknowledge that using Jaccard similarity on concatenated reagent fingerprints implicitly assumes that similarity at the (A,B) reagent-pair level correlates with similarity of the resulting products and, by extension, their biological phenotypes. However, within the computational constraints and design intentions outlined above, concatenated MHFP6 fingerprints for reagent pairs provided the highest resolution, tractable representation of reaction similarity and was therefore the most informative option available for guiding the sampling strategy in the closed-loop system.

We have clarified in the discussion as follows: "Here we used the concatenated fingerprints of the reagent pairs, as applying MHFP6 to fully enumerated products would cause a dramatic expansion in molecular shingles, significantly increasing computational time per iteration (estimated at >12 h GPU in this case). We also chose not to use fast bit-based product fingerprints (e.g., Morgan) which lose the substructure-level resolution that motivated the use of MHFP6, opting therefore for maximising information while maintaining tractability. "

2.5(from the abstract) "phenotype-driven discovery can yield bioactive molecules with unexpected mechanisms of action, and open paths to first-in-class drugs"

a. Having this point in the abstract makes the reader anticipate that a new "first in class" drug will be identified: The tubulin mechanism is well known, strongly represented in Cell Painting reference clusters, rapidly inferred by similarity metrics. No truly unexpected MoA is shown.

b. If the sentiment were correct, the sentence should still be written as "reveal" not yield.

Response: This sentence in the abstract does not refer to our approach specifically, but rather to the potential for phenotype-driven approaches generally to reveal bioactive molecules with unexpected mechanism. Later in the abstract, we state clearly that our approach "enabled the discovery and structural evolution of a series of tubulin modulators". We are happy to stand by this wording. See response to comment 3.5 on the potential of our approach to be generalizable for function-driven exploration of chemical space.

2.6 (from summary) "may reveal new mechanistic insights" Again, in this section, it should be explained that without appropriate threshold setting, the likelihood of finding a new mechanism of action is low.

Response: We thank the reviewer for this comment, and have amended the wording as follows:

"We envisage that our approach, driven by other phenotypic assays with appropriate scoring criteria, may enable the discovery of new classes of chemical probes of biological systems that have the potential to reveal new mechanistic insights and, ultimately, open new opportunities for drug discovery."

2.7Due to the very mixed readership that this manuscript will rightly attract, it would be helpful to be very clear on definitions. Often particular chemical scaffolds are referred to as targets. In the introductory sentences, elaborate: target centric-approaches (that focus on small molecule binding to a particular biomolecular target)

Response: We have used the word "target" throughout to mean specific proteins **not** designed molecules. We have clarified what "target" means when the word is first used:

"…target-centric approaches (i.e. those that focus on specific proteins)."

2.8Red and green are used frequently in the figures, avoiding colour combinations associated with colour blindness will be helpful to some of the readership.

Response: We did choose palettes carefully, based on feedback from one of the authors who has colour blindness. This is particularly important where the use of colour enables readers to understand data categories. For example, we used grey, light blue, yellow, green and purple to distinguish outcomes in each round of discovery. Where red and green have been used in the same figure, it is not necessary to distinguish these colours in order to understand the scientific content.

2.9 Potency claims are modest. No IC_{50}, EC_{50}, or selectivity data, no comparison to known tubulin binders beyond nocodazole. "Improved potency" claim is qualitative.

Response: We have now included additional data on the effects of compounds at lower concentration (150 nM) (Supplementary Figures 17 and 18). These data are reported for the compounds that had a statistically-significant effect on stained area at 750 nM. We have now noted in the manuscript that: "Notably, two hits from round two (**R2-1** and **R2-14**), unlike **R1-2** from round one, still had a statistically-significant effect on stained area at lower concentration (150 nM) (Supplementary Figures 17 and 18)." These new data substantiate the following revised sentence in the discussion: "In addition, by assaying at a lower concentration (150 nM), we showed that two hits from round 2 were significantly more potent than those obtained in from round 1."

Reviewer 3

3.1A central concern is that the "phenotype-directed, structure-blind" framing is not fully realized in the current implementation. While the workflow avoids early structural elucidation of all products, the algorithmic propagation is performed on reagent-pair fingerprints rather than product fingerprints, and the readout heavily leverages similarity to a predefined tubulin morphological cluster. This makes the optimization loop partially dependent on prior knowledge embedded in the reference clusters and raises the question of whether the system is discovering novel phenotypes or mostly enriching toward a known mechanism-of-action signature.

Response: We have addressed these points in our response to comment 1.4. Specifically, we have explained why we have applied our approach to the discovery of bioactive molecules that induce a specific defined phenotypic effect. We have also commented on the exciting possibility of discovering compounds which induce effects that are dissimilar to sets of reference phenotypes.

3.2Another major issue is the limited mechanistic depth around the discovered series. The tubulin conclusions are supported by follow-up imaging, mitotic arrest, and a polymerization assay for a subset of compounds, which is helpful. However, the screening concentrations in cell painting (10–50 µM) are high, and only a fraction of compounds show effects at sub-micromolar concentrations in the follow-up assay. More quantitative pharmacology may help strengthen the work.

Response: We have addressed this point in our response to comment 2.9. Specifically, we have now included imaging data for lower concentrations of specific compounds.

3.3From a methods and statistics standpoint, the manuscript would benefit from tighter clarity on replication and reproducibility across the closed-loop. Many steps involve multiple potential sources of variance, reaction yield estimation via ELSD, automated purification and weighing, compound plating, and phenotypic profiling.

Response: We have now added a dedicated subsection ("Notes on Controlling Reproducibility") detailing the key controls implemented at each step of the closed-loop workflow. This includes: routine ELSD calibration and verification; replication of the Round 1 reaction array; quantified HPLC and evaporation losses; and triplicated weighing of all isolated products. These additions clarify how variance was monitored and constrained across the closed loop (Supplementary Information pages 34-35).

3.4 A key technical concern is whether the closed-loop is learning biology or learning chemistry productivity. The authors acknowledge that scores correlate with reaction productivity, which is practically useful, but it also risks conflating "high-yielding chemistry regions" with "bioactive chemistry regions." The platform may preferentially select combinations that produce isolable products and thus bias discovery toward chemistries that work well under the chosen conditions. This does not invalidate the approach, but it should be made explicit and ideally addressed by incorporating productivity-aware controls.

Response: Our overall aim was to establish a general, algorithm-driven approach for the discovery of synthetically-accessible bioactive molecules with specific phenotypic effects. After round one, compounds could only be identified as hits if they could BOTH be made AND if they induced the targeted phenotype. The new Supplementary Figure 6 also shows that some of these hits were obtained from lower-yielding reactions. In round two, we demonstrated that there was an increase in the proportion of successful reactions AND an increase in the number of bioactive products. We have adjusted the summary to make clear that our approach can enable discovery of synthetically-accessible bioactive molecules that induce a specific phenotypic effect.

3.5 Generalizability claims are promising but currently under-supported.

Response: We think that it is important to point out the potential for our approach to be applied with different chemistries and/or assays (phenotypic or otherwise). Nonetheless, we recognize that generality has not yet been demonstrated, and we have tempered the wording throughout (removed words struck through; new wording in italics): "…the approach ~~would~~ *may* be generalizable…"; "Our approach *has the potential to be* general: it could *be adapted to* exploit…"; "Overall, we have established a ~~general~~ framework….".

3.6 Finally, presentation could be improved by sharpening the definition of "closed loop" and the decision points that truly depend on the algorithm. It would help to provide an explicit flow diagram showing the inputs and outputs at each stage, which data are used to update the model, and what parameters were fixed versus tuned between rounds. Moreover, I suggest the authors to include discussion of related tools (https://www.nature.com/articles/s41467-025-67479-w) in this study.

Response: We thank the reviewer for highlighting this paper that has been published since we submitted our manuscript. The paper describes an interpretable framework for phenotypic profiling, rather than a tool for phenotype-driven molecular discovery. We have cited the paper (reference 52) in the following context: "We exemplified our discovery approach using the cell painting assay which enables morphological effects of compounds to be profiled; like other assays, high content images may be transformed into low-dimensional representations (morphological fingerprints)."

We have now also included a detailed flow diagram (Supplementary Figure 1) that shows the inputs/outputs; highlights fixed and tunable parameters; and marks the model update points in the loop.

Reviewer 4

This reviewer was a co-reviewer who submitted a joint report with another reviewer.

Thank you for considering our manuscript. We look forward to your response.

Yours sincerely,

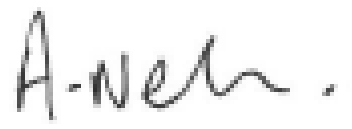

Professor Adam Nelson

Professor of Chemical Biology, School of Chemistry and Astbury Centre for Structural Molecular Biology, University of Leeds, Leeds, UK

and

Head of High-Throughput Molecular Discovery, The Rosalind Franklin Institute, Harwell Campus, Didcot, Oxfordshire, UK

Manuscript #	COMMSCHEM-25-1086-T
**Title**	**Algorithm-Driven, Phenotype-Directed Bioactive Molecular Discovery**

"I co-reviewed this manuscript with one of the reviewers who provided the listed reports. This is part of the Communications Chemistry initiative to facilitate training in peer review and to provide appropriate recognition for Early Career Researchers who co-review manuscripts."

The ECR will be asked to complete optional demographic information, and to submit the form from their account to ensure appropriate recognition of the co-review activity (e.g. by ORCID).

Overall Assessment

We were deeply impressed by the content of the manuscript and found it a genuine pleasure to read. The work is conceptually exciting, thorough, well-structured, and both scientifically interesting and sound. The manuscript outlines an elegant experimental design where the integration of automated synthesis, phenotypic screening and iterative algorithmic re-design are beautifully exemplified, in a closed-loop manner. From the experimental section it is clear that the experimental execution of this beautiful proof-of-concept is excellent.

The authors comment that as molecular diversity is dominated by a small number of robust synthetic reactions, and that this has contributed to uneven historic exploration of chemical space. We strongly agree with this assessment. Whilst the work outlined in this manuscript enables a level of chemical space scoping (as beautifully illustrated in figure 3) hard to reach chemical space still remains unexplored. It would be good for the authors to iterate this in their conclusion, and emphasise the volume of synthetic discovery research that still remains paramount.

Major Comments

None, though the actual diversity of chemical space is not so wide, and the authors must mention this in the conclusions. It is an exciting starting point for this concept, but there is much work required by a community to enable fully diverse chemical space to be accessed.

Minor Comments

1. The presentation of the statistical rigor could be improved, (for example see points A, B, and C), however we believe that the authors probably have these data and that these points should be readily addressed.

A. The yield threshold (15%, on page 6) is simply stated, and as such seems arbitrary. We are sure that the authors will have thought carefully as to why to set this at 15%. They should justify this in the text (why not 5%, why not 20%, or 40%). The setting of the threshold will promote bias toward certain chemotypes. If the threshold is set too high, low-yielding but potent products are excluded (again diminishing access to diverse and relevant chemical space). It would be good to also discuss the bias that selection, and threshold setting can introduce.

The rational behind the setting of the following thresholds should also be discussed, with appropriate detail, and its impact on potential outcomes/bias briefly outlined:

Thresholds for “hit” definition. Page 8
Induction >5% and >80% tubulin cluster similarity: score 1.
Induction >5% and 70-80% tubulin cluster similarity: score between 0 and 1 (in 0.1 steps).
Thresholds 80% 70-80% . Sensitivity to parameter choice should be discussed.
"Hits that have <70% similarity are likely to have mechanisms that are distinct from all of the defined functional clusters": please justify this statement in the manuscript.

B. Small error: Filters at Figure 3A and Supplementary Table 11 do not match, moreover the table filter is not full.

Property	Filter 1<br>Figure 3A (group A)	Filter 1<br>Figure 3A (group B)	Filter 2<br>Supplementary Table 11<br>Filter Condition | Include if
Heavy atoms	6≤HA≤**13**	6≤HA≤**14**	**<**6 | TRUE<br>**>**14 | TRUE
XLogP	**-0.5**≤XLogP≤3.5;	**-1.5**≤XLogP≤3.5;	**<** −1.5 | TRUE<br>**>**3.5 | TRUE
Rotatable bonds (non-terminal)	≤2	≤2	**>**2 | TRUE
Aromatic atoms	5≤AA≤12	Not constrained	Not constrained

These filters are **logically opposite**.

Again the selection of filters for group A and B, in figure 3A (and throughout the experimental design) should be discussed.

C. Algorithm development. Was the approach compared with a more simplistic algorithm? Is it possible that your algorithm is more complex than it need be? It would be really good to exemplify how your approach out-performs the standard simple models, where random, yet chemical sensible selection is implemented. Showing this as a baseline would be really great. You probably have this work already, and just need to embed it into the manuscript.

You have shown that the propagated scores correlate with round 2 hits and a statistical significance (t-test). However, this is a test of internal consistency. It does not compare the algorithm against alternative strategies.

Algorithm-guided round 2 was stated to have generated more hits, and higher productivity. Please include comparative data that you have of the performance of your algorithm vs the obvious alternatives that are commonly used. For example, the scoring/propagation scheme is not compared against: random sampling with yield bias,[1]* or simple nearest-neighbour selection,[2]** or “Greedy similarity search”.

---

[1] *Random sampling (with yield bias) = Randomly select new substrate–co-substrate combinations; but restrict to combinations likely to react (e.g. similar chemistry, productive reagents)

[2] **Simple nearest-neighbour selection.

Take round 1 hits, pick the most similar reagents (fingerprint similarity), combine them directly, without scoring, propagation, or Max/Min. This is a very common heuristic in medicinal chemistry.

2. A narrow chemical space is accessed and explored, despite the original positioning within the abstract .

Jaccard similarity on concatenated reagent fingerprints.

Using similarity of (A,B) pairs rather than predicted products: assumes a single reaction outcome. In the authors approach, similarity is set to track with the reagent similarity. The authors' hypothesis/assumption here isn't tested, validated or discussed. It is really important to reflect on this aspect.

Similarity(Reaction 1, Reaction 2) = Jaccard(Fingerprint(A1 + B1), Fingerprint(A2 + B2))

The use of Jaccard similarity on concatenated reagent fingerprints implicitly assumes that similarity at the reagent-pair level correlates with similarity of reaction products and thus similarity of biological phenotypes. This assumption is not discussed or validated and may not hold for reactions with multiple possible outcomes.

But same reagents ≠ same products, (unless there is only one way in which they can combine with a single regiochemistry/stereochemistry etc) Minor substituent changes can of course redirect C–H activation.

Other very minor points, that would help with expectation setting and clarity:

1. **(from the abstract)**

"phenotype-driven discovery can yield bioactive molecules with unexpected mechanisms of action, and open paths to first-in-class drugs"

a. Having this point in the abstract makes the reader anticipate that a new "first in class" drug will be identified: The tubulin mechanism is well known, strongly represented in Cell Painting reference clusters, rapidly inferred by similarity metrics

   No truly *unexpected* MoA is shown.

b. If the sentiment were correct, the sentence should still be written as "reveal" not yield.

(from summary)

"may reveal new mechanistic insights"

Again, in this section, it should be explained that without appropriate threshold setting, the likelihood of finding a new mechanism of action is low.

2. Due to the very mixed readership that this manuscript will rightly attract, it would be helpful to be very clear on definitions. Often particular chemical scaffolds are referred to as targets. In the introductory sentences, elaborate: target centric-approaches (that focus on small molecule binding to a particular biomolecular target)

3. Red and green are used frequently in the figures, avoiding colour combinations associated with colour blindness will be helpful to some of the readership.

4. Potency claims are modest. No IC□□, EC□□, or selectivity data, no comparison to known tubulin binders beyond nocodazole. “Improved potency” claim is qualitative.

These are all minor points that we are sure that the authors will have considered and can readily address. Once these modifications are incorporated this will be a superb and timely contribution to the literature.
