# Supplementary material for: Algorithm-driven, phenotype-directed bioactive molecular discovery
Source: Commun Chem. 2026 May 16;9:267. doi: 10.1038/s42004-026-02066-8 (PMC13439441; doi:10.1038/s42004-026-02066-8)

Supplementary Information

Algorithm-Driven, Phenotype-Directed Bioactive Molecular Discovery

Amalia-Sofia Piticari, Samuel D Griggs, Laura Crawford, Joss Whittle, Brian S Mantilla, Sonja Sievers, Megan H Wright, Stephen P Marsden, Mark Basham and Adam Nelson

Table of Contents

General Considerations..1

A. Data Flow in the Closed Loop Cycle..2

B. Algorithmic Design Considerations..3

1. Comparison of Defined Virtual Reaction Space to Drug-Like Space..............3
2. Impact of Cost Filtered Sampling on Chemical Diversity..............4
3. Selecting the Batch Sampling Method (Maxmin vs Uniform)..............5

C. Experimental Workflow Procedures..7

1. Automated Reaction Set-up..............7
2. Quantification Protocol..............12
3. Purification Method Parameters..............14
4. Weighing, Drying and Plating of Isolated Compounds..............16
5. Methods for Cell Painting Assay..............17
6. KNIME Workflow for Filtering Substrates/Co-substrates..............20
7. Methods for Cell Biology..............28
8. Notes on Controlling Reproducilibility..............34

D. First Discovery Round..36

1. Summary and Outcomes..............36
2. Scoring Strategies..............39

E. Subsequent Discovery Round – Summary and Outcomes..40

F. Synthetic Procedures..44

References..57

NMR Spectra..58

General Considerations

Commercially available starting materials were obtained from SigmaAldrich (Merck), Fluorochem or Fisher. Solvents was purchased from SigmaAldrich (Merck) and was used as supplied.

All array reactions were set up using a Zinsser Analytic Lissy platform, equipped with the Zinsser WinLissy software version 8. The reactions were set up in Zinsser 96-well blocks equipped with 1 mL glass vials.

Plate reformatting and aliquoting for analytical and purification purposes were performed using a Hamilton Microlab STAR automated liquid handling system, operated via the Venus control software. The reaction mixtures were transferred into Greiner clear polypropylene 96-deep well plates (1.1 mL/well) and corresponding aliquots into Greiner clear polypropylene 96-well microtiter plates (392 µL/well).

Analytical LC-MS was performed using a system comprising a Waters Acquity H-CLASS UPLC with a PDA detector, evaporative light-scattering (ELS) detector, and SQD2 with electrospray ionisation. The system ran in positive mode using a Waters Acquity UPLC BEH C18 (50 mm × 2.1 mm × 1.7 µm) column and gradient elution with a binary solvent system comprising of MeCN + 0.1% formic acid and H_2O + 0.1% formic acid. All solvents used in analytical studies were of LC-MS grade.

Mass directed purification was performed using a Waters HPLC system comprising of a PDA detector, ELS detector and SQD2 with electrospray ionisation. The system ran in positive mode using a Waters XBridge Prep C18 (100 mm × 19 mm × 5 µm) column and gradient elution with a binary solvent system comprising of MeCN + 0.1% formic acid and H_2O + 0.1% formic acid. All solvents used in purification were of HPLC grade.

All scale-up reactions were carried out under an inert atmosphere (argon) using standard techniques, unless otherwise specified. Analytical thin-layer chromatography (TLC) was performed on precoated, aluminium-backed silica gel plates. Visualisation of the developed chromatogram was performed by UV absorbance (254 nm) and/or stained with a permanganate solution in ethanol. Chromatographic purification was performed either manually using 230–400 mesh silica according to standard techniques or using an automated flash chromatography Teledyne Isco® using the supplier provided cartridges.

Nuclear magnetic resonance spectra were recorded on either a 400 MHz Bruker or a 600 MHz Bruker spectrometer, with the frequency noted in each spectral assignment. Data was collected at 298 K unless otherwise stated. Chemical shifts for ^{1}H NMR spectra were recorded in parts per million from tetramethylsilane with the residual protonated solvent resonance as the internal standard ($CDCl_3$: δ 7.26 ppm, CD_3OD: δ 3.31 ppm). Coupling constants (J) are reported in Hertz (Hz) and splitting patterns are reported in an abbreviated manner: s (singlet), d (doublet), t (triplet), q (quartet), m (multiplet), br (broad). ^{13}C NMR spectra were recorded with complete proton decoupling. Chemical shifts were reported in parts per million from tetramethylsilane with the solvent resonance as the internal standard ($^{13}CDCl_3$: δ 77.0 ppm, $^{13}CD_3OD$: δ 49.0 ppm). Assignments of ^{1}H and ^{13}C spectra, were based upon the analysis of δ and J values, as well as DEPT, COSY, HSQC, HMBC and NOESY experiments where appropriate. ^{19}F NMR spectra are indirectly referenced to $CFCl_3$, automatically via direct measurement of the absolute frequency of the deuterium lock signal by the spectrometer hardware.

A Bruker Daltonics micrOTOF spectrometer with electrospray (ES) ionisation source was used for high-resolution mass spectrometry (HRMS)

A. Data Flow in the Closed Loop Cycle

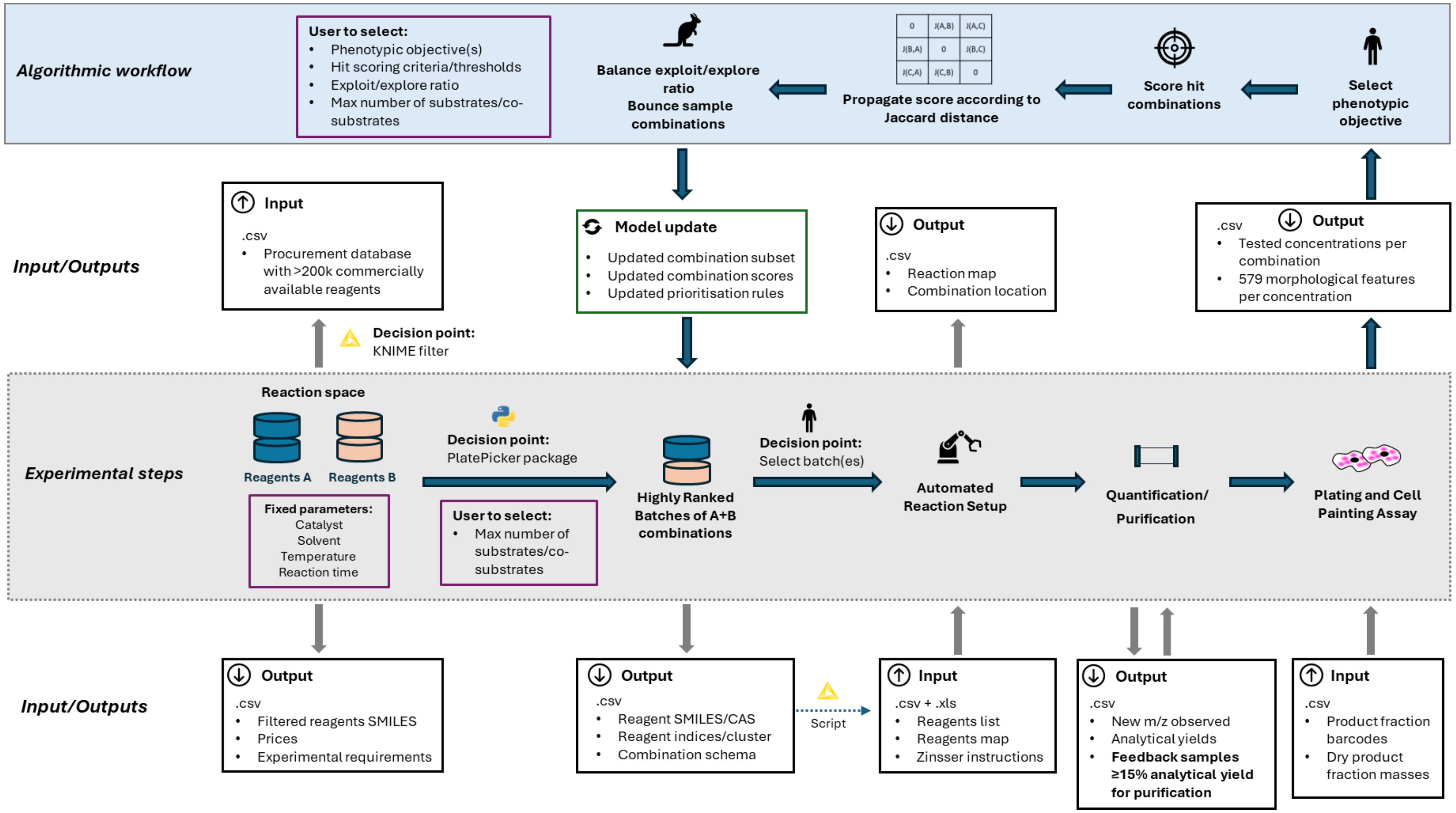

Supplementary Figure 1 Overview of the closed-loop discovery platform for algorithm-driven, phenotype-directed molecular discovery. The cycle operates with fixed experimental parameters, whilst algorithmic parameters are updated each cycle based on experimental outputs and user-defined objectives.

B. Algorithmic Design Considerations

1. Comparison of Defined Virtual Reaction Space to Drug-Like Space

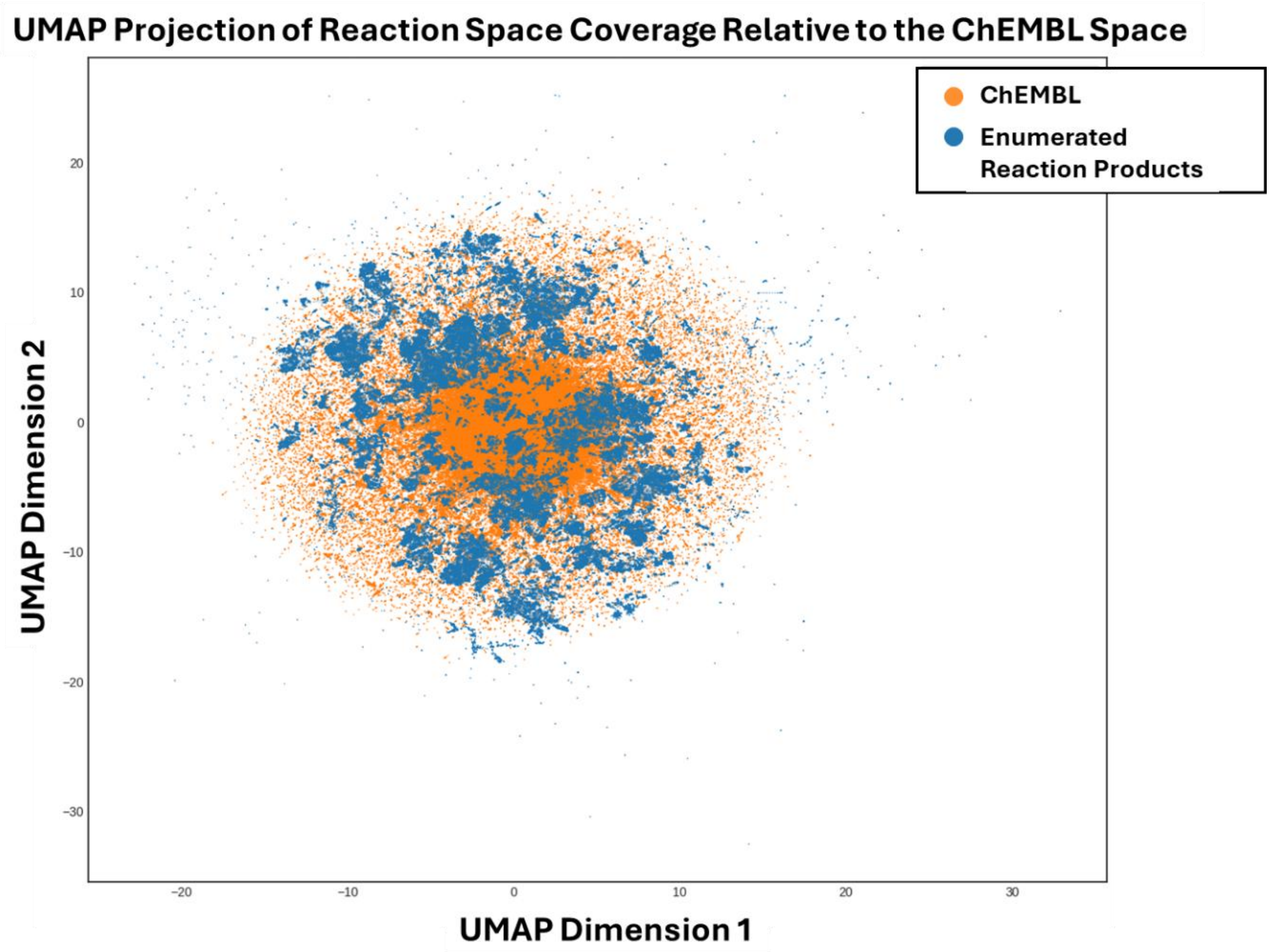

Supplementary Figure 2 Chemical space comparison between the enumerated reaction space products and a reference collection (2.9 million ChEMBL compounds).

For carboxylic acids, A* placeholders were inserted at the available *ortho* position with respect to the COOH group, and the COOH converted to CONH–A* to represent the reactive annulation species. For alkene and alkyne partners, two A* placeholders were introduced via an addition at the double bond (alkynes were converted to the corresponding alkenes without further reduction). Placeholders from each reaction partner were then connected to generate the annulated products.

Enumeration covered all substrate and co-substrate combinations (850 × 354) from our defined reaction space following commercial database filtering (see Figure 2A), including both head-to-head and head-to-tail outcomes, and all implicit stereochemical possibilities by using SMILES without specified enantiomeric or diastereomeric forms. Molecules were encoded as 2048-bit Morgan fingerprints (radius = 2) and embedded with UMAP (n_neighbors = 15, n_components = 2, metric = Jaccard). The resulting projection overlays reaction space embeddings (orange) with ChEMBL molecules (blue), illustrating a broad coverage and substantial overlap with drug-like chemical space.

2. Impact of Cost-Filtered Sampling on Chemical Diversity

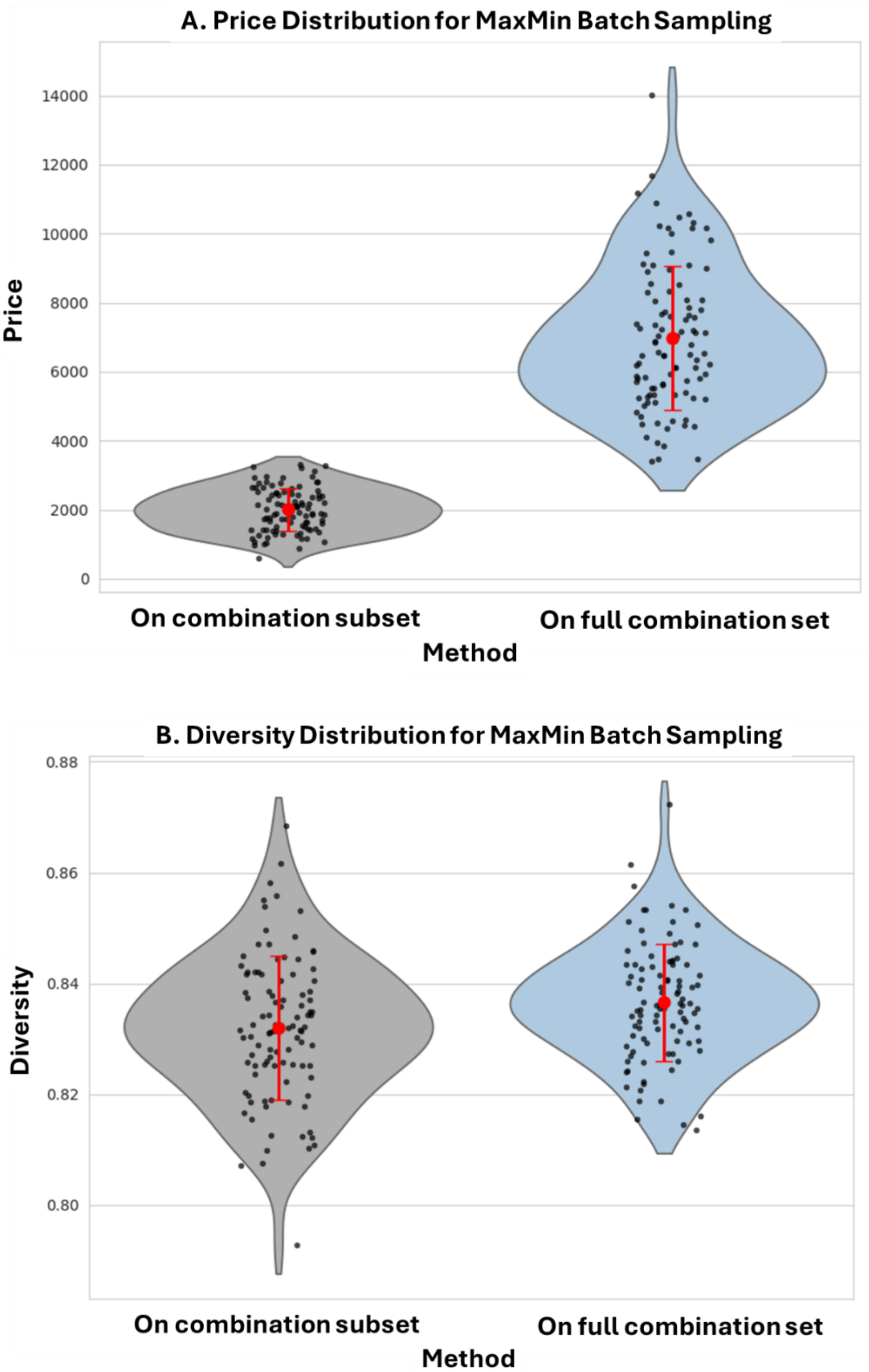

Supplementary Figure 3 Effect of sampling from a cost-filtered combination subset (128 x 128) versus the full combination space (850 x 354) on (a) total array price; (b) array diversity.

Each datapoint represents a single batch of 80 reactions produced by MaxMin sampling. Sampling from the subset significantly reduces cost while maintaining diversity comparable to selection from the full set. Panel A: The total reagent price distribution for batches drawn from the cost-filtered subset (grey) or the full combination set (blue). Two-sided t-test, p-value = 7.65×10^{-45} Panel B: Diversity distributions for the same batches, where diversity was computed as the mean pairwise Jaccard distance between MHFP6 fingerprints within each batch. Two-sided t-test, p-value = 6.38×10^{-3}

3. Selecting the Batch Sampling Method (MaxMin vs Uniform)

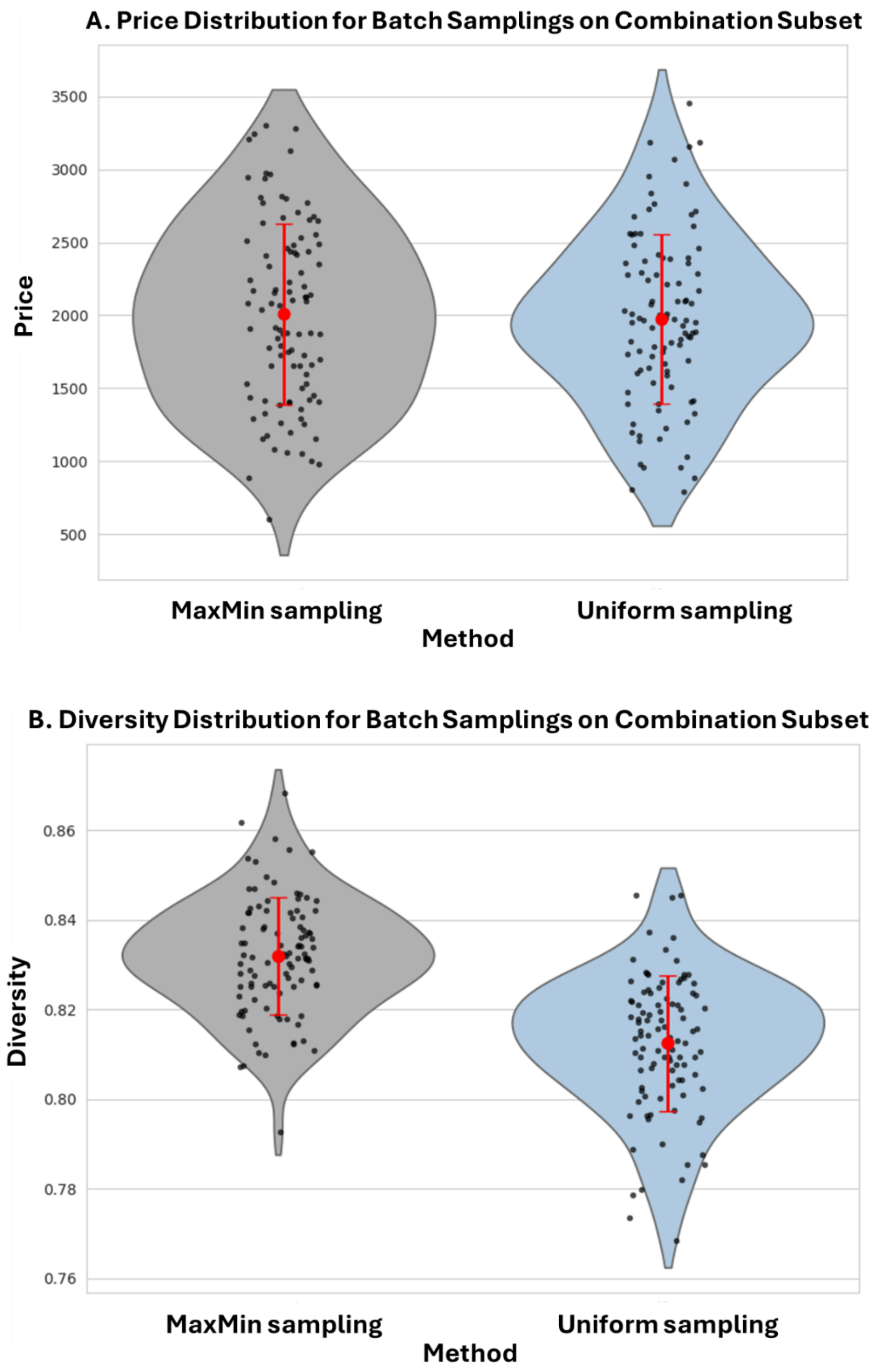

Supplementary Figure 4 Comparison of MaxMin versus uniform sampling applied on the cost-filtered combination subset (128 × 128). Each datapoint represents a batch of 80 reactions. Uniform sampling treats all candidates with equal selection probability (classical random sampling), and MaxMin sampling selects batches by maximising the minimum pairwise distance between candidates (diversity-oriented design). Relative to uniform, MaxMin maintains comparable cost while significantly increasing batch diversity.

Panel A: Total reagent price distribution for batches drawn by MaxMin (grey) versus uniform sampling (blue). Two-sided t-test, p-value = 0.6813

Panel B: Diversity distributions for the same batches, where diversity was computed as the mean pairwise Jaccard distance between MHFP6 fingerprints within each 80-member batch. Two-sided t-test, p-value = $1.91 * 10^{-18}$

C. Experimental Workflow Procedures

1. Automated Reaction Set-up

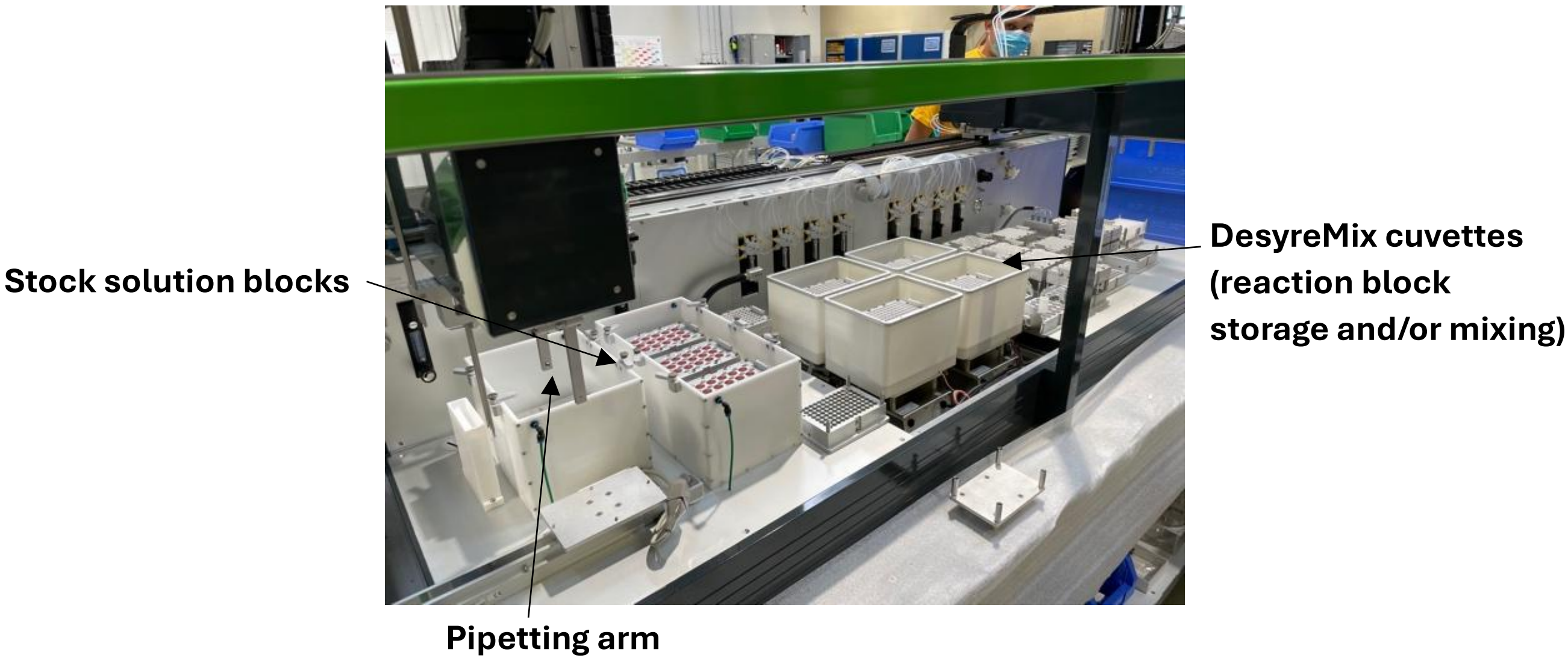

Supplementary Figure 5 Illustration of the Zinsser robot pipetting deck

Supplementary Table 1 Preparation of methanolic stock solutions of reagents for the first round of discovery

ID	SMILES	MW	No of reactions	Mass (mg)	Stock volume (uL)	Stock conc (M)
S1_1-Hy	O=C(C1=CC=C(C(C)(C)C)C=C1)NOC(C(C)(C)C)=O	277	6	93	500	0.67
S1_2-Hy	O=C(C1=C(OC)C(Cl)=CC(Cl)=C1)NOC(C(C)(C)C)=O	320	12	203	950	0.67
S1_3-Hy	O=C(C1=C(C)C(F)=C(F)C=C1)NOC(C(C)(C)C)=O	271	10	145	800	0.67
S1_4-Hy	O=C(C1=CC(C)=CC(C)=C1)NOC(C(C)(C)C)=O	249	6	83	500	0.67
S1_5-Hy	O=C(C1=C(C)C=CC(F)=C1)NOC(C(C)(C)C)=O	253	5	67	425	0.67
S1_6-Hy	O=C(C1=CC=CC(C)=C1)NOC(C(C)(C)C)=O	235	4	55	350	0.67
S1_7-Hy	O=C(NOC(C(C)(C)C)=O)C1=CN2C(C(C)=CC=C2)=N1	275	11	161	875	0.67
S1_8-Hy	O=C(C1=CC=C(CC#N)C=C1)NOC(C(C)(C)C)=O	260	5	74	425	0.67
S1_9-Hy	O=C(NOC(C(C)(C)C)=O)C1=CC=C(C(F)(F)F)S1	295	18	277	1400	0.67
S1_10-Hy	O=C(C1=CC=C(Br)C(F)=C1)NOC(C(C)(C)C)=O	318	3	59	275	0.67
S1_11-Hy	O=C(C1=CC=CC=C1)NOC(C(C)(C)C)=O	221	4	52	350	0.67
Co1_1	C#CCCCC#N	93	5	29	425	0.73
Co1_2	C#CCNC1CCc2ccccc21	171	3	34	275	0.73
Co1_3	C#Cc1ccc(CC#N)cc1	141	1	13	125	0.73
Co1_4	C#Cc1cccc2cccnc12	153	5	47	425	0.73
Co1_5	C#Cc1cccs1	108	5	34	425	0.73
Co1_6	C#Cc1cn2ccnc2cn1	143	4	37	350	0.73
Co1_7	C1=CCN(c2ccccc2)C1	145	4	37	350	0.73
Co1_8	C1=CCOCC1	84	4	21	350	0.73
Co1_9	C=CC(=O)N1CCOCC1	141	5	44	425	0.73
Co1_10	C=CCc1ccccc1	118	2	17	200	0.73
Co1_11	C=Cc1cc[nH]n1	94	3	19	275	0.73
Co1_12	C=CC(=O)NC(C)C	113	2	16	200	0.73

Co1_13	CC1(C)C=Cc2cc(C#N)ccc2O1	185	4	47	350	0.73
Co1_14	CC1CC=CCC1	96	1	10	125	0.73
Co1_15	C#Cc1ccc(N(C)C)cc1	145	1	13	125	0.73
Co1_16	C=CC[C@@H]1CC=C[C@@H](C)N1	137	4	35	350	0.73
Co1_17	C#Cc1cc(C)ccc1C	130	3	26	275	0.73
Co1_18	C=Cc1ccc(C)nc1	119	3	24	275	0.73
Co1_19	C=CC1CC1(Cl)Cl	104	4	27	350	0.73
Co1_20	C=Cc1cc(Cl)ccn1	139	4	36	350	0.73
Co1_21	C#CCOc1cc(F)ccc1F	168	4	43	350	0.73
Co1_22	C#Cc1cc(F)ccc1F	138	1	13	125	0.73
Co1_23	C=Cc1cccc(F)c1	122	3	25	275	0.73
Co1_24	C=CC(F)(F)C(F)(F)Br	206	2	30	200	0.73
Co1_25	C#Cc1cccc(Br)c1	180	3	36	275	0.73
Co1_26	O=C1NC2C=CC1C2	109	1	10	125	0.73
Co1_27	C=CC1=CC=CC=C1	104	1	10	125	0.73
Co1_28	C#CCCO	70	1	7	125	0.73
Co1_29	C1CC=CO1	70	1	7	125	0.73
Rh cat	$[Cp^*RhCl_2]_2$[a]	618	96	61	3314	0.03
Base	CsOPiv	234	96	2242	6386	1.5

[a] Sonicated for 5 min to ensure dissolution

Supplementary Table 2 Preparation of methanolic stock solutions of reagents for the subsequent round of discovery

ID	SMILES	MW	No of reactions	Mass (mg)	Stock volume (uL)	Stock conc (M)
S2_1-Hy	O=C(NOC(C(C)(C)C)=O)c1ccccc1	221	9	109	725	0.67
S2_2-Hy	O=C(NOC(C(C)(C)C)=O)c1ccc(F)cc1	239	4	57	350	0.67
S2_3-Hy	Cc1ccc(C(NOC(C(C)(C)C)=O)=O)cc1	235	6	80	500	0.67
S2_4-Hy	N#CCc1ccc(C(NOC(C(C)(C)C)=O)=O)cc1	260	9	128	725	0.67
S2_5-Hy	O=C(NOC(C(C)(C)C)=O)C1=CC=C(C(F)(F)F)S1	295	6	100	500	0.67
S2_6-Hy	CC1=CC=C(C(NOC(C(C)(C)C)=O)=O)S1	241	2	33	200	0.67
S2_7-Hy	N#CCc1cccc(C(NOC(C(C)(C)C)=O)=O)c1	260	12	167	950	0.67
S2_8-Hy	O=C(NOC(C(C)(C)C)=O)c1ccc(Br)c(F)c1	318	2	43	200	0.67
S2_9-Hy	O=C(NOC(C(C)(C)C)=O)C1=N[N]C(C2=CC=CS2)=C1	292	5	85	425	0.67
S2_10-Hy	O=C(NOC(C(C)(C)C)=O)C1=Cc2ncccc2O1	262	3	49	275	0.67
S2_11-Hy	O=C(NOC(C(C)(C)C)=O)C1=CN2C(CCCC2)=N1	265	9	132	725	0.67
S2_12-Hy	O=C(NOC(C(C)(C)C)=O)C1=CC=CS1	227	6	77	500	0.67
S2_13-Hy	O=C(NOC(C(C)(C)C)=O)c1ccncc1	222	7	87	575	0.67
Co2_1	C#Cc1c(F)c(F)c(F)c(F)c1F	192	3	53	375	0.73
Co2_2	C#Cc1c(C)ccc(C)c1	130	2	29	300	0.73
Co2_3	C#Cc1ccc(C#C)cc1	126	2	28	300	0.73
Co2_4	C#Cc1ccc(C)cc1	116	5	45	525	0.73
Co2_5	C#Cc1cc(C#C)ccc1	126	2	28	300	0.73
Co2_6	C#CC1CCCC1	94	3	26	375	0.73
Co2_7	C#Cc1c(C)cccc1	116	7	57	675	0.73
Co2_8	C#CCOc1c(F)ccc(F)c1	168	2	37	300	0.73
Co2_9	C#CCOc1ccccc1	132	5	51	525	0.73
Co2_10	C=Cc1ccc(F)cc1	122	4	40	450	0.73
Co2_11	C=Cc1ccc(C)cc1	118	5	45	525	0.73

Co2_12	C=Cc1cc(CC2)c2cc1	130	1	21	225	0.73
Co2_13	C=Cc1ccccc1	104	6	46	600	0.73
Co2_14	C=Cc1c(C)cccc1	118	1	19	225	0.73
Co2_15	C=Cc1c(F)cccc1	122	3	33	375	0.73
Co2_16	C/C=C/c1ccccc1	118	2	26	300	0.73
Co2_17	C/C=C/C(C)C	84	2	18	300	0.73
Co2_18	C/C=C/CC#N	81	1	13	225	0.73
Co2_19	C=CC(Cl)(C(F)(Br)F)F	223	3	61	375	0.73
Co2_20	C=Cc1ccncc1	105	1	17	225	0.73
Co2_21	C=Cc1nccnc1	106	4	35	450	0.73
Co2_22	C#CCN(C)C	83	3	23	375	0.73
Co2_23	CC#CC(C)C	82	3	22	375	0.73
Co2_24	CN1C2C=CCC1CC2	123	3	34	375	0.73
Co2_25	N#CC1C[C@H]2C=C[C@@H]1C2	119	3	33	375	0.73
Co2_26	O=C1C=CCCN1	97	4	32	450	0.73
Rh cat	$[Cp^*RhCl_2]_2$ [a]	618	96	61	3314	0.03
Base	CsOPiv	234	96	2242	6386	1.5

[a] Sonicated for 5 min to ensure dissolution

Supplementary Table 3 Dispensing order of reagents

Addition order	Stock solution	Volume per well (μL)	mmol reagent per well
1	$[Cp^*RhCl_2]_2$	34	0.001
2	CsOPiv	66	0.1
3	Hydroxamate (A)	75	0.05
4	Alkene/alkyne (B)	75	0.055

2. Quantification Protocol

2.1. Calibration of Quantification Method

Supplementary Table 4 Preparation of multi-component calibration mixes at a range of concentrations. Seven calibration mixes were prepared, each containing all five components at equal individual concentrations of **0.5, 1.0, 2.5, 5.0, 7.5, 10.0, or 12.5 mg/mL**.

Component in the mix	Source (supplier)	Individual component concentration range (mg/mL)	Solvent system (v:v)
7-hydroxyethyl theophylline[a]	Sigma-Aldrich	0.5–12.5	DMSO:H_2O (80:20)
Hydrocortisone	Sigma-Aldrich		
Dibenzyl 2,3-dihydroxysuccinate	Fluorochem		
Dibenzyl succinate	Fluorochem		
Dibenzyl phthalate	Fisher Scientific		

[a] Sonicated for 5 min to ensure dissolution

1 μL of each of the seven calibration concentration mixes were injected in triplicate onto the UPLC-MS system. The ELSD peak areas and retention times were manually extracted from the generated results files.

Three-dimensional calibration surfaces were generated in Origin, with:

- **X-axis:** retention time (minutes)
- **Y-axis:** log (ELSD peak area)
- **Z-axis:** log (concentration)

Supplementary Equation 1 The calibration surface curve

$$\log(conc) = A + B \times rt + C \times \log_{10}(response\ area) + D \times rt^2 + E \times rt \times \log_{10}(response\ area) + F \times (\log_{10}(response\ area))^2$$

(where *rt* = *retention time*)

2.2. Quantification Method Parameters

Supplementary Table 5 Quantification parameters for crude reaction mixtures

Parameter/Step	Description/Setting
Solvent evaporation	Genevac HT Series 3i; 200 mbar, 40 °C, 30 min
Sample handling	Hamilton liquid handler
Aliquoting	10 μL aliquot dissolved in 40 μL DMSO
Plate format	Shallow 96-wellplate
Analysis method	UPLC
Injection volume	1 μL
Column temperature	C18 column, 40 °C
Detection (PDA) range	210-250 nm
Detection (ELS)	Nebuliser: cooling mode;<br>drift tube: 35 °C;<br>gas pressure: 60 psi;<br>gain: 30;<br>data acquisition rate: 40 Hz
Mobile phase A	H_2O + 0.1 % formic acid
Mobile phase B	MeCN + 0.1 % formic acid
Flow rate	0.8 mL min^{-1}
Gradient / run time	Binary gradient / 3.5 min

3. Purification Method Parameters

Supplementary Table 6 Purification parameters for shortlisted crude reaction mixtures

Parameter/Step	Description/Setting
Sample selection	Reactions with >15% analytical yield of new product(s) by MW
Plate format	Deep 96-wellplate
Sample handling	Preparative HPLC
Injection volume	240 μL
Column temperature	C18 column, 40 °C
Detection (PDA) range	210-250 nm
Detection (ELS)	Nebuliser: cooling mode;<br>drift tube: 35 °C;<br>gas pressure: 60 psi;<br>gain: 30;<br>data acquisition rate: 40 Hz
Mobile phase A	H_2O + 0.1 % formic acid
Mobile phase B	MeCN + 0.1 % formic acid
Flow rate	25 mL min^{-1}
Gradient / run time	Binary gradient / 17.5 min

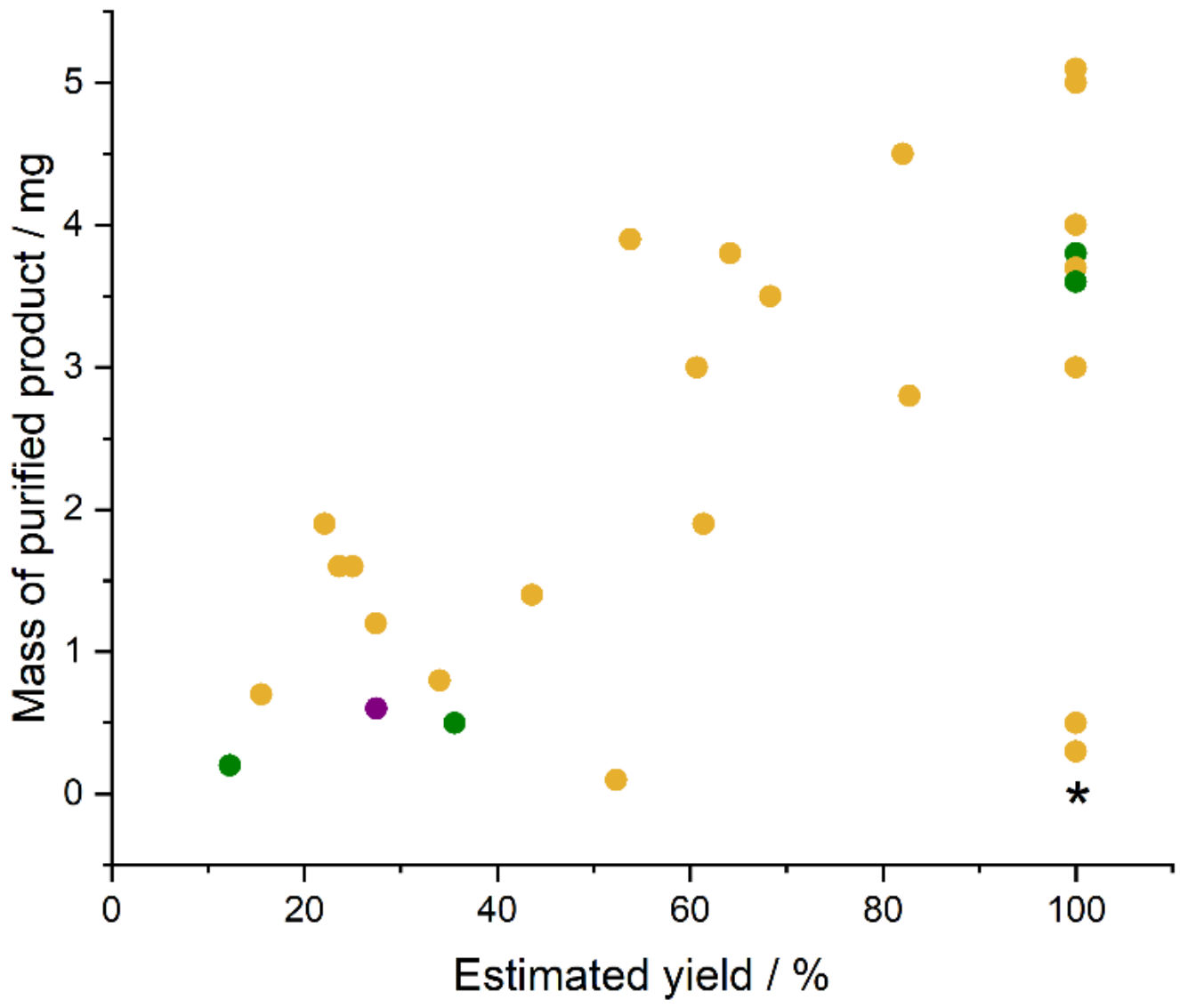

Supplementary Figure 6 ELSD-estimated yields and masses of purified products obtained in round one. *Estimated yield >100%. Activity in the cell painting assay at 10 μM is shown by colour: yellow, inactive; green, hits with >70% similarity to tubulin cluster; purple, hit with <70% similarity to any defined functional cluster.

4. Weighing, Drying and Plating of Isolated Products

Supplementary Table 7 Purification parameters for shortlisted crude reaction mixtures

Parameter/Step	Description/Setting
Sample handling	HTZ Selecta equipped with tube balance and barcode-guided sample handling
Tube labelling	Thermal barcode printer; empty (tare) weight recorded
Fraction tracking	Post-HPLC fractions identified and tracked by barcode
Solvent evaporation	Genevac HT Series 3i;<br>200 mbar, 40 °C, 16 h
Weighing protocol	Dried fractions reweighed three times using HTZ Selecta; mean value used to calculate net sample weight
Concentration calculation	Excel spreadsheet generated to determine acetonitrile (MeCN) volume for 10 mM final concentration
Dissolution step	Hamilton Microlab STAR used to add MeCN; included 1–2 min induction time with repeated aspiration/dispensing to ensure dissolution
Transfer to plate	50 µL of each solution transferred into shallow well plate
Stock storage	Remaining product stock solutions stored in deep well plates and catalogued
Second evaporation	Shallow well plates evaporated overnight in Genevac (spinning only)
Post-processing	Plates sealed and submitted for biological screening

5. Methods for Cell Painting Assay

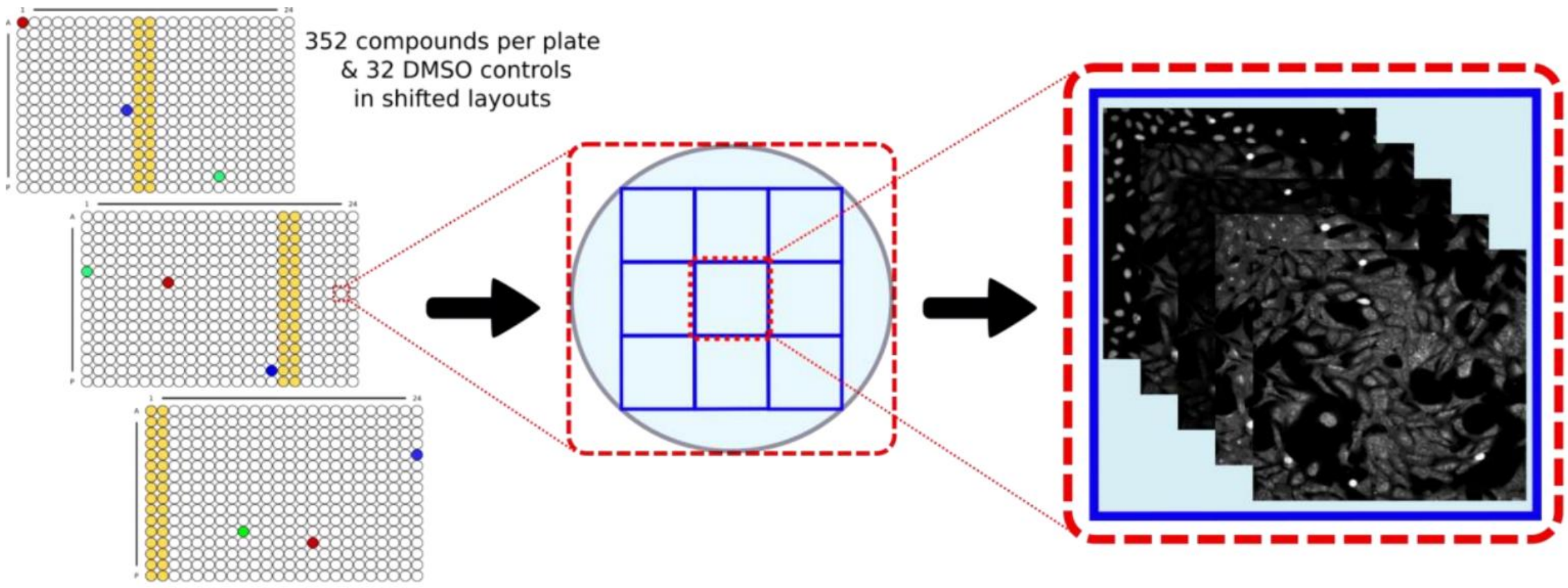

Supplementary Figure 7 Overview of Cell Painting assay plate layout and image acquisition workflow.[1]

Supplementary Table 8 Experimental parameters for the Cell Painting assay

Parameter/Step	Description/Setting
Cell line	U2OS (human osteosarcoma)
Plate format	384-well CellCarrier Ultra plate (PerkinElmer)
Cell seeding	1,600 cells per well in 20 µL medium (5 µL pre-wet medium per well prior to seeding).
Pre-incubation	10 min ambient, then 4 h at 37 °C, 5 % CO_2
Compound treatment	Echo 520 acoustic dispenser (Labcyte); final concentrations 1–10 µM; incubation 20 h (37 °C, 5 % CO_2)
Staining panel	Multiplexed fluorescent dyes labelling nuclei, mitochondria, actin, and membranes
Mitochondrial staining	MitoTracker Deep Red (Thermo Fisher Scientific, Cat. No. M22426);<br>Stock: 1 mM; final concentration: 100 nM in prewarmed medium;<br>25 µL MitoTracker solution added per well; incubated 30 min in darkness (37 °C, 5 % CO_2).
Fixation	3.7 % formaldehyde in PBS (from 18.5 % stock), 20 min at ambient temperature in darkness
Permeabilization	0.1 % Triton X-100 in PBS, 15 min at ambient temperature in darkness
Blocking and staining	25 µL of staining solution containing 1 % BSA in buffer and the following fluorescent conjugates:

	Hoechst 33342 (5 µg mL⁻¹; Sigma, Cat. No. B2261-25 mg)<br><br>Phalloidin–Alexa 594 (5 µL mL⁻¹; Thermo Fisher Scientific, Cat. No. A12381)<br><br>Concanavalin A–Alexa 488 (25 µg mL⁻¹; Thermo Fisher Scientific, Cat. No. C11252)<br><br>WGA–Alexa 594 (1.5 µg mL⁻¹; Thermo Fisher Scientific, Cat. No. W11262)<br><br>SYTO 14 (1.5 µM; Thermo Fisher Scientific, Cat. No. S7576)<br><br>The plate was incubated for 30 min at room temperature in darkness.
Washing	3 × 70 µL PBS (BioTek Elx405 washer)
Final handling	PBS left in wells post-wash; plates sealed and centrifuged 1 min at 500 rpm
Imaging system	Molecular Devices ImageXpress Micro XL; 20× objective; 9 sites/well; 5 channels (DAPI: Ex350-400/ Em410-480; FITC: Ex470-500/ Em510- 540; Spectrum Gold: Ex520-545/ Em560-585; TxRed: Ex535-585/ Em600-650; Cy5: Ex605-650/ Em670-715)
Image analysis	CellProfiler v3.0.0; 1,716 features/site aggregated per well and replicate
Feature curation	Robustness filtering;[2] 579 reproducible features retained
Cell line	U2OS (human osteosarcoma)

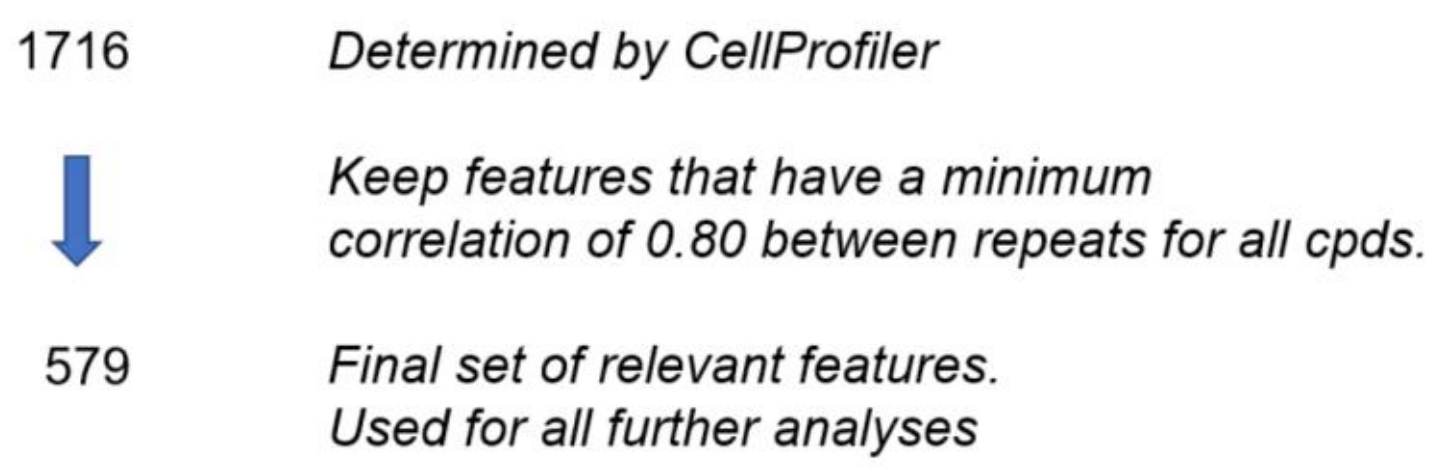

Supplementary Figure 8 Feature extraction and selection workflow. Images were processed with *CellProfiler* (v3.0.0) to extract 1,716 morphological features per site. Features were aggregated as medians per well (9 sites per well) and then over the 3 replicates. Data analysis: Python (pandas, dask, scipy). Reproducibility filtering, following Woehrmann et al.[2] retained features showing a similarity ≥ 0.8 between biological repeats of reference plates, yielding 579 robust features used for all subsequent analyses**.**

Supplementary Equation 3 Calculation of Z-scores for phenotypic features. Z-scores were calculated as the difference between the measured feature value and the control median, normalised by the median absolute deviation (MAD) of the controls. This defines how many MADs a feature value is away from the control median, as illustrated below. The phenotypic compound profile is then determined as the list of Z-scores of all features for one compound.

$$z\_score = \frac{\text{value}_{\text{measured}} - median_{controls}}{\text{MAD}_{\text{controls}}}$$

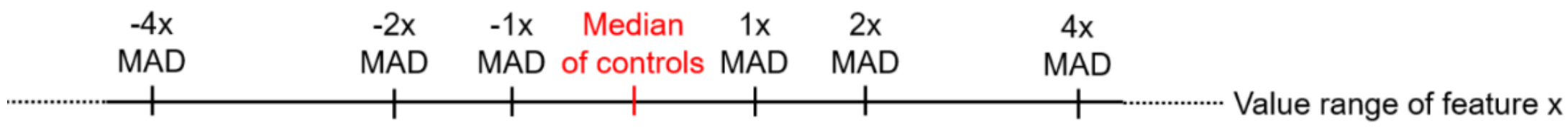

Supplementary Equation 9 An induction value was determined for each compound as the fraction of significantly changed features, in percent

$$Induction\ [\%] = \frac{\text{number of features with abs. values} > 1}{\text{total number of features}}$$

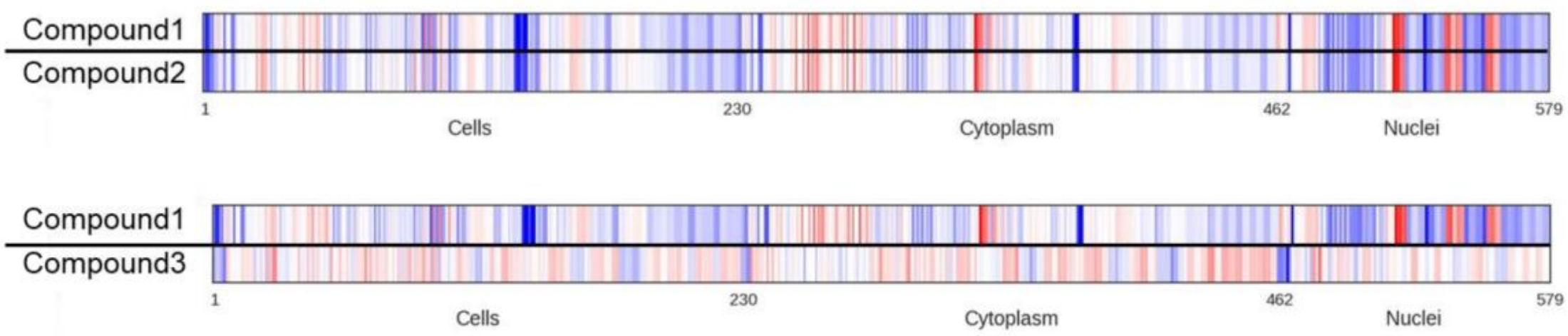

Supplementary Figure 10 Similarities between phenotypic profiles were computed using correlation distance (*scipy.spatial.distance.correlation*; Similarity = 1 − Correlation Distance). Each coloured band represents the Z-score of an individual morphological feature. Shown are examples of two compounds with high (top, 96 % similarity) and low (bottom, 0 % similarity) phenotypic similarity.

6. KNIME Workflow for Filtering Substrates/Co-substrates

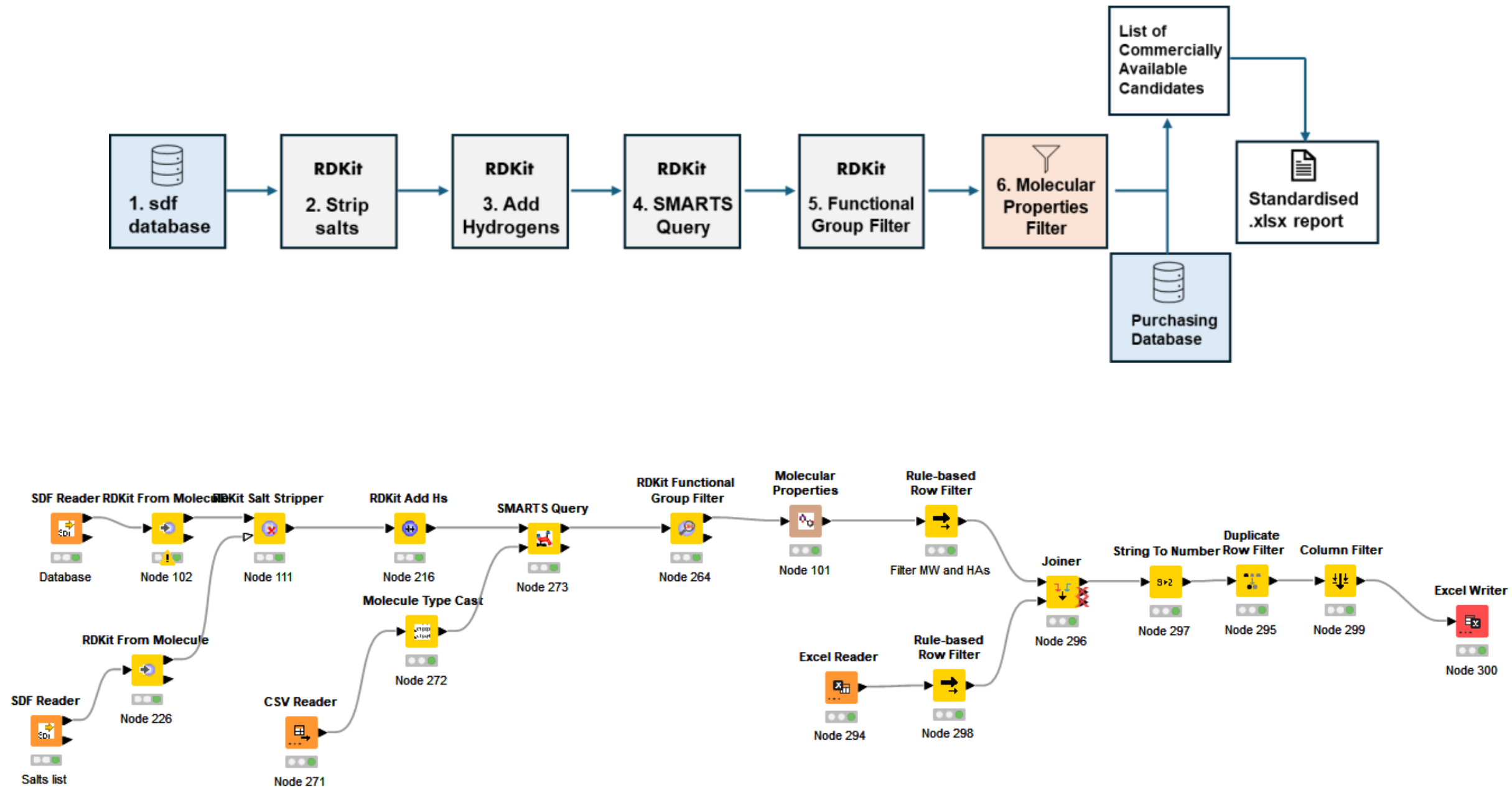

Supplementary Figure 11 Schematic overview of the KNIME workflow. The KNIME workflow and supporting files are available on GitHub (link). The top panel shows a conceptual design of the workflow, while the bottom panel displays a screenshot of the implemented pipeline within the KNIME environment.

SDF Reader - Reads in the input database of molecular structures from an SDF (Structure Data File) format. This serves as the starting point of the workflow, importing chemical information such as atom coordinates, connectivity, and metadata.

RDKit From Molecule - Converts the imported molecular structures into RDKit-compatible objects, enabling further cheminformatics operations using RDKit's toolkit. This step ensures compatibility and standardisation across subsequent nodes.

RDKit Salt Stripper - Removes salts and counterions from the molecules to improve chemical consistency. The node uses RDKit's standard salt list by default but can also be configured with a user-defined list of undesired fragments, typically read via an additional SDF Reader node and merged.

RDKit Add Hs - Adds explicit hydrogen atoms to the molecular structures. This step is critical for accurate substructure searching, as it ensures hydrogen counts are properly considered during SMARTS-based matching and reduces false negatives due to implicit hydrogen discrepancies.

SMARTS Query - Applies user-defined SMARTS (SMiles ARbitrary Target Specification) patterns to search for specific substructures within the molecules. The SMARTS patterns are imported from a CSV file and can represent functional groups, scaffolds, or motifs of interest. This enables highly customisable substructure filtering.

Functional Group Filter - Filters out molecules containing reactive or undesirable functional groups. The filter contains an editable list of SMARTS patterns representing these groups, and a dropdown interface allows users to enable or disable specific exclusions without modifying the SMARTS file directly.

Molecular Properties - Calculates key physicochemical properties of each molecule, including molecular weight (MW), hydrogen bond donors (HBDs), and acceptors (HBAs). These computed values are essential for evaluating drug-likeness and other developability criteria.

Rule-based Row Filter - Applies filtering rules to retain only molecules that meet predefined property thresholds (e.g., $MW < 500$, $HBD \leq 5$). This step narrows the dataset to compounds with favourable developability profiles.

Excel Reader - Loads a list of commercially available compounds from an Excel file, typically representing the internal or external procurement catalogue.

Joiner - Matches the filtered set of molecules against the commercial availability list. Only compounds that exist in both datasets are retained, ensuring that subsequent selections are available for purchase.

String to Number, Duplicate Row Filter, Column Filter - Converts text-based values into numerical formats as needed for downstream operations or reporting, ensuring data consistency. Removes duplicate molecular entries to prevent redundancy and ensure unique compound representation in the final dataset. Eliminates unnecessary columns from the dataset, simplifying the output and focusing only on relevant information for reporting or export.

Excel Writer - Outputs the final, curated list of compounds to an Excel file. This file includes all necessary annotations and filtered data, ready for reporting, sharing, or further analysis.

Supplementary Table 9 Functional group dictionary, adapted from the version created by Greg Landrum, 2006, Copyright (c) 2010, Novartis Institutes for BioMedical Research Inc.

Functional Group	SMARTS
**Alkenes/Alkynes/Conjugates**	
Alkene	[CX3]=[CX3]
Alkene.Substituted	[CX3]=C(C)(C)
Alkene.Cyclic	C[CX3]=C(c)(C)
Alkenyl.Halide	[F,Cl,Br,I][CX3]=[CX3]
Diene	[C]=[C][C]=[C]
Alkyne	[$([CX2]#C)]
Ethyl chain	[*][CH2][CH3]
Isopropyl chain	[*][CH]([CH3])([CH3])
Cyclopropyl	C1CC1
Cyclobutyl	C1CCC1
Cyclopentyl	C1CCCC1
**Carboxylic Acids & Derivatives**	
CarboxylicAcid	C(=O)[O;H,-]
Carboxylate	[CX3](=[OX1])O
CarboxylicAcid.Aromatic	[$(C-!@[a])](=O)([O;H,-])
CarboxylicAcid.Aliphatic	[$(C-!@[A;!O])](=O)([O;H,-])
CarboxylicAcid.AlphaAmino	[$(C-[C;!$(C=[!#6])]-[N;!H0;!$(N-[!#6;!#1]);!$(N-C=[O,N,S])])](=O)([O;H,-])
CarboxylicAcid.AlphaHalo	[F,Cl,Br,I]cc([$(C-!@[a])](=O)([O;H,-]))
**Acid Derivatives**	
AcidChloride	C(=O)Cl
AcidChloride.Aromatic	[$(C-!@[a])](=O)(Cl)
AcidChloride.Aliphatic	[$(C-!@[A;!Cl])](=O)(Cl)
Anhydride	[CX3](=[OX1])[OX2][CX3](=[OX1])
Ester	[CX3](=O)[OX2H0][#6]
Ester.Cyclic	[CX3;R](=O)[OX2H0;R]
Lactone	[#6;r](=[OX1])[oX2;r]
Peracid	C(=O)OO[H]
CarboxylicPhosphate	C(=O)O[P]
SulfonylChloride	[$(S-!@[#6])](=O)(=O)(Cl)
SulfonylChloride.Aromatic	[$(S-!@c)](=O)(=O)(Cl)
SulfonylChloride.Aliphatic	[$(S-!@C)](=O)(=O)(Cl)
**Amines**	
Amine	[N;!H0;$(N-[#6]);!$(N-[!#6;!#1]);!$(N-C=[O,N,S])]
Amine.Aromatic	[N;!H0;$(N-c);!$(N-[!#6;!#1]);!$(N-C=[O,N,S])]
Amine.Aliphatic	[N;!H0;!$(N-c);$(N-C);!$(N-[!#6;!#1]);!$(N-C=[O,N,S])]
Amine.Primary	[N;H2;D1;$(N-!@[#6]);!$(N-C=[O,N,S])]
Amine.Primary.Aromatic	[N;H2;D1;$(N-!@c);!$(N-C=[O,N,S])]
Amine.Primary.Aliphatic	[N;H2;D1;$(N-!@C);!$(N-C=[O,N,S])]
Amine.Secondary	[N;H1;D2;$(N(-[#6])-[#6]);!$(N-[!#6;!#1]);!$(N-C=[O,N,S])]
Amine.Secondary.Aromatic	[N;H1;D2;$(N(-[c])-[#6]);!$(N-[!#6;!#1]);!$(N-C=[O,N,S])]
Amine.Secondary.Aliphatic	[N;H1;D2;$(N(-C)-C);!$(N-[!#6;!#1]);!$(N-C=[O,N,S])]
Amine.Cyclic	[N;!H0;R;$(N-[#6]);!$(N-[!#6;!#1]);!$(N-C=[O,N,S])]
**Boronic Acids**	
BoronicAcid	[$(B-!@[#6])](O)(O)
BoronicAcid.Aromatic	[$(B-!@c)](O)(O)
BoronicAcid.Aliphatic	[$(B-!@C)](O)(O)
**Isocyanate**	
Isocyanate	[$(N-!@[#6])](=!@C=!@O)
Isocyanate.Aromatic	[$(N-!@c)](=!@C=!@O)
Isocyanate.Aliphatic	[$(N-!@C)](=!@C=!@O)
**Alcohols/Acetals/Epoxides**	

Alcohol	[O;H1;$(O-!@[#6;!$(C=!@[O,N,S])])]
Alcohol.Aromatic	[O;H1;$(O-!@c)]
Alcohol.Aliphatic	[O;H1;$(O-!@[C;!$(C=!@[O,N,S])])]
Acetal	[CX4H1,H2,H3](-O)(-O)
Epoxide	[$([C;r3]-[O;r3]-[C;r3])]
**Carbonyl Compounds**	
Aldehyde	[CX3H1](=O)[#6,#7]
Aldehyde.Aromatic	[CH;D2;$(C-!@[a])](=O)
Aldehyde.Aliphatic	[CH;D2;$(C-!@C)](=O)
Ketone	[#6][CX3](=O)[#6]
Enone	[#6]=[#6]-[#6](=O)
Enone.Aromatic	[c]=[C]-[C](=O)
Ene-dione	[CX3](=O)C=C[CX3](=O)
**Amides/Imides/Ureas**	
Amide	[NX3][CX3]=[OX1]
Acrylamide	[CX3]=C[CX3](=[OX1])[NX3]
Diacrylamide	C=CC(=O)N1CCN(C(=O)C=C)CC1
Imide	[CX3](=[OX1])[NX3H0]([#6])[CX3](=[OX1])
Urea	[#7][#6](=O)[#7]
Thioguanidine ester	[NX3]=[CX3]([NX3])[SX2]
**Halogens**	
Halogen	[$([F,Cl,Br,I]-!@[#6]);!$([F,Cl,Br,I]-!@C-!@[F,Cl,Br,I]);!$([F,Cl,Br,I]-[C,S](=[D1;O,S,N]))]
Halogen.Aromatic	[F,Cl,Br,I;$(*-!@c)]
Halogen.Aliphatic	[$([F,Cl,Br,I]-!@C);!$([F,Cl,Br,I]-!@C-!@[F,Cl,Br,I])]
Halo-2-N-Hetarene	[n][c]([F,Cl,Br,I])[c,n]
Iodine.Hypervalent	[I]=O
**Nitrogen-Containing Groups**	
Azide	[NX2,NX1-]=[NX2+]=[NX1,NX1-]
Diazo	[$([#6]=[N+]=[N-]),$([#6-]-[N+]#[N])]
Nitro	[$([NX3](=O)=O),$([NX3+](=O)[O-])][!#8]
Nitrate	[$([NX3](=[OX1])(=[OX1])O),$([NX3+]([OX1-])(=[OX1])O)]
Nitroso	[NX2](=O)
N-oxide	[$([#7+][OX1-]),$([#7v5]=[OX1])]
Oxime	[CX3](=N-O)
Hydrazine	[NX3][NX3]
Hydrazone	[NX3][NX2]=[*]
Hydroxylamine	[NX3][OX2]
Hydrazine	[NX3][NX3]
Enamine	[n,NX3][CX3]=[CX3]
Amidine	[NX3][CX3]=[NX2]
**Sulfur Compounds**	
SulfonylChloride	[$(S-!@[#6])](=O)(=O)(Cl)
SulfonylFluoride	[$(S-!@[#6])](=O)(=O)(F)
Sulfonamide	[$([#16X4]([NX3])(=[OX1])(=[OX1])[#6]),$([#16X4+2]([NX3])([OX1-])([OX1-])[#6])]
Sulfonic acid/ester	[OX2,-]S(=O)(=O)(*)
Sulfone	[$([#16X4](=[OX1])(=[OX1])([#6])[#6])]
Sulfoxide	[$([#16X3]=[OX1]),$([#16X3+][OX1-])]
Sulfinate	[$([#16X3](=[OX1])[OX2H0]),$([#16X3+]([OX1-])[OX2H0])]
Thiol	[#16!H0]
Isothiocyanate	[NX2]=[C]=[S]
Thiourea	[NX3][CX3]=[SX1][#6]
Thioamide	[NX3][CX3]=[SX1]
Thione	[#6]=S

Sulfide	[c,C]SC
**Rings and Heterocycles**	
Dihydropyrrole	C1C=CC[NH]1
2-Pyridone	O=c1n*ccc1
4-Pyridone	O=c1ccncc1
Pyran-one	O=c1ccocc1
All substituted benzene	[cH0]1[cH0][cH0][cH0][cH0][cH0]1
**Miscellaneous**	
Isonitrile	[CX1-]#[NX2+]
Ene.Nitrile	[CX3]=[CX3]-C#N
N-Carbonyl.Amine	*C(=O)N(C(*)([H])[H])C(*)([H])[H]
N-Boc.Aliphatic.Amine	*C([H])([H])N(C(=O)OC(C)(C)C)C(*)([H])[H]
N-Acyl.Aliphatic.Amine	*C([H])([H])N(C(=O)C([H])([H])[H])C(*)([H])[H]
N-Chloroacetamide	*C([H])([H])N(C(=O)C[Br,I,Cl,F])C(*)([H])[H]
Aromatic N+	[n+]
Aliphatic N+	[N+]
**Metals/Elements**	
Zinc	[Zn]
Magnesium	[Mg]
Lithium	[Li]
Tin	[Sn]
Copper	[Cu]
Silicon	[Si]
Palladium	[Pd]
Boron	[B]
Ruthenium	[Ru]
Germanium	[Ge]
Aluminium	[Al]
Deuterium	[2H]
13C	[13c,13C]
15N	[15n,15N]

Supplementary Table 10 Node configuration for carboxylic acid (species A, Figure 3A) filtering

Node/Step	Description/Configuration		
**SMARTS Query**	[cH][c]C(=O)[O;H,-]<br>Selects aromatic carboxylic acids with at least one available tertiary α-CH		
**Functional Group Filter**	All dictionary SMARTS **excluded except** those listed below:		
	**Functional Group**	**SMARTS**	
	CarboxylicAcid	C(=O)[O;H,-]	
	Acetal	[CX4H1,H2,H3](-O)(-O)	
	Amide	[NX3][CX3]=[OX1]	
	Halogen	[$([F,Cl,Br,I]-!@[#6]);!$([F,Cl,Br,I]-!@C-!@[F,Cl,Br,I]);!$([F,Cl,Br,I]-[C,S](=[D1;O,S,N]))]	
	Halogen.Aromatic	[F,Cl,Br,I;$(*-!@c)]	
	N-Carbonyl.Amine	*C(=O)N(C(*)([H])[H])C(*)([H])[H]	
	N-Boc.Aliphatic.Amine	*C([H])([H])N(C(=O)OC(C)(C)C)C(*)([H])[H]	
	N-Acyl.Aliphatic.Amine	*C([H])([H])N(C(=O)C([H])([H])[H])C(*)([H])[H]	
**Molecular Property Calculations**	**Property**	**Description**	
	Heavy Atoms Count	Total number of non-hydrogen atoms	
	XLogP	Predicted partition coefficient (lipophilicity)	
	Rotatable Bonds Count (non-terminal)	Count of rotatable bonds excluding terminal groups	
**Rule-Based Row Filter Conditions**	**Property**	**Filter Condition**	**Include If**
	Heavy Atoms Count	≥6	TRUE
	Heavy Atoms Count	≤13	TRUE
	XLogP	≥-0.5	TRUE
	XLogP	≤3.5	TRUE
	Aromatic Atoms	≥5	TRUE
	Aromatic Atoms	≤12	TRUE
	Rotatable Bonds (non-terminal)	≤2	TRUE

Supplementary Table 11 Node Configuration for alkene/alkyne (species B, Figure 3A) filtering

**Node/Step**	**Description/Configuration**		
**SMARTS Query**	[C;H1,H2]=[C;H1,H2] and [C;H0,H1]#[C;H0,H1]<br>Selects any alkene/alkyne with no more than one substituent at each end		
**Functional Group Filter**	All dictionary SMARTS **excluded except** those listed below:		
	**Functional Group**	**SMARTS**	
	Alkene	[CX3]=[CX3]	
	Alkene.Substituted	[CX3]=C(C)(C)	
	Alkene.Cyclic	C[CX3]=C(c)(C)	
	Alkyne	[$([CX2]#C)]	
	Ethyl chain	[*][CH2][CH3]	
	Isopropyl chain	[*][CH]([CH3])([CH3])	
	Cyclopropyl	C1CC1	
	Cyclobutyl	C1CCC1	
	Cyclopentyl	C1CCCC1	
	Amine	[N;!H0;$(N-[#6]);!$(N-[!#6;!#1]);!$(N-C=[O,N,S])]	
	Amine.Aromatic	[N;!H0;$(N-c);!$(N-[!#6;!#1]);!$(N-C=[O,N,S])]	
	Amine.Aliphatic	[N;!H0;!$(N-c);$(N-C);!$(N-[!#6;!#1]);!$(N-C=[O,N,S])]	
	Amine.Secondary	[N;H1;D2;$(N(-[#6])-[#6]);!$(N-[!#6;!#1]);!$(N-C=[O,N,S])]	
	Amine.Secondary.Aromatic	[N;H1;D2;$(N(-[c])-[#6]);!$(N-[!#6;!#1]);!$(N-C=[O,N,S])]	
	Amine.Secondary.Aliphatic	[N;H1;D2;$(N(-C)-C);!$(N-[!#6;!#1]);!$(N-C=[O,N,S])]	
	Amine.Cyclic	[N;!H0;R;$(N-[#6]);!$(N-[!#6;!#1]);!$(N-C=[O,N,S])]	
	Enone	[#6]=[#6]-[#6](=O)	
	Enone.Aromatic	[c]=[C]-[C](=O)	
	Amide	[NX3][CX3]=[OX1]	
	Acrylamide	[CX3]=C[CX3](=[OX1])[NX3]	
	Halogen	[$([F,Cl,Br,I]-!@[#6]);!$([F,Cl,Br,I]-!@C-!@[F,Cl,Br,I]);!$([F,Cl,Br,I]-[C,S](=[D1;O,S,N]))]	
	Halogen.Aromatic	[F,Cl,Br,I;$(*-!@c)]	
	N-Carbonyl.Amine	*C(=O)N(C(*)([H])[H])C(*)([H])[H]	
	N-Boc.Aliphatic.Amine	*C([H])([H])N(C(=O)OC(C)(C)C)C(*)([H])[H]	
	N-Acyl.Aliphatic.Amine	*C([H])([H])N(C(=O)C([H])([H])[H])C(*)([H])[H]	
**Molecular Property Calculations**	**Property**	**Description**	
	Heavy Atoms Count	Total number of non-hydrogen atoms	
	XLogP	Predicted partition coefficient (lipophilicity)	
	Rotatable Bonds Count (non-terminal)	Count of rotatable bonds excluding terminal groups	
**Rule-Based Row Filter Conditions**	**Property**	**Filter Condition**	**Include If**
	Heavy Atoms Count	≥6	TRUE

	Heavy Atoms Count	≤14	TRUE
	XLogP	≥-1.5	TRUE
	XLogP	≤3.5	TRUE
	Rotatable Bonds (non-terminal)	≤2	TRUE

The property ranges from Supplementary Tables 10 and 11 were selected to:

- Focus on chemical space of small to medium fragments to ensure further developability
- ensure solubility in MeOH (XLogP window)
- limit conformational flexibility (≤2 rotatable bonds)

7. Methods for Cell Biology

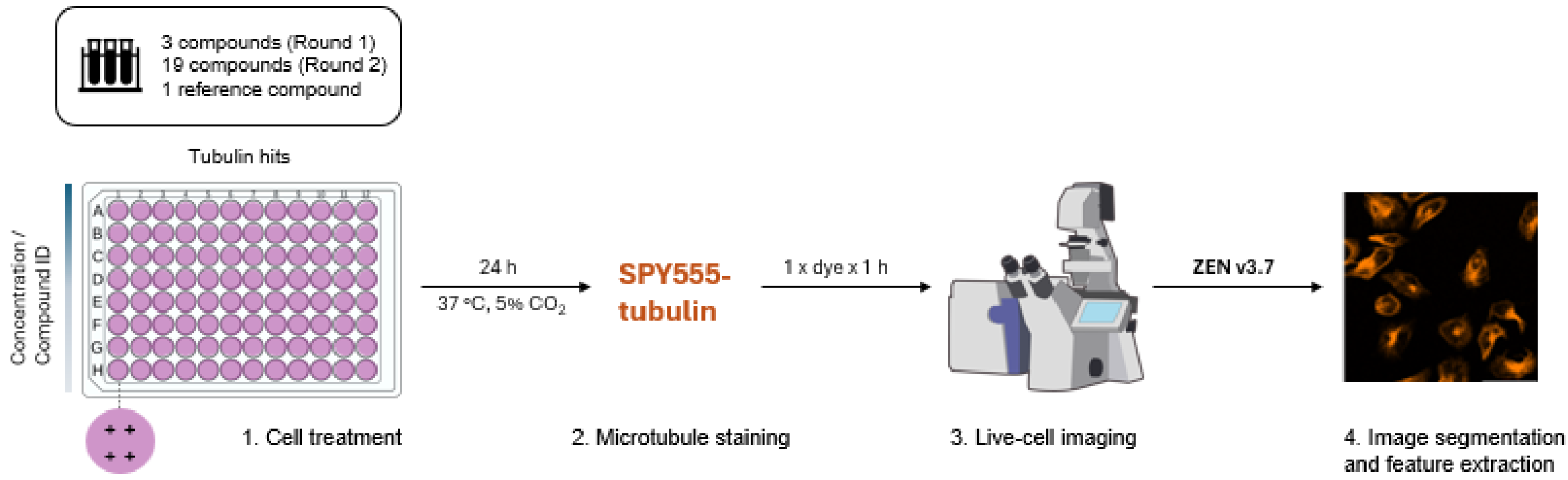

Supplementary Figure 12 Schematic illustration of the workflow for microscopic analysis of microtubule alterations in HeLa cells 1. HeLa cells were treated in 96-well plates with varying compounds or concentrations of selected hits. 2. Microtubules (MT) were labelled using a cell-permeable, tubulin-specific fluorescent probe. 3. Labelled cells were imaged on a confocal microscope, acquiring 4 fields per well with autofocus set to the SYP-555 fluorescence signal. 4. Images were processed and segmented in ZEN software, and data were aggregated by field, well, and compound ID.

A) Live cell microtubule (MT) imaging

HeLa cells were maintained in DMEM high-glucose medium (Thermo Fisher, Life Technologies) supplemented with 10% fetal bovine serum (GE Hyclone, Cytiva) at 37°C in a humidified 5% CO_2 incubator. For microtubule (MT) labelling, cells were counted in haemocytometer and seeded (1.9 x 10^3 cells/well) onto film-bottom chimney 96-well black plates (Greiner, cat No 655866). Cells were incubated overnight prior to compound addition.

Test compounds were prepared as 10 mM DMSO stocks and diluted to 20× working solutions (e.g., 15 μM stocks for a 750 nM test) in a final volume of 200 μl per well; the final DMSO concentration did not exceed 0.05% (v/v). After 24 h compound exposure, cells were washed three times with 150 μl pre-warmed PBS buffer and resuspended in 60 μl of the cell permeable **SYP555-tubulin** (Spirochrome) probe (Abs_{555nm}/Em_{580nm}). Briefly, the probe was diluted to 1X in fresh DMEM and incubated for 50 min. DNA was labelled adding 5 μg/ml of Hoechst 33342 dye (TargetMol Chemicals Inc) and incubated for additional 10 minutes.

Following dye incubation, cells were washed twice with PBS solution and resuspended in 150 μl of Hanks' balanced salt solution for further imaging. Fluorescence microscopy data were acquired in an Axio observer LSM 980 confocal microscope (Carl Zeiss) under 20X magnification lens and UV (405 nm), AF555 (561 nm), oblique (PMT) detection filters. A tile of 384 acquisition events was set where 4 fields per well were acquired in duplicate for each treatment (Supp Fig. 1).

B) MT imaging data analysis

The resulting images were processed with ZEN software v3.7 (Carl Zeiss) using the trainable annotation tool ZEN Intellesis for image segmentation. Classes of interest corresponding to MT-labelling, DNA and background were defined from DMSO and nocodazole-treated cells. Once segmentation was completed, multiple biological features were measured and values

corresponding to individual objects (cells) were exported in tabular format. Data were aggregated by calculating median values per field, and then over the two replicates.

C) Immunofluorescence microscopy

HeLa cells (1x 10^5 cells/well) were seeded onto sterile glass cover slips and incubated overnight in 12-well plates using standard DMEM. Hit compounds were added (1 μM) and incubated for 24h at 37°C degrees. Following compound treatment, cells were fixed using image iT 4% formaldehyde solution in PBS (Fisher Scientific Ltd) for 1h at room temperature and then permeabilised with 0.1% Triton X-100 in PBS (15 min). Cells were washed three times in PBS buffer prior to addition of blocking solution (10% bovine serum albumin, 99.9% purity) (Sigma Aldrich). Antibody labelling was performed using a mix of anti-alpha-tubulin FITC (Thermo, Fisher Scientific Ltd) (1:1,000) and the anti-histone H3 (phospho-Ser10) (Abcam) diluted (1:400) in a solution of freshly prepared 2% BSA in PBS (w/v), and incubated overnight at 4°C. Coverslips were washed five times with PBS and probed against goat anti-rabbit IgG secondary antibody conjugated to Alexa Fluor 565 (1: 800) (Thermo, Fisher Scientific Ltd) for 1h at room temperature. DNA was counterstained by adding 0.2 μg/ml of DAPI solution in PBS (Thermo, Fisher Scientific Ltd) for five minutes. Cover slips were washed out with PBS solution and mounted on glass slides using Everbrite™ (Biotium) wet set mounting media and sealed with Covergrip™ (Biotium). Samples were imaged in an Axio observer LSM 980 confocal microscope (Carl Zeiss) under 40X magnification lens and the UV (465 nm), AF555 (603 nm), and FITC (517 nm) detection filters. Z-stacks were analysed using Fiji software version 1.54p (htttp://imagej.org) and red channel projection was used to quantify phospho-histone H3 foci using the 'Find Maxima' tool (prominence >15, single points, strict) for individual snapshots. Data was analysed in Microsoft Excel and box plot was produced using Origin Pro 2024b (OriginLab).

D) *In vitro* tubulin polymerisation assay

Tubulin polymerisation rates were measured spectrophotometrically as described previously. Tubulin was kindly provided by Prof Michelle Peckham research group (Faculty of Biological Sciences, University of Leeds), following a previously described methodology.[3] Active tubulin was purified using a glycerol cushion (60% v/v) and ultracentrifugation cycles after GTP-induced polymerisation at 37°C, as previously described.[4] Recycled tubulin was dissolved in 1x IB buffer (containing 50 mM potassium glutamate and 0.5 mM $MgCl_2$ pH 7.0) and snap frozen in aliquots of approximately 25 μM (ε_{280nm} = 115,000 M^{-1} cm^{-1}). Tubulin polymerisation was measured in BRB80 buffer (80 mM K-PIPES pH 6.9, 2 mM $MgCl_2$ and 0.5 mM EGTA) adding 10 μM of tubulin per reaction on a 96-well plate. Compounds were added to the plate and incubated for 20 minutes at room temperature. Then, plate was transferred to ice and incubated for 20 minutes prior to addition of 0.5 mM GTP (freshly thawed). Tubulin polymerisation rates were measured for 1h by means of turbidity at 340 nm in a plate reader (Perkin Elmer).

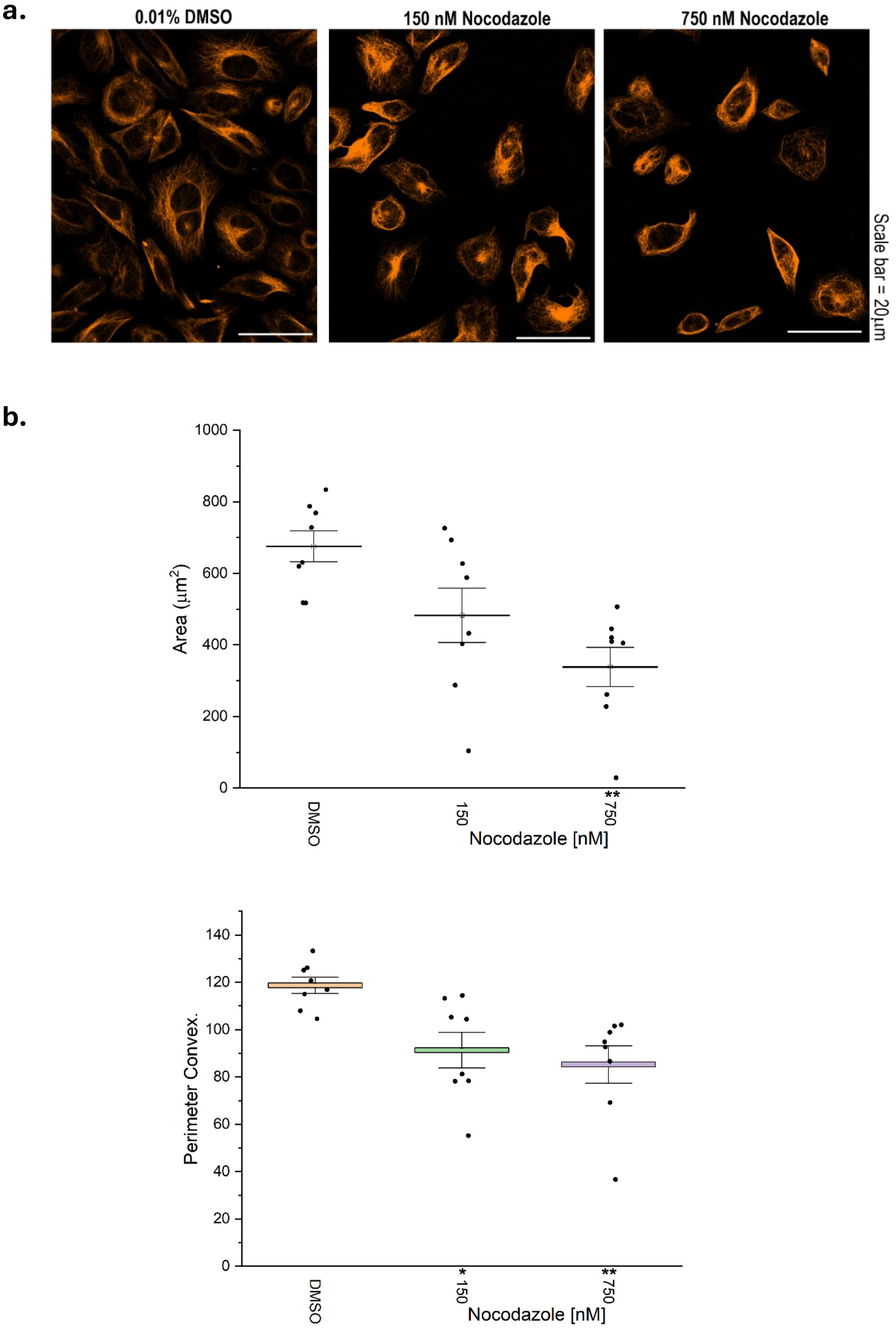

Supplementary Figure 13 Microtubule analysis of live HeLa monolayers under chemical perturbation with nocodazole.

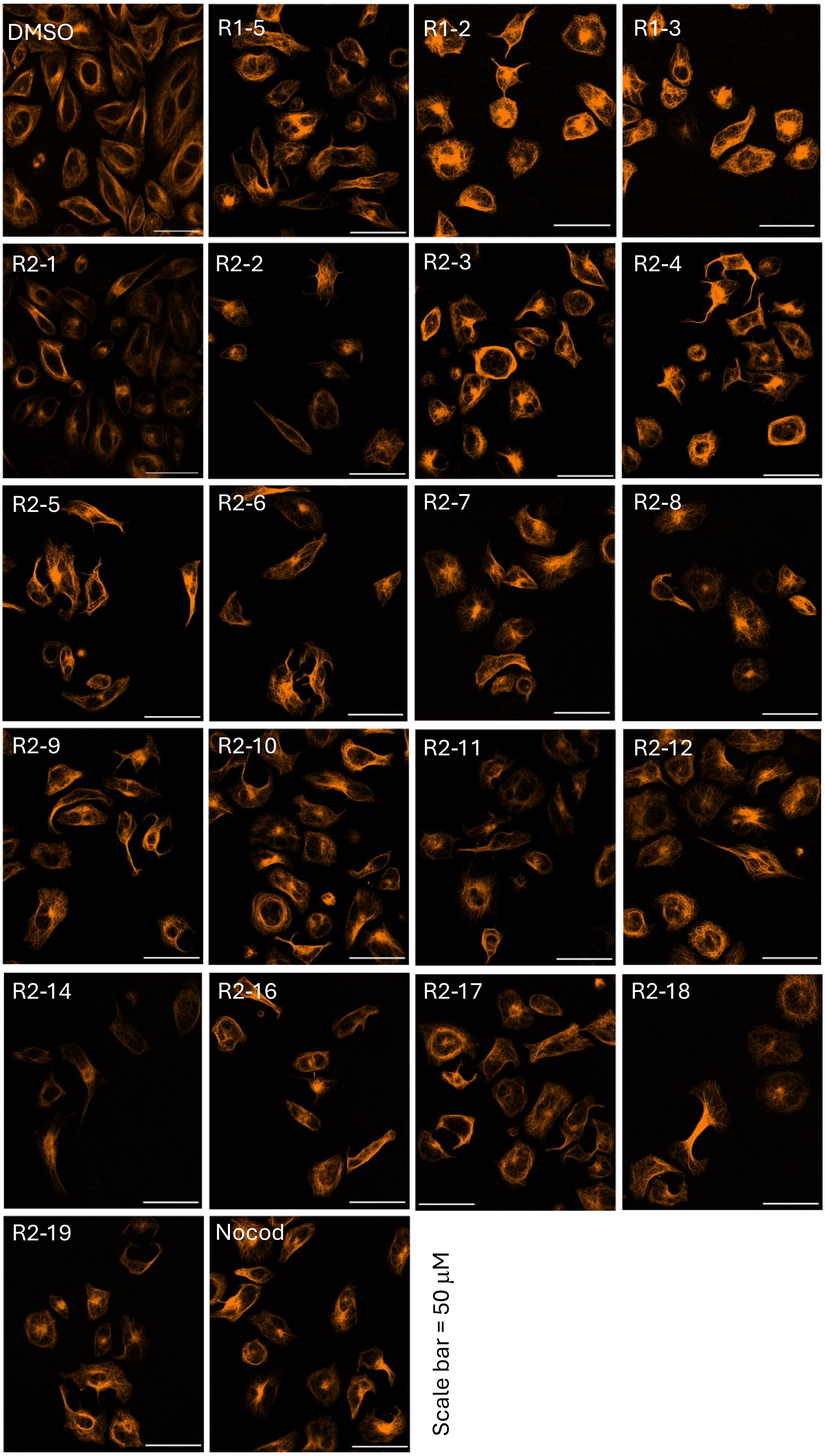

Supplementary Figure 14 Fluorescence microscopy of MT responses on HeLa cells upon treatment with our full set of tubulin hit compounds (750 nM x 24h)

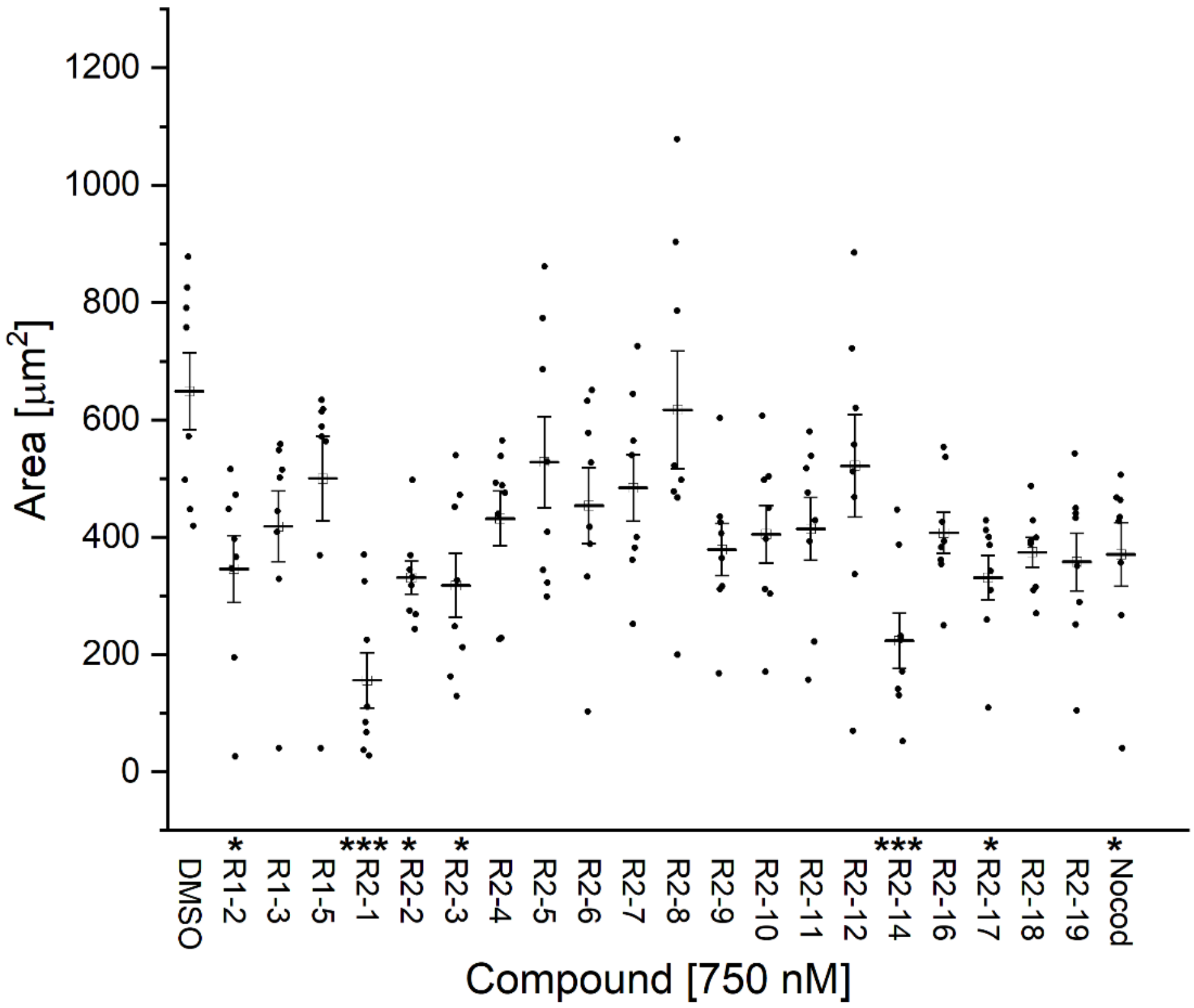

Supplementary Figure 15 Comparison of area measurements for tubulin hit compounds identified in this study. Dots represent median values obtained for each field analysed (n = 4) in duplicate wells. The scatter plot shows the combined median values (n = 8), while boxes depict mean values per well ± standard error. Statistical significance was assessed using a one-way ANOVA followed by Tukey's post hoc test, with significance levels indicated by asterisks (*$P < 0.05$, **$P < 0.01$, ***$P < 0.001$). All compounds were tested at an equal concentration, and the DMSO control was maintained at 0.05% (v/v).

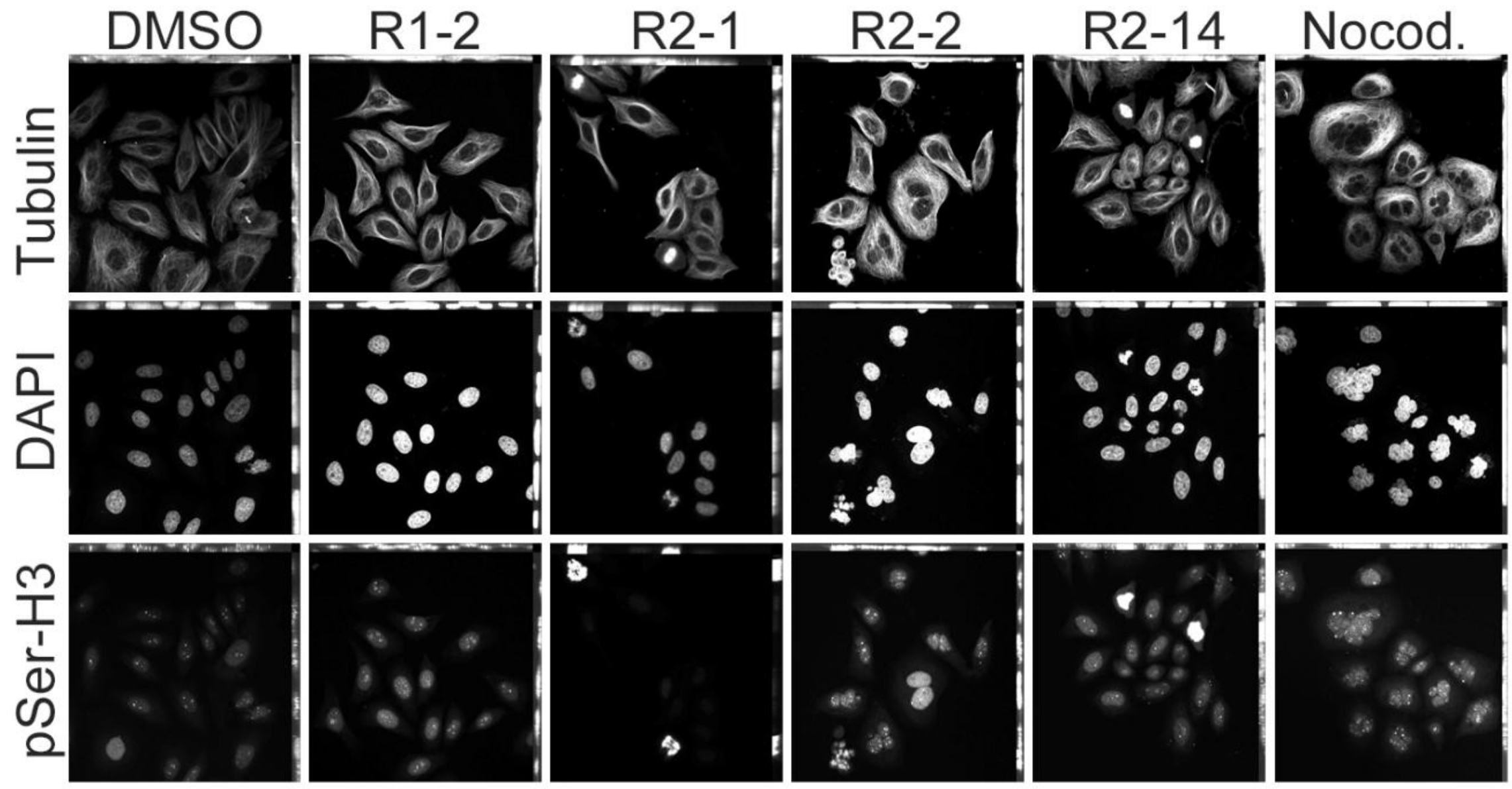

Supplementary Figure 16 Immunofluorescence microscopy images from HeLa cells treated with the tubulin hit compounds. Z-stack sections were processed using the maximum intensity projection option after imaging under the FITC (Tubulin), UV (DAPI) and AF555 (pSer-H3) detectors. Channel images were split as indicated and the top and right borders show the orthogonal views. The merged projection and scale bars are the same as presented in Figure 6C.

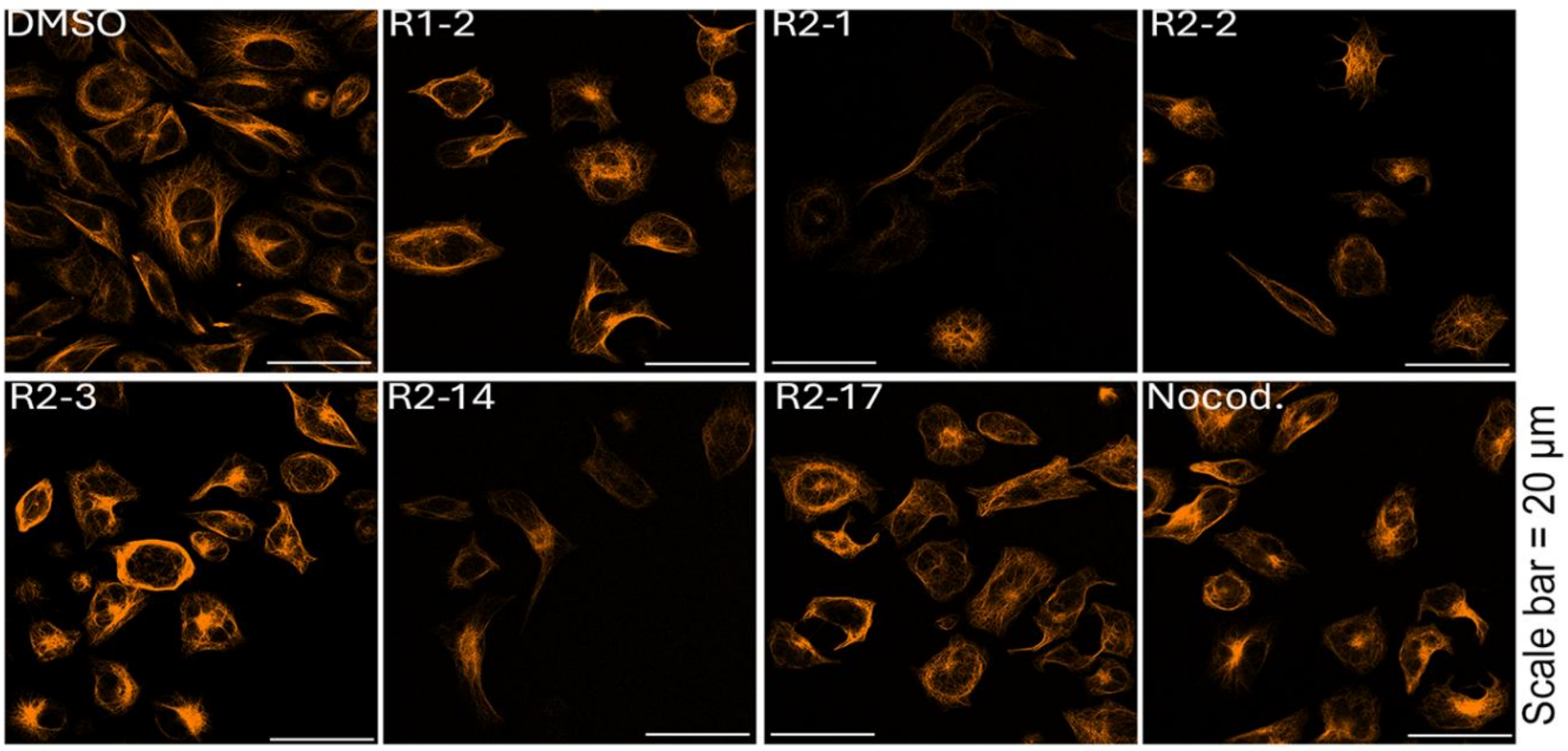

Supplementary Figure 17 Fluorescence microscopy of MT responses on HeLa cells upon treatment with specific compounds (150 nM x 24h)

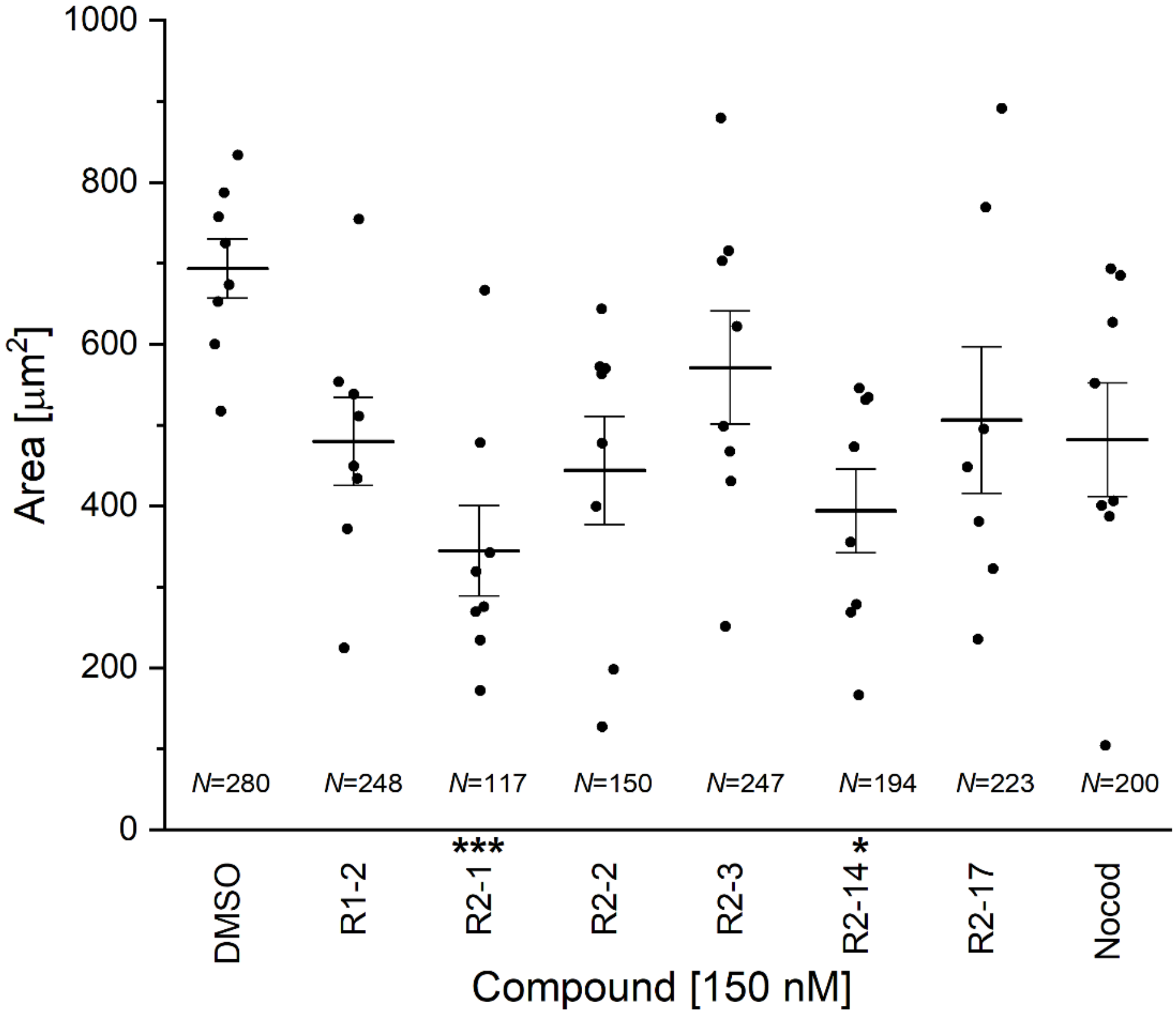

Supplementary Figure 18 Comparison of area measurements for specific compounds identified in this study. Dots represent median values obtained for each field analysed (n = 4) in duplicate wells. The scatter plot shows the combined median values (n = 8), while boxes depict mean values per well ± standard error. Statistical significance was assessed using a one-way ANOVA followed by Tukey's post hoc test, with significance levels indicated by asterisks (*P < 0.05, **P < 0.01, ***P < 0.001). All compounds were tested at an equal concentration, and the DMSO control was maintained at 0.05% (v/v).

8. Notes on Controlling Reproducilibility

To ensure reproducibility across the closed-loop workflow, we implemented routine controls at each step where variance could be introduced, including reaction-yield estimation, purification and weighing:.

- ELSD-based yield estimation was supported by a calibration curve generated from a mixture of standards at multiple concentrations (see Section 2.1). The ELSD response was verified at the beginning of each experimental week using the same standards, monitoring for deviations greater than ±10% in absorbance at known concentrations, which would prompt recalibration.
- Although the yields reported in the manuscript for the Round 1 array correspond to a single execution of the reaction array, this array was repeated to assess consistency. We confirmed that all reactions selected for preparative purification delivered yields above the 15% threshold reproducibly. No significant deviation was observed.

- Losses during preparative HPLC purification were quantified at 15-20% by injecting known amounts of the ELSD standards (as used for the calibration in Section 2.1) and comparing recovered mass to the injected mass; this range is consistent with expectations for automated purification of chemically diverse materials
- During method development, we also observed and additional 10-15% mass loss during Genevac concentration for certain compounds. These losses were eliminated by optimising and adopting a milder evaporation protocol (Section C.3 – Supplementary Table 6), after which no further mass loss was detected, although in some cases it required a second resubjection to the evaporation conditions to remove any remaining solvent
- Each isolated product was weighed in triplicate, and the reported amount reflects the average of these three measurements. The standard deviation of these repeated weighings was always less than 0.3 mg.

D. First Discovery Round

1.Summary and Outcomes

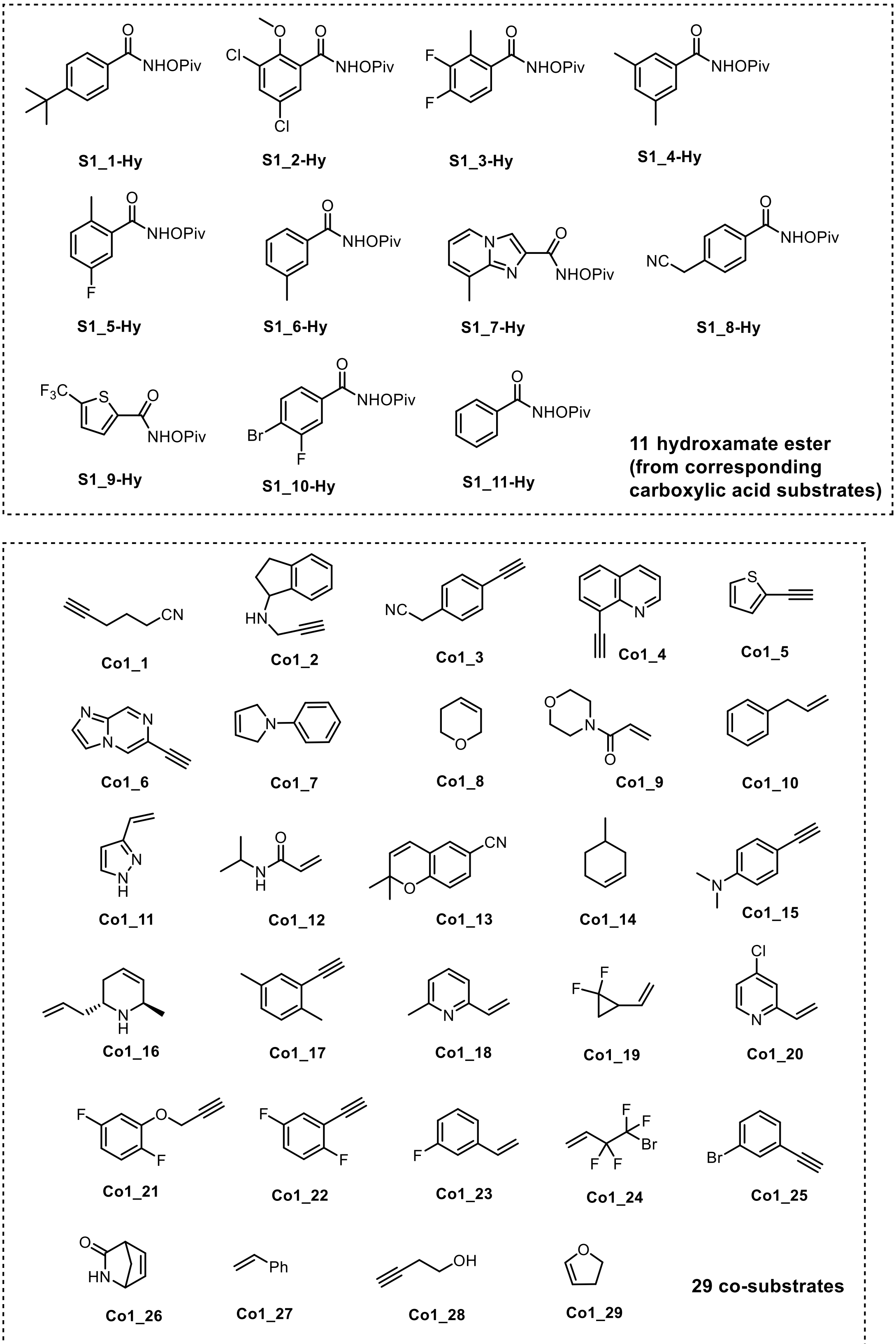

Supplementary Figure 19 Structures of unique substrates and co-substrates selected for the first round of discovery

Supplementary Table 12 First round reactions selected for purification and further evaluation. U = unidentified by ^{1}H NMR.

Synthesis ID	Combination	Target MW	µmol pdt 1	µmol pdt 2	Total isolated yield (%)	Active pdt?	Validated ?
SG-7-41-A1	**S1_1-Hy + Co1_1**	**268**	16.4	-	55		
SG-7-41-A6	**S1_1-Hy + Co1_23**	**297**	17.3	-	58		
SG-7-41-A7	**S1_2-Hy + Co1_1**	**310**	16.5	10.3	89		
SG-7-41-A11	**S1_2-Hy + Co1_9**	**358**	11.2	-	37		
SG-7-41-B3	**S1_2-Hy + Co1_18**	**336**	3.6	-	12		
SG-7-41-B7	**S1_3-Hy + Co1_1**	**262**	5.0	0.4	18		
SG-7-41-B10	**S1_3-Hy + Co1_9**	**310**	12.0	0.3	41		
SG-7-41-C3	**S1_3-Hy + Co1_23**	**291**	5.2	1.4	22		
SG-7-41-C5	**S1_4-Hy + Co1_2**	**318**	5.0	-	17		
SG-7-41-C10	**S1_4-Hy +Co1_24**	**353**	2.3	-	8		
SG-7-41-C11	**S1_5-Hy + Co1_3**	**292**	2.1	-	7	Y, **U, other**	
SG-7-41-D3	**S1_5-Hy + Co1_12**	**447**	7.8	-	26		
SG-7-41-D5	**S1_6-Hy + Co1_10**	**251**	19.9	-	66		
SG-7-41-D6	**S1_6-Hy + Co1_15**	**278**	10.8	-	36		
SG-7-41-D7	**S1_6-Hy + Co1_18**	**252**	19.5	-	65		
SG-7-41-D8	**S1_7-Hy + Co1_1**	**266**	7.1	-	24		
SG-7-41-E10	**S1_8-Hy + Co1_17**	**288**	13.2	-	44	Y, **R1-1, tubulin**	Y
SG-7-41-E11	**S1_8-Hy + Co1_25**	**338**	0.3	-	1		
SG-7-41-E12	**S1_9-Hy + Co1_1**	**286**	13.6	-	45		
SG-7-41-F7	**S1_9-Hy + Co1_9**	**334**	2.1	-	7		
SG-7-41-F12	**S1_9-Hy + Co1_18**	**312**	4.8	-	16		
SG-7-41-G3	**S1_9-Hy + Co1_21**	**361**	8.9	-	30	Y, **R1-3, tubulin**	Y
SG-7-41-G4	**S1_9-Hy + Co1_23**	**315**	8.3	0.6	30		
SG-7-41-G6	**S1_10-Hy + Co1_10**	**333**	8.4	-	28		
SG-7-41-G8	**S1_10-Hy + Co1_17**	**345**	1.5	-	5	Y, **R1-2, tubulin**	Y
SG-7-41-G9	**S1_11-Hy + Co1_26**	**228**	16.2	-	54		
SG-7-41-G10	**S1_11-Hy + Co1_27**	**223**	16.1	-	54	Y, **R1-4, tubulin**	Y
SG-7-41-G11	**S1_11-Hy + Co1_28**	**189**	15.3	0.5	51		
SG-7-41-G12	**S1_11-Hy + Co1_29**	**189**	16.4	-	55		

Supplementary Table 13 Reagents added to the initial combination subset based on hits identified in the first round of discovery.

Reagent	Additional reagent added	Selection Method[a]
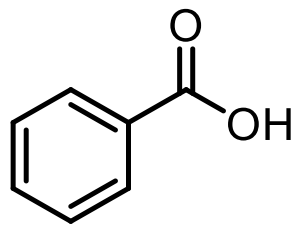	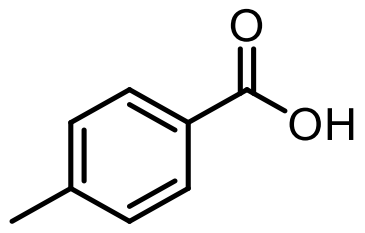	**Same cluster**
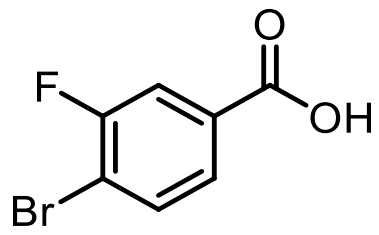	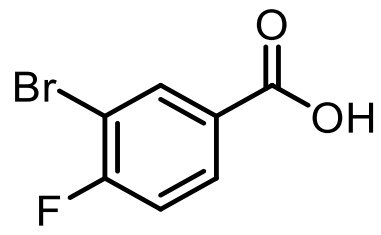	**Same cluster**
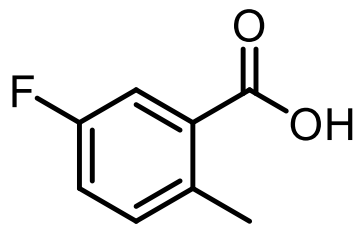	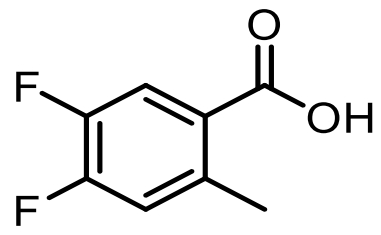	**Same cluster**
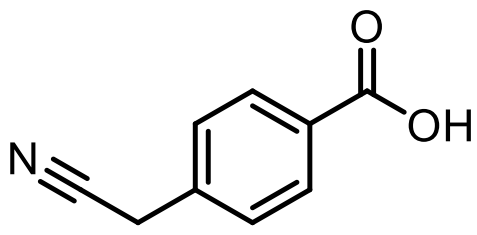	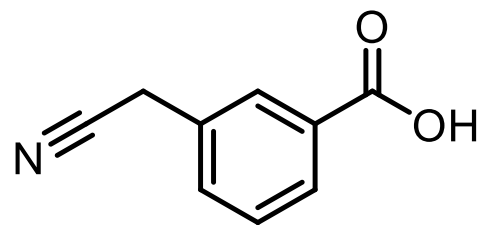	**Same cluster**
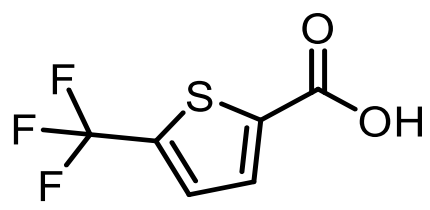	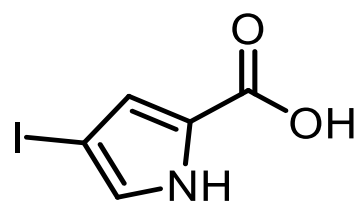	**Nearest Jaccard**
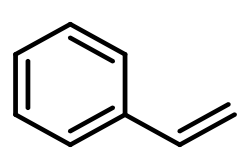	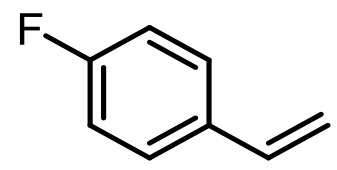	**Same cluster**
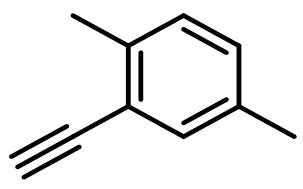	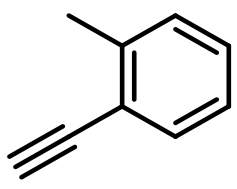	**Same cluster**
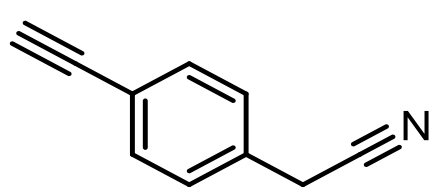	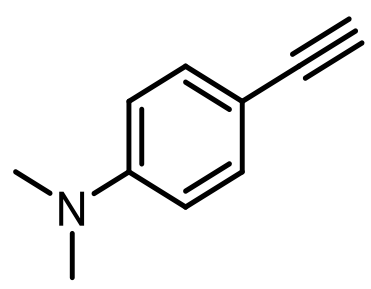	**Nearest Jaccard**
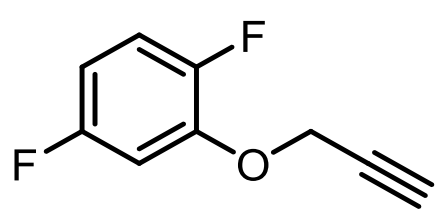	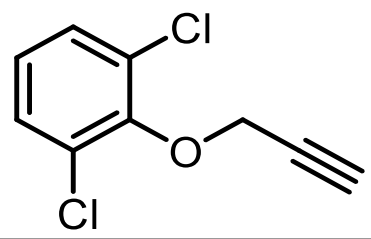	**Nearest Jaccard**

[a] *Reagents were added by selecting either the next most cost-effective compound within each cluster or the nearest neighbour by Jaccard distance in case of singleton clusters. After each addition, all pairwise combinations were recalculated to give the updated subset.*

2. Scoring Strategies

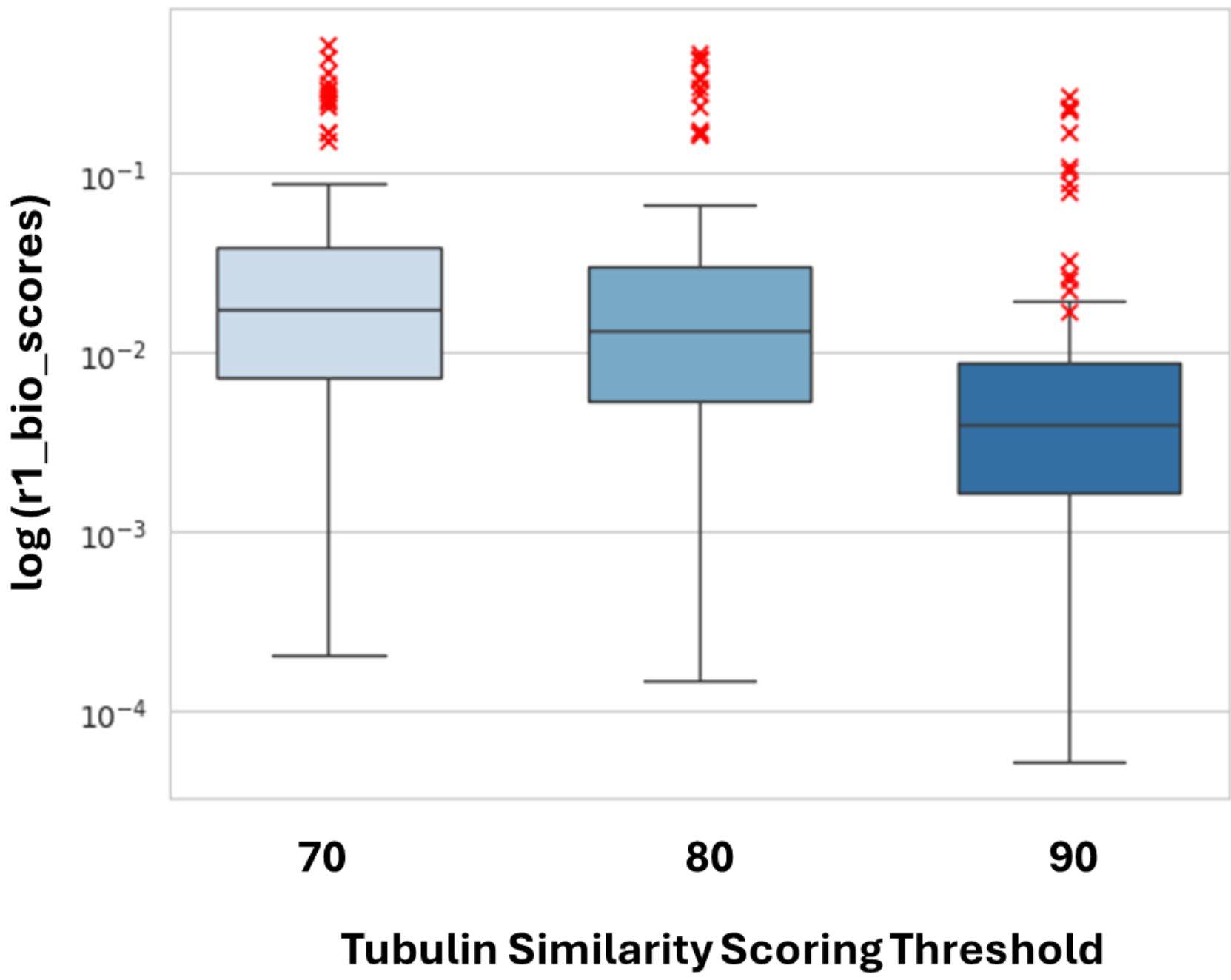

Supplementary Figure 20 Distribution of propagated scores based on round 1 hits with three different tubulin cluster similarity thresholds (70%, 80%, 90%). Boxplots show the distribution of all non-zero scores under each thresholding scheme. Red markers indicate the combinations that were later confirmed as experimental hits in round 2.

A ramp-based scoring scheme was used. For the 70% threshold (see main paper), similarities between 70–80% receive partial scores increasing linearly from 0.1 to 1.0, with full scores assigned at ≥80%. For the 80% threshold the ramp shifts to 80–90%, and for the 90% threshold a single ramp spans 90–100%.

Thresholds of 70% and 80% produce similarly smooth score distributions, with more graded score separation at 70%. In contrast, the 90% threshold yields a more compressed distribution with uniformly lower scores. Notably, the experimentally-confirmed hits fall within the graded continuum at the 70% threshold, where they receive a spread of partial scores, as opposed to the clustering observed with a 80% threshold. With the 90% threshold, these same hits split into two distinct groups – one high-scoring and one low-scoring – such that only the closest structural analogues would have been prioritised at this more stringent cutoff.

E. Subsequent Discovery Round - Summary and Outcomes

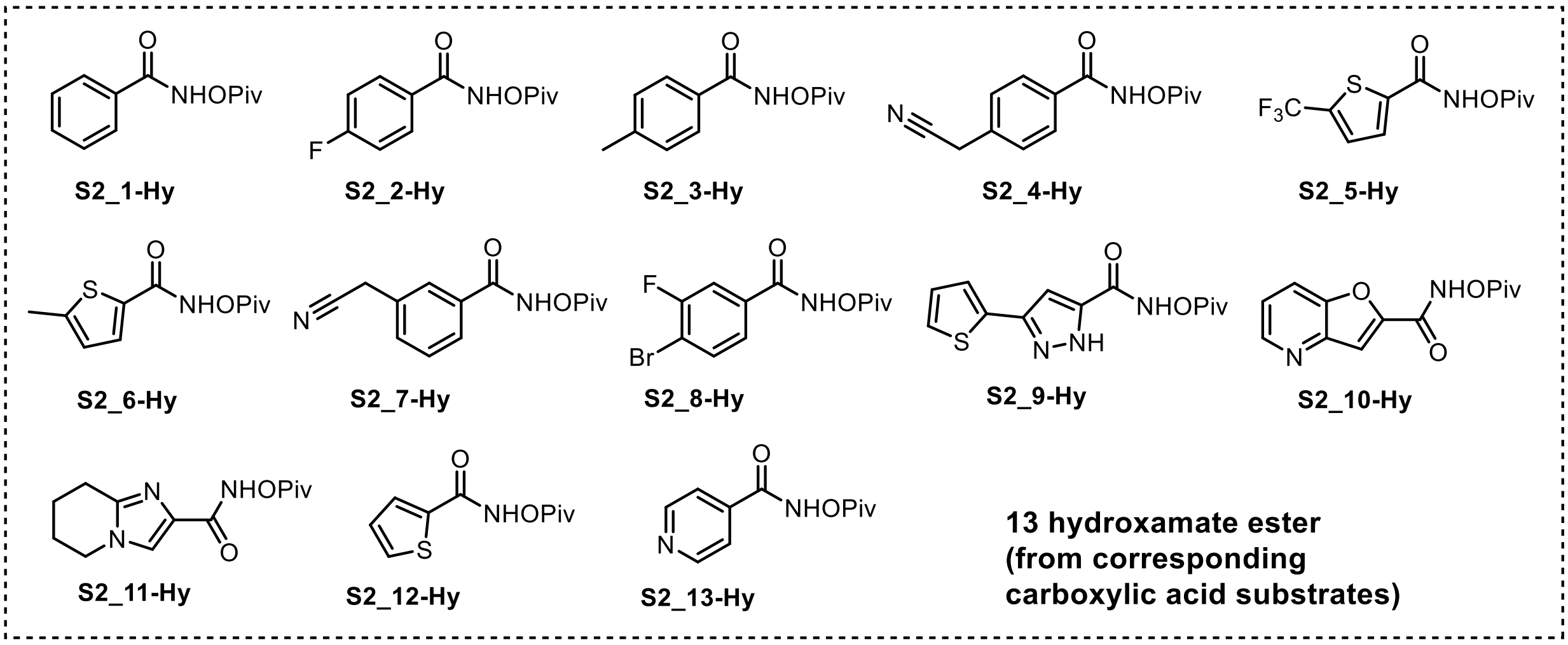

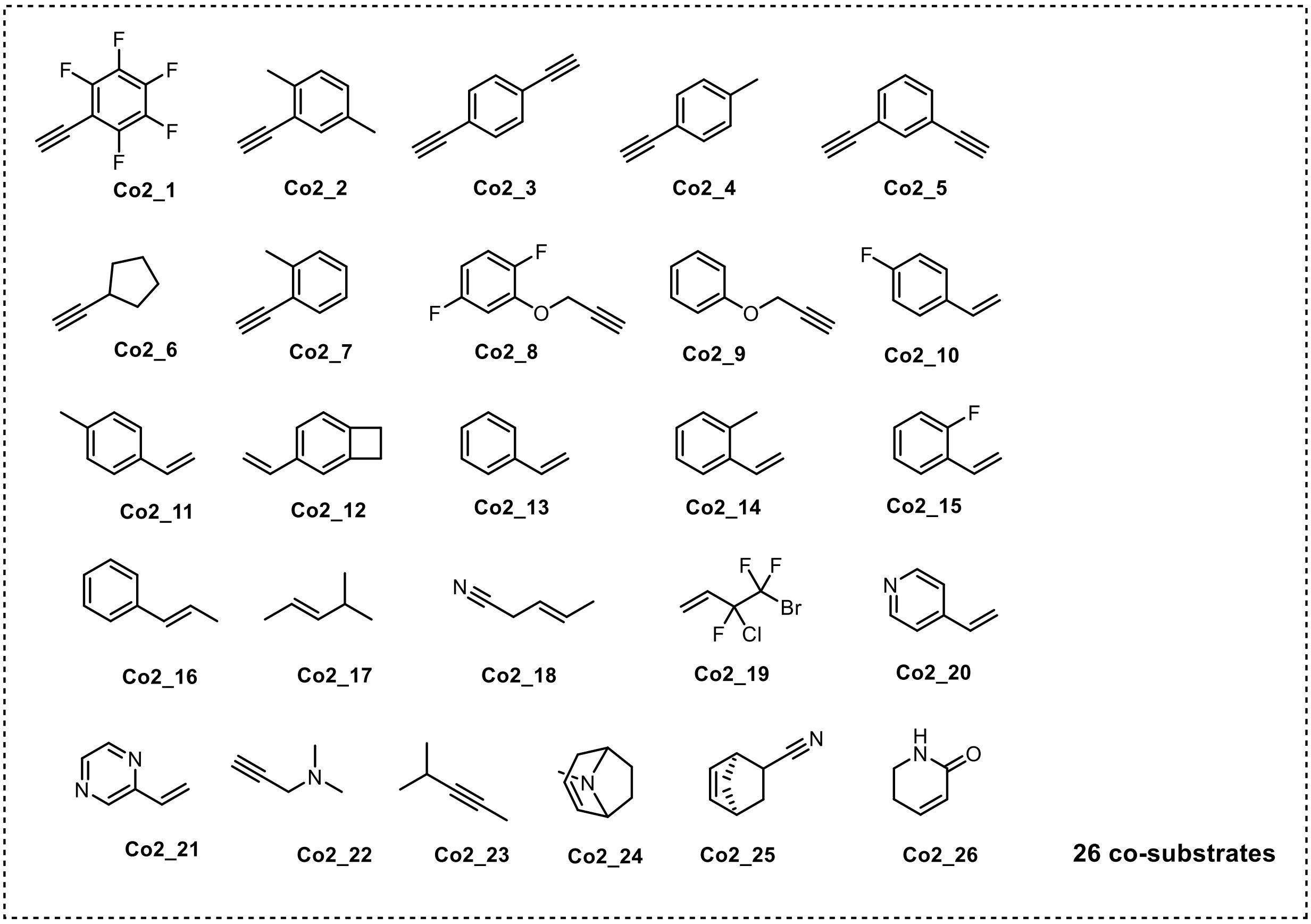

Supplementary Figure 21 Structures of unique substrates and co-substrates selected for the second round of discovery

Supplementary Table 14 Reactions selected for purification and further evaluation in the second discovery round. U = unidentified by ^{1}H NMR.

Synthesis ID	Combination	Target MW 1	Target MW 2	μmol products	Total isolated yield (%)	Active product?	Cluster
RHA3_RP1_A1	**S2_1-Hy + Co2_11**	**238**		[23.9, 5.5]	98		
RHA3_RP1_A3	**S2_1-Hy + Co2_14**	**238**		18.1	60		
RHA3_RP1_A4	**S2_1-Hy + Co2_20**	**225**	**327**	[3.6, 2.1]	19		
RHA3_RP1_A5	**S2_1-Hy + Co2_4**	**236**		3.0	10	Y, **R2-1**	Tubulin
RHA3_RP1_A6	**S2_1-Hy + Co2_12**	**250**		[1.6, 0.8]	8	Y, **R2-2**	Tubulin
RHA3_RP1_A8	**S2_1-Hy + Co2_7**	**250**	**236**	[16.4, 2.5, 2.4]	71	Y, **R2-3**	Tubulin + other
RHA3_RP1_A9	**S2_1-Hy + Co2_5**	**246**		3.3	11	Y, **U**	Tubulin
RHA3_RP1_A11	**S2_2-Hy + Co2_4**	**254**		8.7	29	Y, **R2-4**	Tubulin
RHA3_RP1_A12	**S2_2-Hy + Co2_7**	**254**		12.2	41	Y, **R2-5**	Tubulin
RHA3_RP1_B1	**S2_2-Hy + Co2_15**	**260**		[14.6, 5.8]	68	Y, **R2-6**	Tubulin
RHA3_RP1_B2	**S2_3-Hy + Co2_13**	**238**		[13.4, 7.1]	68	Y, **R2-7**	Tubulin
RHA3_RP1_B3	**S2_3-Hy + Co2_16**	**252**		4.0	13		
RHA3_RP1_B4	**S2_3-Hy + Co2_4**	**281**	**250**	[5.0, 4.4, 3.6, 0.8]	46	Y, **R2-8, R2-9**	Tubulin + other
RHA3_RP1_B6	**S2_3-Hy + Co2_8**	**302**		10.6	35		
RHA3_RP1_B7	**S2_3-Hy + Co2_2**	**266**		1.9	6	Y, **U**	Tubulin + other
RHA3_RP1_B8	**S2_4-Hy + Co2_11**	**277**		[22.0, 1.8]	79		
RHA3_RP1_B9	**S2_4-Hy + Co2_13**	**263**		[12.9, 9.9]	76	Y, **R2-10**	Tubulin
RHA3_RP1_B12	**S2_4-Hy + Co2_4**	**275**	**294**	[3.3, 0.3]	12	Y, **R2-11**	Tubulin
RHA3_RP1_C1	**S2_4-Hy + Co2_9**	**291**	**310**	[0.7, 0.7, 0.7, 0.6]	9		
RHA3_RP1_C2	**S2_4-Hy + Co2_7**	**275**	**294**	[2.2, 1.7, 1.1, 0.7]	19	Y, **R2-12**	Tubulin
RHA3_RP1_C4	**S2_4-Hy + Co2_22**	**242**		5.4	18		
RHA3_RP1_C5	**S2_5-Hy + Co2_13**	**462**		6.7	22	Y, **S2_5**	Other

RHA3_RP1_C6	**S2_5-Hy + Co2_3**	**296**		13.5	45	Y, **S2_5**	Other
RHA3_RP1_C7	**S2_5-Hy + Co2_7**	**310**		14.5	48	Y, **R2-13**	Tubulin
RHA3_RP1_C8	**S2_5-Hy + Co2_5**	**320**		12.8	43	Y, **S2_5**	Other
RHA3_RP1_C9	**S2_5-Hy + Co2_15**	**316**		[16.8, 3.8]	69	Y, **S2_5**	Other
RHA3_RP1_C11	**S2_6-Hy + Co2_11**	**258**		[0.4, 0.4]	3	Y, **R2-14**	Tubulin
RHA3_RP1_C12	**S2_6-Hy + Co2_13**	**244**		[6.1, 0.4]	22	Y, **R2-15**	Tubulin
RHA3_RP1_D2	**S2_7-Hy + Co2_13**	**263**		26.2	75	Y, **R2-16**	Tubulin
RHA3_RP1_D4	**S2_7-Hy + Co2_7**	**275**		3.3	11	Y, **R1-17**	Tubulin
RHA3_RP1_D5	**S2_7-Hy + Co2_8**	**328**		0.3	1		
RHA3_RP1_D6	**S2_7-Hy + Co2_2**	**291**		[1.0, 0.7]	6	Y, **R2-18**	Tubulin
RHA3_RP1_D7	**S2_7-Hy + Co2_23**	**242**	**302**	[9.9, 0.7, 0.4, 0.3]	38	Y, **S2_7**	Other
RHA3_RP1_D8	**S2_7-Hy + Co2_25**	**279**		5.4	18		
RHA3_RP1_D9	**S2_7-Hy + Co2_21**	**279**	**261**	[5.4, 4.6, 1.9, 0.4]	41		
RHA3_RP1_D10	**S2_7-Hy + Co2_26**	**279**	**302**	[6.5, 4.3, 0.3, 0.3]	37		
RHA3_RP1_D11	**S2_7-Hy + Co2_6**	**253**		[11.4, 3.6, 2.4, 1.6, 1.2, 0.8, 0.8, 0.8, 0.4]	77	Y, **R2-O1**	Other
RHA3_RP1_E1	**S2_8-Hy + Co2_10**	**340**		[3.5, 0.3]	13	Y, **R2-O2**	Other
RHA3_RP1_E2	**S2_8-Hy + Co2_7**	**334**		1.5	5	Y, **R2-19**	Tubulin
RHA3_RP1_E5	**S2_9-Hy + Co2_26**	**294**		30.0	100	Y, **S2_9**	Other
RHA3_RP1_E8	**S2_10-Hy + Co2_6**	**263**		8.7	29		
RHA3_RP1_E11	**S2_12-Hy + Co2_23**	**228**	**208**	[1.0, 0.9]	6	Y, **S2_12**	Other
RHA3_RP1_E12	**S2_12-Hy + Co2_25**	**245**		[11.8, 1.2]	43		
RHA3_RP1_F1	**S2_4-Hy + Co2_10**	**281**		22.1	74		
RHA3_RP1_F10	**S2_13-Hy + Co2_11**	**223**		14.8	49		

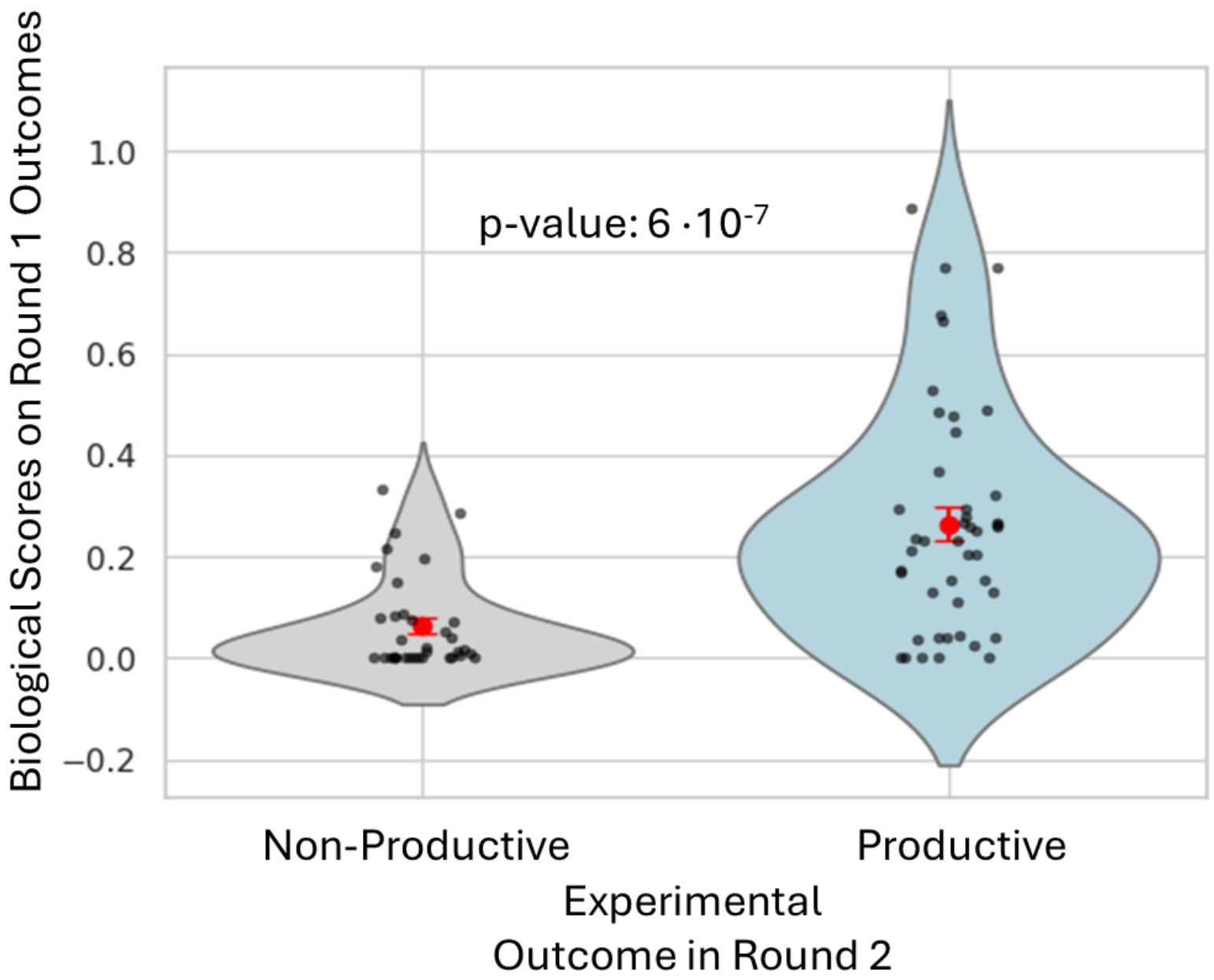

Supplementary Figure 22 Violin plots showing the distribution of biological scores based on similarity to round 1 hits for combinations that corresponded to productive or non-productive reactions in round 2. Each dot represents an individual compound, and red bars indicate the mean ± standard error. The difference between groups was statistically significant (independent two-sample t-test, $p = 6 \times 10^{-7}$).

F. Synthetic Procedures

O-pivaloylhydroxylamine triflate salt (SI-1)

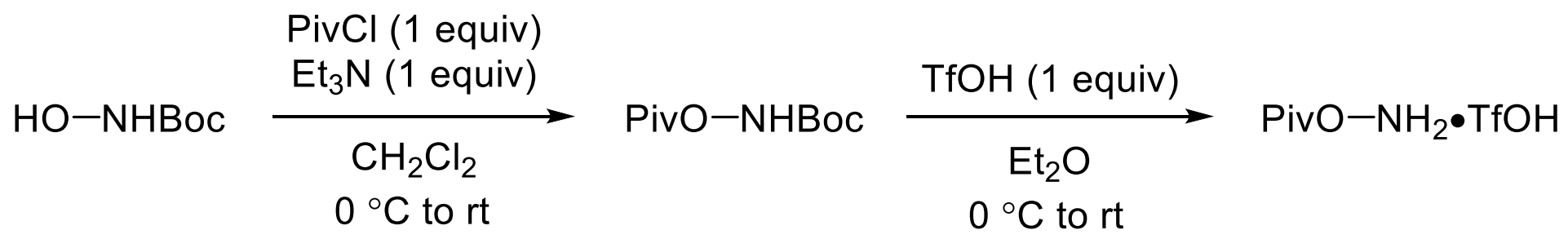

Adapting a procedure from Org. Synth. **2020**, 97, 207–216.

To a solution of N-Boc-hydroxylamine (3.00 g, 22.5 mmol) and Et_3N (3.13 mL, 22.5 mmol) in DCM (40 mL) at 0 °C was added trimethylacetyl chloride (2.80 mL, 22.5 mmol) over 5 mins. The mixture was stirred for 30 mins before warming to room temperature and stirring for a further 2 h. The solids were removed by filtration, and the filtrate was washed with water (50 mL), sat. aq. $NaHCO_3$ (50 mL) and brine (50 mL), dried (Na_2SO_4), filtered and concentrated in vacuo to give PivO–NHBoc as a colourless solid (4.34 g, 89%). ^{1}H NMR (400 MHz, CD_3OD) δ 1.47 (s, 9 H, Boc CH_3), 1.27 (s, 9 H, Piv CH_3); ^{13}C NMR (126 MHz, $CDCl_3$) δ 178.0 (Piv C=O), 155.8 (amide C=O), 83.2 (Boc C), 38.3 (Piv C), 28.2 (Boc CH_3), 27.1 (Piv CH_3);

To a solution of PivO-NHBoc (3.00 g, 13.8 mmol) in Et_2O (30 mL) at 0 °C was added trifluoromethanesulfonic acid (1.22 mL, 13.8 mmol) over 5 mins. The mixture was stirred for 30 mins before warming to room temperature and stirring for a further 3 h. Petroleum ether (40:60, 20 mL) was added, and the mixture was filtered. The solids were washed with petroleum ether (40:60, 3 x 10 mL) and ice-cooled CH_2Cl_2 (3 x 5 mL), giving PivO–NH_2•TfOH as a colourless solid (2.71 g, 74%). ^{1}H NMR (400 MHz, CD_3OD) δ 1.33 (s, 9 H, Piv CH_3); ^{13}C NMR (126 MHz, CD_3OD) δ 175.8 (Piv C=O), 121.8 (q, J_{CF} = 318.4 Hz, CF_3), 39.5 (Piv C), 27.0 (Piv CH_3);

1. Synthesis of hydroxamate esters from carboxylic acids

General Procedure A

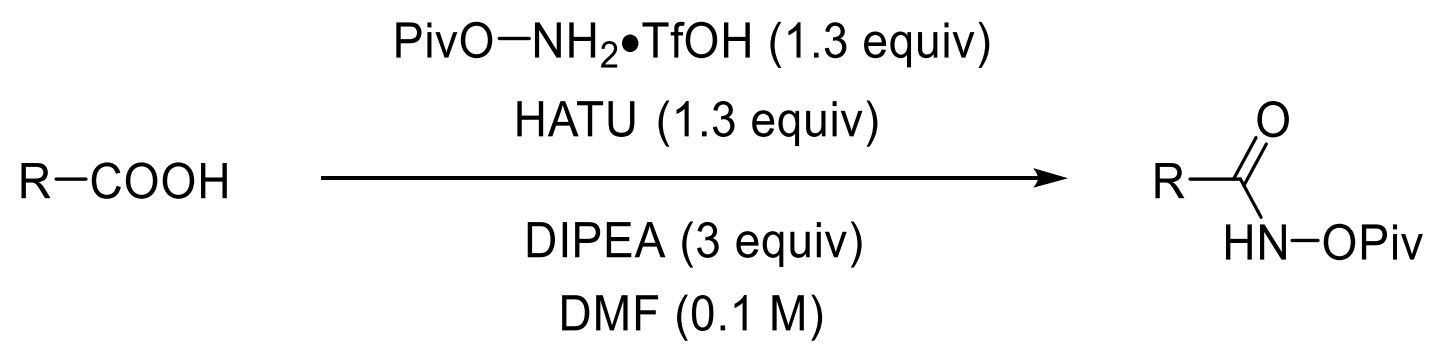

To a solution of *O*-Pivaloyl hydroxylamine triflate salt (1.3 equiv) in DMF (1 mL) was added DIPEA (1.0 equiv). The mixture was stirred for 1 h at room temperature. Separately, DIPEA (2.0 equiv) was added to a solution of carboxylic acid (1.0 equiv) and HATU (1.3 equiv) in DMF (7.5 mL) at room temperature. The *O*-Pivaloyl hydroxylamine solution was added dropwise, and the mixture was stirred for 2 h at room temperature. The mixture was diluted with EtOAc (30 mL), and the organics were washed with water (30 mL), sat. aq. LiCl (3 x 30 mL) and sat. aq. NaCl (30 mL), dried ($MgSO_4$), then filtered and concentrated *in vacuo*. The crude residue was purified by column chromatography under the specified conditions to give the corresponding hydroxamate ester.

Note*: For water-soluble substrates, the aqueous wash was avoided, with the DMF removed under reduced pressure or under a stream of N_2 overnight, followed by purification of the resulting crude residue.*

4-(tert-Butyl)-N-(pivaloyloxy)benzamide (S1_1-Hy)

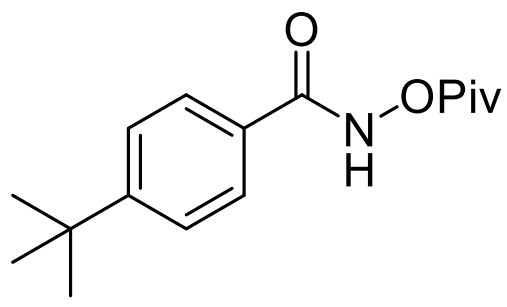

Following **General Procedure A** using 4-*tert*-butylbenzoic acid (100 mg, 0.56 mmol). The crude material was purified by column chromatography (10% EtOAc in hexane) to give hydroxamate ester **S-2** as a colourless oil that solidified on standing (104 mg, 67%). ^{1}H NMR (500 MHz, CD_3OD) δ 7.80 (m, 2 H, 2-H and 6-H), 7.55–7.49 (m, 2 H, 3-H and 5-H), 1.35 (s, 9 H, Piv CH_3), 1.34 (s, 9 H, Ar-*t*Bu CH_3); ^{13}C NMR (126 MHz, CD_3OD) δ 177.5 (Piv C=O), 167.9 (amide C=O), 157.2 (C-4), 129.6 (C-1), 128.4 (C-2 and C-6), 126.7 (C-3 and C-5), 39.4 (Piv C), 35.9 (Ar-*t*Bu C), 31.5 (Piv CH_3), 27.5 (Ar-*t*Bu CH_3); HRMS (ESI) m/z Calculated for $C_{16}H_{23}NO_3Na$ $[M+Na]^+$ 300.1570; Found 300.1573.

3,5-Dichloro-2-methoxy-N-(pivaloyloxy)benzamide (S1_2-Hy)

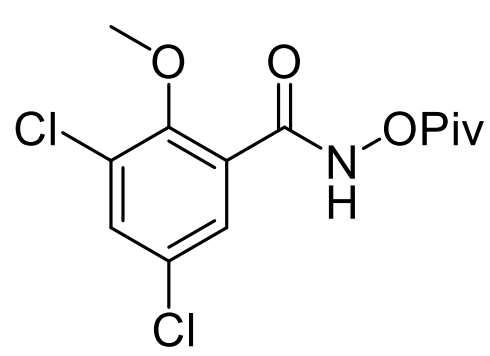

Following **General Procedure A** with 3,5-dichloro-2-methoxybenzoic acid (247 mg, 1.12 mmol). The crude material was purified by column chromatography (10% EtOAc in hexane) to give hydroxamate ester **S-3** as a colourless oil (280 mg, 78%). ^{1}H NMR (500 MHz, CD_3OD) δ 7.68 (d, *J* = 2.6 Hz, 1 H, 6-H), 7.61 (d, *J* = 2.6 Hz, 1 H, 4-H), 3.94 (s, 3 H, OCH_3), 1.35 (s, 9 H, Piv CH_3); ^{13}C NMR (126 MHz, CD_3OD) δ 175.8 (Piv C=O), 162.4 (amide C=O), 153.1 (C-2), 132.7 (C-4), 129.5 (C-3), 129.4 (C-5), 128.8 (C-1), 128.3 (C-6), 61.7 (OCH_3), 38.0 (Piv C), 26.1 (Piv CH_3); HRMS (ESI) m/z Calculated for $C_{13}H_{15}Cl_2NO_4Na$ $[M+Na]^+$ 342.0270; Found 342.0268.

3,4-Difluoro-2-methyl-N-(pivaloyloxy)benzamide (S1_3-Hy)

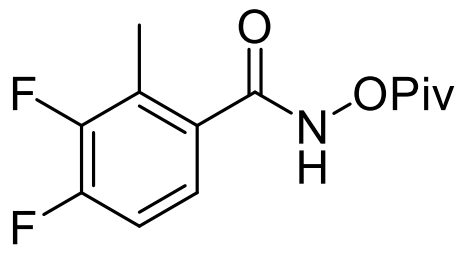

Following **General Procedure A** with 3,4-difluoro-2-methylbenzoic acid (97 mg, 0.56 mmol). The crude material was purified by column chromatography (20% EtOAc in hexane) to give hydroxamate ester **S-4** as a colourless solid (100 mg, 66%).^{1}H NMR (500 MHz, CD_3OD) δ 7.36–7.25 (m, 1 H, 6-H), 7.24–7.13 (m, 1 H, 5-H), 2.40 (d, *J* = 2.4 Hz, 3 H, CH_3), 1.35 (s, 9 H, Piv CH_3); ^{13}C NMR (126 MHz, CD_3OD) δ 175.9 (Piv C=O), 167.5 (amide C=O), 153.1 (dd, J_{CF} = 250.8, 13.8 Hz, C-4), 150.5 (dd, J_{CF} = 245.8, 12.8 Hz, C-3), 131.6 (t, J_{CF} = 3.0 Hz, C-1), 128.4 (d, J_{CF} = 14.8 Hz, C-2), 125.1 (dd, J_{CF} = 7.6, 4.6 Hz, C-6), 115.6 (d, J_{CF} = 18.0 Hz, C-5), 39.4 (Piv C), 27.4 (Piv CH_3), 11.6 (dd, J_{CF} = 4.8, 2.3 Hz, CH_3); ^{19}F NMR (471 MHz, CD_3OD) δ –136.7- –136.9 (m), –142.4 - –142.6 (br d, *J* = 18.8 Hz); HRMS (ESI) m/z Calculated for $C_{13}H_{15}F_2NO_3Na$ $[M+Na]^+$ 294.0912; Found 294.0913.

3,5-Dimethyl-N-(pivaloyloxy)benzamide (S1_4-Hy)

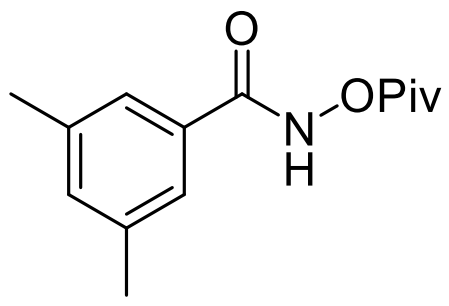

Following **General Procedure A** with 3,5-dimethylbenzoic acid (84 mg, 0.56 mmol). The crude material was purified by column chromatography (20% EtOAc in hexane) to give hydroxamate ester **S-5** as a colourless solid (99 mg, 71%). ^{1}H NMR (500 MHz, CD_3OD) δ 7.42 (br s, 2 H, 2-H and 6-H), 7.24 (s, 1 H, 4-H), 2.36 (br s, 6 H, 2 × CH_3), 1.34 (s, 9 H, Piv CH_3); ^{13}C NMR (126 MHz, CD_3OD) δ 177.6 (Piv C=O), 168.3 (amide C=O), 139.7

(C-4), 134.9 (C-3 and C-5), 132.5 (C-1), 126.2 (C-2 and C-6), 39.4 (Piv C), 27.5 (Piv CH_3), 21.2 (2 × CH_3); HRMS (ESI) m/z Calculated for $C_{14}H_{19}NO_3Na$ [M+Na]$^+$ 272.1257; Found 272.1255.

5-Fluoro-2-methyl-N-(pivaloyloxy)benzamide (S1_5-Hy)

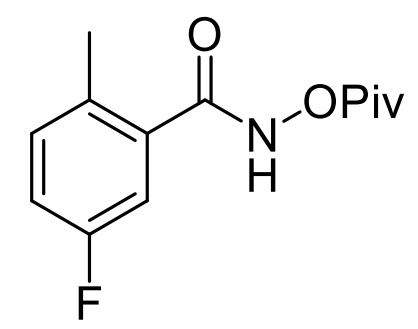

Following **General Procedure A** with 5-fluoro-2-methylbenzoic acid (87 mg, 0.56 mmol). The crude material was purified by column chromatography (10% EtOAc in hexane) to give hydroxamate ester **S-6** as a colourless solid (102 mg, 72%). ^{1}H NMR (500 MHz, CD_3OD) δ 7.31 (dd, *J* = 8.4, 5.4 Hz, 1 H, 6-H), 7.23–7.18 (m, 1 H, 3-H), 7.15 (dd, *J* = 8.4, 2.7 Hz, 1 H, 4-H), 2.41 (s, 3 H, CH_3), 1.35 (s, 9 H, Piv CH_3); ^{13}C NMR (126 MHz, CD_3OD) δ 177.3 (Piv C=O), 168.2 (amide C=O), 161.9 (d, J_{CF} = 244.4 Hz, C-5), 135.3 (d, J_{CF} = 6.6 Hz, C-1), 134.0 (d, J_{CF} = 2.9 Hz, C-2), 133.8 (d, J_{CF} = 7.6 Hz, C-3), 118.4 (d, J_{CF} = 21.0 Hz, C-6), 115.5 (d, J_{CF} = 23.4 Hz, C-4), 39.4 (Piv C), 27.5 (Piv CH_3), 18.8 (CH_3); ^{19}F{^{1}H} NMR (471 MHz, CD_3OD) δ –119.1 (s); HRMS (ESI) m/z Calculated for $C_{13}H_{16}FNO_3Na$ [M+Na]$^+$ 276.1006; Found 276.1006.

3-Methyl-N-(pivaloyloxy)benzamide (S1_6-Hy)

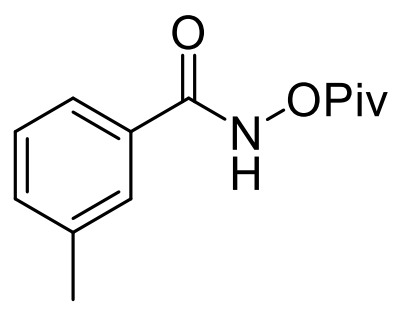

Following **General Procedure A** with 3-methylbenzoic acid (76 mg, 0.56 mmol). The crude material was purified by column chromatography (10% EtOAc in hexane) to give hydroxamate ester **S-7** as a colourless oil that solidified on standing (105 mg, 80%). ^{1}H NMR (500 MHz, CD_3OD) δ 7.64 (s, 1 H, 2-H), 7.60 (d, *J* = 7.6 Hz, 1 H, 6-H), 7.41 (d, *J* = 7.6 Hz, 1 H, 4-H), 7.37 (t, *J* = 7.6 Hz, 1 H, 3-H), 2.41 (s, 3 H, CH_3), 1.35 (s, 9 H, Piv CH_3); ^{13}C NMR (126 MHz, CD_3OD) δ 177.6 (Piv C=O), 168.2 (amide C=O), 139.0 (C-5), 134.2 (C-4), 132.5 (C-1), 129.7 (C-6), 129.0 (C-2), 125.6 (C-3), 39.4 (Piv C), 27.5 (Piv CH_3), 21.3 (CH_3); HRMS (ESI) m/z Calculated for $C_{13}H_{17}FNO_3Na$ [M+Na]$^+$ 258.1101; Found 258.1100.

8-Methyl-N-(pivaloyloxy)imidazo[1,2-a]pyridine-2-carboxamide (S1_7-Hy)

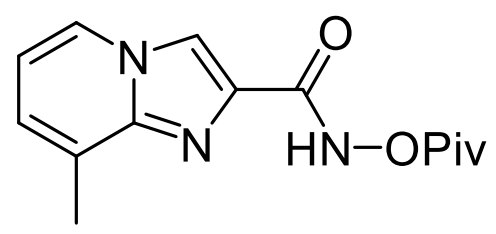

Following **General Procedure A** with 8-methylimidazo[1,2-a]pyridine-2-carboxylic acid (99 mg, 0.56 mmol). The crude material was purified by column chromatography (5% MeOH in CH_2Cl_2) to give hydroxamate ester **S-8** as a colourless solid (79 mg, 51% yield). ^{1}H NMR (500 MHz, CD_3OD) δ 8.30 (s, 1 H, 5-H), 8.28 (d, *J* = 6.8 Hz, 1 H, 3-H), 7.15–7.11 (m, 1 H, 7-H), 6.85 (t, *J* = 6.8 Hz, 1 H, 6-H), 2.55 (s, 3 H, CH_3), 1.36 (s, 9 H, Piv CH_3); ^{13}C NMR (126 MHz, CD_3OD) δ 177.4 (Piv C=O), 163.0 (amide C=O), 146.9 (C-8a), 137.3 (C-8), 129.0 (C-2), 126.6 (C-5), 126.2 (C-7), 117.3 (C-3), 115.0 (C-6), 39.4 (Piv C), 27.5 (Piv CH_3), 17.0 (CH_3); HRMS (ESI) m/z Calculated for $C_{14}H_{18}N_3O_3Na$ [M+Na]$^+$ 276.1343; Found 276.1345.

4-(Cyanomethyl)-N-(pivaloyloxy)benzamide (S1_8-Hy, S2_4-Hy)

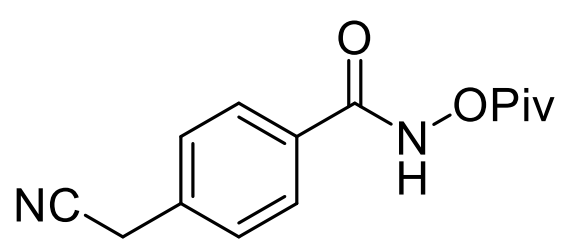

Following **General Procedure A** using 4-(cyanomethyl)benzoic acid (90 mg, 0.56 mmol). The crude material was purified by column chromatography (40% EtOAc in hexane) to give hydroxamate ester **S-9** as a colourless solid (70 mg, 48%). ^{1}H NMR (500 MHz, CD_3OD) δ 7.85 (d, *J* = 8.4 Hz, 2 H, 2-H and 6-H), 7.51 (br d, *J* = 8.4 Hz, 2 H, 3-H and 5-H), 4.01 (s, 2 H, CH_2CN), 1.35 (s, 9 H, Piv CH_3); ^{13}C NMR (126 MHz, CD_3OD) δ 177.5 (Piv C=O), 167.3 (amide

C=O), 137.1 (C-4), 132.2 (C-1), 129.5 (C-2 and C-6), 129.3 (C-3 and C-5), 119.0 (CN), 39.4 (Piv C), 27.5 (Piv CH_3), 23.5 (CH_2); HRMS (ESI) m/z Calculated for $C_{14}H_{16}N_2NaO_3$ [M+Na]+ 283.1053; Found 283.1050..

N-(pivaloyloxy)-5-(trifluoromethyl)thiophene-2-carboxamide (S1_9-Hy and S2_5-Hy)

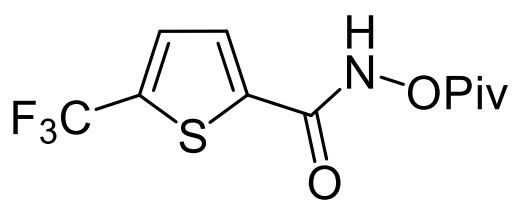

Following **General Procedure A** using 5-(trifluoromethyl)thiophene-2-carboxylic acid (219 mg, 1.12 mmol). The crude material was purified by column chromatography (25% EtOAc in hexane) to give hydroxamate ester **S-10** as a yellow solid (228 mg, 69%). ^{1}H NMR (500 MHz, CD_3OD) δ 7.70–7.66 (m, 1 H, 3-H), 7.62–7.59 (m, 1 H, 4-H), 1.34 (s, 9 H, Piv CH_3); ^{13}C NMR (126 MHz, CD_3OD) δ 177.3 (Piv C=O), 161.0 (amide C=O), 140.4 (C-2), 136.4 (q, J_{CF} = 38.5 Hz, C-5), 130.8 (q, J_{CF} = 3.7 Hz, C-4), 130.0 (C-3), 123.5 (q, J_{CF} = 268.7 Hz, CF_3), 39.4 (Piv C), 27.4 (Piv CH_3); ^{19}F{^{1}H} NMR (376 MHz, CD_3OD) δ –57.6 (s); HRMS (ESI) m/z Calculated for $C_{11}H_{12}F_3NNaO_3S$ [M+Na]+ 318.0382; Found 318.0384.

4-Bromo-3-fluoro-N-(pivaloyloxy)benzamide (S1_10-Hy, S2_8-Hy)

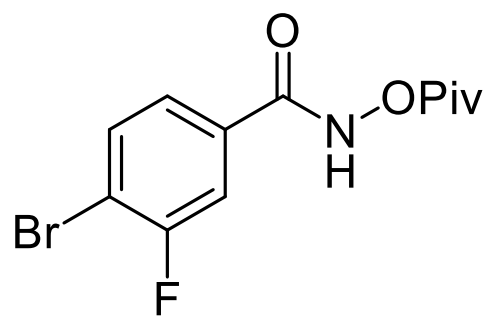

Following **General Procedure A** using 4-bromo-3-fluorobenzoic acid (123 mg, 0.56 mmol). The crude material was purified by column chromatography (15% EtOAc in hexane) to give hydroxamate ester **S-11** as a colourless solid (150 mg, 84%). ^{1}H NMR (500 MHz, CD_3OD) δ 7.77 (dd, *J* = 8.3, 6.9 Hz, 1 H, 5-H), 7.64 (dd, *J* = 9.2, 1.9 Hz, 1 H, 6-H), 7.56 (dd, *J* = 8.3, 1.9 Hz, 1 H, 2-H), 1.34 (s, 9 H, Piv CH_3); ^{13}C NMR (126 MHz, CD_3OD) δ 177.4 (Piv C=O), 165.5 (amide C=O), 160.3 (d, J_{CF} = 247.7 Hz, C-3), 135.4 (C-1), 134.1 (d, J_{CF} = 6.4 Hz, C-5), 125.6 (d, J_{CF} = 3.8 Hz, C-6), 116.5 (d, J_{CF} = 24.5 Hz, C-2), 114.4 (d, J_{CF} = 22.0 Hz, C-4), 39.4 (Piv C), 27.4 (Piv CH_3); ^{19}F NMR (376 MHz, CD_3OD) δ –107.8 (t); HRMS (ESI) m/z Calculated for $C_{11}H_{12}BrFNO_3Na$ [M+Na]+ 339.9955 and 341.9935; Found 339.9944 and 341.9940.

N-(pivaloyloxy)benzamide (S1_11-Hy, S2_1-Hy)

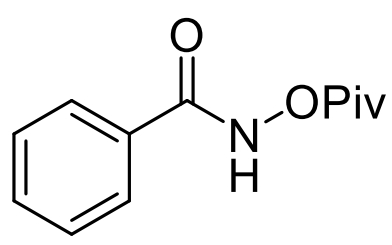

To a solution of PivO–NH_2•TfOH (1.00 g, 3.75 mmol) in THF (17 mL) at 0 °C was added Et_3N (949 μL, 6.82 mmol) and benzoyl chloride (396 μL, 3.41 mmol). The mixture was stirred for 2 h, then concentrated in vacuo. The residue was re-dissolved in EtOAc (40 mL) and organics were washed with sat. aq. $NaHCO_3$ (40 mL) and brine (40 mL), dried ($MgSO_4$), filtered and concentrated in vacuo to give hydroxamate ester **S-1** as a colourless solid (648 mg, 86%). ^{1}H NMR (500 MHz, $CDCl_3$) δ 9.40 (s, 1 H, NH), 7.84–7.79 (m, 2 H, 2-H and 6-H), 7.59–7.53 (m, 1 H, 4-H), 7.49–7.43 (m, 2 H, 3-H and 5-H), 1.36 (s, 9 H, Piv CH_3); ^{13}C NMR (126 MHz, $CDCl_3$) δ 177.2 (Piv C=O), 166.9 (amide C=O), 132.9 (C-4), 131.0 (C-1), 129.0 (C-2 and C-6), 127.6 (C-3 and C-5), 38.6 (Piv C), 27.2 (Piv CH_3); HRMS (ESI) m/z Calculated for $C_{12}H_{16}NO_3$ [M+H]+ 222.1125; Found 222.1125.

4-Fluoro-N-(pivaloyloxy)benzamide (S2_2-Hy)

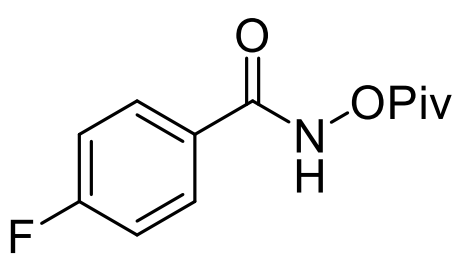

Following **General Procedure A** using 4-fluorobenzoic acid (280 mg, 2.00 mmol). The crude material was purified by column chromatography (20% EtOAc in hexane) to give hydroxamate ester **S-12** as a pale yellow solid (187 mg, 39%). ^{1}H NMR (400 MHz, CD_3OD) δ 7.95–7.86 (m, 2 H, 2-H and 6-H), 7.30–7.20 (m, 2 H, 3-H and 5-H), 1.37 (s, 9 H, Piv CH_3); ^{13}C NMR (101 MHz, CD_3OD) δ 176.2 (Piv C=O), 165.5 (amide C=O), 165.3 (d, J_{CF} = 247.7 Hz, C-4), 129.8

(d, J_{CF} = 9.0 Hz, C-2 and C-6), 127.6 (d, J_{CF} = 3.2 Hz, C-1), 115.4 (d, J_{CF} = 22.3 Hz, C-3 and C-5), 38.0 (Piv C), 26.1 (Piv CH_3); $^{19}F\{^1H\}$ NMR (376 MHz, CD_3OD) δ –109.04 (s); HRMS (ESI) m/z Calculated for $C_{11}H_{14}FNO_3Na$ [M+Na]$^+$ 262.0855; Found 262.0853.

4-Methyl-N-(pivaloyloxy)benzamide (S2_3-Hy)

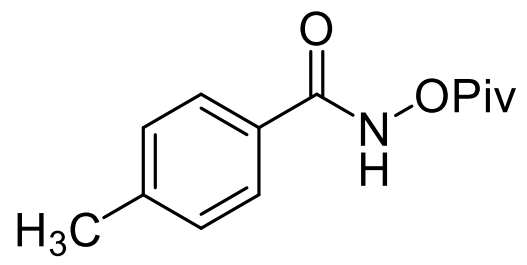

Following **General Procedure A** using 4-methylbenzoic acid (272 mg, 2.00 mmol). The crude material was purified by column chromatography (20% EtOAc in hexane) to give hydroxamate ester **S-13** as a white solid (131 mg, 25%). ^{1}H NMR (400 MHz, CD_3OD) δ 7.75–7.71 (m, 2 H, 2-H and 6-H), 7.35–7.29 (m, 2 H, 3-H and 5-H), 2.42 (s, 3 H, CH_3), 1.36 (s, 9 H, Piv CH_3); ^{13}C NMR (101 MHz, CD_3OD) δ 176.2 (Piv C=O), 166.7 (amide C=O), 143.0 (C-4), 129.0 (C-3 and C-5), 128.3 (C-1), 127.2 (C-2 and C-6), 38.0 (Piv C), 26.1 (Piv CH_3), 20.2 (CH_3); HRMS (ESI) m/z Calculated for $C_{13}H_{18}NO_3$ [M+H]$^+$ 236.1281; Found 236.1280.

5-Methyl-N-(pivaloyloxy)thiophene-2-carboxamide (S2_6-Hy)

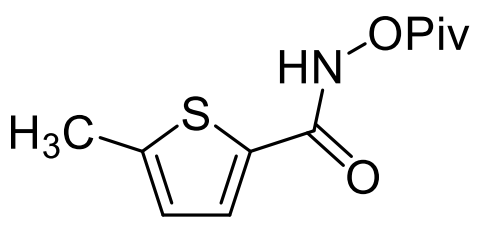

Following **General Procedure A** using 5-methylthiophene-2-carboxylic acid (284 mg, 2.00 mmol). The crude material was purified by column chromatography (20% EtOAc in hexane) to give hydroxamate ester **S-14** as a white solid (210 mg, 43%). ^{1}H NMR (400 MHz, $CDCl_3$) δ 9.17 (br s, 1 H, NH), 7.49 (d, J = 3.7 Hz, 1 H, 3-H), 6.84–6.75 (m, 1 H, 4-H), 2.54 (s, 3 H, CH_3), 1.37 (s, 9 H, Piv CH_3); ^{13}C NMR (101 MHz, $CDCl_3$) δ 177.2 (Piv C=O), 162.1 (amide C=O), 147.4 (C-5), 131.0 (C-2), 130.9 (C-3), 126.4 (C-4), 38.4 (Piv C), 27.0 (Piv CH_3), 15.7 (aryl CH_3); HRMS (ESI) m/z Calculated for $C_{11}H_{15}NO_3SNa$ [M+Na]$^+$ 264.0665; Found 264.0663.

[3-(Cyanomethyl)phenyl]formamido 2,2-dimethylpropanoate (S2_7-Hy)

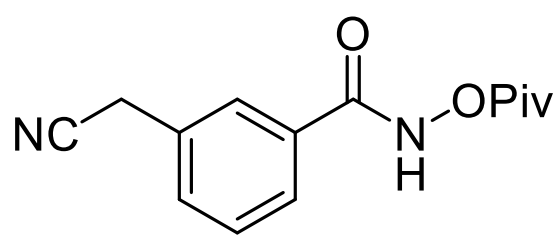

Following **General Procedure A** using 3-(cyanomethyl)benzoic acid (322 mg, 2.00 mmol). The crude material was purified by column chromatography (20% EtOAc in hexane) to give hydroxamate ester **S-15** as a white solid (166 mg, 32%). ^{1}H NMR (400 MHz, CD_3OD) δ 7.85 (d, J = 1.9 Hz, 1 H, 2-H), 7.80 (dt, J = 7.7, 1.6 Hz, 1 H, 6-H), 7.63 (ddt, J = 7.7, 1.9, 1.0 Hz, 1 H, 5-H), 7.55 (t, J = 7.7 Hz, 1 H, 4-H), 4.02 (s, 2 H, CH_2CN), 1.37 (s, 9 H, Piv CH_3); ^{13}C NMR (101 MHz, $CDCl_3$) δ 176.1 (Piv C=O), 165.9 (amide C=O), 132.2 (C-3), 132.0 (C-1), 131.6 (C-4), 129.2 (C-6), 126.9 (C-2), 126.5 (C-5), 117.8 (CN), 38.0 (Piv C), 26.1 (Piv CH_3), 22.0 (CH_2); HRMS (ESI) m/z Calculated for $C_{14}H_{16}N_2O_3Na$ [M+Na]$^+$ 283.1053; Found 283.1055.

N-(pivaloyloxy)-5-(thiophen-2-yl)-1H-pyrazole-3-carboxamide (S2_9-Hy)

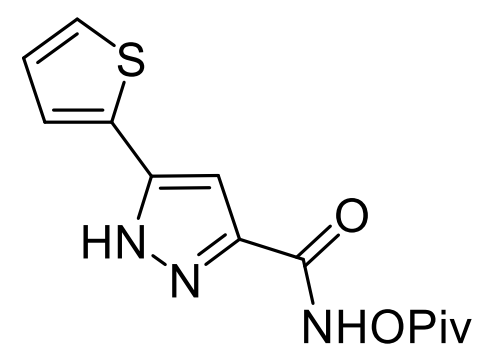

Following **General Procedure A** using 5-(thiophen-2-yl)-1H-pyrazole-3-carboxylic acid (194 mg, 1.00 mmol). The crude material was purified by column chromatography (20% EtOAc in hexane) to give hydroxamate ester **S-16** as an off-white solid (132 mg, 45%). ^{1}H NMR (400 MHz, $CDCl_3$) δ 7.37 (dd, J = 5.1, 1.2 Hz, 1 H, thiophenyl 5-H), 7.31 (dd, J = 3.7, 1.2 Hz, 1 H, thiophenyl 3-H), 7.10 (dd, J = 5.1, 3.6 Hz, 1 H, thiophenyl 4-H), 6.98 (s, 1 H, 4-H), 1.39 (s, 9 H, Piv CH_3); ^{13}C NMR (101 MHz, CD_3OD) δ 176.1 (Piv C=O), 161.1 (amide C=O), 144.6 (C-3 pyrazole), 138.7 (C-2 thiophene), 130.4 (C-5 pyrazole),

127.6 (C-3 thiophene), 125.8 (C-5 thiophene), 124.8 (C-4 thiophene), 102.8 (C-4 pyrazole), 38.0 (Piv C), 26.0 (Piv CH_3); HRMS (ESI) m/z Calculated for $C_{13}H_{16}N_3O_3S$ $[M+H]^+$ 294.0907; Found 294.0904.

N-(pivaloyloxy)furo[3,2-b]pyridine-2-carboxamide (S2_10-Hy)

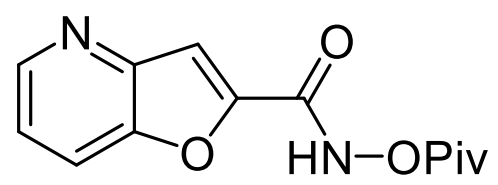

Following **General Procedure A** using furo[3,2-b]pyridine-2-carboxylic acid (250 mg, 1.53 mmol). The crude material was purified by column chromatography (40% EtOAc in hexane) to give hydroxamate ester **S-17** as a white solid (175 mg, 71%). ^{1}H NMR (400 MHz, CD_3OD) δ 8.62 (dt, *J* = 4.8, 1.2 Hz, 1 H, 5-H), 8.11–8.06 (m, 1 H, 7-H), 7.66 (d, *J* = 1.2 Hz, 1 H, 3-H), 7.54 (dd, *J* = 8.4, 4.7, 1.0 Hz, 1 H, 6-H), 1.38 (s, 9 H, Piv CH_3); ^{13}C NMR (101 MHz, CD_3OD) δ 175.7 (Piv C=O), 156.6 (amide C=O), 149.8 (C-2), 148.8 (C-7a), 147.0 (C-5), 145.6 (C-3a), 122.0 (C-6), 120.1 (C-7), 110.8 (C-3), 38.1 (Piv C), 26.1 (Piv CH_3); HRMS (ESI) m/z Calculated for $C_{13}H_{15}N_2O_4$ $[M+H]^+$ 263.1026; Found 263.1023.

N-(pivaloyloxy)-5,6,7,8-tetrahydroimidazo[1,2-a]pyridine-2-carboxamide (S2_11-Hy)

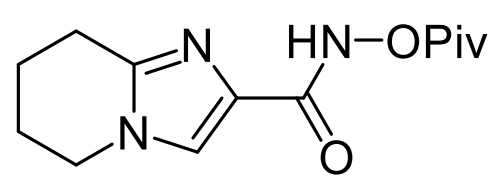

Following **General Procedure A** using 5,6,7,8-tetrahydroimidazo[1,2-a]pyridine-2-carboxylic acid (249 mg, 1.50 mmol). The crude mixture was purified by automated flash column chromatography (reverse-phase CombiFlash on a C18 column with ELS detection) using a gradient of 0–100% acetonitrile in water. Fractions were collected for each peak; however, LC–MS analysis did not identify any fraction containing the desired molecular weight. No isolable product was obtained. For algorithmic consistency, the code S2_11-Hy is retained; MeOH blank was used in place of this substrate in subsequent steps.

N-(pivaloyloxy)thiophene-2-carboxamide (S2_12-Hy)

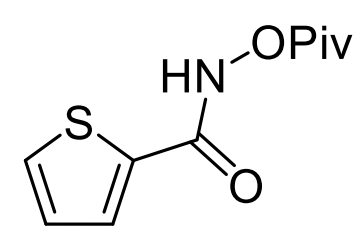

Following **General Procedure A** using thiophene-2-carboxylic acid (256 mg, 2.00 mmol). The crude material was purified by column chromatography (40% EtOAc in hexane) to give hydroxamate ester **S-18** as a white solid (171 mg, 37%). ^{1}H NMR (400 MHz, CD_3OD) δ 7.76 (dt, *J* = 5.1, 1.4 Hz, 1 H, 5-H), 7.72 (dd, *J* = 3.8, 1.2 Hz, 1 H, 3-H), 7.18 (ddd, *J* = 5.2, 3.7, 1.6 Hz, 1 H, 4-H), 1.36 (s, 9 H, Piv CH_3); ^{13}C NMR (101 MHz, CD_3OD) δ 176.2 (Piv C=O), 161.5 (amide C=O), 134.4 (C-2), 131.3 (C-3), 129.4 (C-5), 127.6 (C-4), 38.0 (Piv C), 26.1 (Piv CH_3); HRMS (ESI) m/z Calculated for $C_{10}H_{14}NO_3S$ $[M+H]^+$ 228.0689; Found 228.0688.

(Pyridin-4-yl)formamido 2,2-dimethylpropanoate (S2_13-Hy)

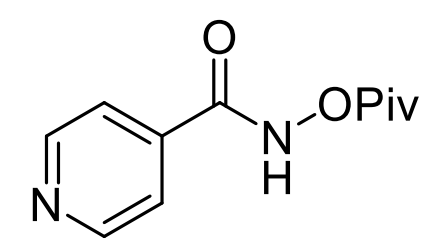

Following **General Procedure A** using isonicotinic acid (246 mg, 2.00 mmol). The crude material was purified by column chromatography (60% EtOAc in hexane) to give hydroxamate ester **S-19** as a yellow solid (212 mg, 47%). ^{1}H NMR (400 MHz, $CDCl_3$) δ 8.79–8.62 (m, 2 H, H-2 and H-6), 7.68 (dd, *J* = 4.0, 1.9 Hz, 2 H, H-3 and H-5), 1.35 (s, 9 H, Piv CH_3); ^{13}C NMR (101 MHz, $CDCl_3$) δ 176.6 (Piv C=O), 164.2 (amide C=O), 150.3 (C-3 and C-5), 138.8 (C-1), 121.4 (C-2 and C-6), 38.5 (Piv C), 27.0 (Piv CH_3); HRMS (ESI) m/z Calculated for $C_{11}H_{15}N_2O_3$ $[M+H]^+$ 223.1077; Found 223.1076.

2. Hit Compound Structural Elucidation

General Procedure B – Rh-Catalysed Annulation

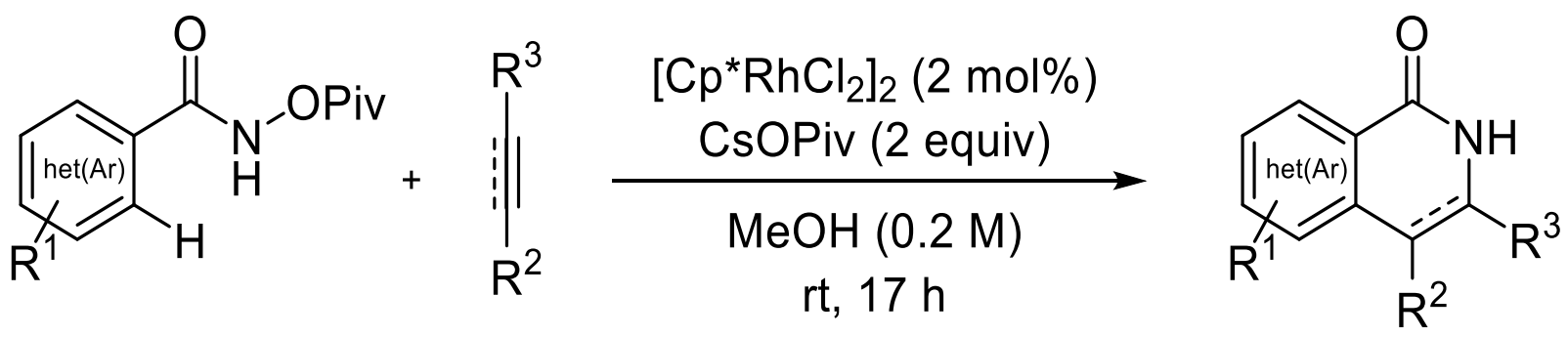

To a solution of $[Cp^*RhCl_2]_2$ (2 mol%, 0.004 mmol) in MeOH (10 mL) was added CsOPiv (0.4 mmol), then the hydroxamate ester of choice (0.2 mmol) and lastly the alkene/alkyne co-substrate (0.22 mmol). The mixture was stirred for 17 h at room temperature. The mixture was concentrated *in vacuo* and the crude residue was purified by column chromatography under the specified conditions to give the corresponding annulation product.

2-(3-(2,5-Dimethylphenyl)-1-oxo-1,2-dihydroisoquinolin-6-yl)acetonitrile (R1-1)

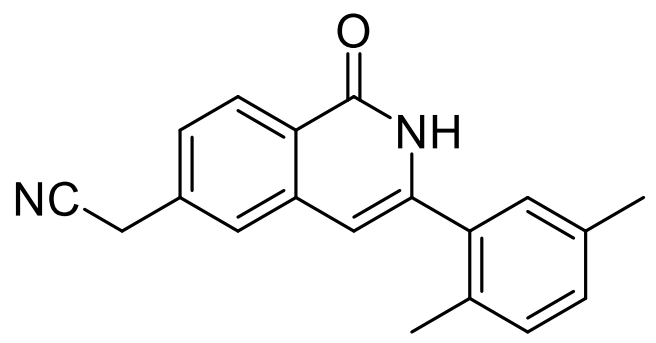

Prepared according to **General Procedure B** using [4-(cyanomethyl)phenyl]formamido 2,2-dimethylpropanoate (**S1_8-Hy**, 57 mg) as substrate and 2-ethynyl-1,4-dimethylbenzene (32 μL) as co-substrate. The crude material was purified by column chromatography (20% to 50% EtOAc in pentane) to give **R1-1** as a colourless oil (11 mg, 20%). ^{1}H NMR (400 MHz, $CDCl_3$) δ 9.02 (br s, 1 H, NH), 8.40 (d, *J* = 8.3 Hz, 1 H, 8-H), 7.58 (d, *J* = 1.8 Hz, 1 H, 5-H), 7.40 (dd, *J* = 8.2, 1.8 Hz, 1 H, 7-H), 7.24–7.19 (m, 3 H, 3 × $HC_{Ar,}$, phenyl-H all), 6.47 (s, 1 H, 4-H), 3.91 (s, 2 H, CH_2CN), 2.38 (s, 3 H, CH_3), 2.37 (s, 3 H, CH_3); ^{13}C NMR (101 MHz, $CDCl_3$) δ 162.6 (amide C=O), 141.0 (phenyl C-1), 138.7 (phenyl C-5), 136.0 (C-6), 134.8 (C-4a), 134.2 (phenyl C-2), 132.8 (C-3), 131.0 (phenyl C-3), 130.4 (phenyl C-4), 129.7 (C-7), 128.7 (C-8), 126.1 (phenyl C-6), 125.4 (C-5), 124.4 (C-8a), 117.1 (CN), 105.9 (C-4), 23.9 (CH_2), 20.8 (CH_3), 19.6 (CH_3); HRMS (ESI) m/z Calculated for $C_{19}H_{17}N_2O$ $[M+H]^+$ 289.1335; Found 289.1333.

6-Bromo-3-(2,5-dimethylphenyl)-5-fluoroisoquinolin-1(2H)-one (R1-2)

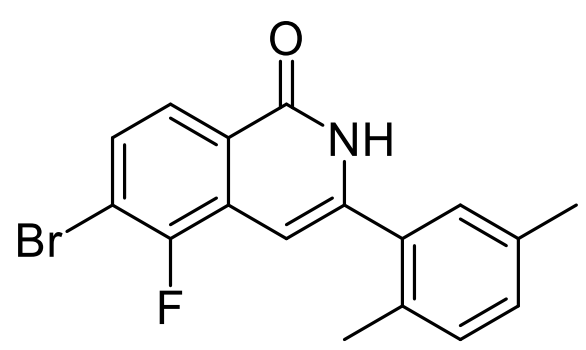

Prepared according to **General Procedure B** using (4-bromo-3-fluorophenyl)formamido 2,2-dimethylpropanoate (**S1_10-Hy**, 68 mg) as substrate and 2-ethynyl-1,4-dimethylbenzene (32 μL) as co-substrate. The crude material was purified by column chromatography (20% to 50% acetone in pentane) to give **R1-2** as a pale yellow oil (18 mg, 25%). ^{1}H NMR (500 MHz, $CDCl_3$) δ 9.14 (br s, 1 H, NH), 8.04 (d, J_{CF} = 8.6 Hz, 1 H, 7-H), 7.60 (dd, J_{CF} = 8.6, 6.3 Hz, 1 H, 8-H), 7.21 (dd, J_{CF} = 4.7, 2.3 Hz, 3 H, all phenyl-H), 6.66 (s, 1 H, 4-H), 2.38 (s, 3 H, CH_3), 2.36 (s, 3 H, CH_3); ^{13}C NMR (126 MHz, $CDCl_3$) δ 161.8 (amide C=O), 154.2 (d, J_{CF} = 256.5 Hz, C-5), 141.8 (phenyl C-1), 136.1 (phenyl C-5), 134.0 (C-3), 132.8 (phenyl C-2), 131.1 (C-8), 130.7 (phenyl C-3), 130.3 (phenyl C-4), 129.7 (phenyl C-6), 128.4 (d, J_{CF} = 17.3 Hz, C-4a), 125.4 (C-8a), 124.0 (d, J_{CF} = 5.0 Hz, C-7), 113.2 (d, J_{CF} = 17.5 Hz, C-6), 98.3 (d, J_{CF} =

5.9 Hz, C-4), 20.8 (CH_3), 19.5 (CH_3); $^{19}F\{^1H\}$ NMR (471 MHz, $CDCl_3$) δ –114.55 (s); HRMS (ESI) m/z Calculated for $C_{17}H_{14}NOBrF$ $[M+H]^+$ 346.0237; Found 346.0231.

5-((2,5-difluorophenoxy)methyl)-2-(trifluoromethyl)thieno[2,3-c]pyridin-7(6H)-one (R1-3)

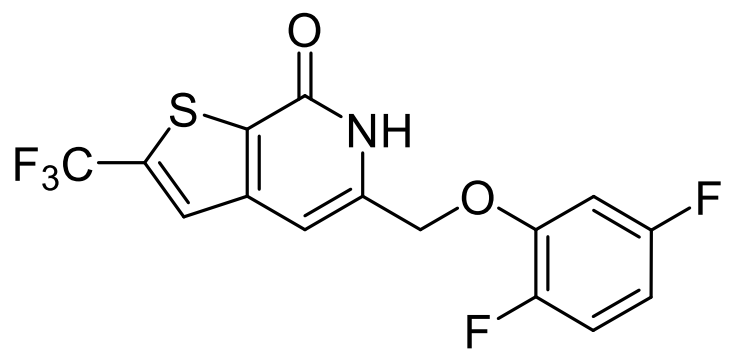

Prepared according to **General Procedure B** using [5-(Trifluoromethyl)thiophen-2-yl]formamido-2,2-dimethylpropanoate (**S1_9-Hy**, 59 mg) as substrate and 2-(allyloxy)-1,4-difluorobenzene (38 mg) as co-substrate. The crude material was purified by column chromatography (20% to 50% ethyl acetate in pentane) to give **R1-3** as a colourless oil (14 mg, 20%). ^1H NMR (400 MHz, $CDCl_3$) δ 10.81 (br s, NH), 7.64–7.57 (d, *J* = 1.1 Hz, 1 H, phenyl 6-H), 7.10 (ddd, *J* = 10.5, 9.0, 5.1 Hz, 1 H, phenyl 3-H), 6.88 (ddd, *J* = 9.4, 6.6, 3.0 Hz, 1 H, phenyl 4-H), 6.80–6.75 (d, *J* = 1.0 Hz, 1 H, 3-H), 6.77–6.68 (m, 1 H, 4-H), 5.11 (s, 2 H, CH_2); ^{13}C NMR (from HSQC, $CDCl_3$) δ 125.8 (phenyl C-6), 117.1 (phenyl C-3), 109.0 (C-3), 104.7 (phenyl C-4), 102.1 (C-4), 68.1 (CH_2); $^{19}F\{^1H\}$ NMR (471 MHz, $CDCl_3$) δ –56.67 (s), –115.76 - –115.87 (m), –138.35 - –138.47 (m); HRMS (ESI) m/z Calculated for $C_{15}H_9NO_2SF_5$ $[M+H]^+$ 362.0269; Found 362.0271.

3-Phenyl-3,4-dihydroisoquinolin-1(2H)-one (R1-4)

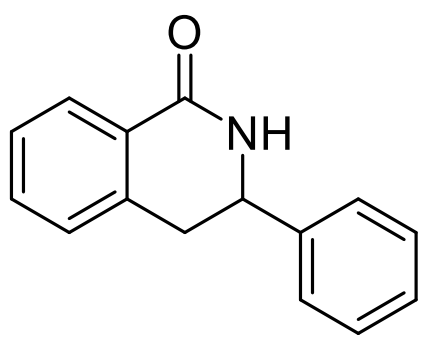

Prepared according to **General Procedure B** using phenylformamido 2,2-dimethylpropanoate (**S1_11-Hy**, 44 mg) as substrate and styrene (25 μL) as co-substrate. The crude material was purified by column chromatography (10% to 40% ethyl acetate in pentane) to give **R1-4** as a colourless oil (24 mg, 54%). ^1H NMR (500 MHz, $CDCl_3$) δ 8.18–8.10 (m, 1 H, 6-H), 7.48 (td, *J* = 7.5, 1.5 Hz, 1 H, 8-H), 7.44–7.33 (m, 6 H, 5-H and all phenyl-H), 7.20 (dd, *J* = 7.5, 1.3 Hz, 1 H, 7-H), 6.10 (br s, 1 H, NH), 4.87 (ddd, *J* = 10.9, 4.9, 1.2 Hz, 1 H, 3-H), 3.31–3.06 (m, 2 H, 4-H); ^{13}C NMR (126 MHz, $CDCl_3$) δ 166.3 (amide C=O), 140.9 (phenyl C-1), 137.6 (C-4a), 132.5 (C-8a), 129.0 (C-6), 128.4 (C-7), 128.3 (phenyl C-2 and C-6), 128.1 (phenyl C-4), 127.31 (C-5), 127.29 (C-8), 126.4 (phenyl C-3 and C-5), 56.2 (C-3), 37.5 (C-4); HRMS (ESI) m/z Calculated for $C_{15}H_{14}NO$ $[M+H]^+$ 224.1070; Found 224.1065. (*DHQ = dihydroisoquinoline scaffold*)

4. Characterisation Directly From Compound Stocks

Following the general reaction protocol described in Section A (reaction set-up, purification, plating and drying) the pure isolated products were stored for future analysis. The products that were flagged as biologically active (with an induction above 5%) were characterised by NMR and HRMS to confirm molecular structure. For NMR analysis, each dry compound of interest was dissolved in $CDCl_3$ (200 μL – 400 μL) and transferred to 3 mm NMR tubes. ^{1}H and ^{13}C NMR spectra were then recorded in series on a 600 MHz spectrometer. Where insufficient material was available to acquire a full ^{13}C NMR spectrum, carbon resonances were assigned based on HSQC and HMBC correlation data. Chemical shifts (δ) are reported in parts per million (ppm) relative to tetramethylsilane (TMS) as the internal standard. *Residual peaks arising from DMSO, used to dissolve the crude reaction mixtures prior to purification, may be present and are noted where applicable. As DMSO is also the solvent used to dissolve compounds prior to biological testing, its presence does not interfere with assay interpretation or compound evaluation.*

3-(p-Tolyl)isoquinolin-1(2H)-one (R2-1)

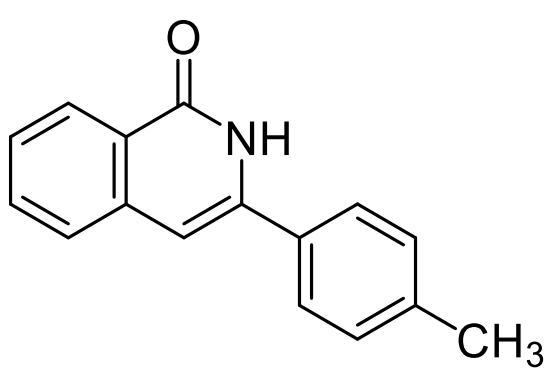

^{1}H NMR (600 MHz, $CDCl_3$) δ 8.84 (br s, 1 H, NH), 8.43–8.39 (m, 1 H, 6-H), 7.69 (dd, *J* = 8.2, 7.0 Hz, 1 H, 8-H), 7.60 (d, *J* = 8.1 Hz, 1 H, 5-H), 7.56–7.53 (m, 2 H, phenyl 2-H and phenyl 6-H), 7.49 (dd, *J* = 8.2, 7.0 Hz, 1 H, 7-H), 7.33 (d, *J* = 8.0 Hz, 2 H, phenyl 3-H and phenyl 5-H), 6.75 (s, 1 H, 4-H), 2.44 (s, 3 H, CH_3); ^{13}C NMR (from HSQC, $CDCl_3$) δ 133.0 (C-8), 130.1 (phenyl C-3 and phenyl C-5), 127.5 (C-6), 126.7 (C-7), 126.6 (C-5), 125.6 (phenyl C-2 and phenyl C-6), 103.8 (C-4), 21.3 (CH_3); (from HMBC, $CDCl_3$) δ 139.8 (phenyl C-1), 133.1 (C-8a), 130.1 (phenyl C-4), 126.4 (C-4a), 125.4 (C-3); HRMS (ESI) m/z Calculated for $C_{16}H_{14}NO$ $[M+H]^+$ 236.1070; Found 236.1069.

3-(Bicyclo[4.2.0]octa-1,3,5-trien-3-yl)-3,4-dihydroisoquinolin-1(2H)-one (R2-2)

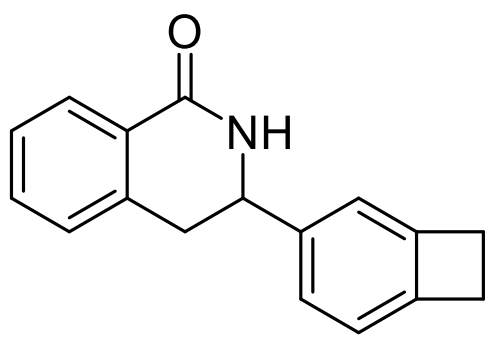

^{1}H NMR (600 MHz, $CDCl_3$) δ 8.12 (dd, *J* = 7.7, 1.4 Hz, 1 H, 6-H), 7.47 (td, *J* = 7.5, 1.5 Hz, 1 H, 8-H), 7.38 (tt, *J* = 7.6, 1.1 Hz, 1 H, 5-H), 7.25–7.16 (m, 2 H, phenyl 2-H and phenyl 6-H), 7.12 (d, *J* = 1.5 Hz, 1 H, phenyl 5-H), 7.07 (dd, *J* = 7.4, 0.9 Hz, 1 H, phenyl 7-H), 5.94 (s, 1 H, NH), 4.81 (dd, *J* = 11.7, 4.4 Hz, 1 H, 3-H), 3.21–3.17 (m, 1 H, 4-H), 3.19 (s, 4 H, all cyclobutyl H), 3.12–3.06 (m, 1 H, 4-H); ^{13}C NMR (151 MHz, $CDCl_3$) δ 166.4 (amide C=O), 146.6 (phenyl C-3), 146.2 (phenyl C-4), 139.6 (phenyl C-1), 137.8 (C-4a), 132.5 (C-6), 128.3 (C-8a), 128.1 (C-7), 127.4 (C-5), 127.3 (C-8), 125.2 (phenyl C-2), 123.0 (phenyl C-5), 120.7 (phenyl C-6), 56.9 (C-3), 37.9 (C-4), 29.5 (2 × cyclobutyl C); HRMS (ESI) m/z Calculated for $C_{17}H_{16}NO$ $[M+H]^+$ 250.1226; Found 250.1224.

6-methyl-3-(o-tolyl)isoquinolin-1(2H)-one (R2-3)

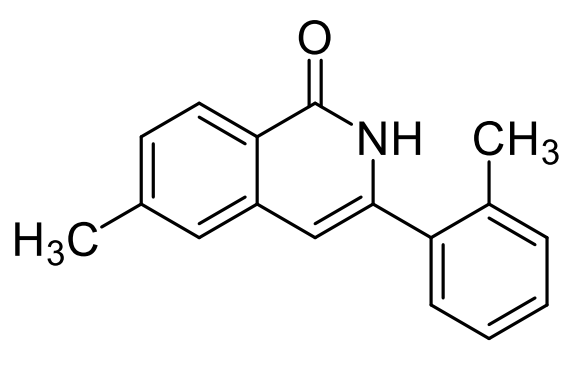

^{1}H NMR (600 MHz, $CDCl_3$) δ 9.08 (br s, 1 H, NH), 8.27 (d, *J* = 8.2 Hz, 1 H, 8-H), 7.39–7.34 (m, 3 H, 5-H, phenyl 5-H and phenyl 6-H), 7.33–7.27 (m, 3 H, 7-H, phenyl 3-H and phenyl 4-H), 6.41 (s, 1 H, 4-H), 2.50 (s, 3 H, phenyl CH_3), 2.39 (s, 3 H, CH_3); ^{13}C NMR (151 MHz, $CDCl_3$) δ 163.2 (amide C=O), 143.5 (C-6), 139.7 (phenyl C-1), 138.3 (phenyl C-2), 136.2 (C-4a), 134.9 (C-3), 130.9 (phenyl C-3), 129.6 (C-7), 129.4

(phenyl C-4), 129.2 (C-8), 128.3 (C-5), 127.4 (phenyl C-6), 126.3 (phenyl C-5), 126.1 (C-8a), 106.4 (C-4), 21.9 (CH_3), 20.1 (CH_3); HRMS (ESI) m/z Calculated for $C_{17}H_{16}NO$ $[M+H]^+$ 250.1226; Found 250.1223.

6-fluoro-3-(p-tolyl)isoquinolin-1(2H)-one (R2-4)

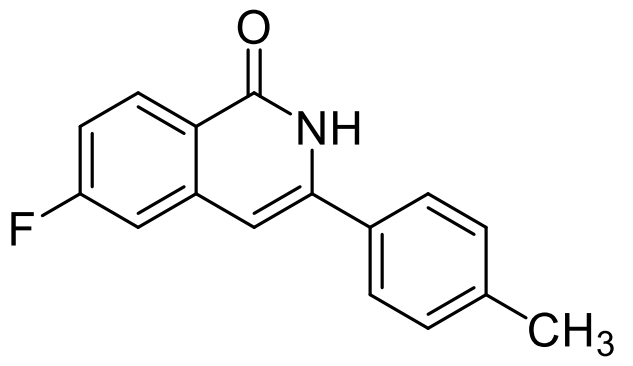

^1H NMR (600 MHz, $CDCl_3$) δ 9.62 (br s, 1 H, NH), 8.39 (dd, J = 8.8, 5.8 Hz, 1 H, 8-H), 7.60–7.56 (m, 2 H, phenyl 2-H and phenyl 6-H), 7.35–7.30 (m, 2 H, phenyl 3-H and phenyl 5-H), 7.21 (dd, J = 9.4, 2.5 Hz, 1 H, 7-H), 7.16 (dd, J = 8.6, 2.5 Hz, 1 H, 5-H), 6.68 (s, 1 H, 4-H), 2.43 (s, 3 H, CH_3); ^{13}C NMR (151 MHz, $CDCl_3$) δ 165.6 (d, J_{CF} = 254.4 Hz, C-6), 163.0 (amide C=O), 140.8 (C-3), 140.7 (d, J_{CF} = 12.6 Hz, C-4a), 140.2 (phenyl C-1), 131.1 (phenyl C-4), 130.7 (d, J_{CF} = 10.9 Hz, C-8), 130.1 (phenyl C-2 and phenyl C-6), 125.9 (phenyl C-3 and phenyl C-5), 121.5 (C-8a), 115.2 (d, J_{CF} = 25.3 Hz, C-5), 111.2 (d, J_{CF} = 21.3 Hz, C-7), 103.2 (d, J_{CF} = 5.4 Hz, C-4), 21.3 (CH_3); $^{19}F\{^1H\}$ NMR (376 MHz, $CDCl_3$) δ – 105.9 (s); HRMS (ESI) m/z Calculated for $C_{16}H_{13}FNO$ $[M+H]^+$ 254.0976; Found 254.0976.

6-fluoro-3-(o-tolyl)isoquinolin-1(2H)-one (R2-5)

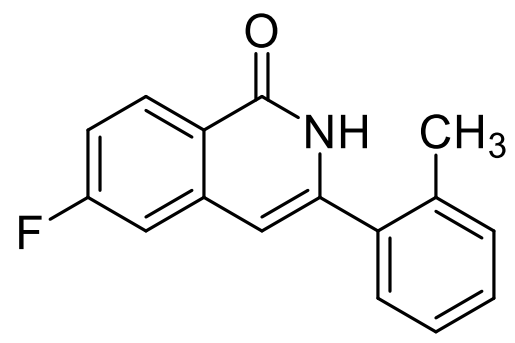

^1H NMR (600 MHz, $CDCl_3$) δ 8.43–8.37 (m, 1 H, 8-H), 7.42–7.37 (m, 2 H, 5-H and 7-H), 7.36–7.29 (m, 2 H, phenyl 5-H and phenyl 6-H), 7.20 (m, 2 H, phenyl 3-H and phenyl 4-H), 6.42 (d, J = 2.6 Hz, 1 H, 4-H), 2.41 (s, 3 H, CH_3); ^{13}C NMR (151 MHz, $CDCl_3$) δ 165.6 (d, J_{CF} = 254.6 Hz, C-6), 162.7 (amide C=O), 141.3 (C-3), 140.4 (d, J_{CF} = 11.9 Hz, C-4a), 136.1 (phenyl C-1), 134.4 (phenyl C-2), 131.0 (C-8a), 130.8 (d, J_{CF} = 11.8 Hz, C-5), 129.7 (phenyl C-3), 129.2 (phenyl C-4), 126.3 (phenyl C-6), 121.5 (phenyl C-5), 115.3 (d, J_{CF} = 21.3 Hz, C-7), 111.2 (d, J_{CF} = 10.1 Hz, C-8), 105.9 (d, J_{CF} = 5.4 Hz, C-4), 20.1 (CH_3); $^{19}F\{^1H\}$ NMR (376 MHz, $CDCl_3$) δ –105.8 (s); HRMS (ESI) m/z Calculated for $C_{16}H_{13}FNO$ $[M+H]^+$ 254.0976; Found 254.0973.

6-fluoro-3-(2-fluorophenyl)-3,4-dihydroisoquinolin-1(2H)-one (R2-6)

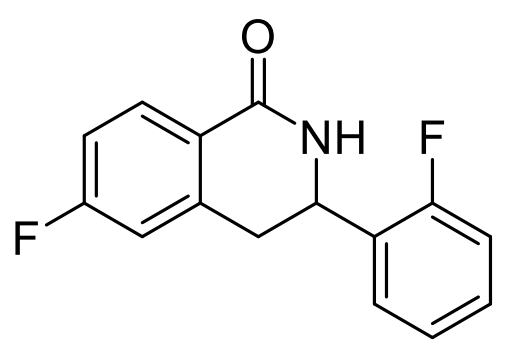

^1H NMR (600 MHz, $CDCl_3$) δ 8.13 (dd, J = 8.6, 5.8 Hz, 1 H, 8-H), 7.38 (td, J = 7.6, 1.7 Hz, 1 H, phenyl 4-H), 7.34–7.28 (m, 1 H, phenyl 3-H), 7.14 (td, J = 7.6, 1.2 Hz, 1 H, 6-H), 7.09 (ddd, J = 10.6, 8.2, 1.2 Hz, 1 H, phenyl 4-H), 7.05 (td, J = 8.6, 2.5 Hz, 1 H, phenyl 5-H), 6.88 (dt, J = 8.7, 1.8 Hz, 1 H, phenyl 6-H), 6.13 (br s, 1 H, NH), 5.25 (ddd, J = 9.1, 5.1, 2.0 Hz, 1 H, 3-H), 3.29 (dd, J = 15.9, 5.1 Hz, 1 H, 4-H_a), 3.18 (dd, J = 15.9, 9.0 Hz, 1 H, 4-H_b); ^{13}C NMR (151 MHz, $CDCl_3$) δ 165.6 (amide C=O), 165.3 (d, J_{CF} = 253.9 Hz, C-6), 160.0 (d, J_{CF} = 247.8 Hz, phenyl C-2), 140.0 (d, J_{CF} = 9.7 Hz, C-4a), 130.9 (d, J_{CF} = 11.5 Hz, C-8), 130.0 (d, J_{CF} = 8.3 Hz, phenyl C-4 and phenyl C-6), 127.7 (d, J_{CF} = 12.5 Hz, phenyl C-1), 127.3 (d, J_{CF} = 3.8 Hz, phenyl C-5), 124.7 (d, J_{CF} = 3.8 Hz, C-8a), 124.6 (d, J_{CF} = 2.9 Hz, C-7), 114.7 (d, J_{CF} = 22.1 Hz, C-5), 114.4 (d, J_{CF} = 22.4 Hz, phenyl C-3), 48.9 (d, J_{CF} = 3.4 Hz, C-3), 35.3 (C-4); $^{19}F\{^1H\}$ NMR (376 MHz, $CDCl_3$) δ –106.2 (s), –118.8 (s); HRMS (ESI) m/z Calculated for $C_{15}H_{12}F_2NO$ $[M+H]^+$ 260.0881; Found 260.0879.

6-methyl-3-phenyl-3,4-dihydroisoquinolin-1(2H)-one (R2-7)

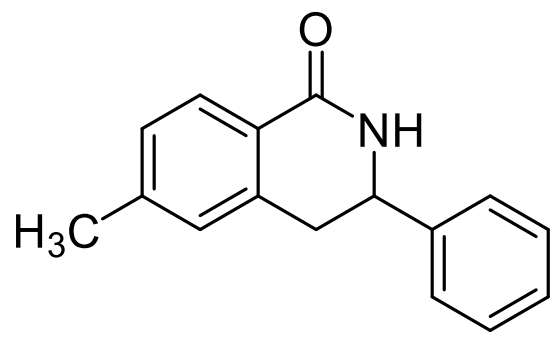

^{1}H NMR (600 MHz, $CDCl_3$) δ 8.02 (d, *J* = 7.9 Hz, 1 H, 8-H), 7.43–7.37 (m, 4 H, phenyl 2-H, phenyl 6-H, phenyl 3-H and phenyl 5-H), 7.36 (dt, *J* = 6.4, 2.8 Hz, 1 H, 5-H), 7.21–7.18 (m, 1 H, phenyl 4-H), 7.00–6.98 (m, 1 H, 7-H), 5.93 (br s, 1 H, NH), 4.84 (ddd, *J* = 11.2, 4.6, 1.2 Hz, 1 H, 3-H), 3.17 (dd, *J* = 15.7, 11.2 Hz, 1 H, 4-H_a), 3.08 (ddd, *J* = 15.9, 4.7, 1.2 Hz, 1 H, 4-H_b), 2.39 (s, 3 H, CH_3); ^{13}C NMR (151 MHz, $CDCl_3$) δ 166.5 (amide C=O), 143.1 (C-6), 141.0 (C-4a), 137.5 (phenyl C-1), 129.0 (C-8), 128.4 (phenyl C-3 and phenyl C-5), 128.11 (C-8a), 128.09 (C-5), 127.9 (phenyl C-4), 126.4 (phenyl C-2 and phenyl C-6), 125.7 (C-7), 56.3 (C-3), 37.5 (C-4), 21.6 (CH_3); HRMS (ESI) m/z Calculated for $C_{16}H_{16}NO$ $[M+H]^+$ 238.1226; Found 238.1224.

6-methyl-3-(p-tolyl)isoquinolin-1(2H)-one (R2-8)

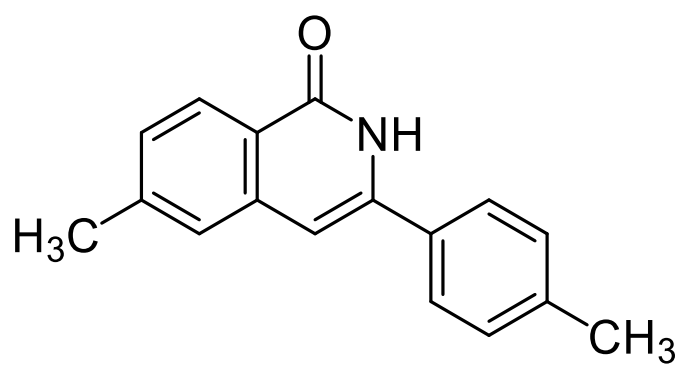

^{1}H NMR (600 MHz, $CDCl_3$) δ 9.00 (br s, 1 H, NH), 8.28 (d, *J* = 8.2 Hz, 1 H, 8-H), 7.57–7.50 (m, 2 H, phenyl 2-H and phenyl 6-H), 7.38–7.36 (m, 1 H, 7-H), 7.32–7.30 (m, 2 H, phenyl 3-H and phenyl 5-H), 6.67 (s, 1 H, 5-H), 2.49 (s, 3 H, CH_3), 2.42 (s, 3 H, CH_3); ^{13}C NMR (151 MHz, $CDCl_3$) δ 163.4 (amide C=O), 143.5 (C-6), 139.8 (C-4a), 139.2 (phenyl C-4), 138.5 (phenyl C-1), 131.6 (C-3), 130.0 (phenyl C-3 and phenyl C-5), 129.3 (C-8a), 128.3 (C-5), 127.4 (C-7), 126.3 (C-8), 125.6 (phenyl C-2 and phenyl C-6), 103.7 (C-4), 21.9 (CH_3), 21.3 (CH_3); HRMS (ESI) m/z Calculated for $C_{17}H_{16}NO$ $[M+H]^+$ 250.1226; Found 250.1225.

2-(3-(2-fluorophenyl)-1-oxo-1,2,3,4-tetrahydroisoquinolin-7-yl)acetonitrile (R2-9)

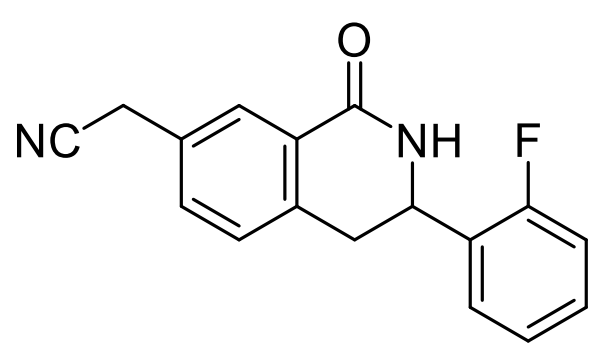

^{1}H NMR (600 MHz, $CDCl_3$) δ 8.13 (d, *J* = 8.0 Hz, 1 H, 8-H), 7.37 (td, *J* = 7.6, 1.7 Hz, 1 H, phenyl 4-H), 7.34–7.29 (m, 2 H, 6-H and phenyl 3-H), 7.21–7.19 (m, 1 H, phenyl 6-H), 7.16–7.08 (m, 2 H, 5-H and phenyl 5-H), 6.12 (br s, 1 H, NH), 5.25 (ddd, *J* = 9.0, 5.1, 2.1 Hz, 1 H, 3-H), 3.79 (s, 2 H, CH_2), 3.33 (dd, *J* = 15.9, 5.1 Hz, 1 H, 4-H_a), 3.20 (dd, *J* = 15.8, 8.9 Hz, 1 H, 4-H_b); ^{13}C NMR (151 MHz, $CDCl_3$) δ 165.7 (amide C=O), 160.0 (d, J_{CF} = 264.2 Hz, phenyl C-2), 138.2 (C-6), 134.5 (C-4a), 130.0 (d, J_{CF} = 8.7 Hz, phenyl C-5), 129.0 (C-8a), 128.2 (C-8), 127.6 (d, J_{CF} = 13.3 Hz, phenyl C-4), 127.3 (d, J_{CF} = 2.6 Hz, phenyl C-1), 127.0 (C-7 and C-5), 124.7 (d, J_{CF} = 3.4 Hz, phenyl C-6), 117.2 (CN), 115.9 (d, J_{CF} = 21.5 Hz, phenyl C-3), 48.9 (C-3), 35.1 (C-4), 23.7 (CH_2); ^{19}F{^{1}H} NMR (376 MHz, $CDCl_3$) δ –118.8; HRMS (ESI) m/z Calculated for $C_{17}H_{14}FN_2O$ $[M+H]^+$ 281.1085; Found 281.1081.

2-(1-oxo-3-phenyl-1,2,3,4-tetrahydroisoquinolin-6-yl)acetonitrile (R2-10)

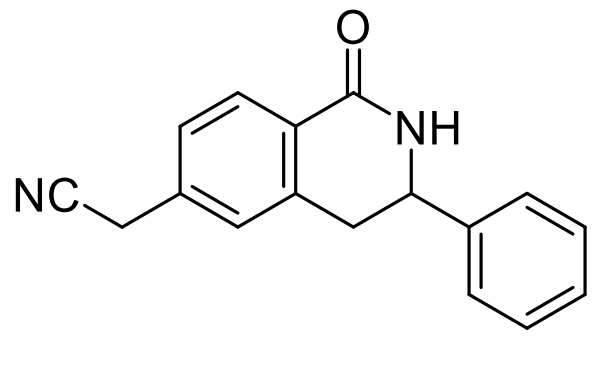

^{1}H NMR (600 MHz, $CDCl_3$) δ 8.13 (dt, *J* = 8.0, 2.2 Hz, 1 H, 8-H), 7.43–7.38 (m, 4 H, phenyl 6-H, phenyl 2-H, phenyl 3-H and phenyl 5-H), 7.38–7.35 (m, 1 H, phenyl 4-H), 7.33 (d, *J* = 8.1 Hz, 1 H, 7-H), 7.21 (s, 1 H, 5-H), 6.11 (br s, 1 H, NH), 4.92–4.81 (m, 1 H, 3-H), 3.79 (s, 2 H, CH_2), 3.27–3.12 (m, 2 H, 4-H); ^{13}C NMR (151 MHz, $CDCl_3$) δ 165.6 (amide C=O), 140.5 (phenyl C-1), 138.6 (C-4a), 134.4 (C-6), 129.1 (phenyl C-3, phenyl C-5 and C-8a), 129.0 (C-5), 128.6 (C-7), 127.0 (C-8), 126.9

(phenyl C-4), 126.4 (phenyl C-2 and phenyl C-6), 117.2 (CN), 56.0 (C-3), 37.3 (C-4), 23.7 (CH_2); HRMS (ESI) m/z Calculated for $C_{17}H_{15}N_2O$ $[M+H]^+$ 263.1179; Found 263.1176.

2-(1-oxo-3-(p-tolyl)-1,2-dihydroisoquinolin-6-yl)acetonitrile (R2-11)

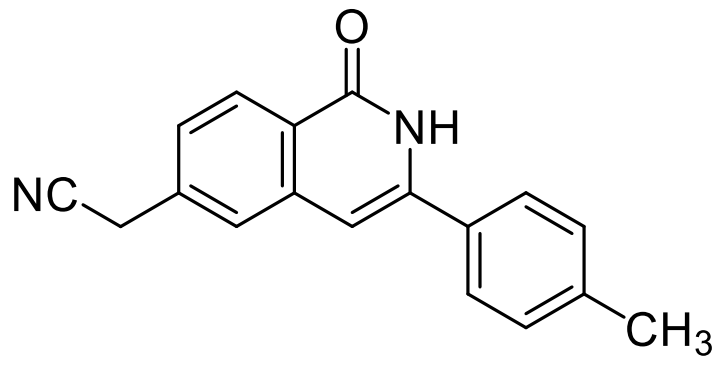

^{1}H NMR (600 MHz, $CDCl_3$) δ 9.28 (br s, 1 H, NH), 8.41 (d, *J* = 8.2 Hz, 1 H, 8-H), 7.61 (q, *J* = 0.9 Hz, 1 H, 5-H), 7.59–7.55 (m, 2 H, phenyl 2-H and phenyl 6-H), 7.41–7.37 (m, 1 H, 7-H), 7.36–7.32 (m, 2 H, phenyl 3-H and phenyl 5-H), 6.74 (s, 1 H, 4-H), 3.92 (s, 2 H, CH_2), 2.45 (s, 3 H, CH_3); ^{13}C NMR (151 MHz, $CDCl_3$) δ 163.0 (amide C=O), 140.4 (phenyl C-4), 140.3 (C-4a), 139.0 (C-6), 134.8 (C-3), 131.1 (phenyl C-1), 130.2 (phenyl C-2 and phenyl C-6), 128.7 (C-7), 126.0 (C-5), 125.8 (phenyl C-3 and phenyl C-5), 125.5 (C-8), 124.5 (C-8a), 117.2 (CN), 103.2 (C-4), 23.9 (CH_2), 21.4 (CH_3); HRMS (ESI) m/z Calculated for $C_{18}H_{15}N_2O$ $[M+H]^+$ 275.1179; Found 275.1176.

2-(1-oxo-3-(o-tolyl)-1,2-dihydroisoquinolin-6-yl)acetonitrile (R2-12)

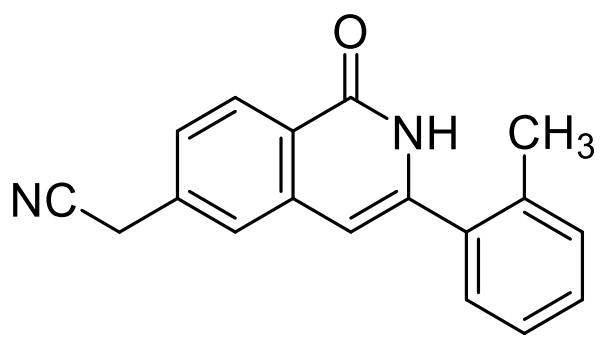

^{1}H NMR (600 MHz, $CDCl_3$) δ 8.85 (br s, 1 H, NH), 8.34 (d, *J* = 1.9 Hz, 1 H, 8-H), 7.69 (dd, *J* = 8.2, 2.0 Hz, 1 H, phenyl 5-H), 7.62 (d, *J* = 8.1 Hz, 1 H, 7-H), 7.41–7.36 (m, 2 H, 5-H and phenyl 6-H), 7.35–7.29 (m, 2 H, phenyl 3-H and phenyl 4-H), 6.49 (s, 1 H, 4-H), 3.91 (s, 2 H, CH_2), 2.40 (s, 3 H, CH_3); ^{13}C NMR (151 MHz, $CDCl_3$) δ 162.5 (amide C=O), 140.7 (phenyl C-2), 138.7 (phenyl C-1), 136.0 (C-4a), 134.9 (C-6), 134.4 (C-3), 131.1 (phenyl C-3), 129.8 (phenyl C-4), 129.1 (phenyl C-6), 128.7 (C-8), 128.4 (C-8a), 126.5 (C-5), 126.2 (C-7), 125.5 (phenyl C-5), 117.2 (CN), 106.0 (C-4), 27.0 (CH_3), 23.9 (CH_2); HRMS (ESI) m/z Calculated for $C_{18}H_{15}N_2O$ $[M+H]^+$ 275.1179; Found 275.1176.

2-methyl-5-(p-tolyl)-5,6-dihydrothieno[2,3-c]pyridin-7(4H)-one (R2-14)

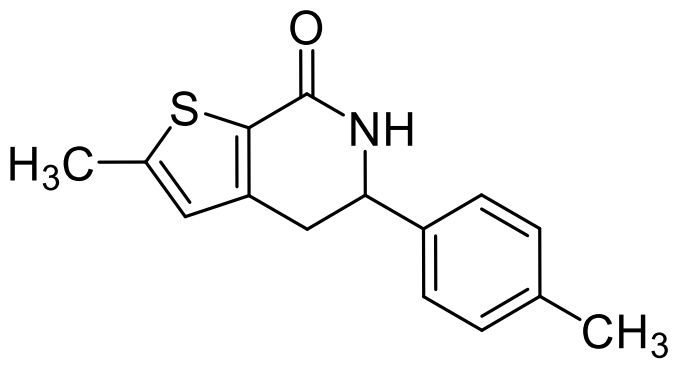

^{1}H NMR (600 MHz, $CDCl_3$) δ 7.30 (d, *J* = 8.0 Hz, 2 H, phenyl 2-H and phenyl 6-H), 7.26–7.09 (d, *J* = 7.9 Hz, 2 H, phenyl 3-H and phenyl 5-H), 6.61 (d, *J* = 1.2 Hz, 1 H, 3-H), 5.56 (br s, 1 H, NH), 4.89 (dd, *J* = 11.3, 5.6 Hz, 1 H, 3-H), 3.09–2.88 (m, 2 H, 4-H), 2.54 (d, *J* = 1.0 Hz, 3 H, CH_3), 2.38 (s, 3 H, CH_3); ^{13}C NMR (151 MHz, $CDCl_3$) δ 163.3 (C=O amide), 156.1 (C-3a), 147.4 (phenyl C-1), 144.5 (phenyl C-4), 138.2 (C-2a), 138.0 (C-3), 129.6 (phenyl C-3 and phenyl C-5), 126.4 (phenyl C-2 and phenyl C-6), 125.4 (C-2), 57.6 (C-5), 34.0 (C-4), 21.1 (CH_3), 16.0 (CH_3); HRMS (ESI) m/z Calculated for $C_{15}H_{16}NOS$ $[M+H]^+$ 258.0947; Found 258.0945.

2-methyl-5-phenyl-5,6-dihydrothieno[2,3-c]pyridin-7(4H)-one (R2-15)

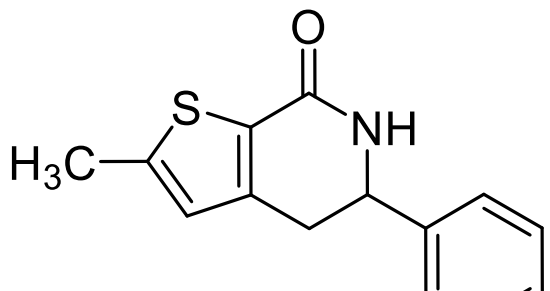

R2-15a

+

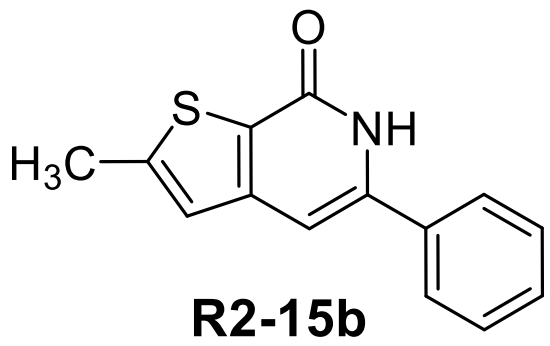

R2-15b

Isolated as a 2:1 mixture of isomers (**R2-15a** and the corresponding unsaturated annulation product **R2-15b**, respectively). The characterisation for the major **R2-15a** is provided below: ^{1}H NMR (600 MHz, $CDCl_3$) δ 7.50 (d, *J* = 3.7 Hz, 1 H, phenyl 4-H), 7.45–7.33 (m, 5 H, phenyl H all), 6.78 (3-H), 5.61 (br s, 1 H, NH), 4.90 (dd, *J* = 11.7, 5.3 Hz, 1 H, 5-H), 3.04 (ddd, *J* = 16.3, 5.3 Hz, 1 H, 4-H_a), 2.97 (dd, *J* = 16.2, 11.7 Hz, 1 H, 4-H_b), 2.52 (s, 3 H, CH_3); ^{13}C NMR

(151 MHz, $CDCl_3$) δ 163.4 (amide C=O), 147.6 (C-3a), 144.4 (phenyl C-1), 140.9 (C-2a), 131.0 (C-3), 129.0 (phenyl C-3 and phenyl C-5), 126.5 (phenyl C-4), 126.3 (phenyl C-2 and phenyl C-6), 125.4 (C-2), 57.8 (C-5), 38.5 (C-4), 16.0 (CH_3); HRMS (ESI) m/z Calculated for **R2-15a** $C_{14}H_{14}NOS$ [M+H] 244.0791; Found 244.0790; Calculated for **R2-15b** $C_{14}H_{12}NOS$ $[M+H]^+$ 242.0645; Found 242.0644.

2-(1-oxo-3-phenyl-1,2,3,4-tetrahydroisoquinolin-7-yl)acetonitrile (R2-16)

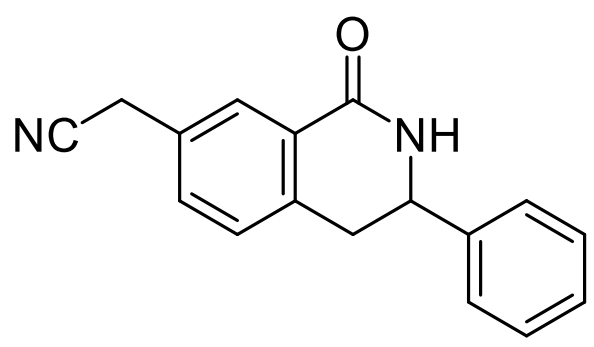

^1H NMR (600 MHz, $CDCl_3$) δ 8.04 (d, J = 2.1 Hz, 1 H, 8-H), 7.51–7.47 (m, 1 H, 6-H), 7.42–7.38 (m, 4 H, phenyl 2-H, phenyl 3-H, phenyl 5-H and phenyl 6-H), 7.38–7.34 (m, 1 H, phenyl 4-H), 7.23 (d, J = 7.8 Hz, 1 H, 5-H), 6.23 (br s, 1 H, NH), 4.86 (ddd, J = 10.1, 5.6, 1.4 Hz, 1 H, 3-H), 3.80 (s, 2 H, CH_2), 3.20–3.13 (m, 2 H, 4-H_a and 4-H_b); ^{13}C NMR (151 MHz, $CDCl_3$) δ 165.5 (C=O amide), 140.5 (C-8a), 137.4 (phenyl C-1), 131.7 (C-8), 129.2 (C-7), 129.04 (phenyl C-2 and phenyl C-6), 129.02 (C-6), 128.5 (C-5), 128.4 (C-4a), 127.6 (phenyl C-4), 126.3 (phenyl C-3 and phenyl C-5), 117.4 (CN), 55.9 (C-3), 36.9 (C-4), 23.3 (CH_2); HRMS $C_{17}H_{15}N_2O$ $[M+H]^+$ 263.1179; Found 263.1179.

2-(1-oxo-3-(o-tolyl)-1,2-dihydroisoquinolin-7-yl)acetonitrile (R2-17)

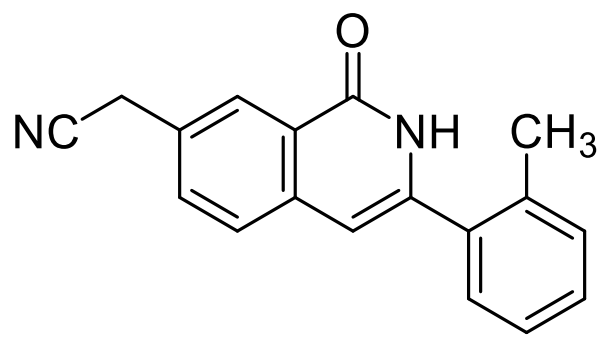

^1H NMR (600 MHz, $CDCl_3$) δ 8.85 (br s, 1 H, NH), 8.34 (d, J = 1.9 Hz, 1 H, 8-H), 7.69 (dd, J = 8.2, 2.0 Hz, 1 H, 5-H), 7.61 (d, J = 8.1 Hz, 1 H, 6-H), 7.41–7.35 (m, 2 H, phenyl 5-H and phenyl 6-H), 7.35–7.29 (m, 2 H, phenyl 3-H and phenyl 4-H), 3.91 (s, 2 H, CH_2), 2.40 (s, 3 H, CH_3); ^{13}C NMR (151 MHz, $CDCl_3$) δ 162.5 (C=O amide), 140.4 (C-8a), 137.8 (phenyl C-1), 136.1 (C-3), 134.4 (C-4a), 132.3 (C-8), 131.1 (C-5), 129.8 (phenyl C-2), 129.1 (phenyl C-6), 128.4 (phenyl C-3), 127.6 (phenyl C-5), 127.1 (phenyl C-4), 126.4 (C-6), 125.2 (C-7), 117.5 (CN), 106.0 (C-4), 23.7 (CH_2), 20.1 (CH_3); HRMS $C_{18}H_{15}N_2O$ $[M+H]^+$ 275.1179; Found 275.1178.

2-(3-(2,5-dimethylphenyl)-1-oxo-1,2,3,4-tetrahydroisoquinolin-7-yl)acetonitrile (R2-18)

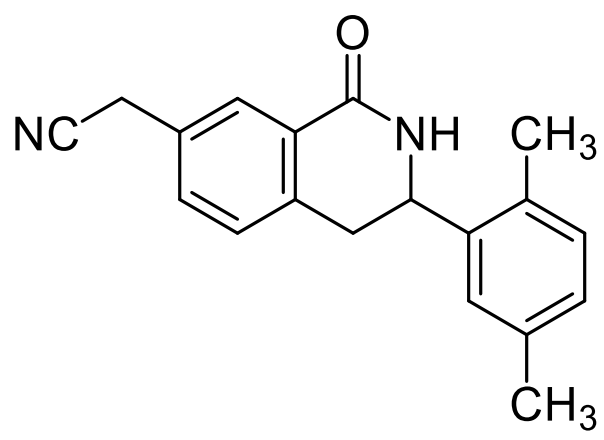

^1H NMR (600 MHz, $CDCl_3$) δ 8.07 (d, J = 2.1 Hz, 1 H, 8-H), 7.52 (dd, J = 7.8, 2.1 Hz, 1 H, 6-H), 7.32 (d, J = 1.5 Hz, 1 H, phenyl 6-H), 7.28–7.25 (m, 1 H, 5-H), 7.11 (d, J = 7.4 Hz, 1 H, phenyl 3-H), 7.09–7.05 (m, 1 H, phenyl 4-H), 5.88 (br s, 1 H, NH), 5.10 (dd, J = 11.8, 4.4 Hz, 1 H, 3-H), 3.82 (s, 2 H, CH_2), 3.15 (dd, J = 15.9, 11.8 Hz, 1 H, 4-H_a), 3.11–3.05 (m, 1 H, 4-H_b), 2.34 (2 × s, 6 H, 2 × CH_3); ^{13}C NMR (from HSQC, $CDCl_3$) δ 131.1 (phenyl C-3), 129.1 (phenyl C-4), 128.0 (C-8), 126.2 (phenyl C-6), 120.4 (C-5), 23.3 (CH_2), 21.2 (CH_3),18.8 (CH_3) other signals not observed due to low quantity of sample; HRMS (ESI) m/z Calculated for $C_{19}H_{19}N_2O$ $[M+H]^+$ 291.1492; Found 291.1490.

6-bromo-5-fluoro-3-(o-tolyl)isoquinolin-1(2H)-one (R2-19)

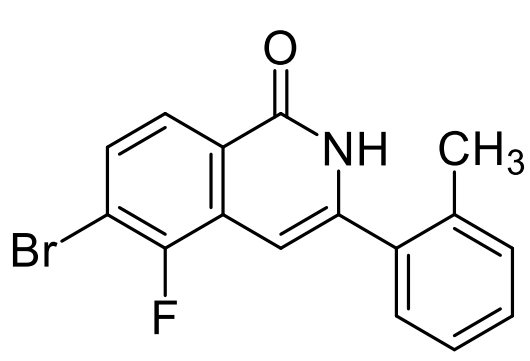

^1H NMR (600 MHz, $CDCl_3$) δ 8.58 (br s, 1 H, NH), 8.08 (d, J = 8.6 Hz, 1 H, 7-H), 7.62 (dd, J = 8.6, 6.4 Hz, 1 H, 8-H), 7.45–7.29 (m, 4 H, all phenyl H), 6.67 (s, 1 H, 4-H), 2.41 (s, 3 H, CH_3); ^{13}C NMR (from HSQC, $CDCl_3$) δ 131.2 (phenyl C-6), 130.4 (C-8), 130.1 (phenyl C-3), 129.1 (phenyl C-5), 126.6 (phenyl C-4), 124.2 (C-7), 98.4 (C-4), 20.1 (CH_3); (from HMBC, $CDCl_3$) δ 161.7 (C=O amide),

153.9 (C-5), 141.5 (C-3), 135.9 (phenyl C-1), 134.4 (phenyl C-2), 134.2 (C-4a), 128.2 (C-6), 125.4 (C-8a); $^{19}F\{^1H\}$ NMR (376 MHz, $CDCl_3$) δ –114.4 (s); HRMS (ESI) m/z Calculated for $C_{16}H_{12}BrFNO$ $[M+H]^+$ 332.0081; Found 332.0081.

2-(3-cyclopentyl-1-oxo-1,2-dihydroisoquinolin-7-yl)acetonitrile (R2-O1)

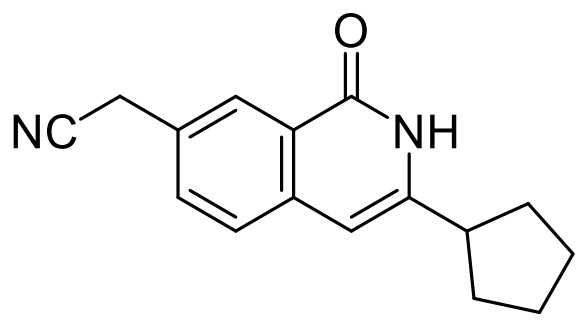

^1H NMR (600 MHz, $CDCl_3$) δ 8.84 (br s, 1 H, NH), 8.30–8.23 (m, 1 H, 8-H), 7.63 (dd, J = 8.2, 2.0 Hz, 1 H, 5-H), 7.52 (d, J = 8.2 Hz, 1 H, 6-H), 6.37–6.32 (m, 1 H, 4-H), 3.87 (s, 2 H, CH_2), 2.95 (p, J = 8.3 Hz, 1 H, cyclopentyl 1-H), 2.17–2.07 (m, 2 H, cyclopentyl 2-H_a and cyclopentyl 5-H_a), 1.86 (qd, J = 4.8, 2.0 Hz, 2 H, cyclopentyl 2-H_b and cyclopentyl 5-H_b), 1.74 (dddd, J = 17.3, 14.9, 12.3, 8.4 Hz, 4 H, cyclopentyl 3-H and 4-H); ^{13}C NMR (151 MHz, $CDCl_3$) δ 145.1 (C-3), 138.2 (C-6), 132.2 (C-5), 127.6 (C-7), 126.9 (C-8), 117.6 (CN), 101.9 (C-4), 43.4 (CH_2), 31.7 (cyclopentyl C-1), 25.3 (cyclopentyl C-2 and cyclopentyl C-5), 23.6 (cyclopentyl C-3 and cyclopentyl C-4) – not observed: amide C=O, C-4a and C-8a due to low quantity of sample; HRMS (ESI) m/z Calculated for $C_{16}H_{17}N_2O$ $[M+H]^+$ 253.1335; Found 253.1335.

6-bromo-5-fluoro-3-(4-fluorophenyl)-3,4-dihydroisoquinolin-1(2H)-one (R2-O2)

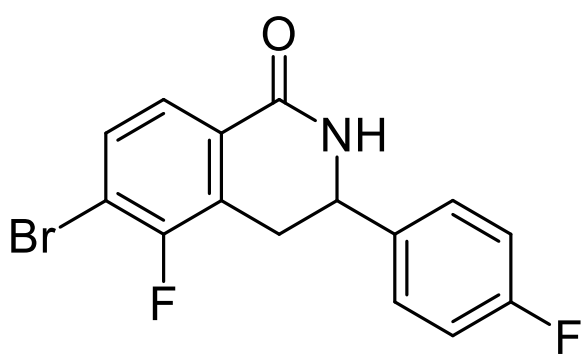

^1H NMR (600 MHz, $CDCl_3$) δ 7.86–7.79 (m, 1 H, 8-H), 7.59 (dd, J = 8.2, 6.5 Hz, 1 H, 7-H), 7.43–7.35 (m, 2 H, phenyl 2-H and phenyl 6-H), 7.16–7.05 (m, 2 H, phenyl 3-H and phenyl 5-H), 5.99 (br s, 1 H, NH), 4.86 (dd, J = 11.2, 4.6 Hz, 1 H, 3-H), 3.35 (dd, J = 16.4, 4.7 Hz, 1 H, 4-H_a), 3.06 (dd, J = 16.4, 11.2 Hz, 1 H, 4-H_b); ^{13}C NMR (151 MHz, $CDCl_3$) δ 164.6 (amide C=O), 162.7 (d, J_{CF} = 246.5 Hz, C-5), 155.4 (d, J_{CF} = 247.2 Hz, phenyl C-4), 136.0 (d, J_{CF} = 3.1 Hz, phenyl C-1), 132.0 (C-8a), 129.2 (d, J_{CF} = 3.5 Hz, C-8), 128.2 (d, J_{CF} = 2.8 Hz, phenyl C-2 and phenyl C-6), 125.6 (d, J_{CF} = 20.1 Hz, C-4a), 124.6 (d, J_{CF} = 4.7 Hz, C-7), 116.2 (d, J_{CF} = 4.7 Hz, phenyl C-3 and phenyl C-5), 114.2 (d, J_{CF} = 21.1 Hz, C-6), 55.02 (C-3), 30.40 (C-4); $^{19}F\{^1H\}$ NMR (376 MHz, $CDCl_3$) δ –111.93 (s), –112.70 (s); HRMS (ESI) m/z Calculated for $C_{15}H_{11}BrF_2NO$ $[M+H]^+$ 339.1598; Found 339.1576.

References

1. M.-A. Bray, S. Singh, H. Han, C. T. Davis, B. Borgeson, C. Hartland, M. Kost-Alimova, S. M. Gustafsdottir, C. Gibson, and A. E. *Nat. Protoc.* 2016, **11**, 1757.
2. M. H. Woehrmann, W. M. Bray, J. K. Durbin, S. C. Nisam, A. K. Michael, E. Glassey, J. M. Stuart and R. S. Lokey, *Mol. BioSyst.* 2013, **9**, 2604.
3. T. Voigt, C. Gerding-Reimers, T. T. Ngoc Tran, S. Bergmann, H. Lachance, B. Schölermann, A. Brockmeyer, P. Janning, S. Ziegler and H. Waldmann, *Angew. Chem. Int. Ed.*, 2013, **52**, 410–414.
4. M. L. Shelanski, F. Gaskin and C. R. Cantor, *PNAS*, 1973, **70**, 765–768.

PivO—NHBoc

^{1}H NMR (400 MHz, CD_3OD)

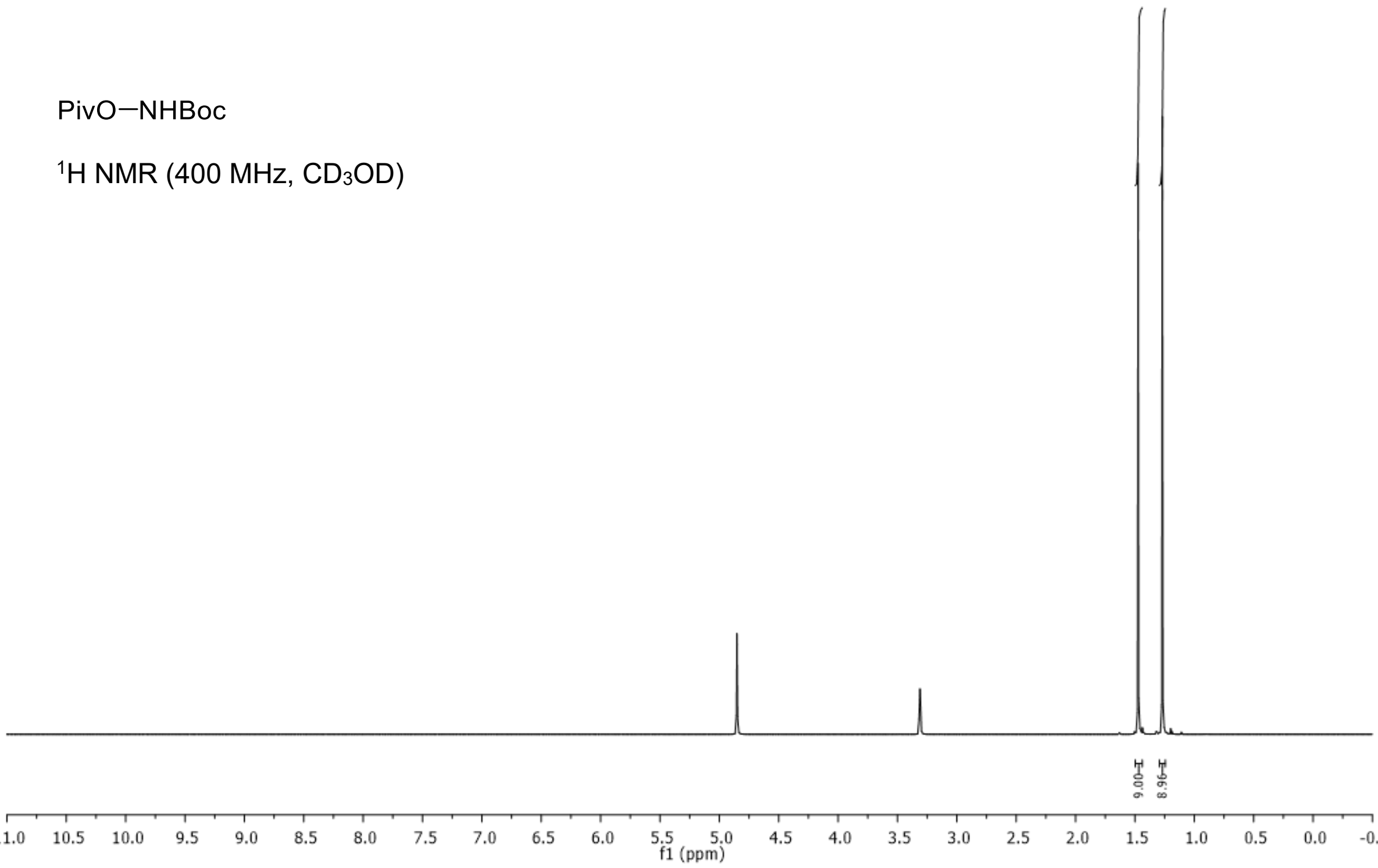

PivO—NHBoc

^{13}C NMR (126 MHz, CD_3OD)

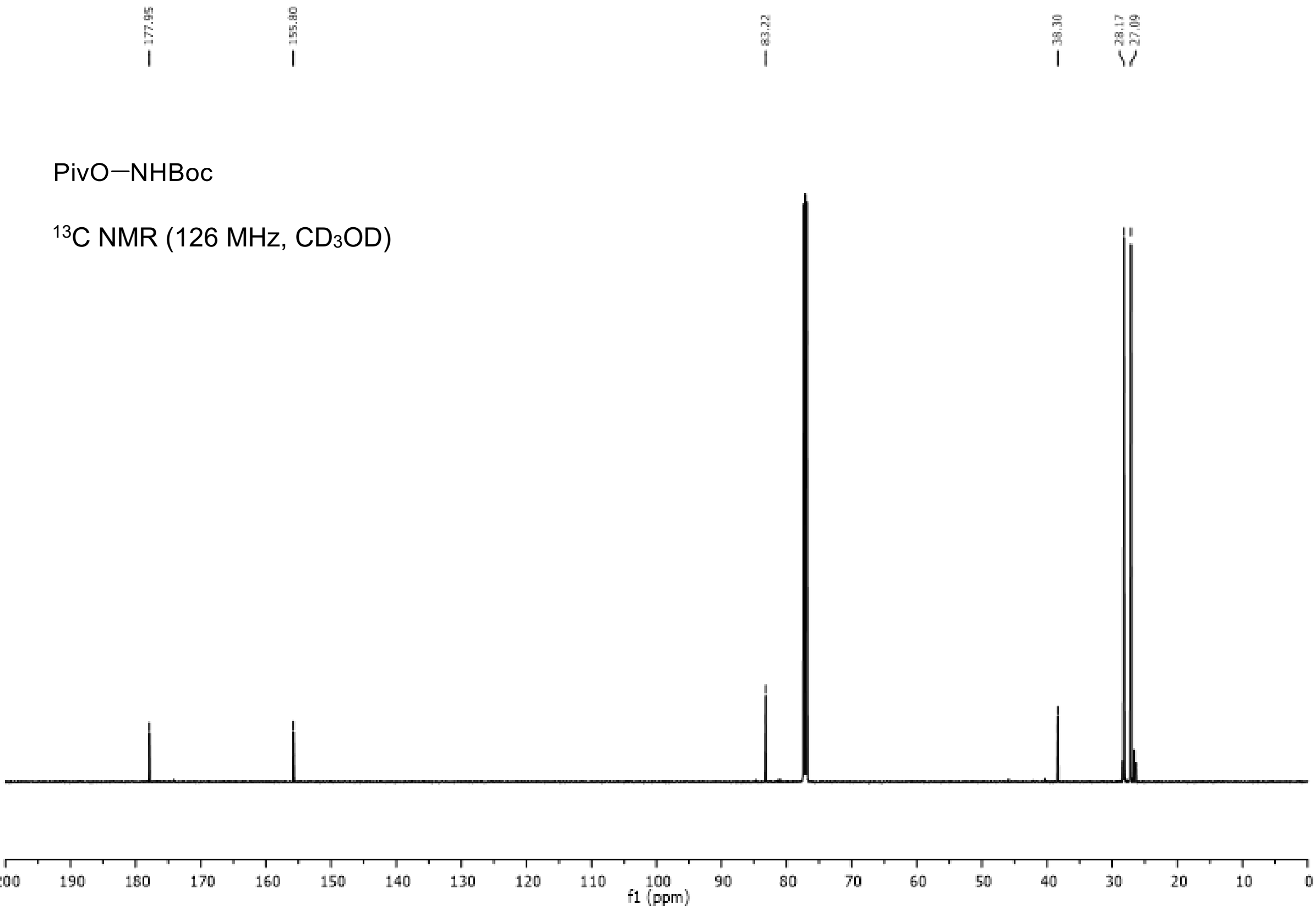

PivO—NH_2•TfOH

SI-1

^{1}H NMR (400 MHz, CD_3OD)

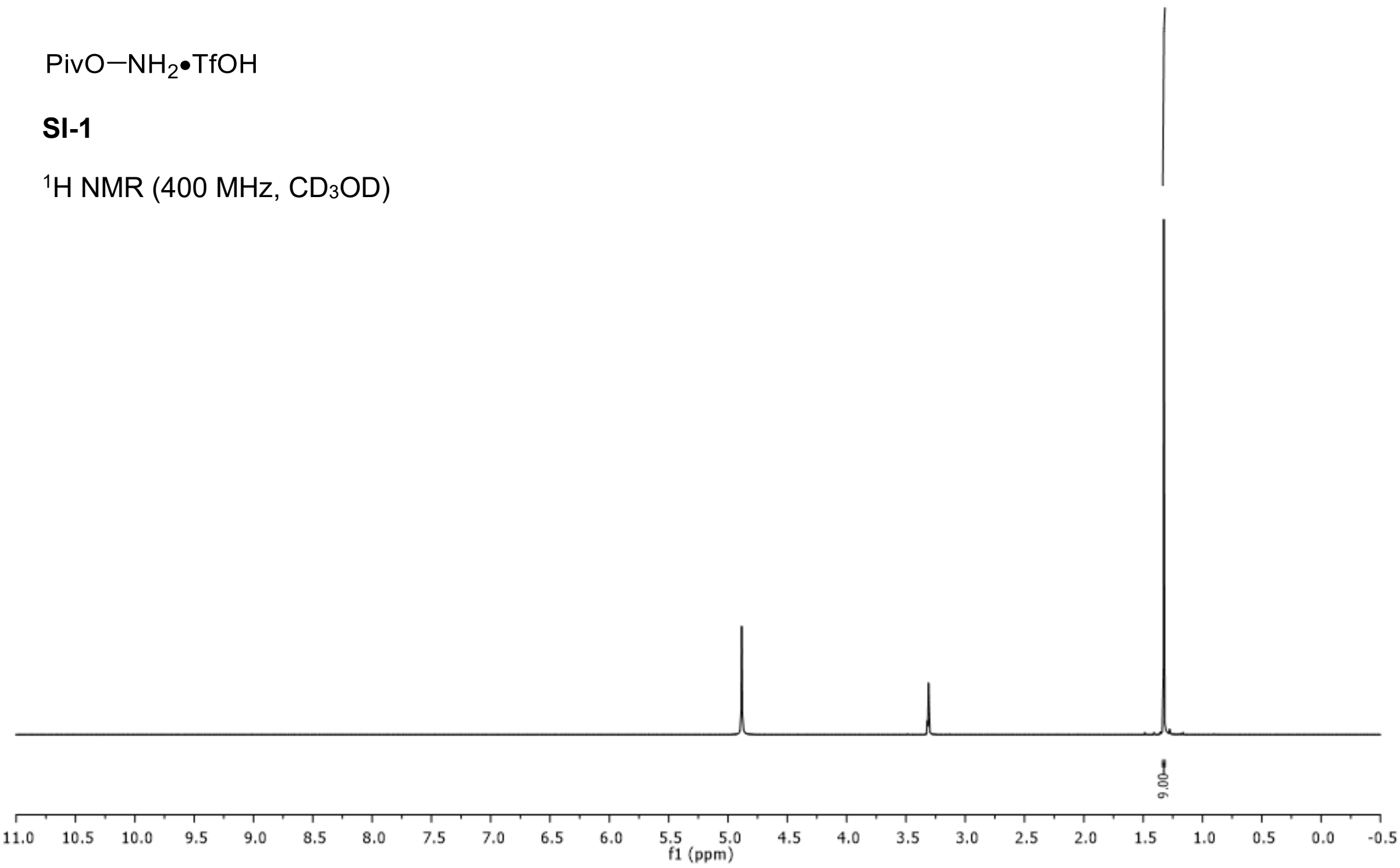

PivO—NH_2•TfOH

SI-1

^{13}C NMR (126 MHz, CD_3OD)

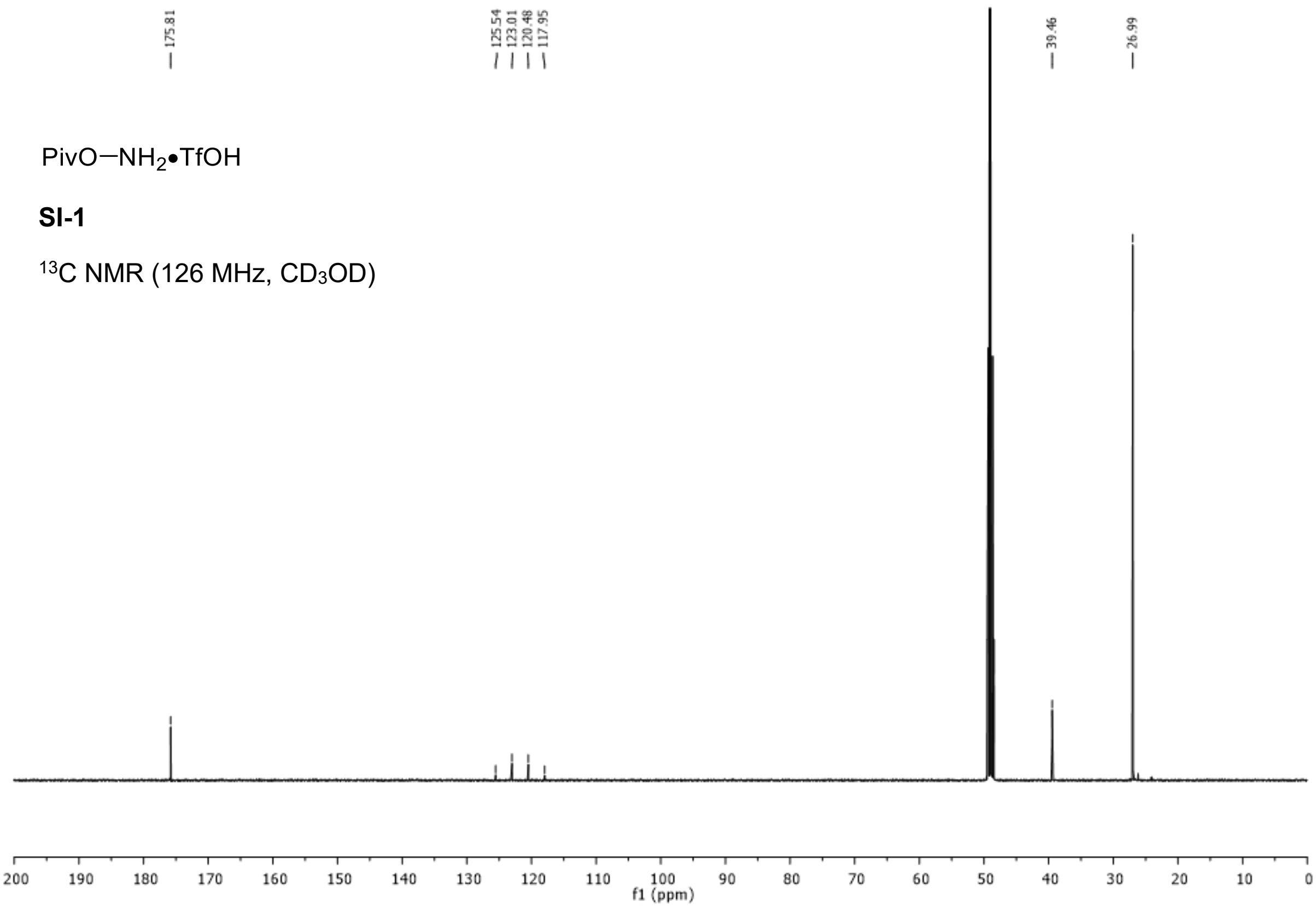

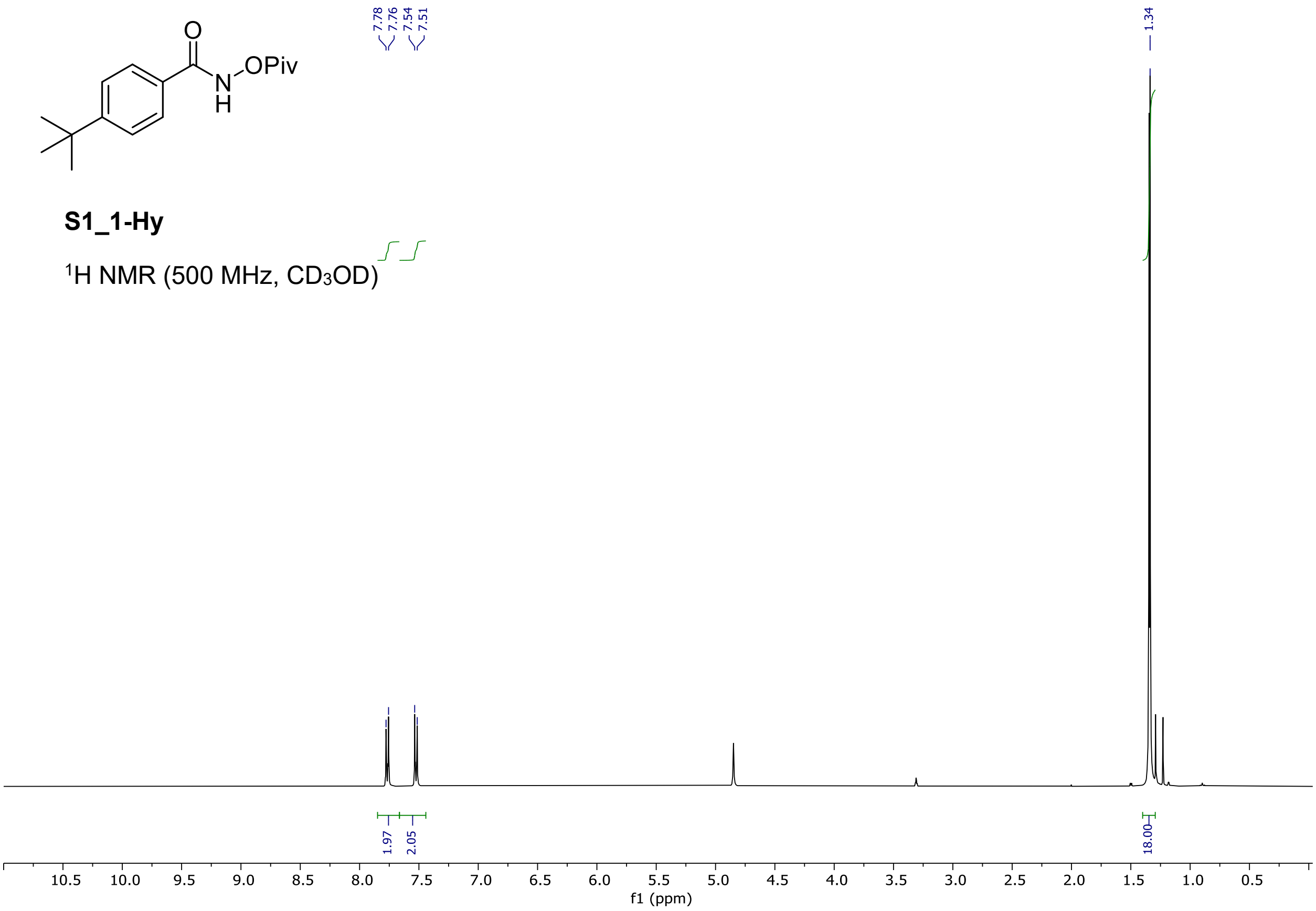
O
N
H
OPiv
S1_1-Hy
¹H NMR (500 MHz, CD₃OD)
7.78
7.76
7.54
7.51
1.34
1.97
2.05
18.00
f1 (ppm)

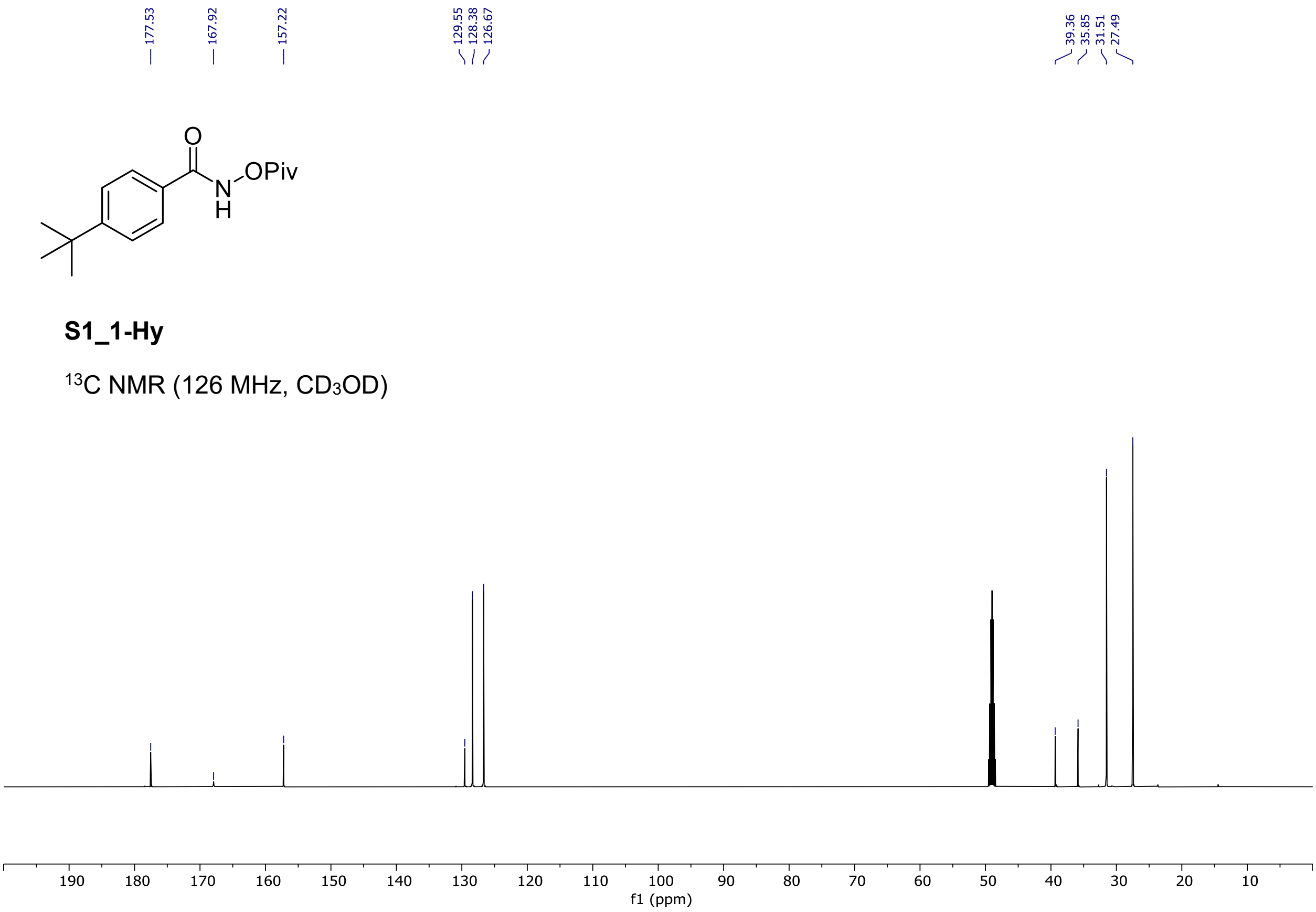
177.53
167.92
157.22
129.55
128.38
126.67
39.36
35.85
31.51
27.49
O
N
H
OPiv
S1_1-Hy
¹³C NMR (126 MHz, CD₃OD)
f1 (ppm)

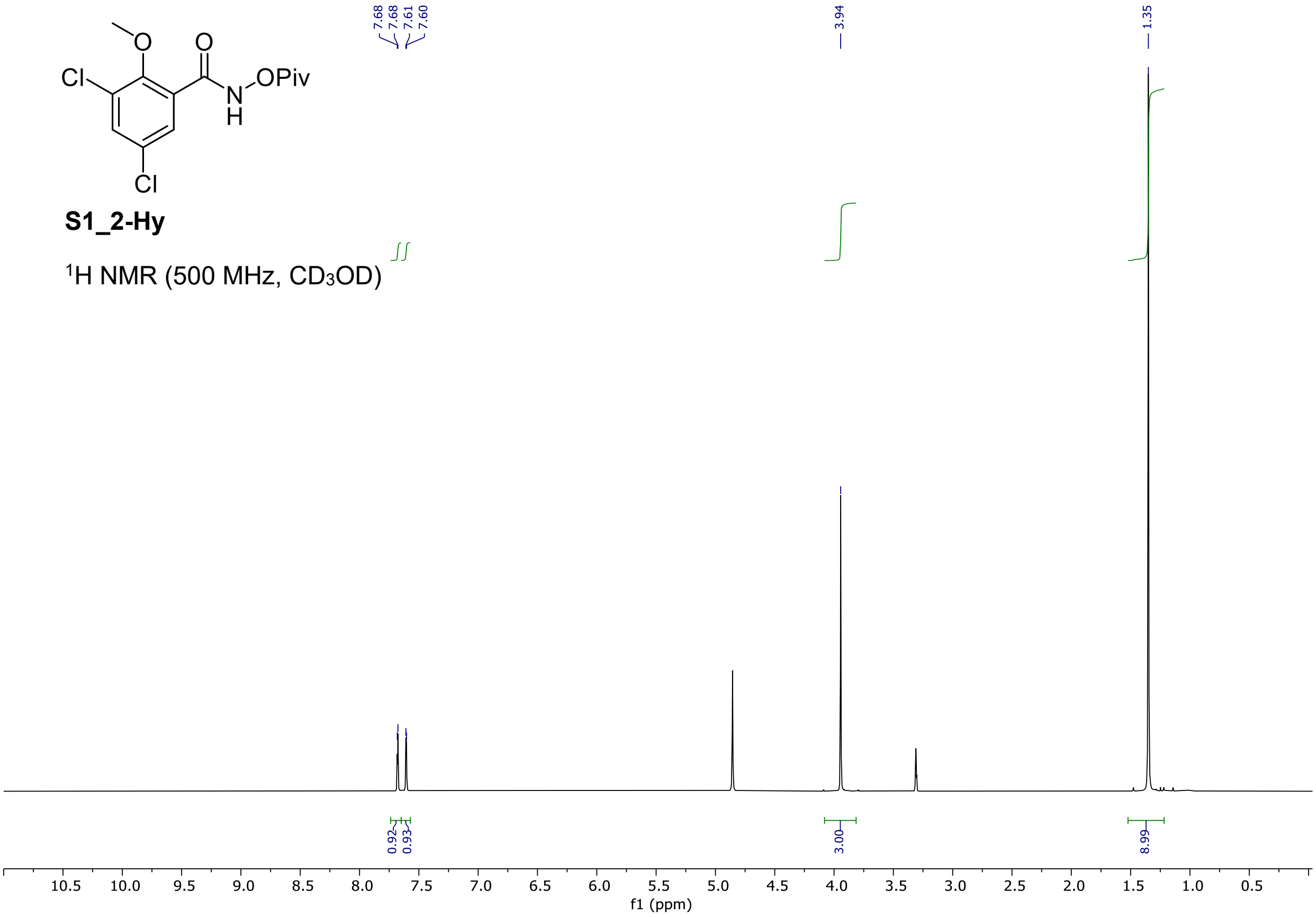
7.68
7.68
7.61
7.60
3.94
1.35
Cl
O
O
N
H
OPiv
Cl
S1_2-Hy
1H NMR (500 MHz, CD3OD)
0.92
0.93
3.00
8.99
10.5 10.0 9.5 9.0 8.5 8.0 7.5 7.0 6.5 6.0 5.5 5.0 4.5 4.0 3.5 3.0 2.5 2.0 1.5 1.0 0.5
f1 (ppm)

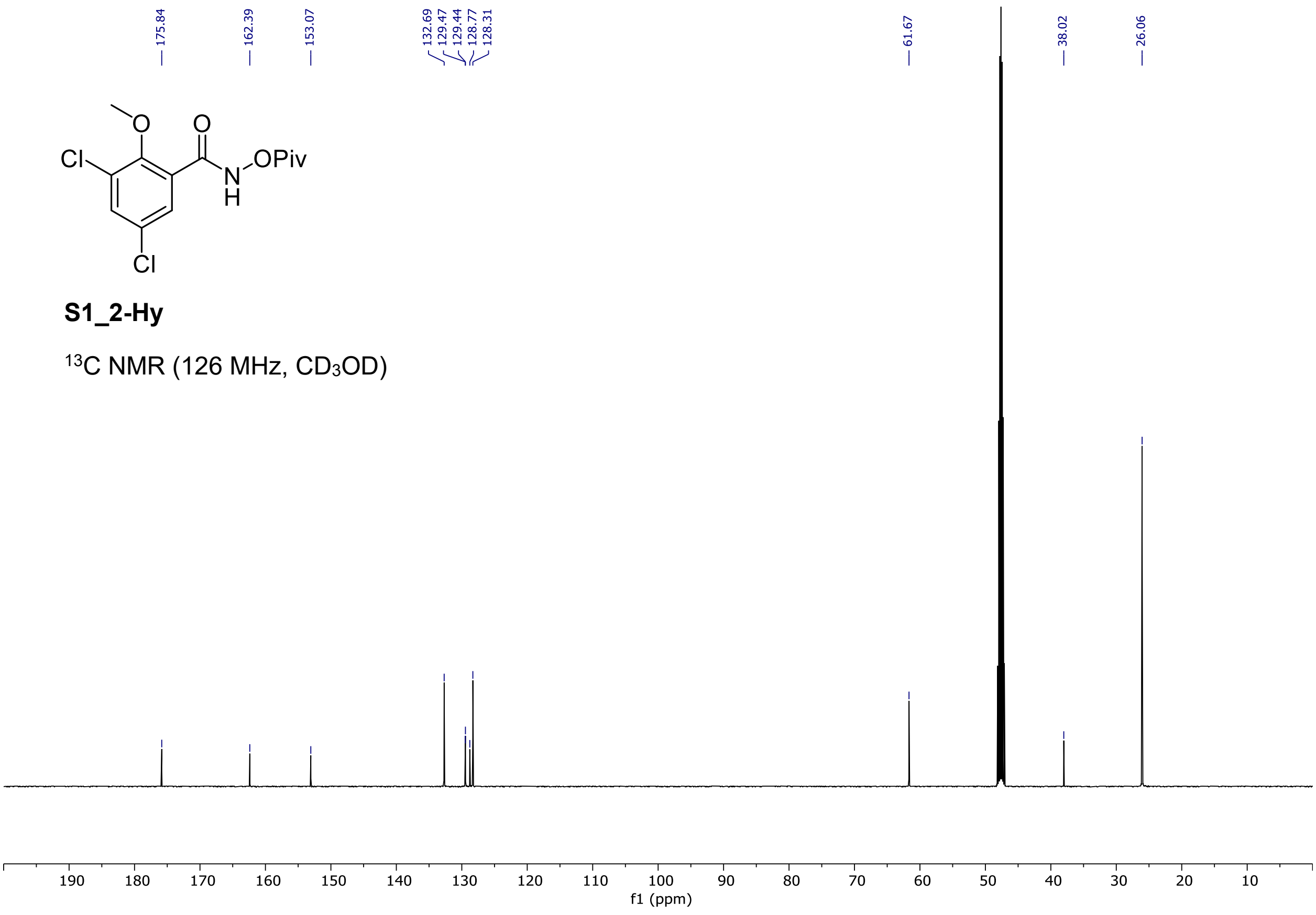
175.84
162.39
153.07
132.69
129.47
129.44
128.77
128.31
61.67
38.02
26.06
Cl
O
O
N
H
OPiv
Cl
S1_2-Hy
13C NMR (126 MHz, CD3OD)
190 180 170 160 150 140 130 120 110 100 90 80 70 60 50 40 30 20 10
f1 (ppm)

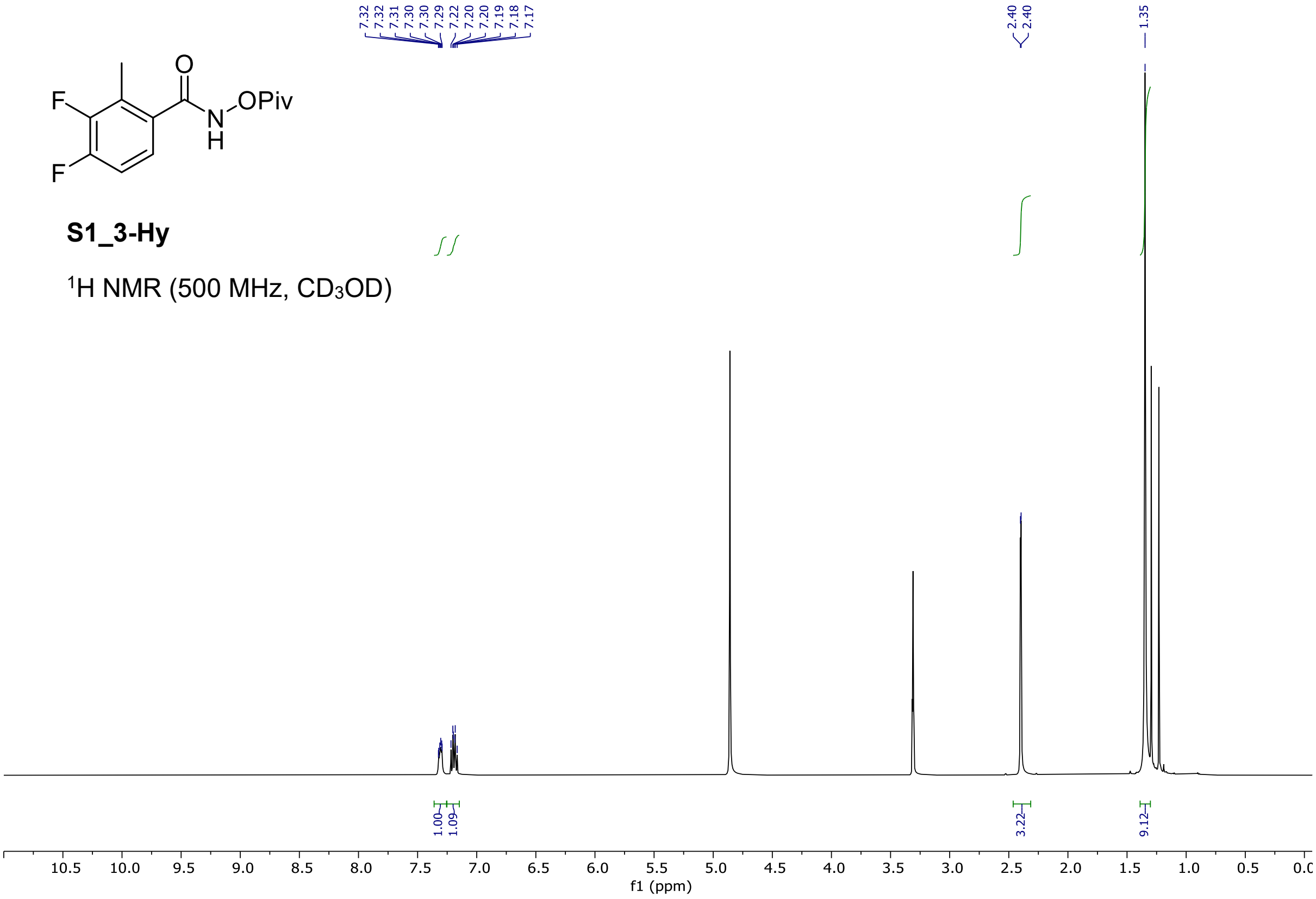

S1_3-Hy
^{1}H NMR (500 MHz, CD$_3$OD)
f1 (ppm)

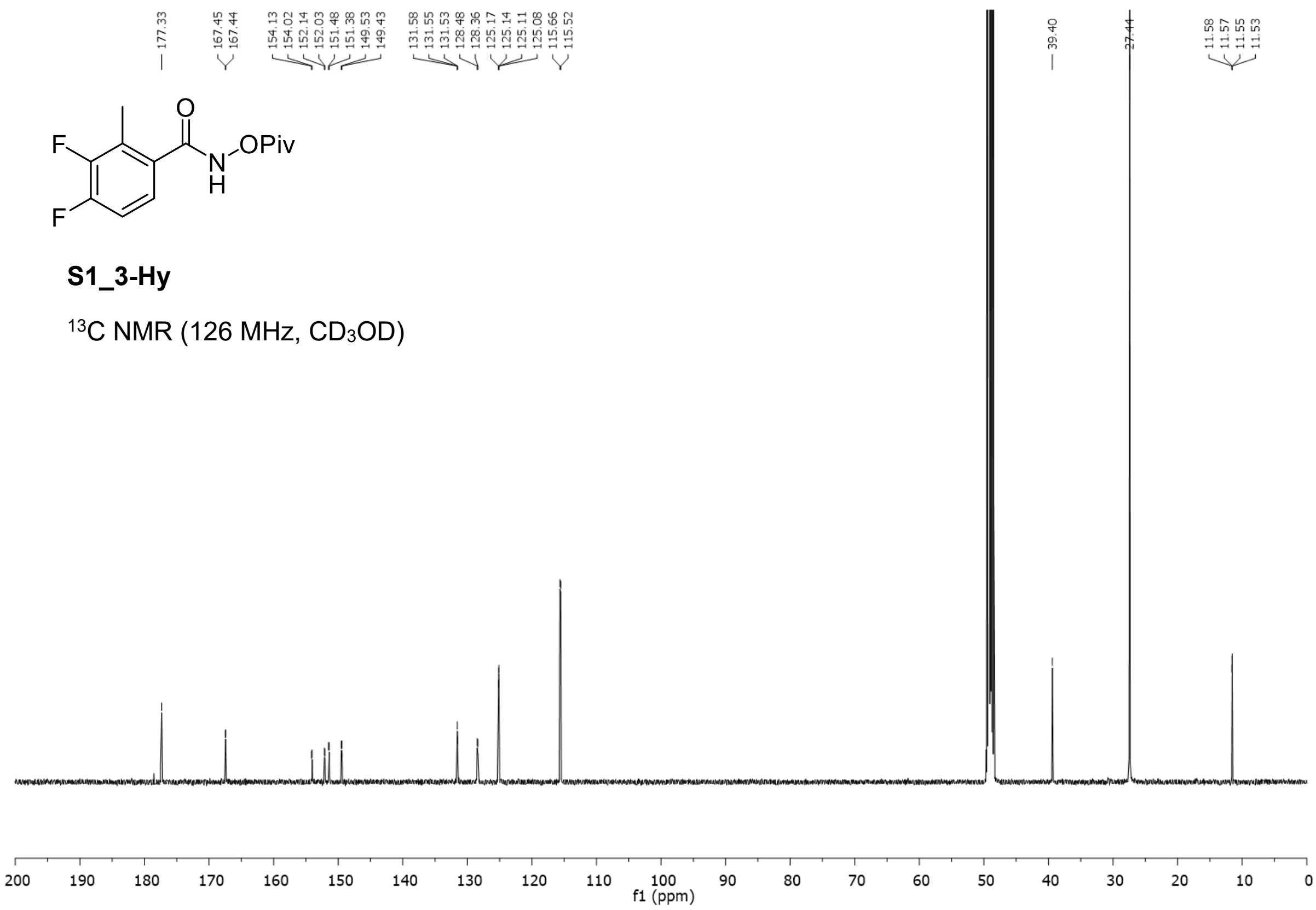

S1_3-Hy
^{13}C NMR (126 MHz, CD$_3$OD)
f1 (ppm)

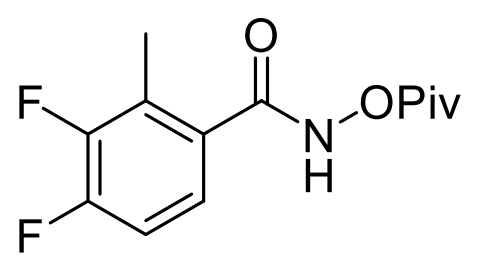

S1_3-Hy

^{19}F NMR (471 MHz, CD_3OD)

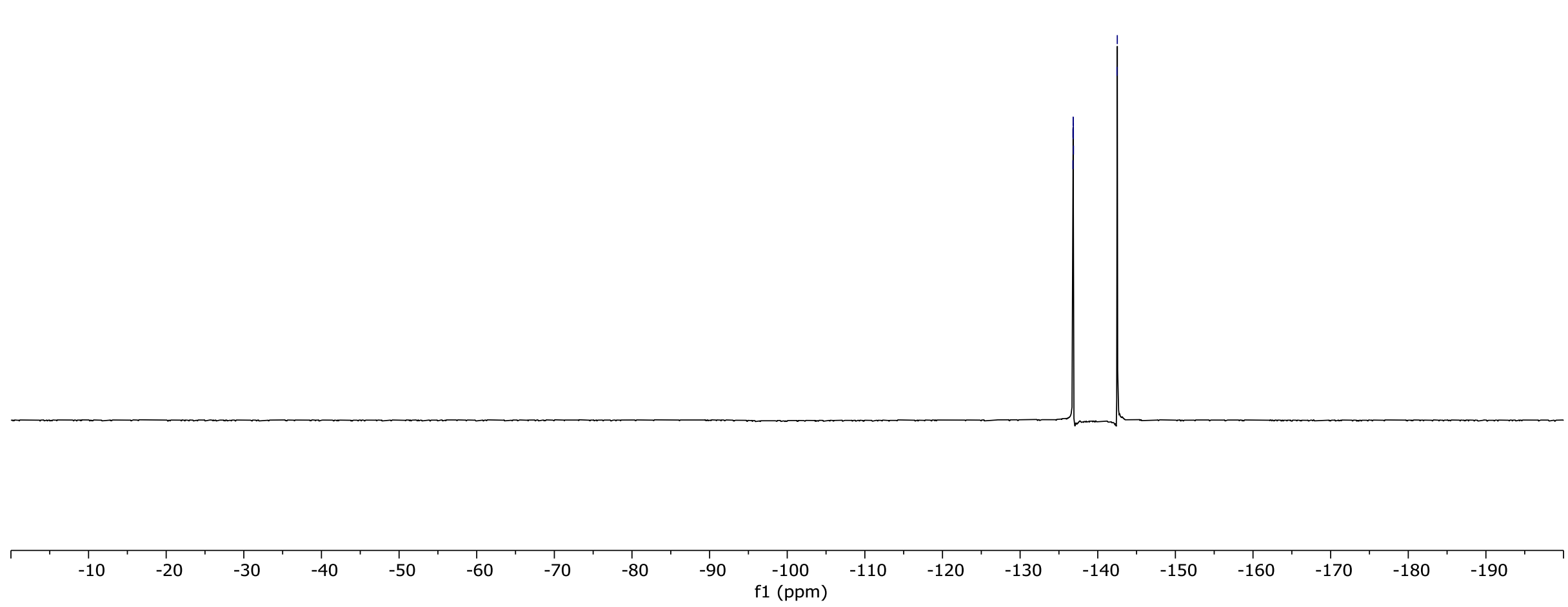

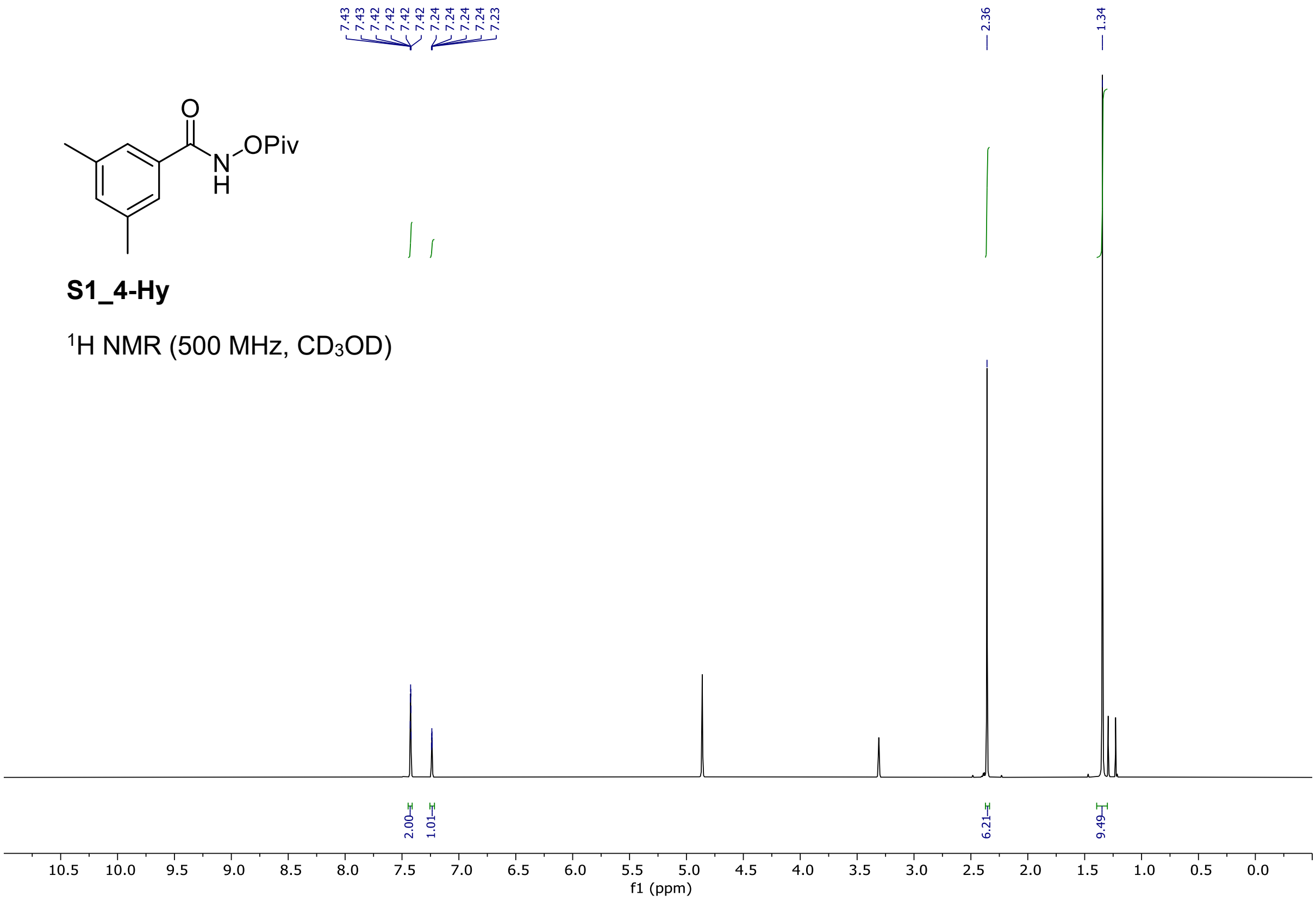

S1_4-Hy

^{1}H NMR (500 MHz, CD_3OD)

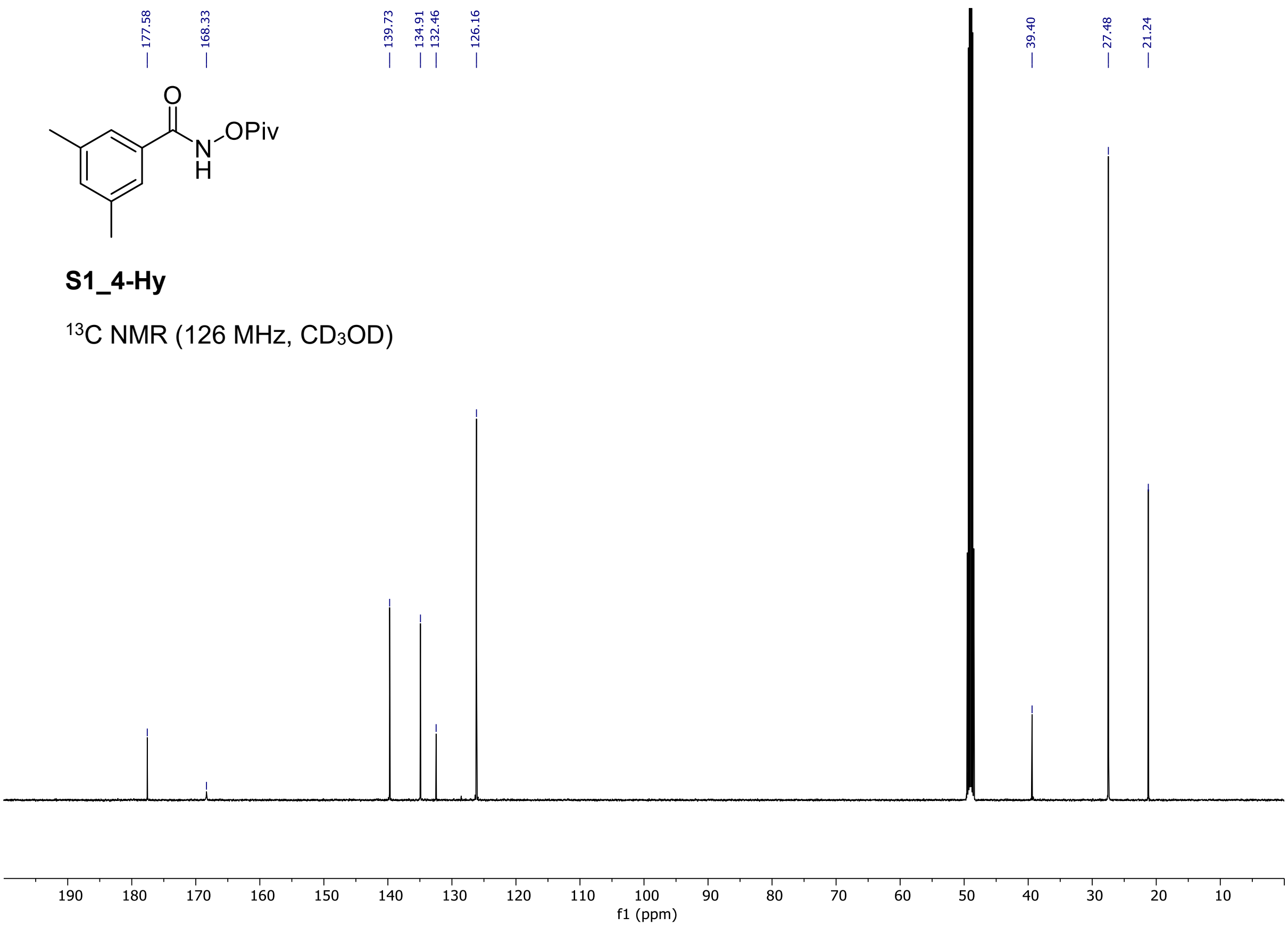

S1_4-Hy

^{13}C NMR (126 MHz, CD_3OD)

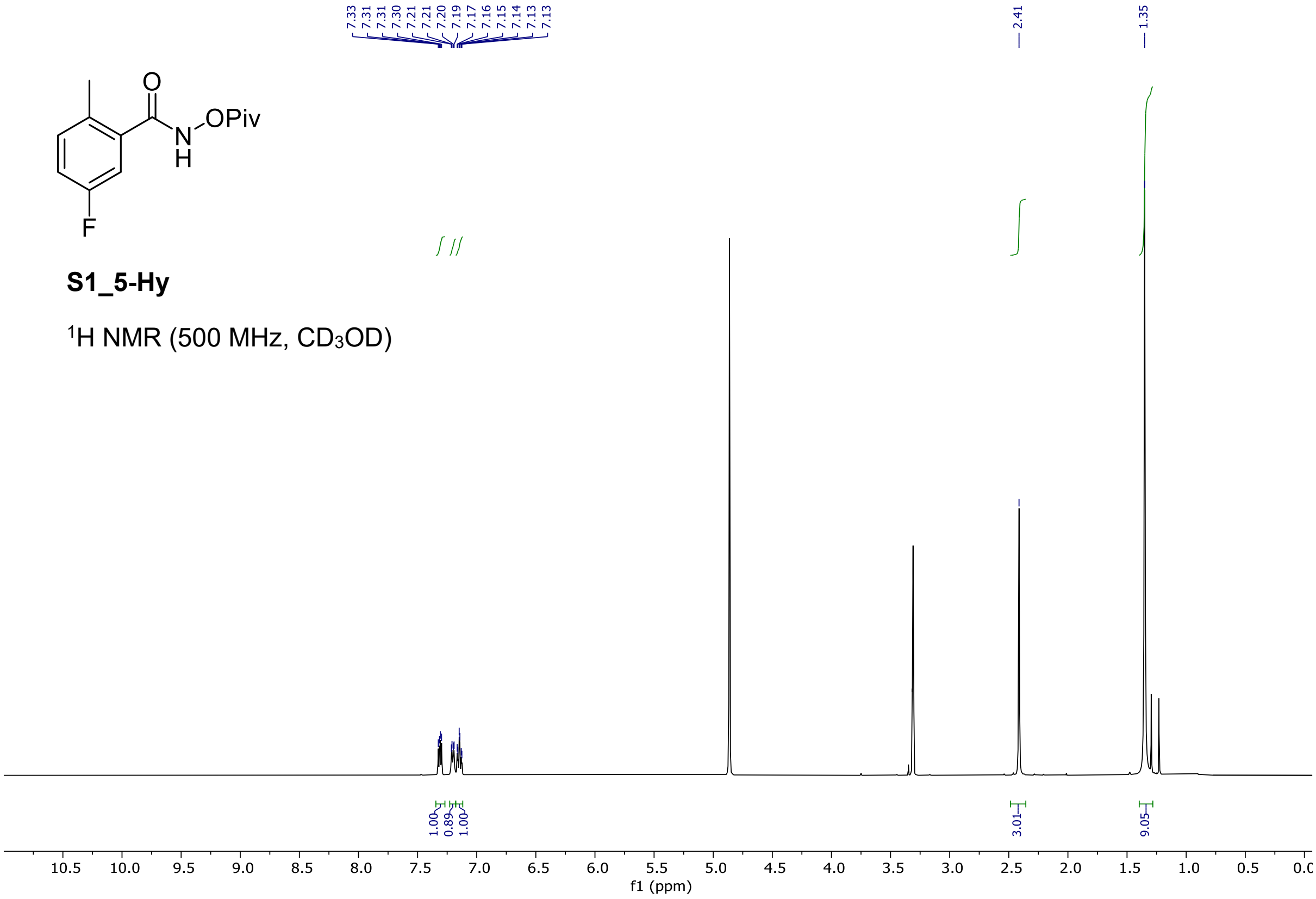

S1_5-Hy

^{1}H NMR (500 MHz, CD_3OD)

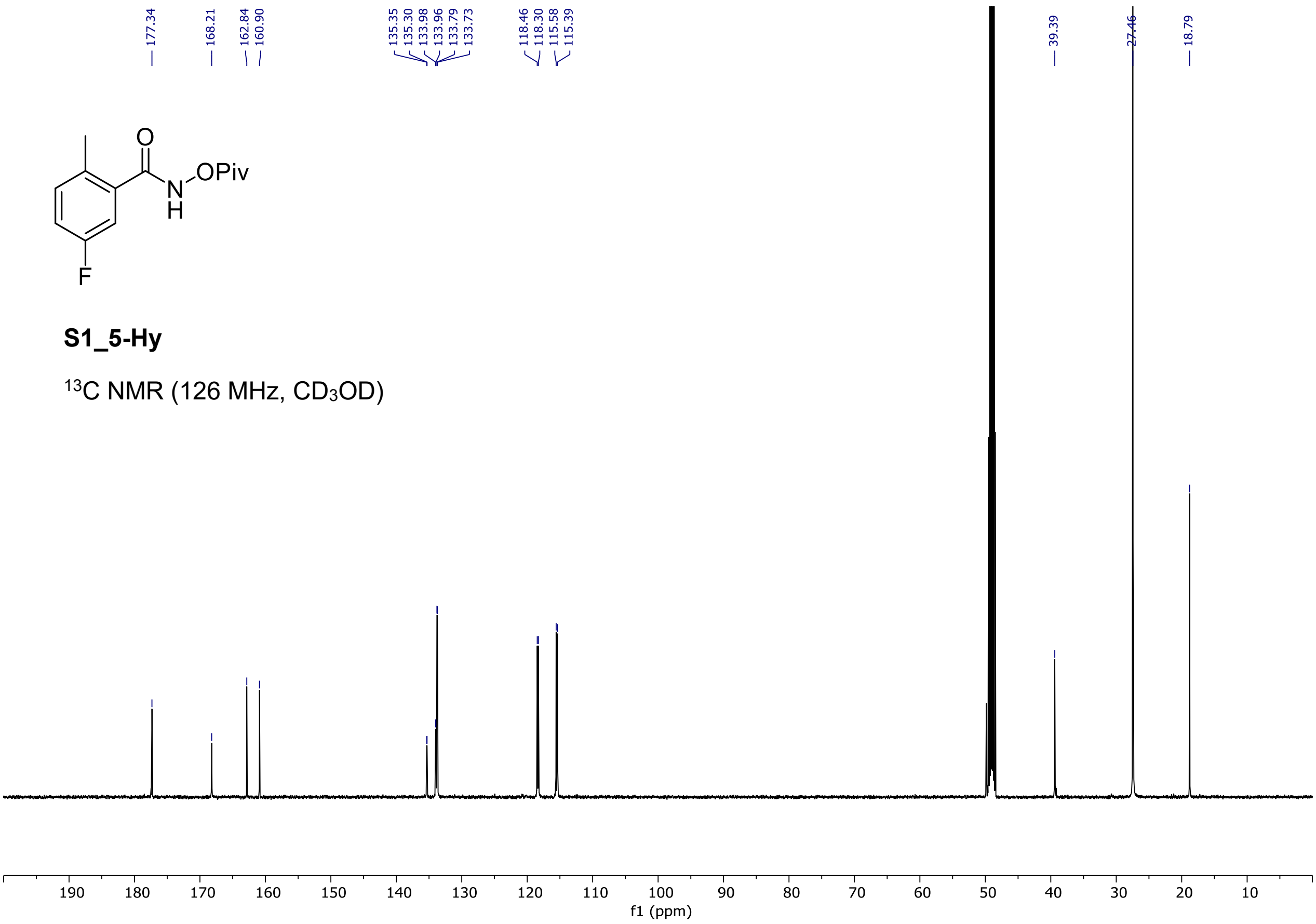

S1_5-Hy

^{13}C NMR (126 MHz, CD_3OD)

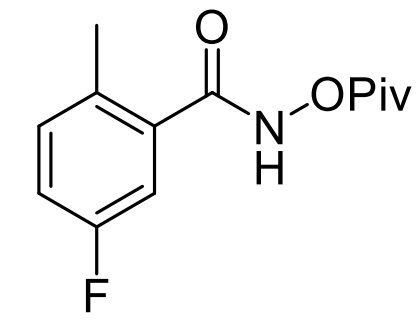

S1_5-Hy

^{19}F NMR (471 MHz, CD_3OD)

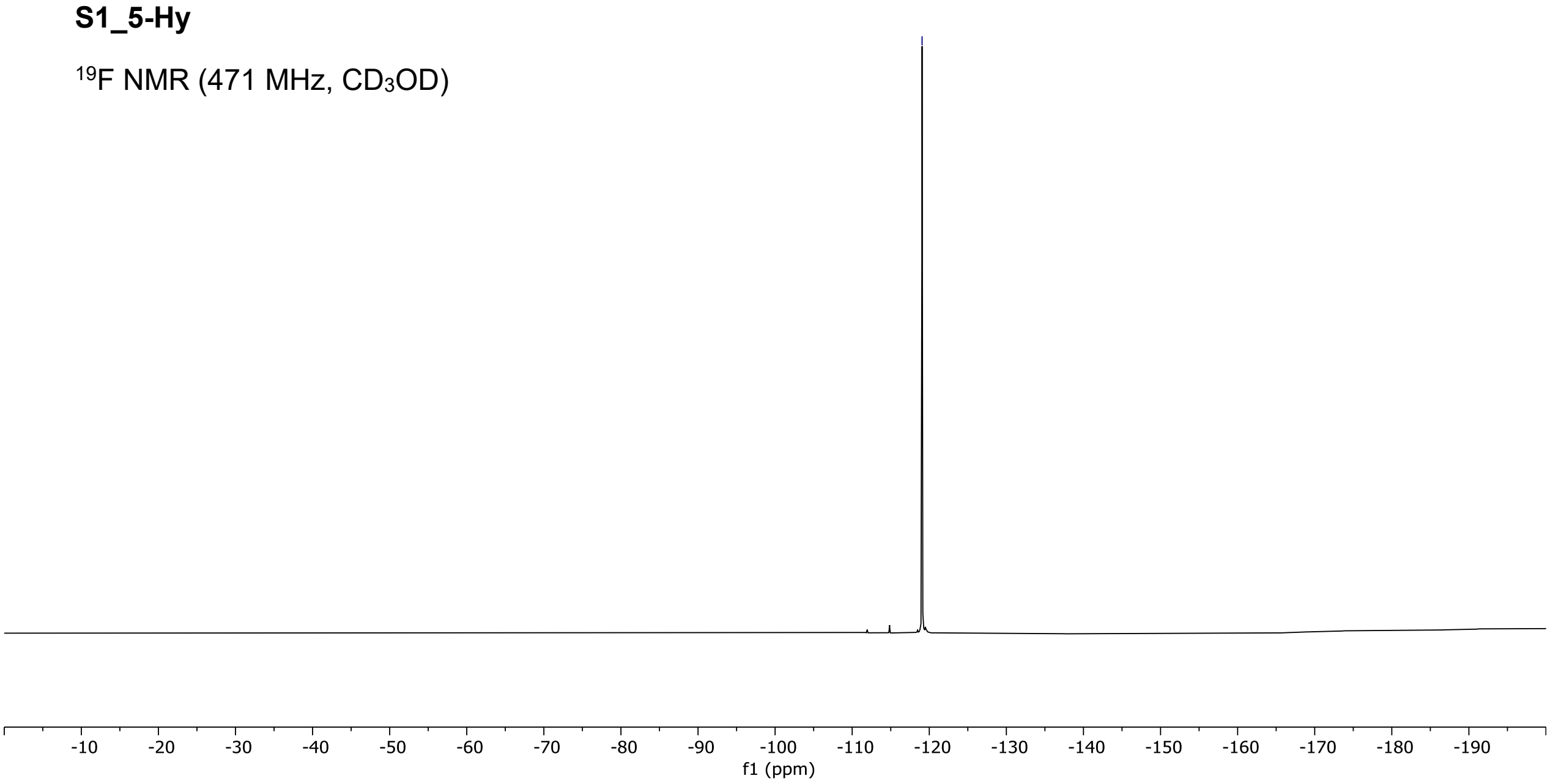

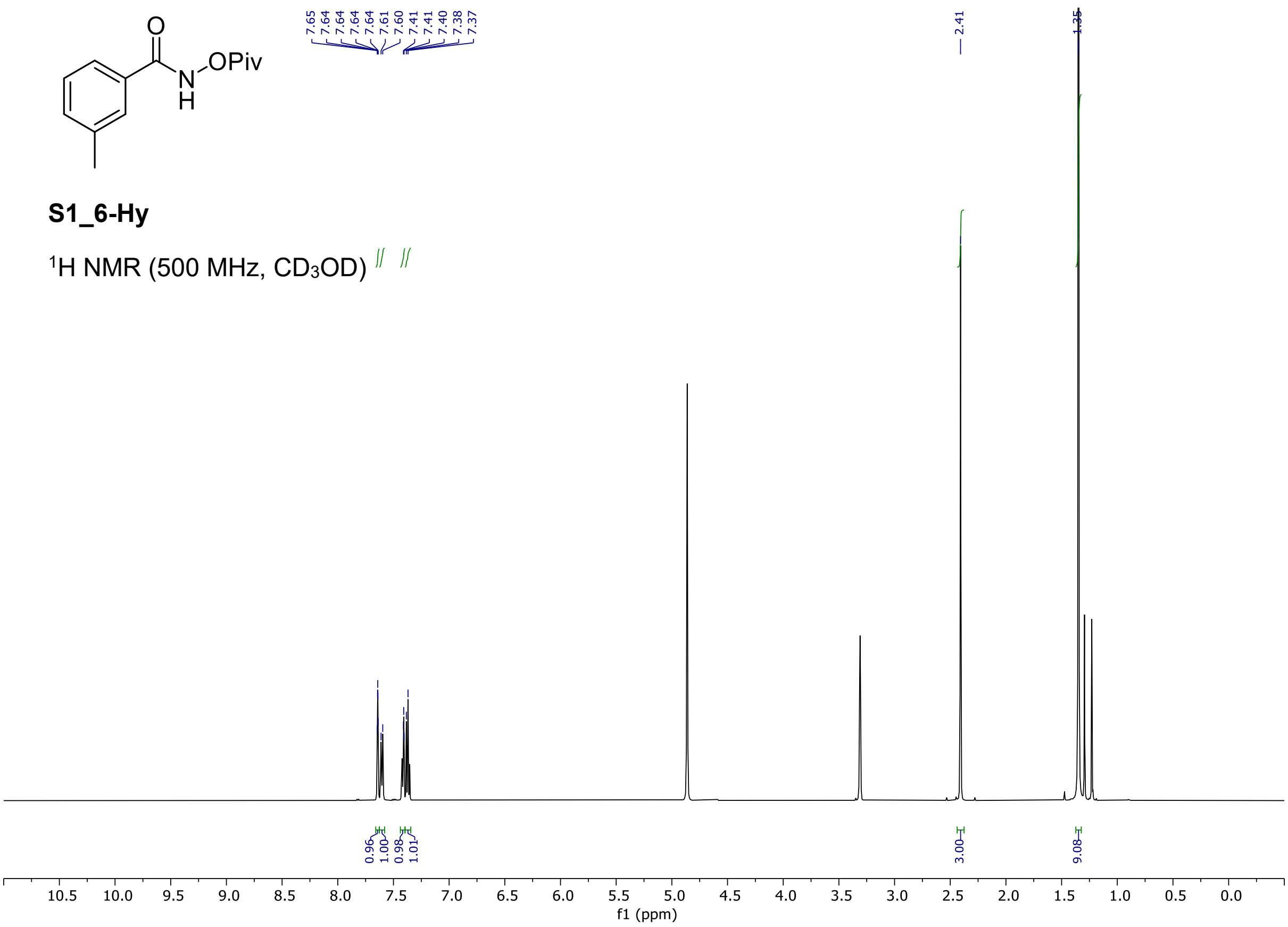
OPiv
N
H
O
S1_6-Hy
1H NMR (500 MHz, CD3OD)
7.65 7.64 7.64 7.64 7.64 7.61 7.60 7.41 7.41 7.40 7.38 7.37
2.41
1.35
0.96 1.00 0.98 1.01
3.00
9.08
10.5 10.0 9.5 9.0 8.5 8.0 7.5 7.0 6.5 6.0 5.5 5.0 4.5 4.0 3.5 3.0 2.5 2.0 1.5 1.0 0.5 0.0
f1 (ppm)

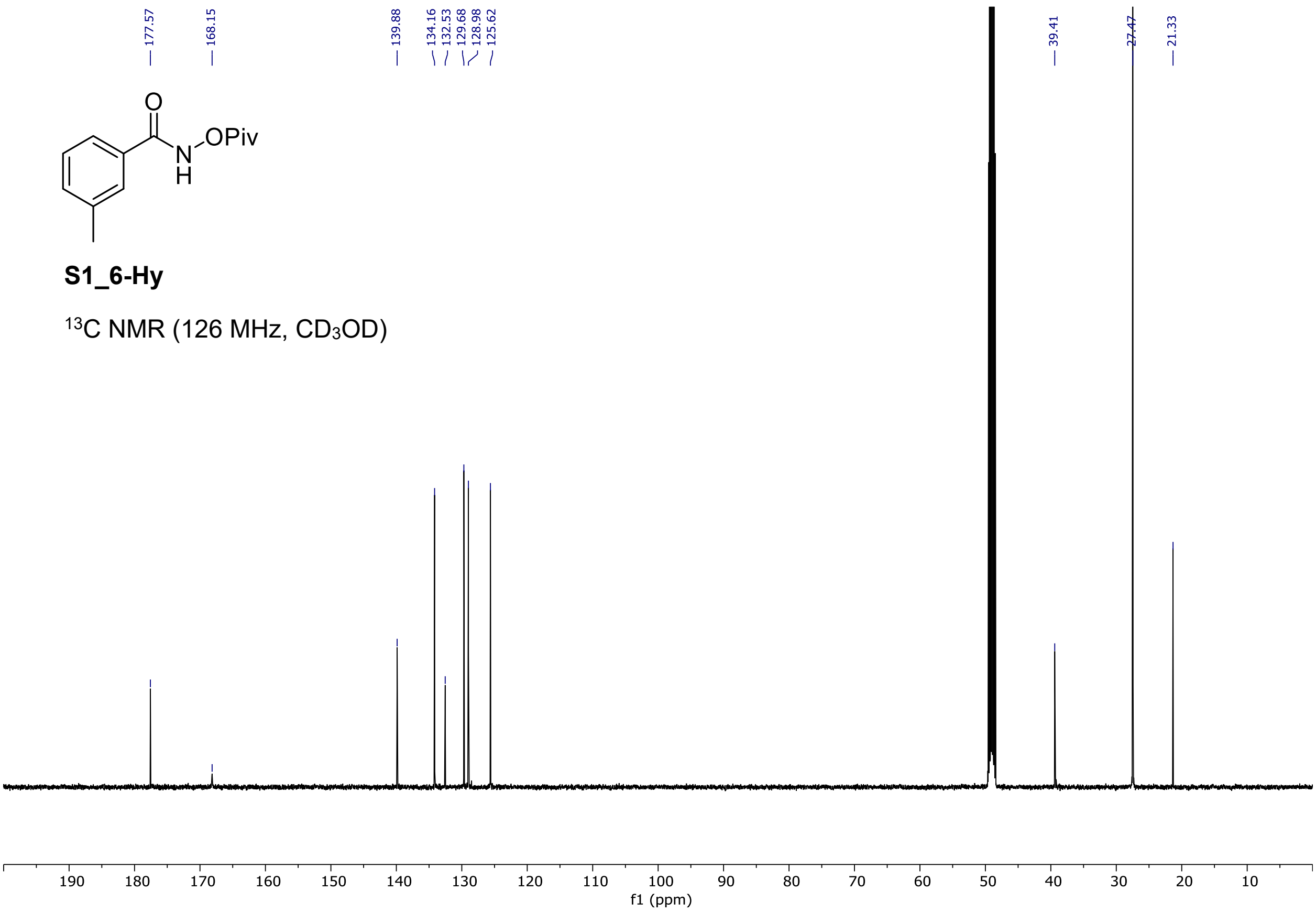
177.57
168.15
139.88
134.16 132.53 129.68 128.98 125.62
39.41
27.47
21.33
OPiv
N
H
O
S1_6-Hy
13C NMR (126 MHz, CD3OD)
190 180 170 160 150 140 130 120 110 100 90 80 70 60 50 40 30 20 10
f1 (ppm)

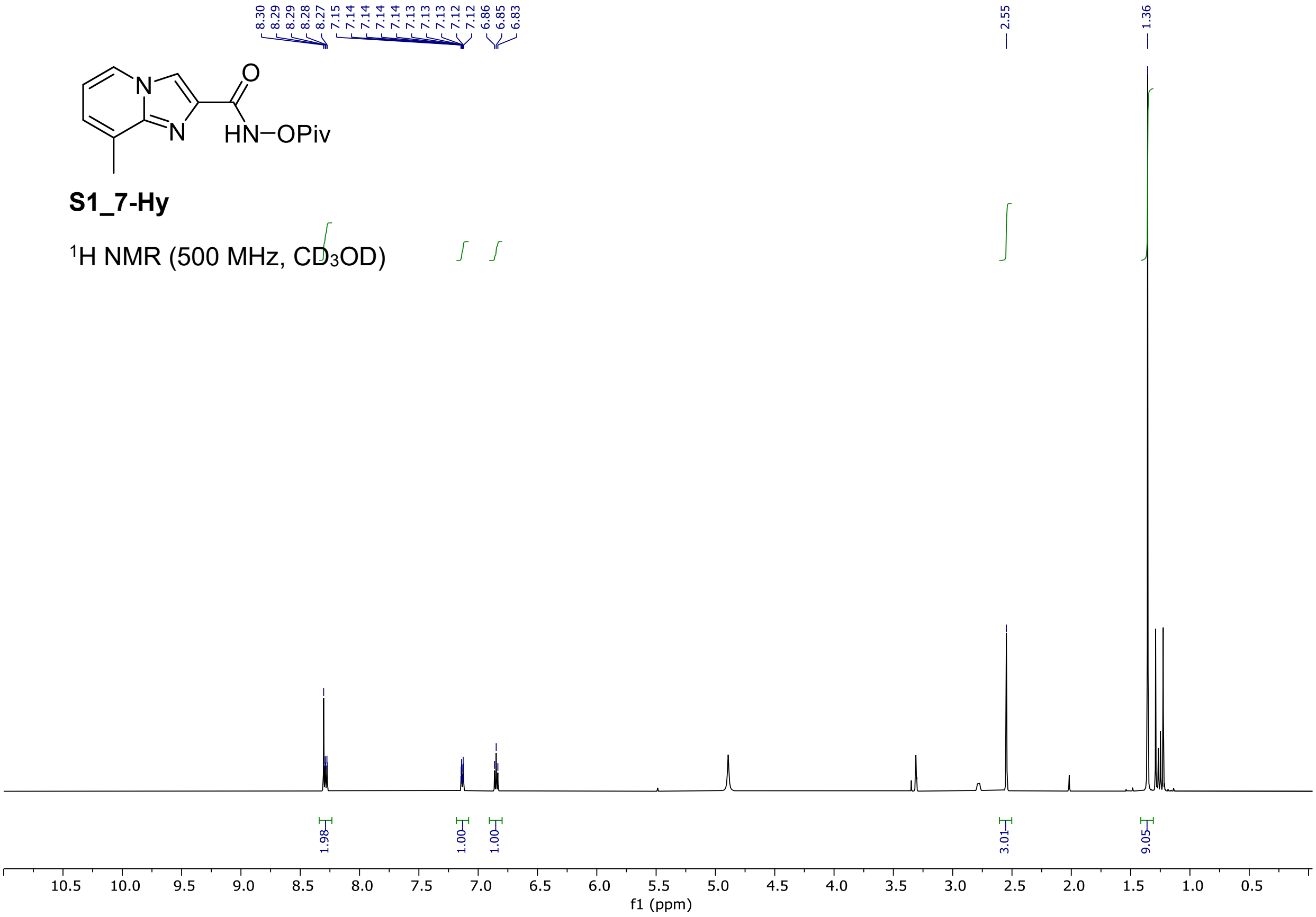

S1_7-Hy

^{1}H NMR (500 MHz, CD_3OD)

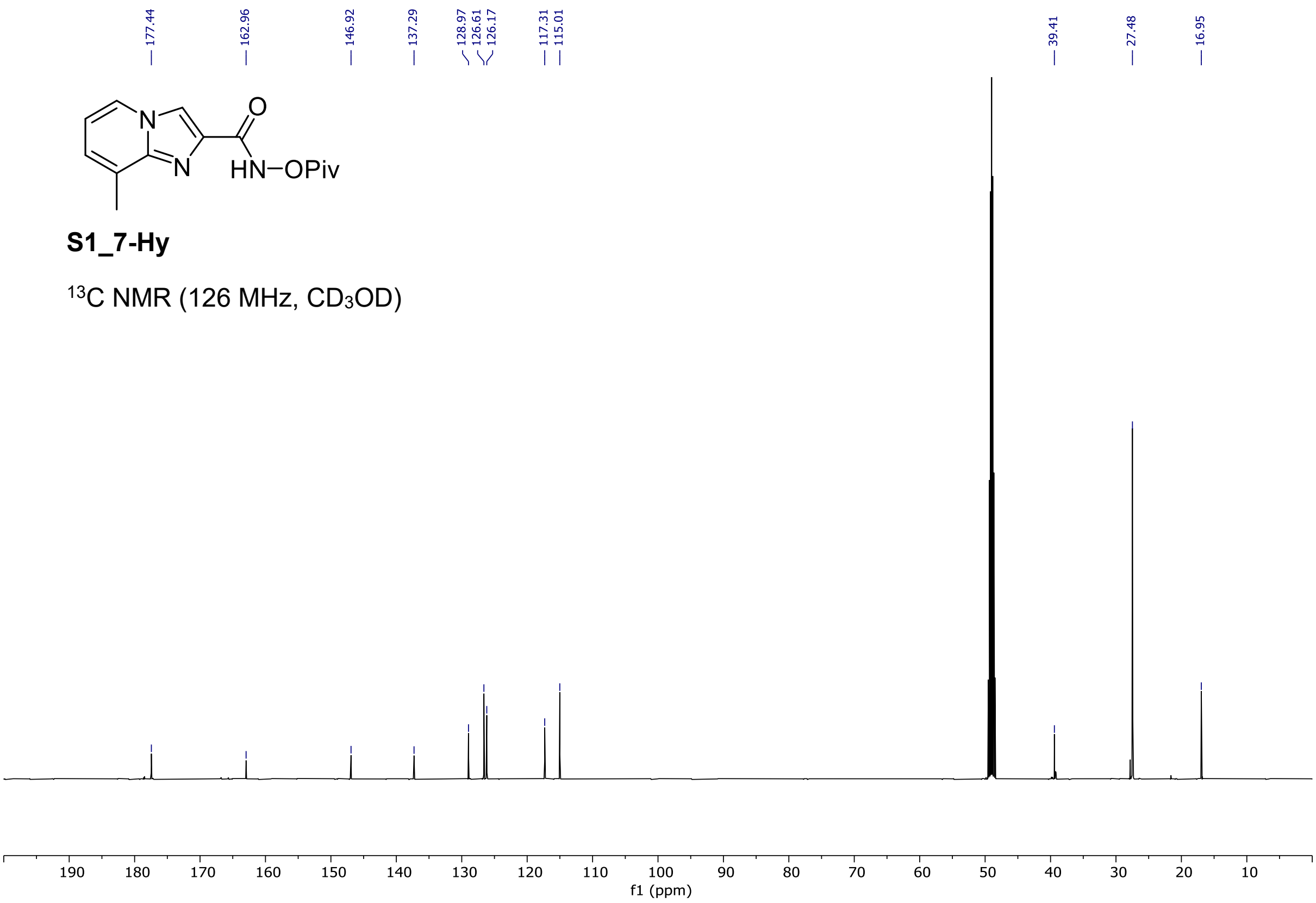

S1_7-Hy

^{13}C NMR (126 MHz, CD_3OD)

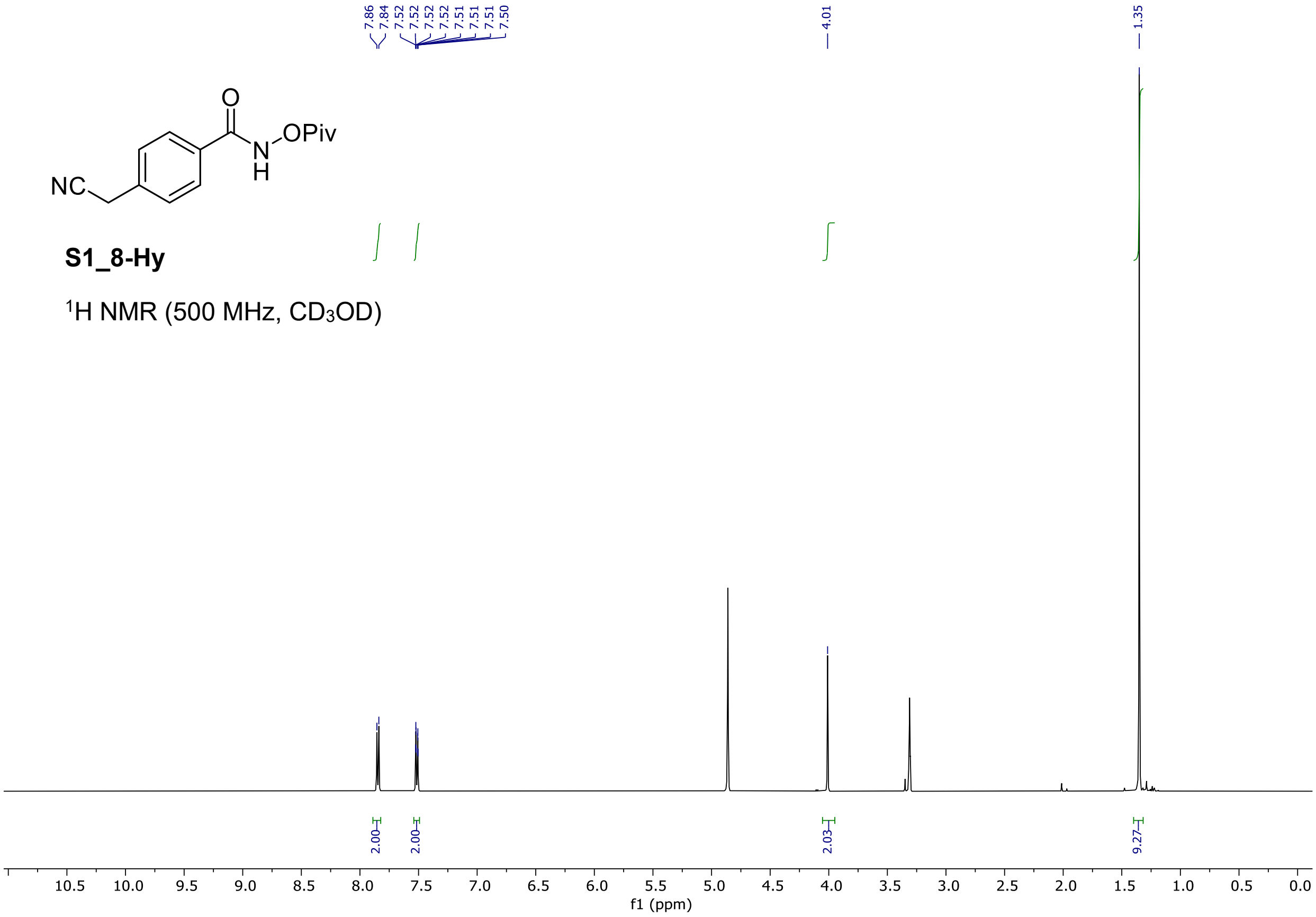

S1_8-Hy

^{1}H NMR (500 MHz, CD_3OD)

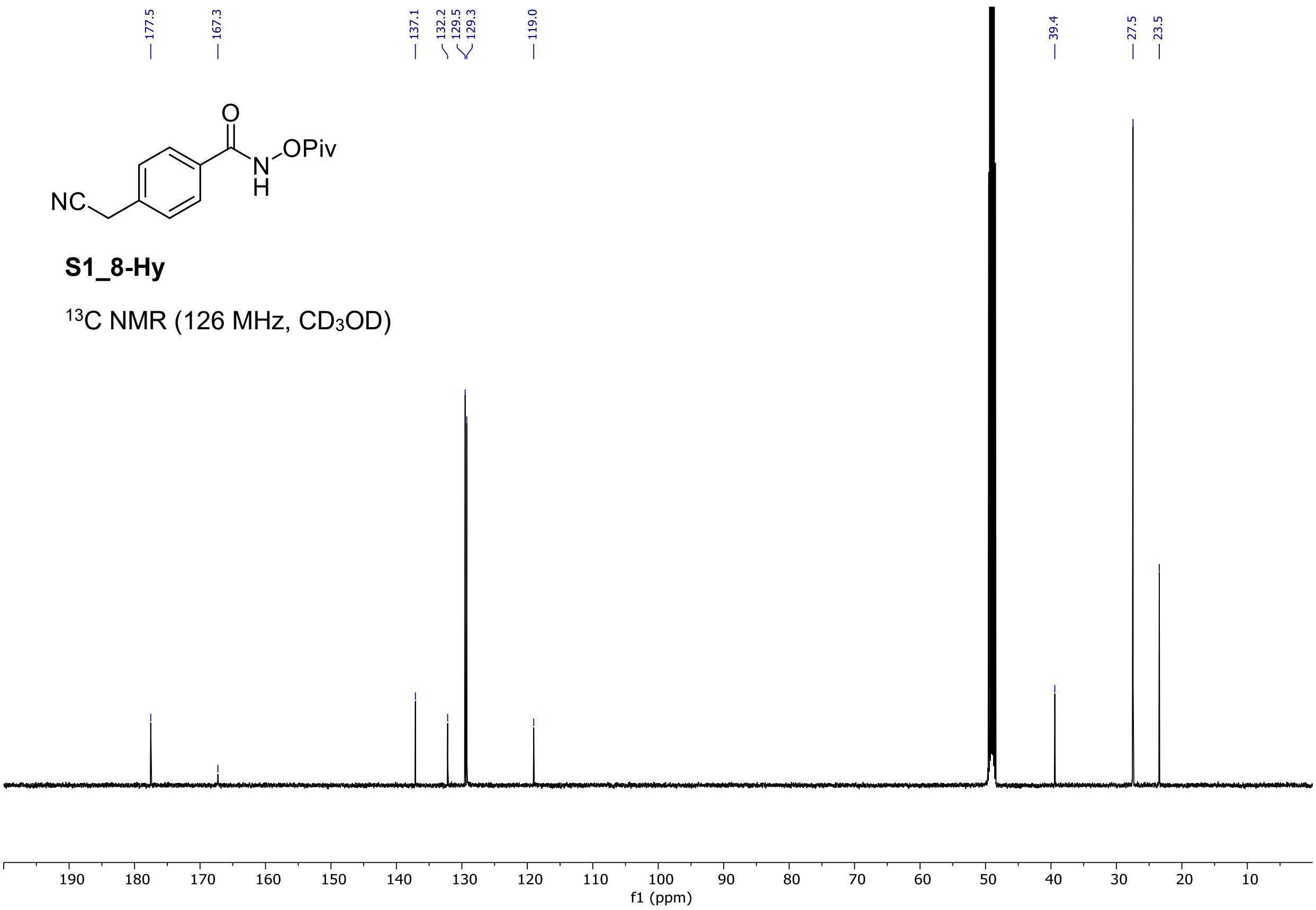

S1_8-Hy

^{13}C NMR (126 MHz, CD_3OD)

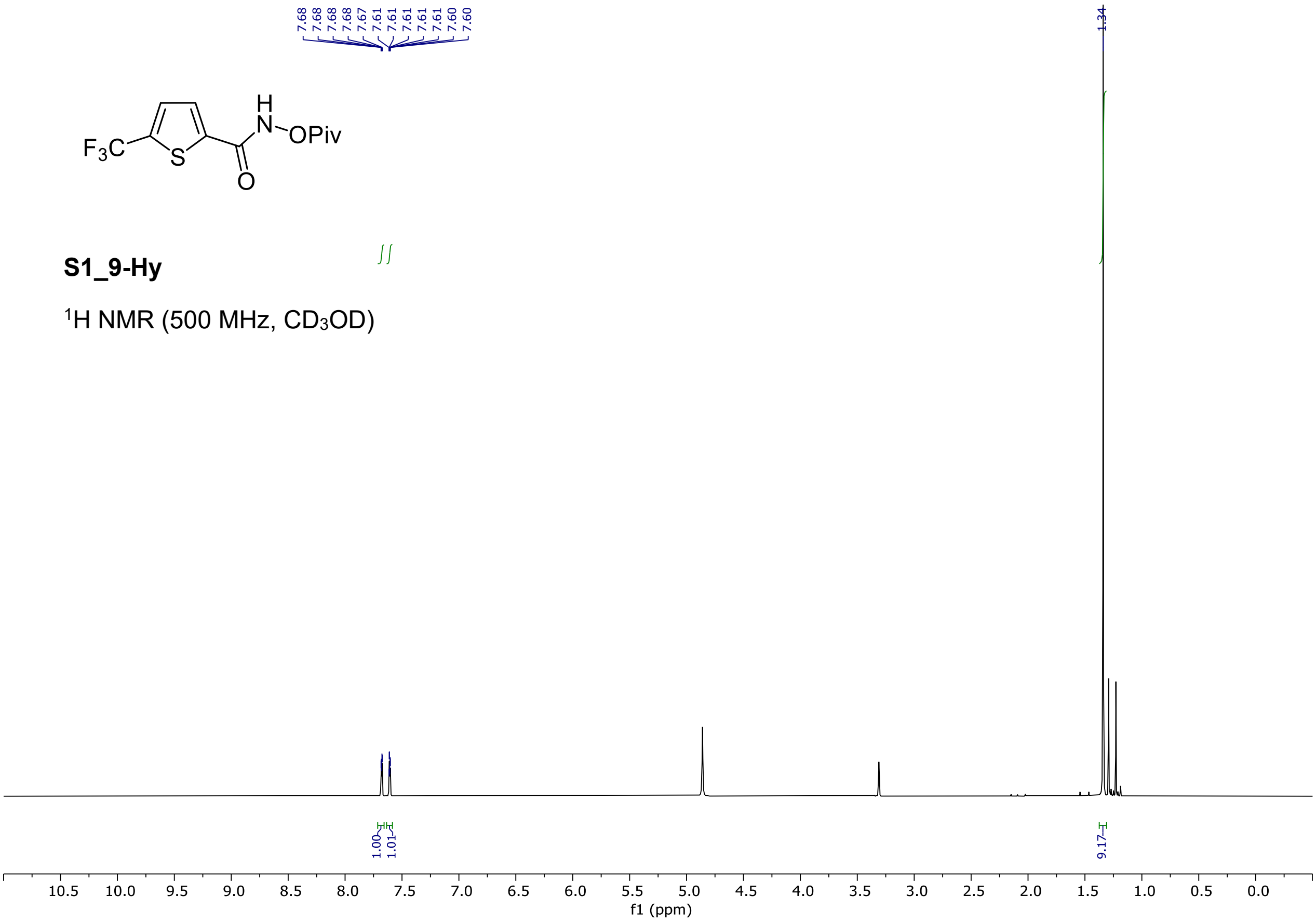
7.68 7.68 7.68 7.68 7.67 7.61 7.61 7.61 7.61 7.61 7.60 7.60
1.34
F3C
S
H
N
OPiv
O
S1_9-Hy
1H NMR (500 MHz, CD3OD)
1.00
1.01
9.17
10.5 10.0 9.5 9.0 8.5 8.0 7.5 7.0 6.5 6.0 5.5 5.0 4.5 4.0 3.5 3.0 2.5 2.0 1.5 1.0 0.5 0.0
f1 (ppm)

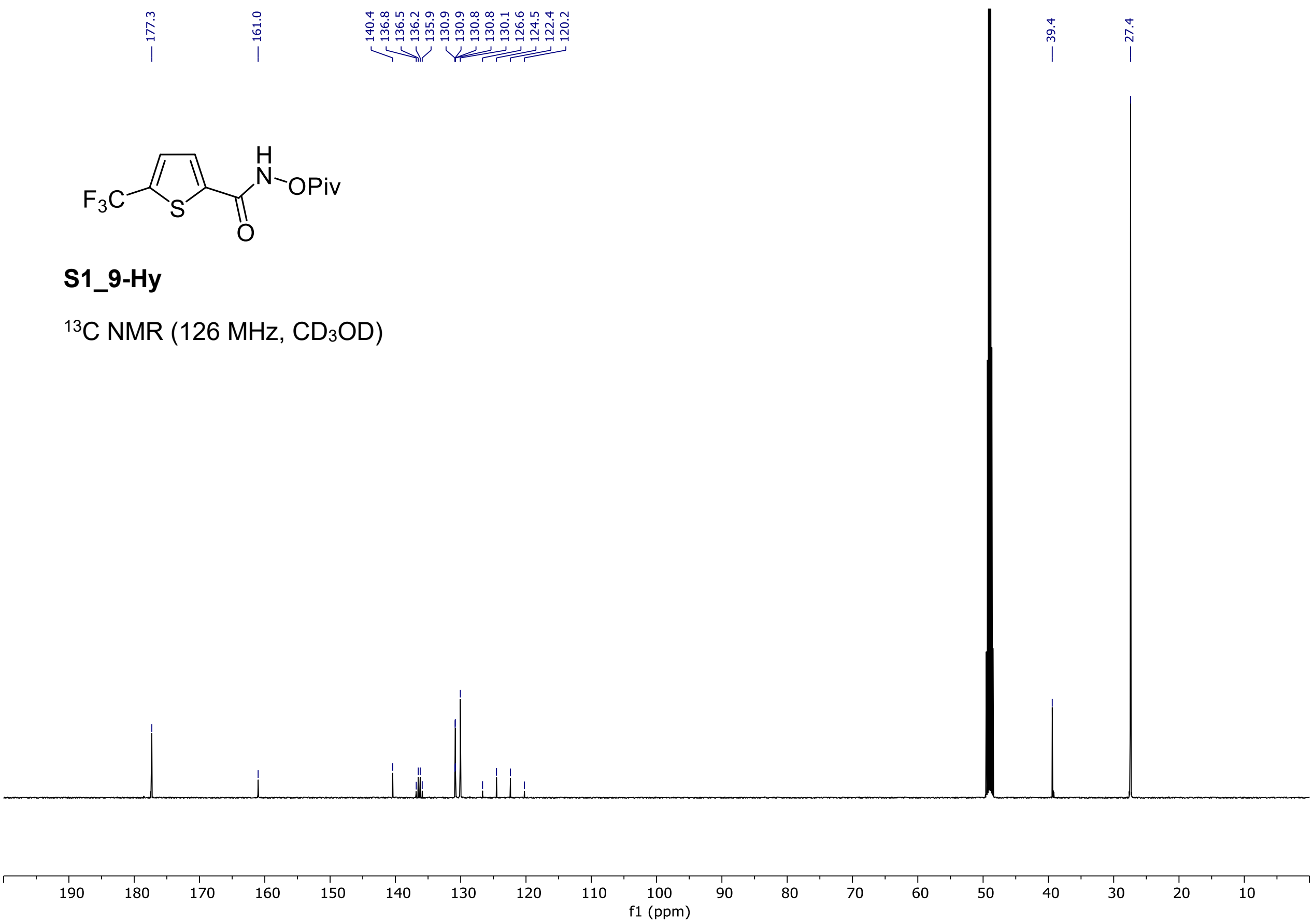
177.3
161.0
140.4 136.8 136.5 136.2 135.9 130.9 130.9 130.8 130.8 130.1 126.6 124.5 122.4 120.2
39.4
27.4
F3C
S
H
N
OPiv
O
S1_9-Hy
13C NMR (126 MHz, CD3OD)
190 180 170 160 150 140 130 120 110 100 90 80 70 60 50 40 30 20 10
f1 (ppm)

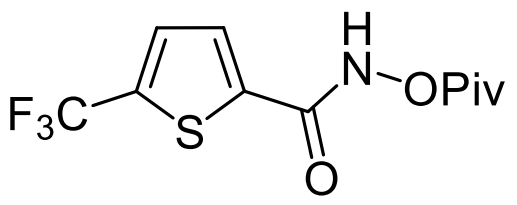

S1_9-Hy

^{19}F NMR (376 MHz, CD_3OD)

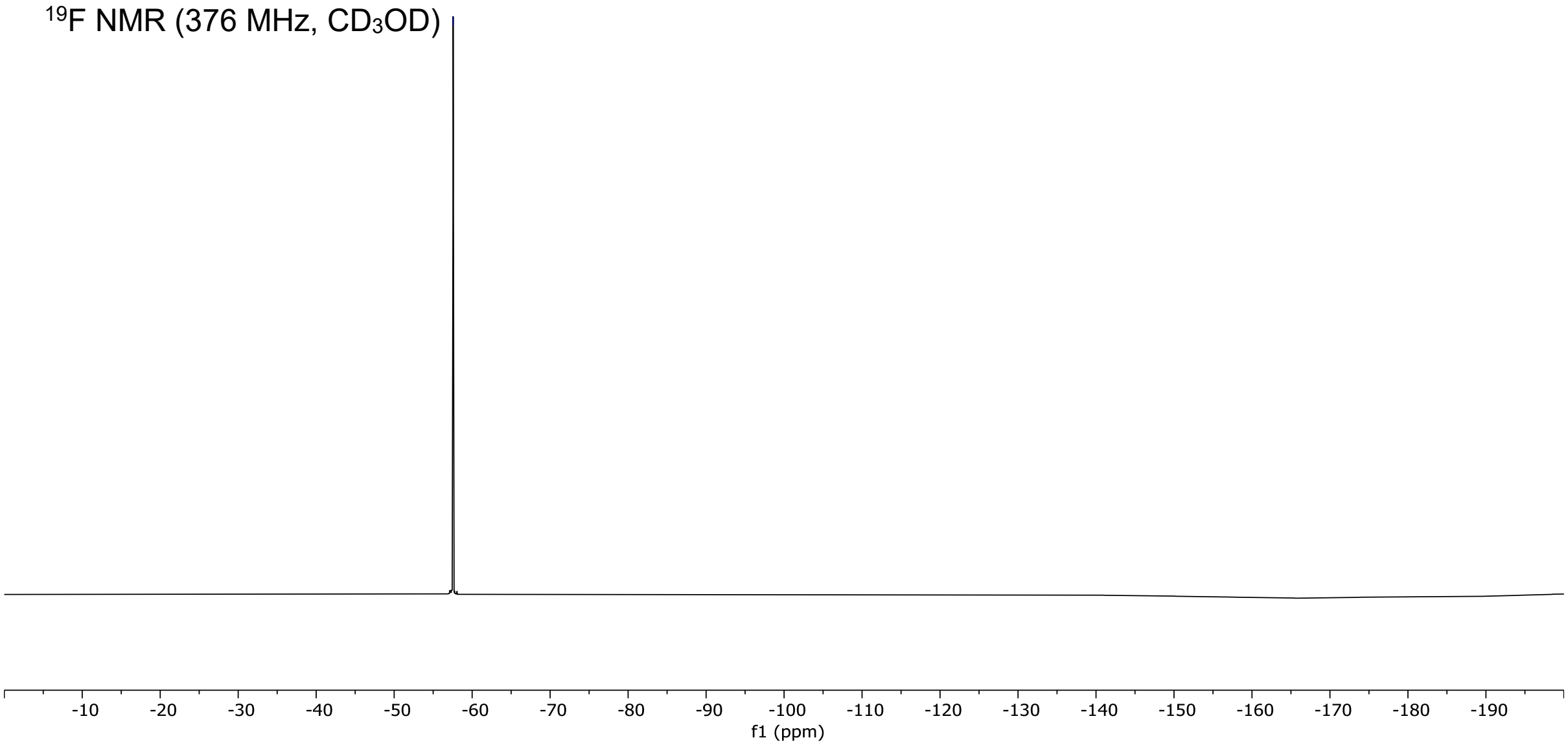

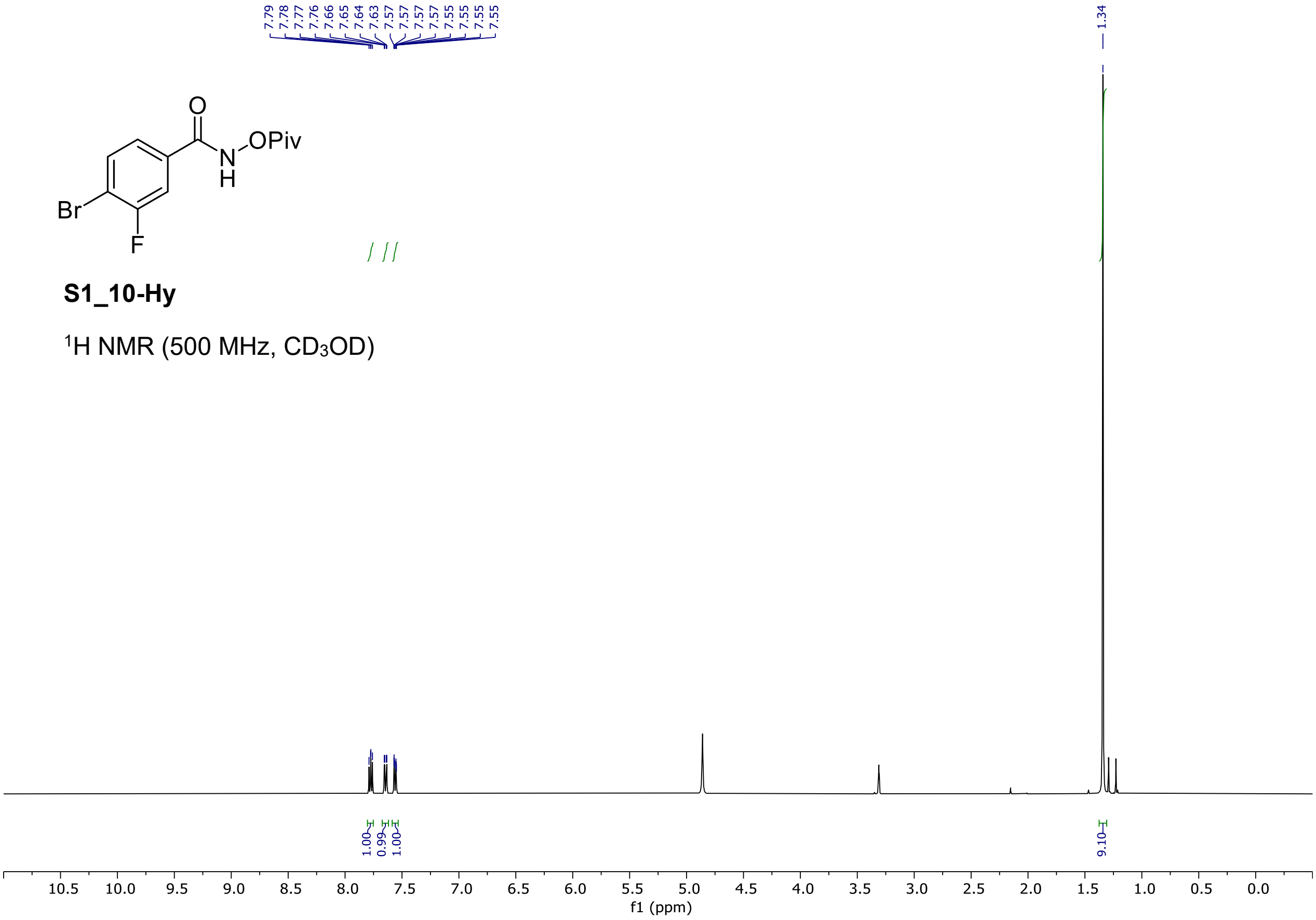

S1_10-Hy

^{1}H NMR (500 MHz, CD_3OD)

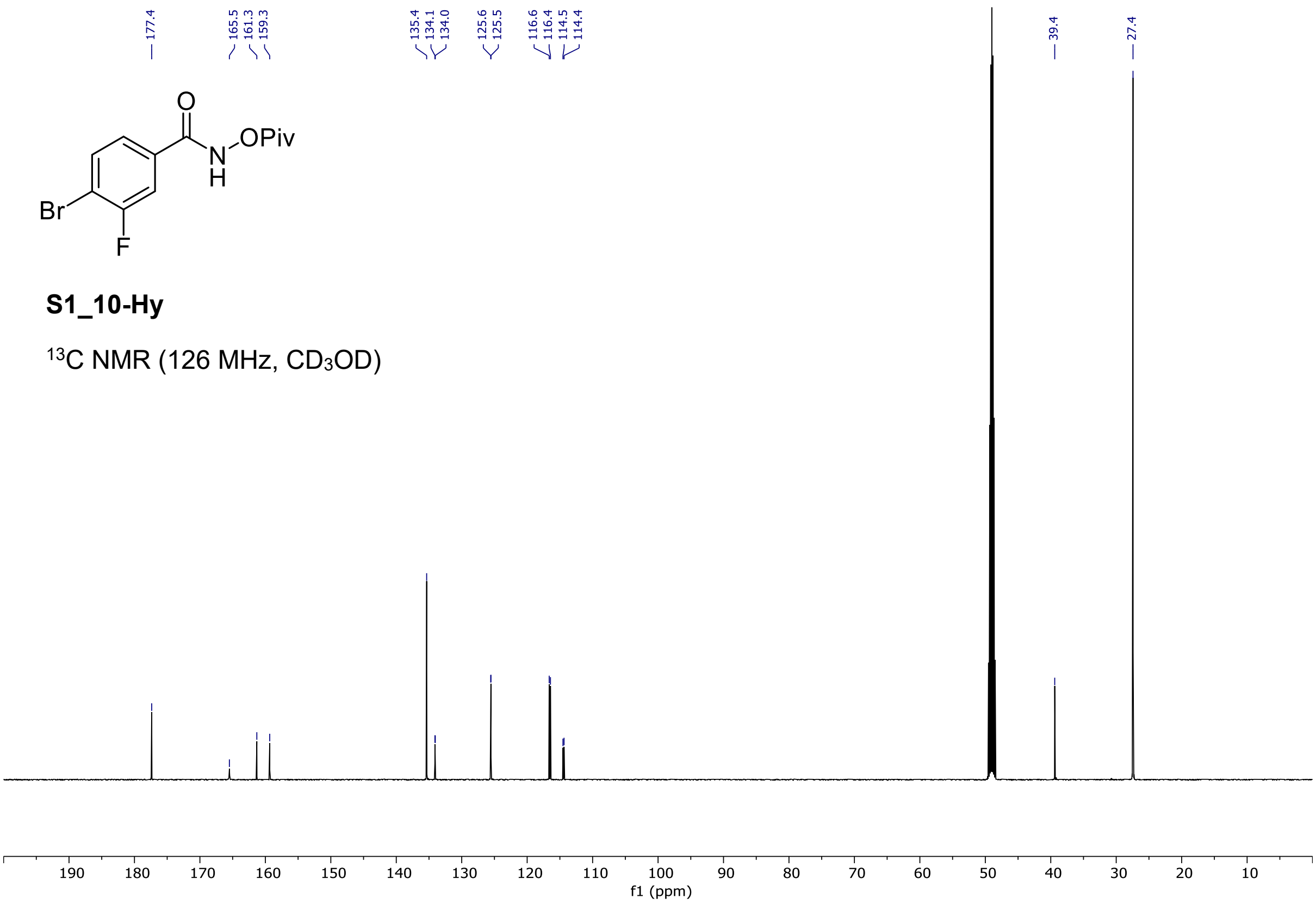

S1_10-Hy

^{13}C NMR (126 MHz, CD_3OD)

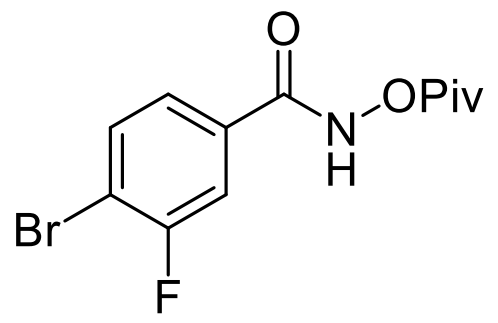

S1_10-Hy

^{19}F NMR (376 MHz, CD_3OD)

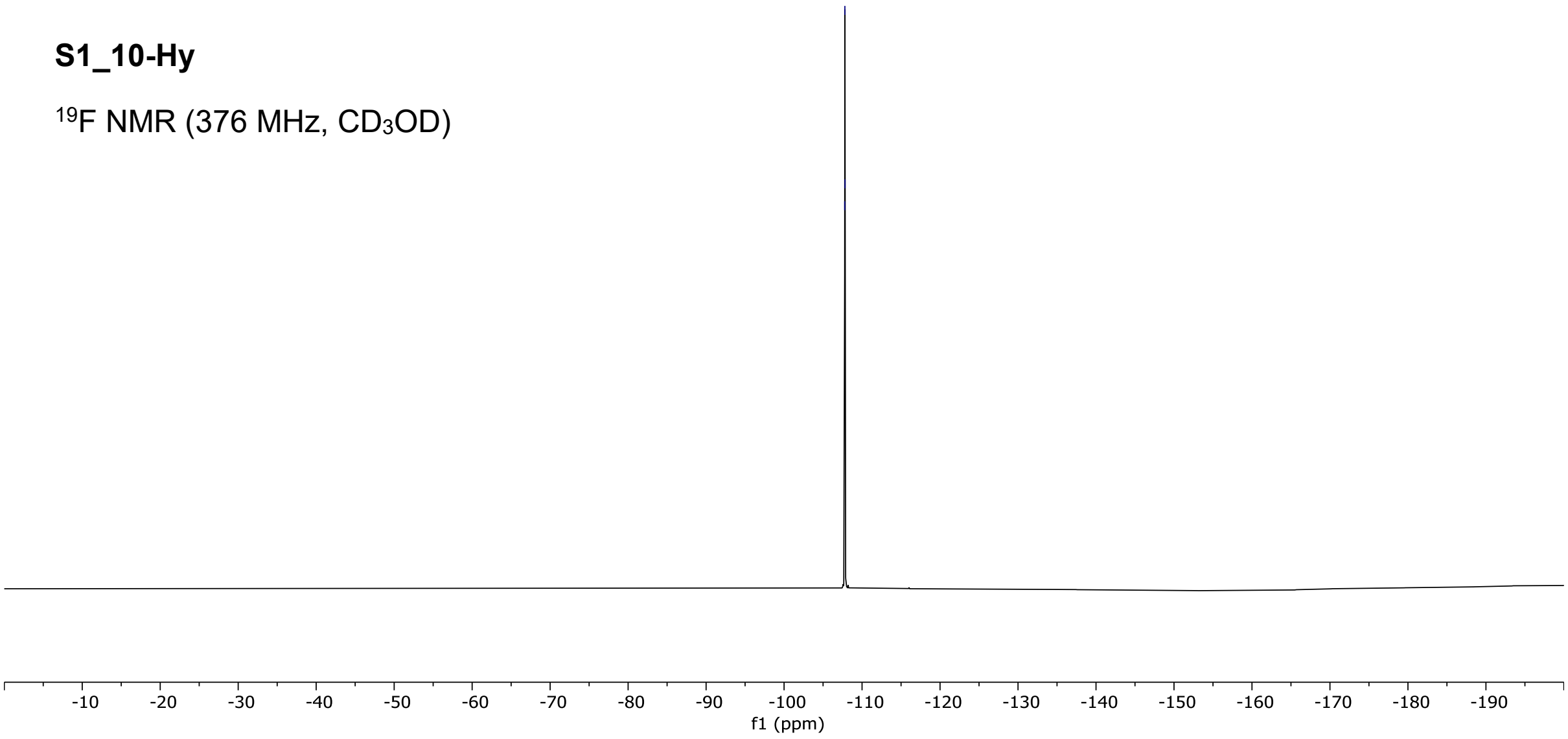

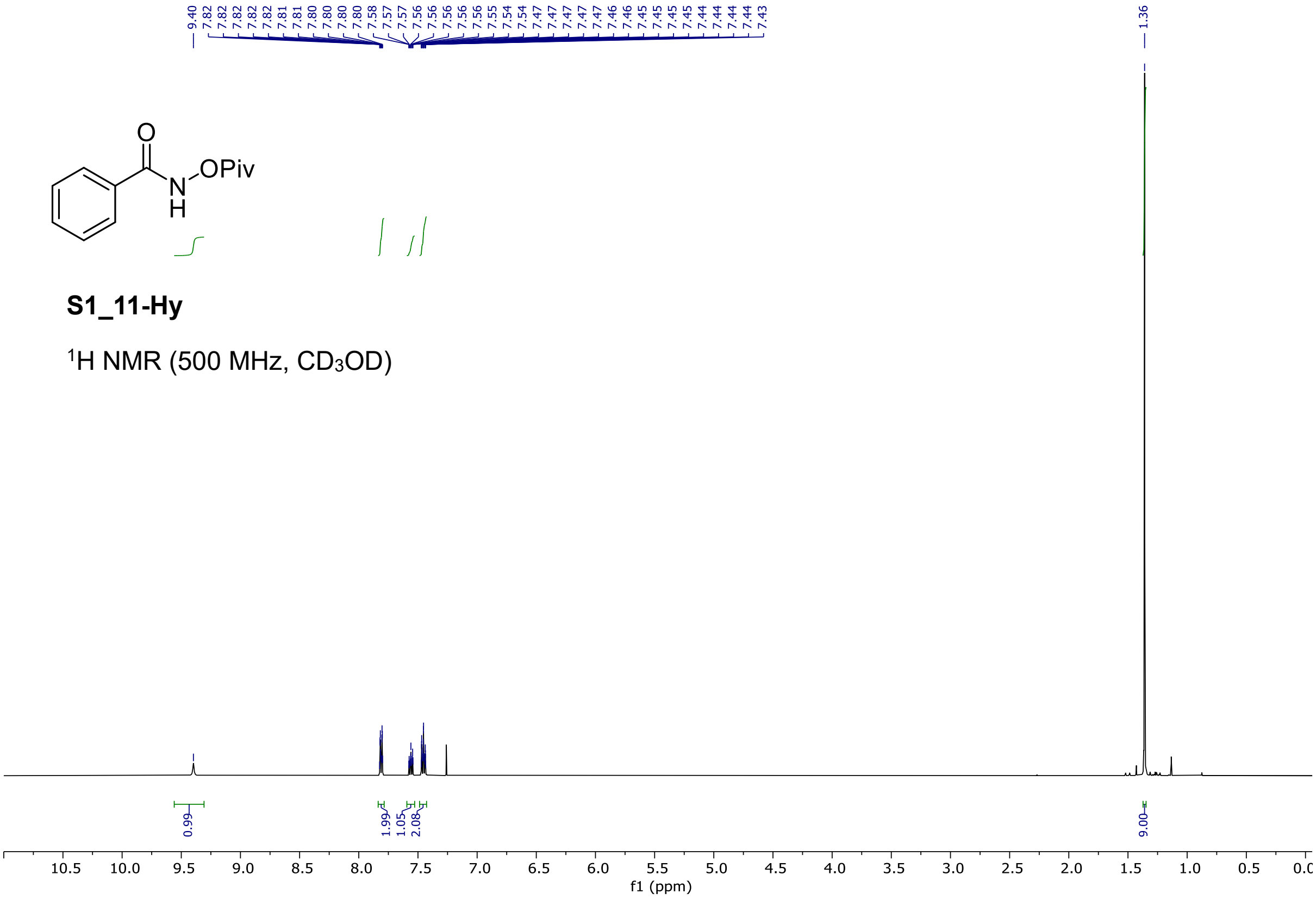

S1_11-Hy

^{1}H NMR (500 MHz, CD_3OD)

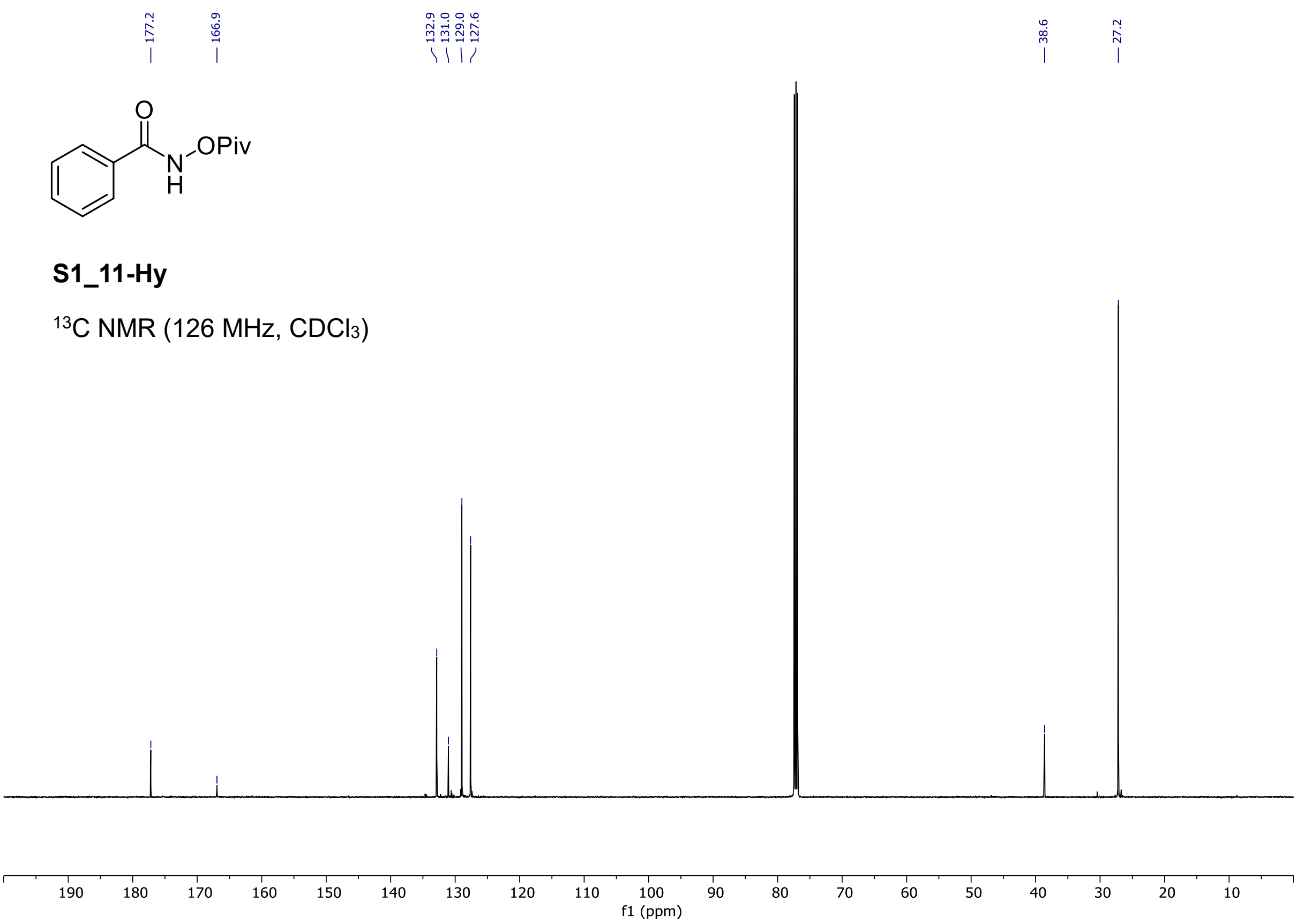

S1_11-Hy

^{13}C NMR (126 MHz, $CDCl_3$)

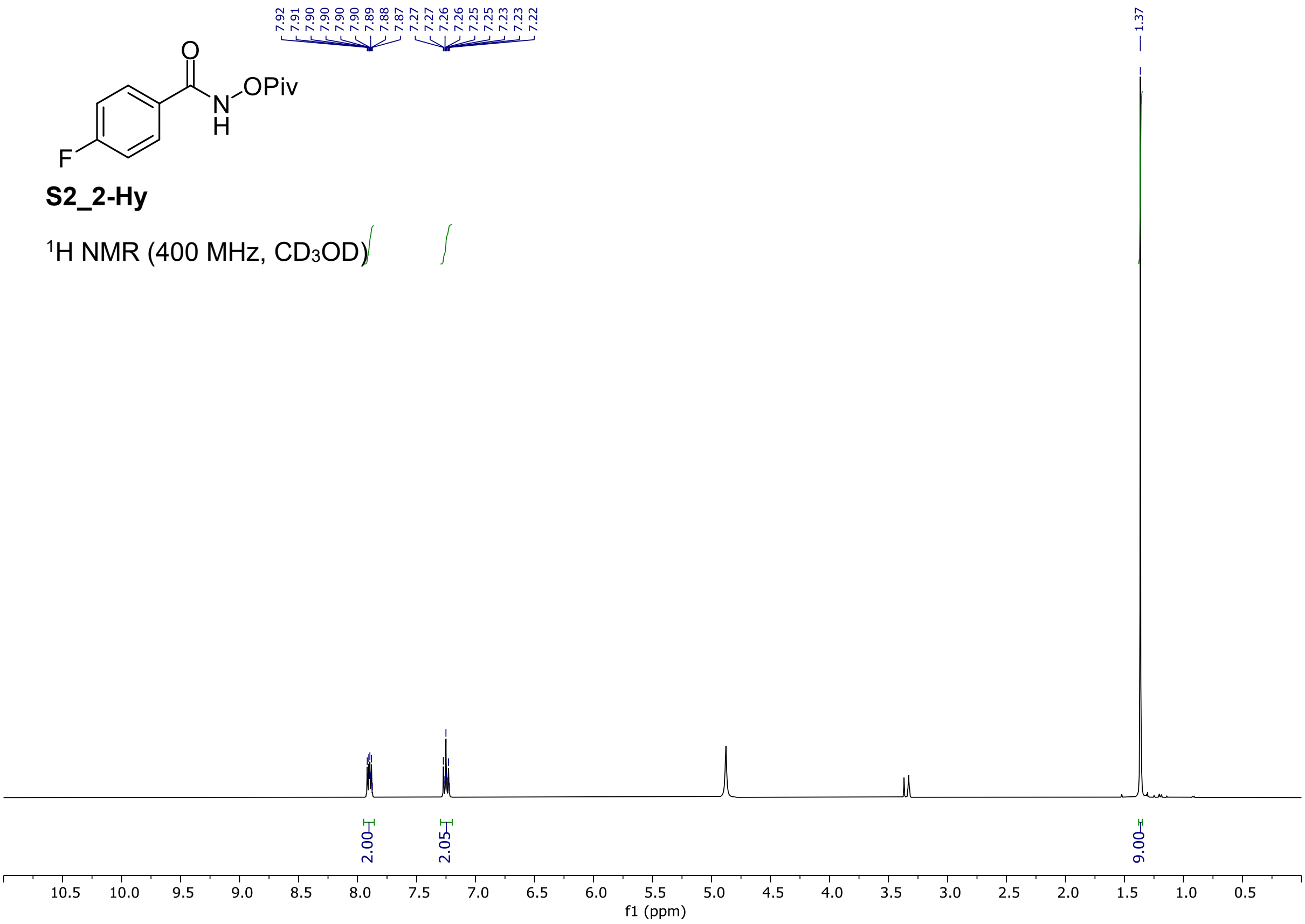
S2_2-Hy
1H NMR (400 MHz, CD3OD)
7.92 7.91 7.90 7.90 7.90 7.90 7.89 7.88 7.87 7.27 7.27 7.26 7.26 7.25 7.25 7.23 7.23 7.22
1.37
2.00
2.05
9.00
10.5 10.0 9.5 9.0 8.5 8.0 7.5 7.0 6.5 6.0 5.5 5.0 4.5 4.0 3.5 3.0 2.5 2.0 1.5 1.0 0.5
f1 (ppm)

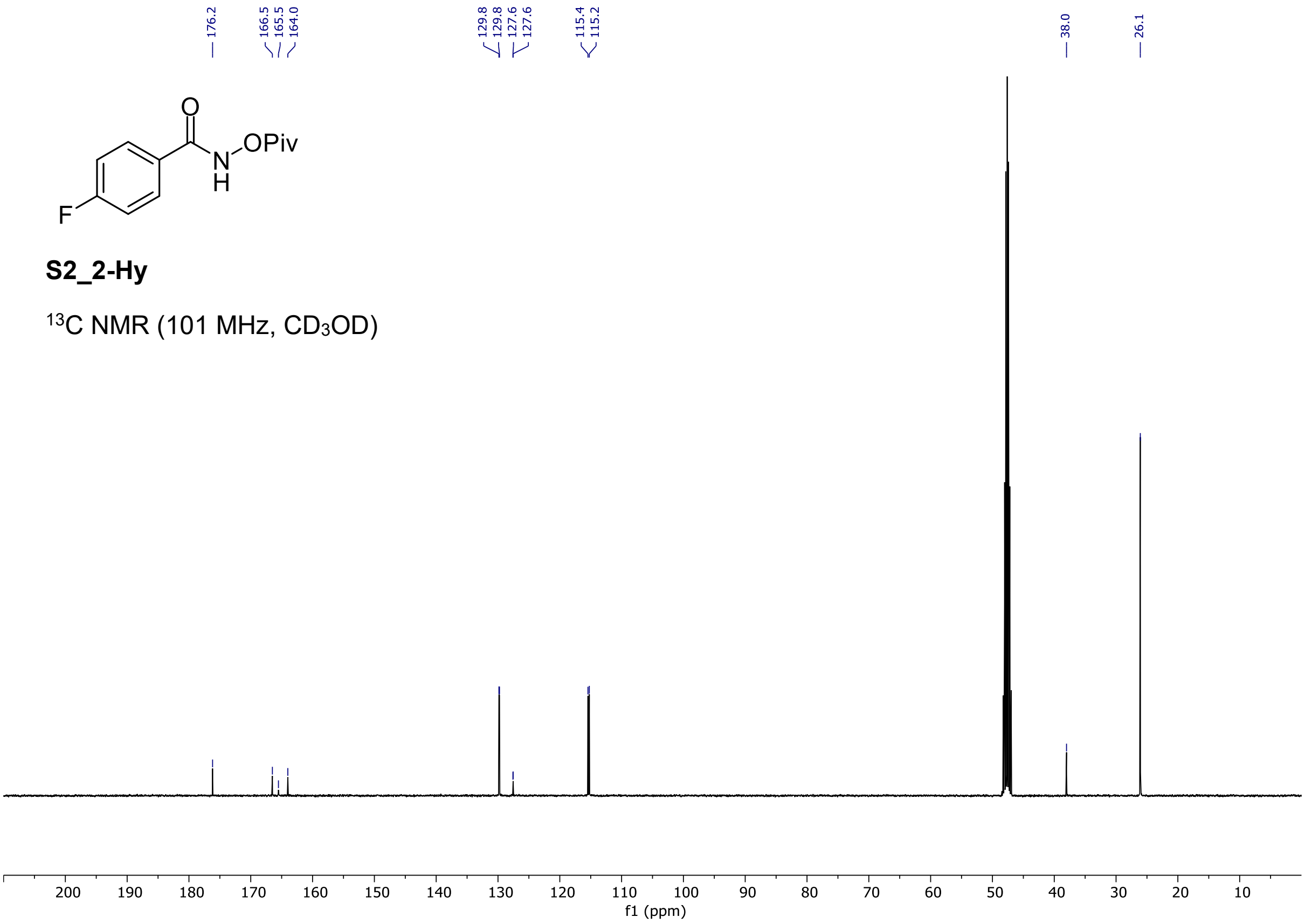
S2_2-Hy
13C NMR (101 MHz, CD3OD)
176.2
166.5 165.5 164.0
129.8 129.8 127.6 127.6
115.4 115.2
38.0
26.1
200 190 180 170 160 150 140 130 120 110 100 90 80 70 60 50 40 30 20 10
f1 (ppm)

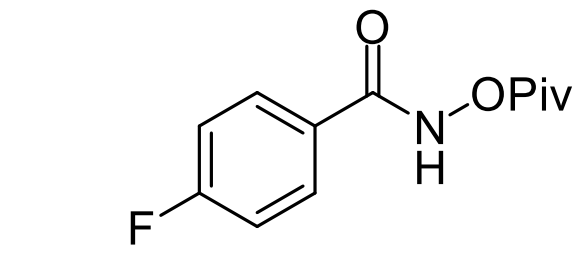

S2_2-Hy

^{19}F NMR (376 MHz, CD_3OD)

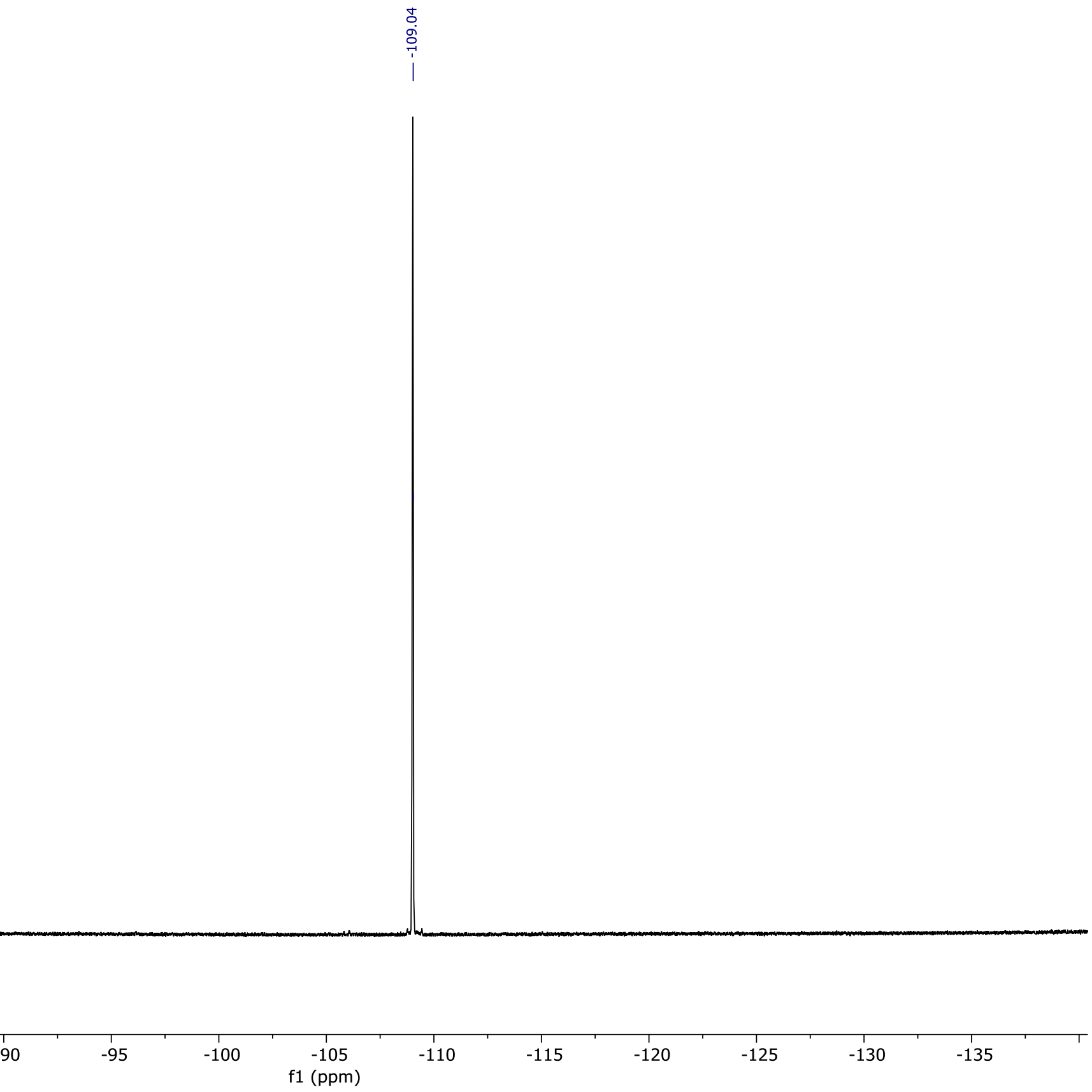

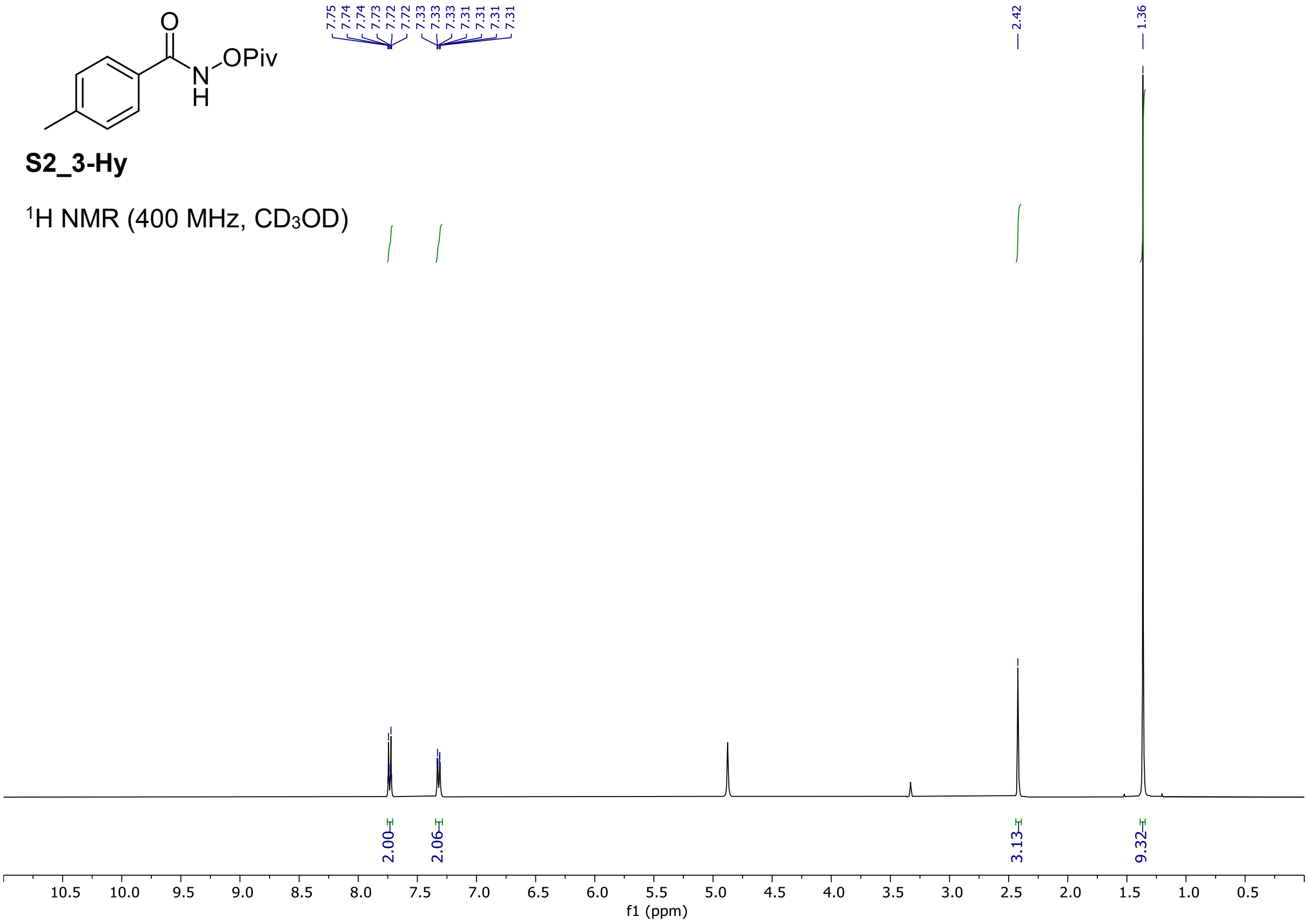
OPiv
S2_3-Hy
1H NMR (400 MHz, CD3OD)
7.75 7.74 7.74 7.73 7.72 7.72 7.33 7.33 7.33 7.31 7.31 7.31 7.31
2.42
1.36
2.00
2.06
3.13
9.32
f1 (ppm)

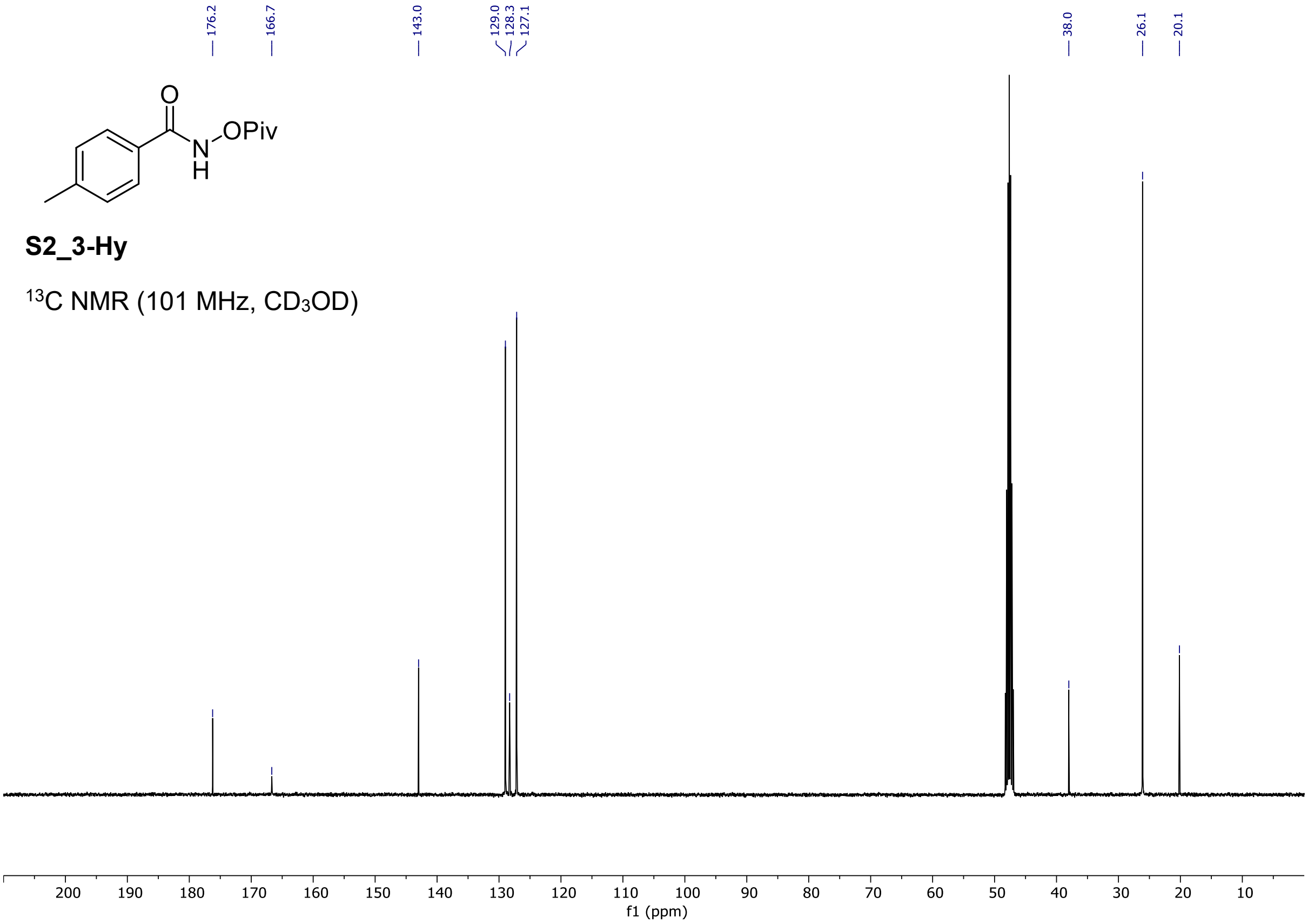
OPiv
S2_3-Hy
13C NMR (101 MHz, CD3OD)
176.2
166.7
143.0
129.0 128.3 127.1
38.0
26.1
20.1
f1 (ppm)

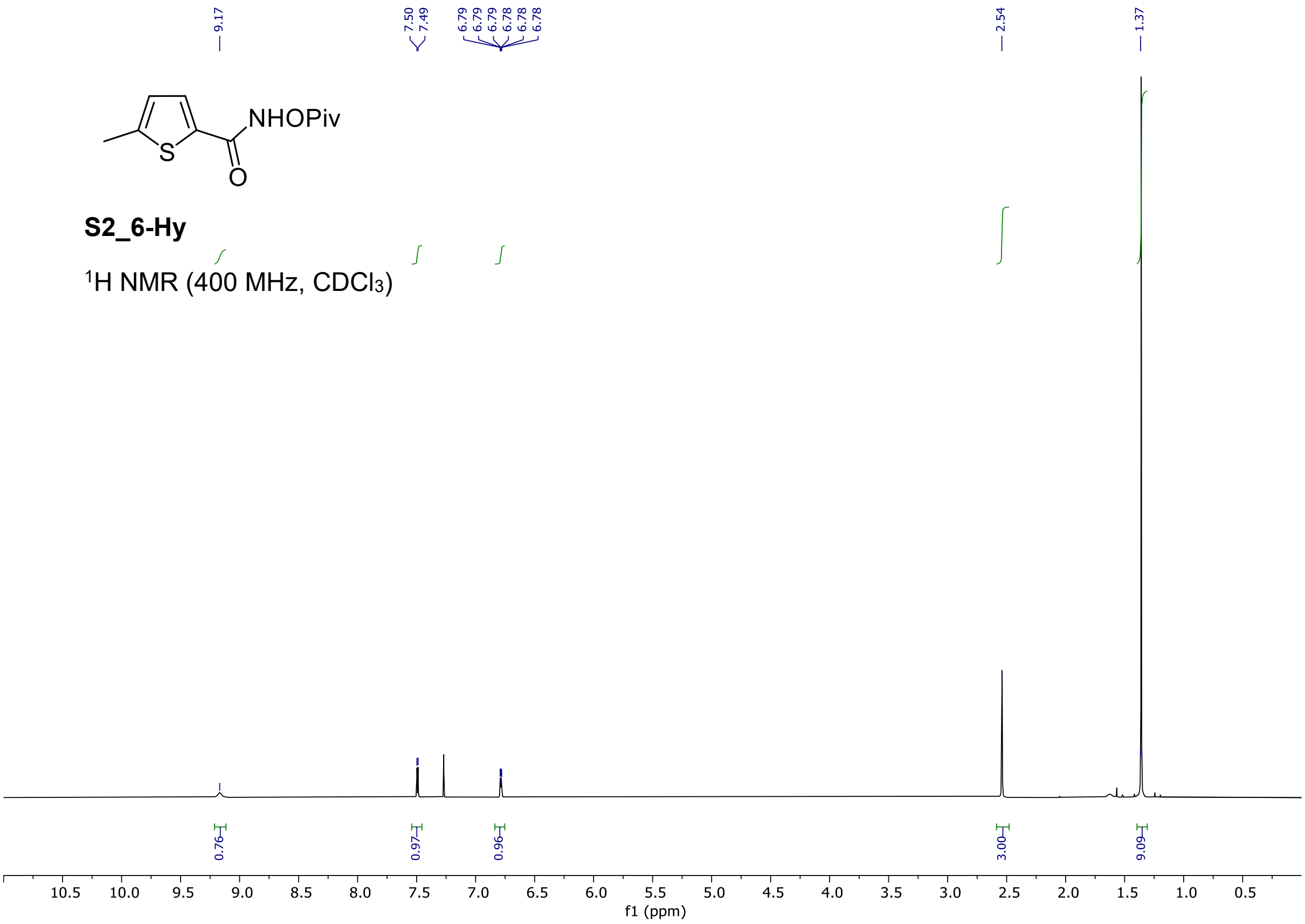
NHOPiv
S
O
S2_6-Hy
1H NMR (400 MHz, CDCl3)
9.17
7.50
7.49
6.79
6.79
6.79
6.78
6.78
6.78
2.54
1.37
0.76
0.97
0.96
3.00
9.09
f1 (ppm)

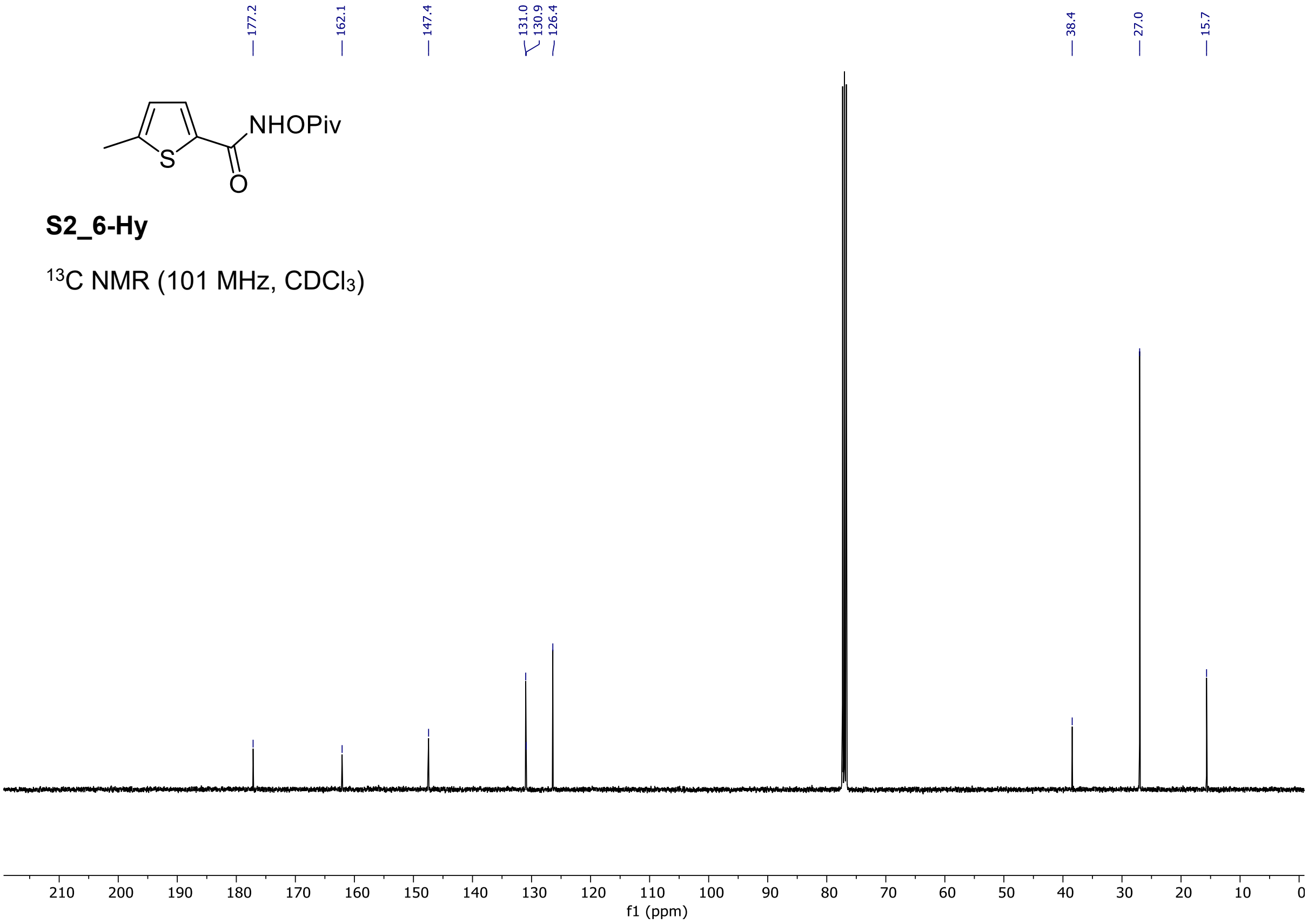
NHOPiv
S
O
S2_6-Hy
13C NMR (101 MHz, CDCl3)
177.2
162.1
147.4
131.0
130.9
126.4
38.4
27.0
15.7
f1 (ppm)

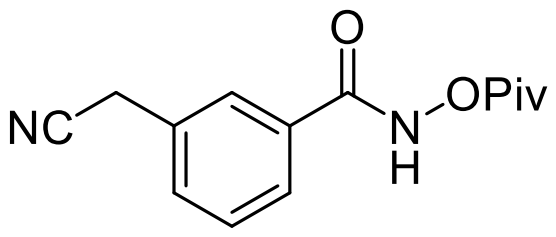

S2_7-Hy

^{1}H NMR (400 MHz, CD_3OD)

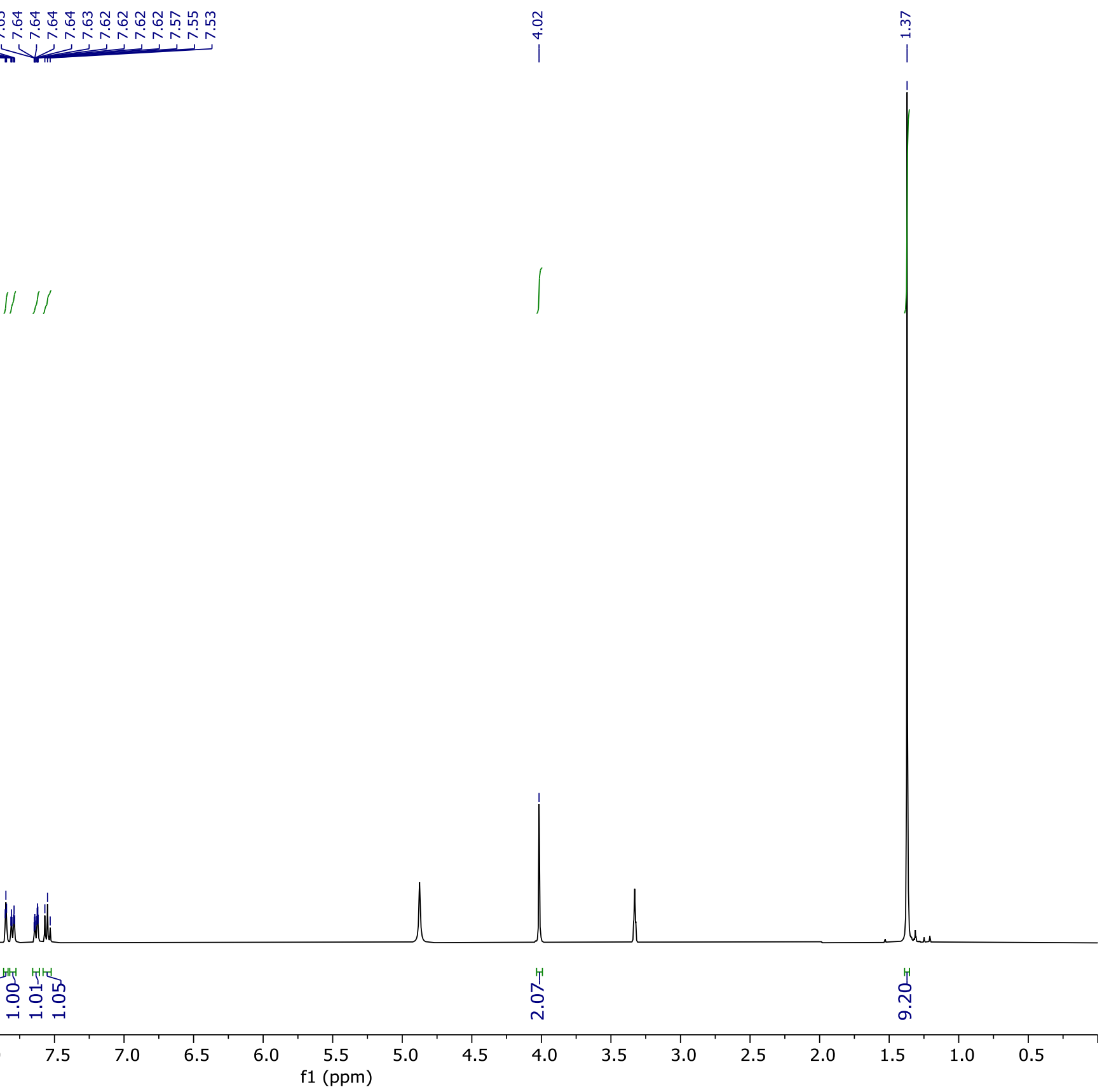

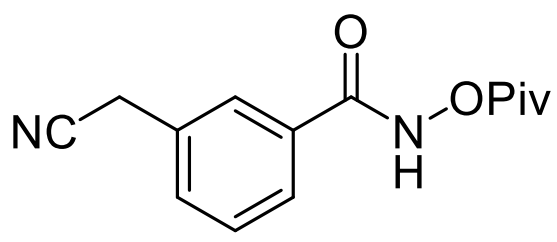

S2_7-Hy

^{13}C NMR (101 MHz, CD_3OD)

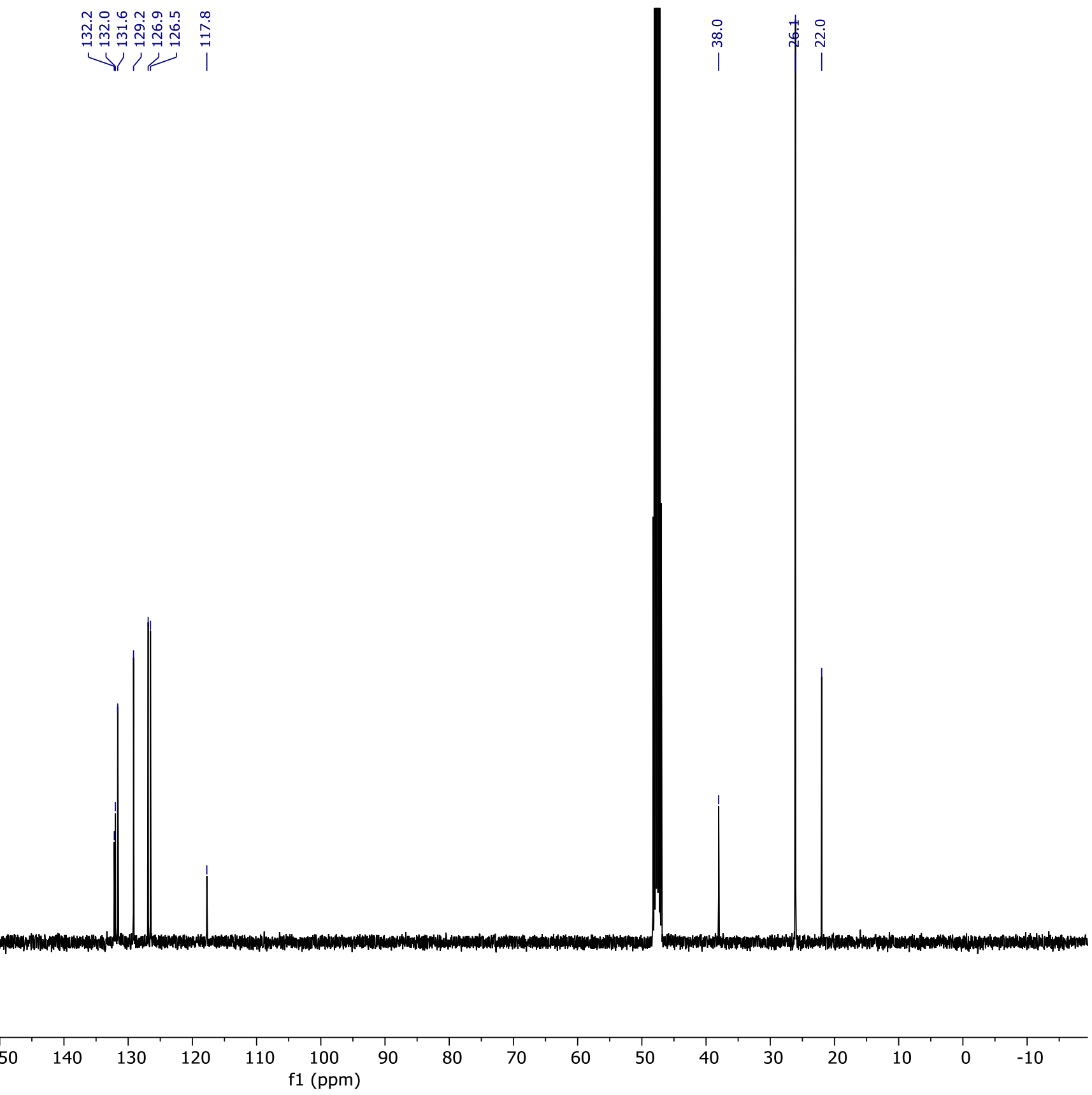

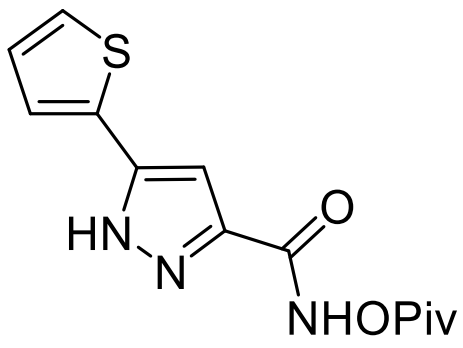

S2_9-Hy

^{1}H NMR (400 MHz, $CDCl_3$)

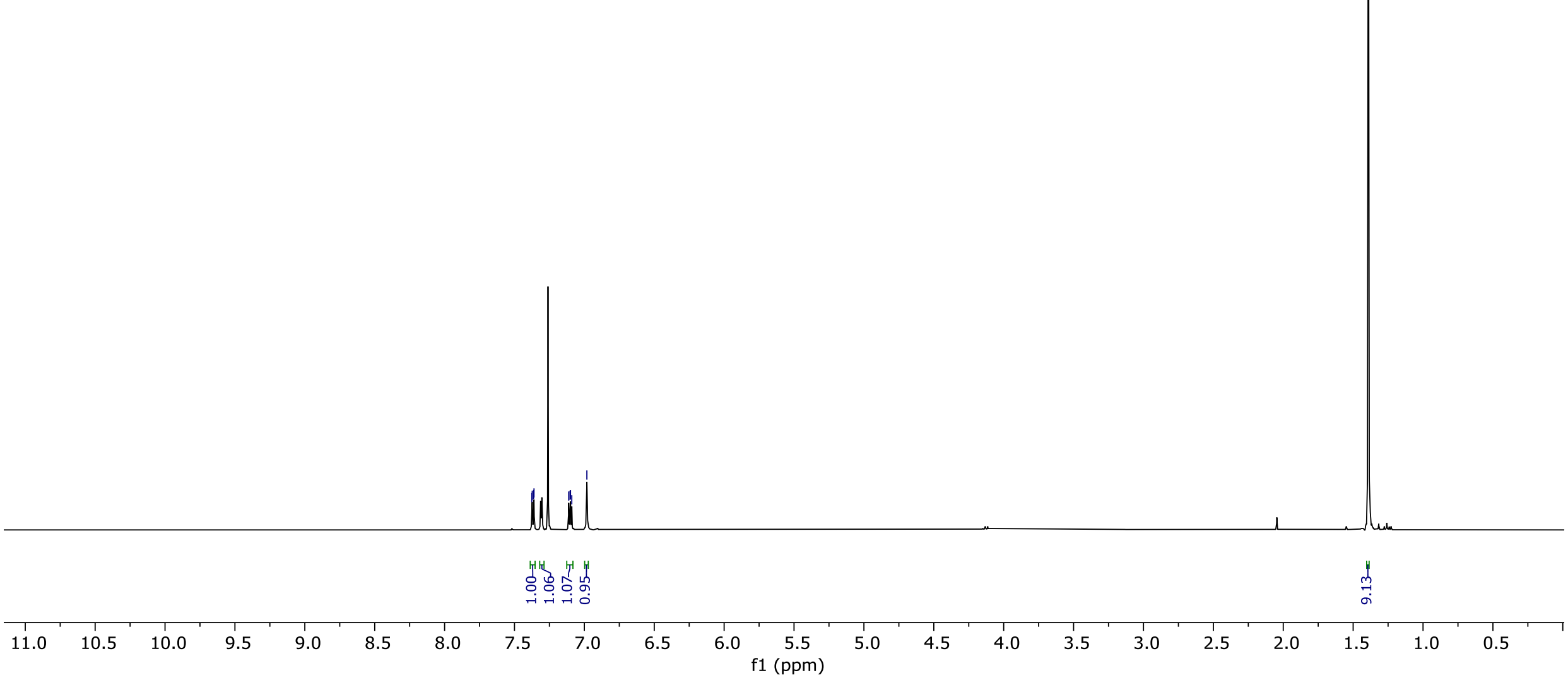

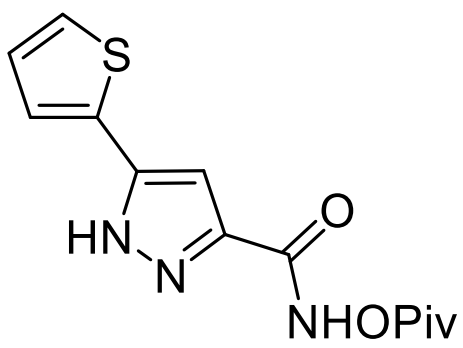

S2_9-Hy

^{13}C NMR (101 MHz, CD_3OD)

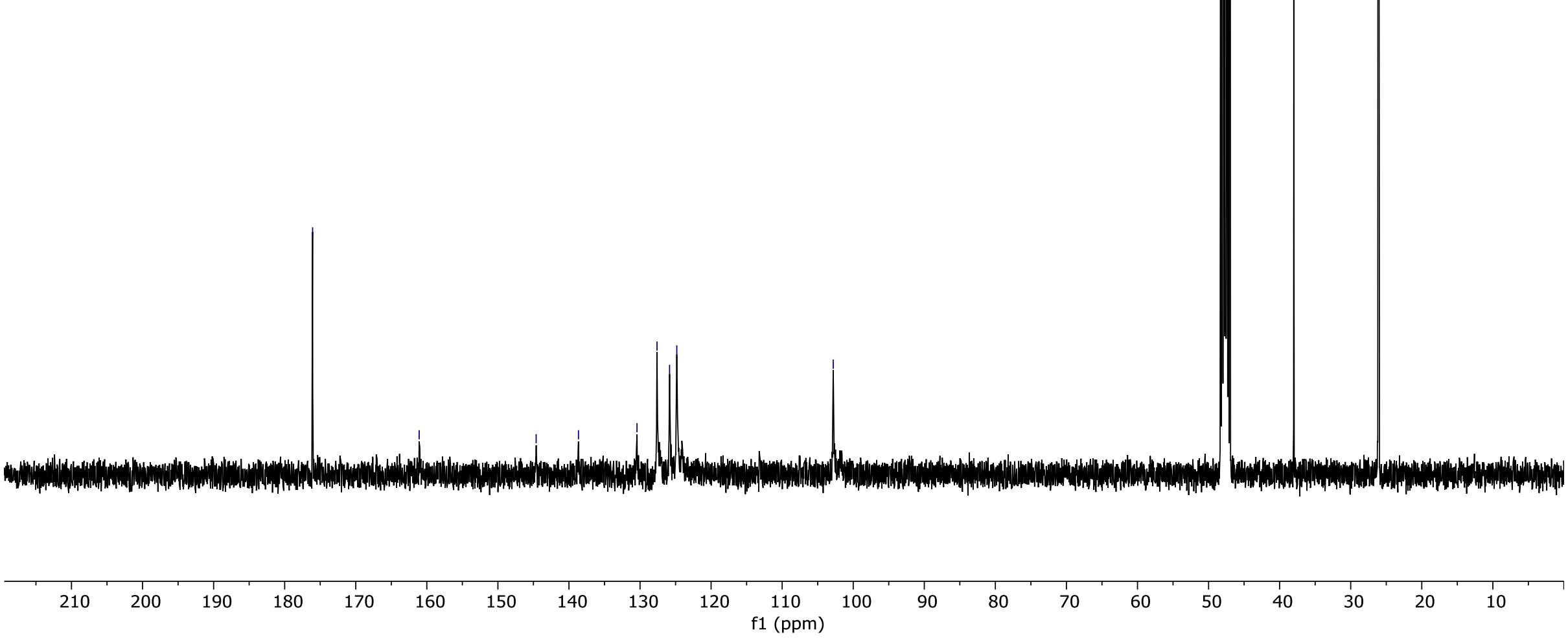

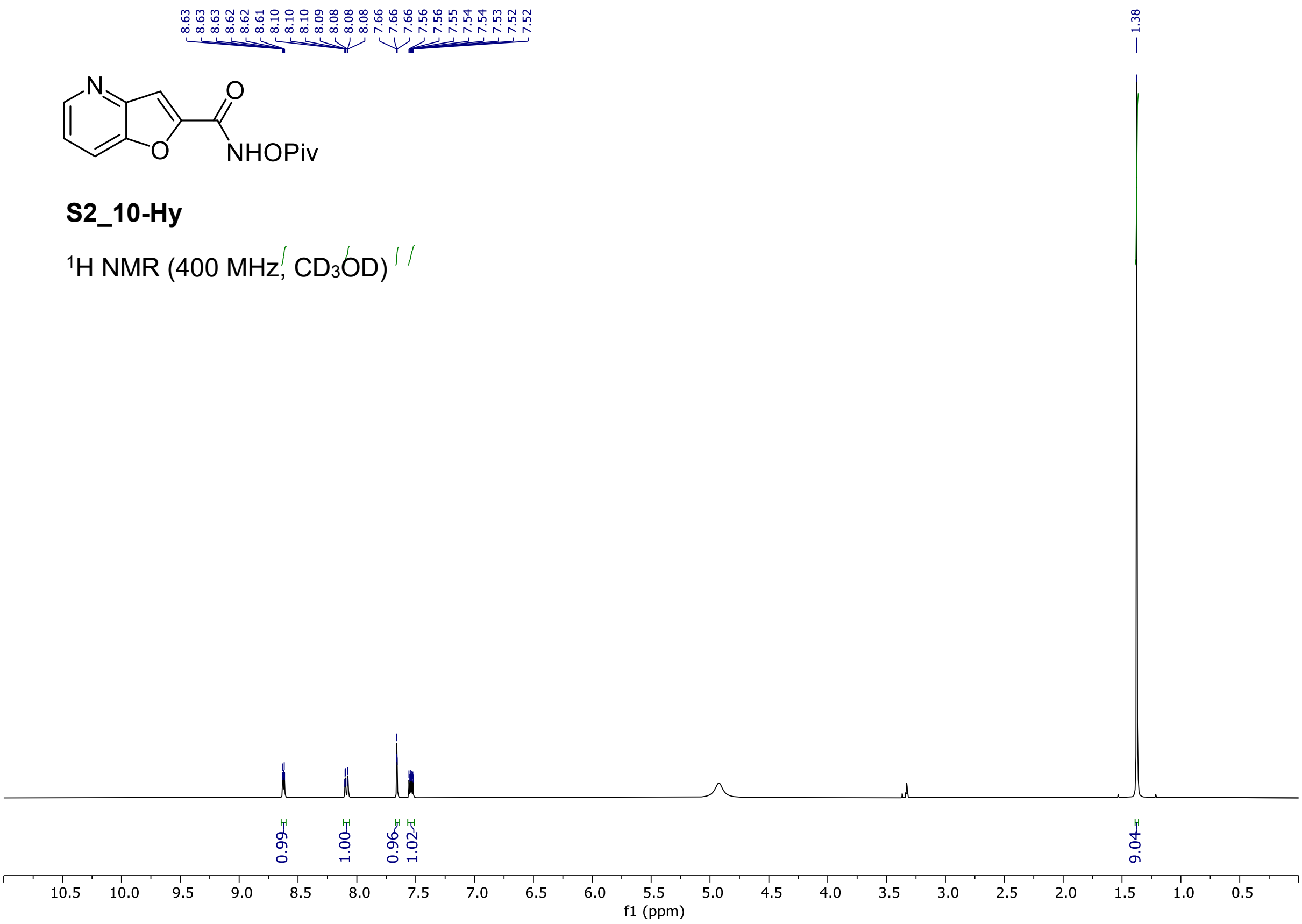

S2_10-Hy

^{1}H NMR (400 MHz, CD_3OD)

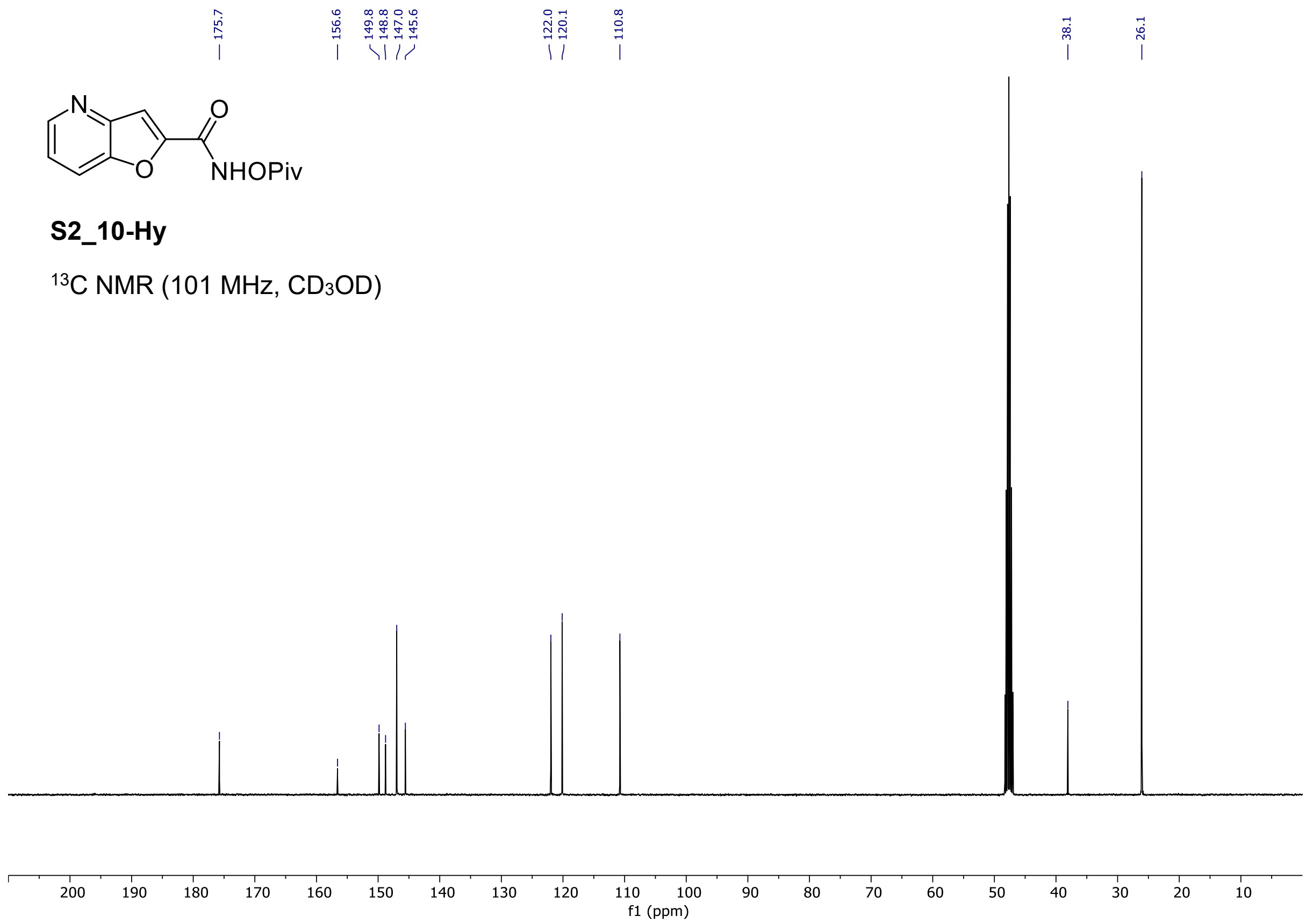

S2_10-Hy

^{13}C NMR (101 MHz, CD_3OD)

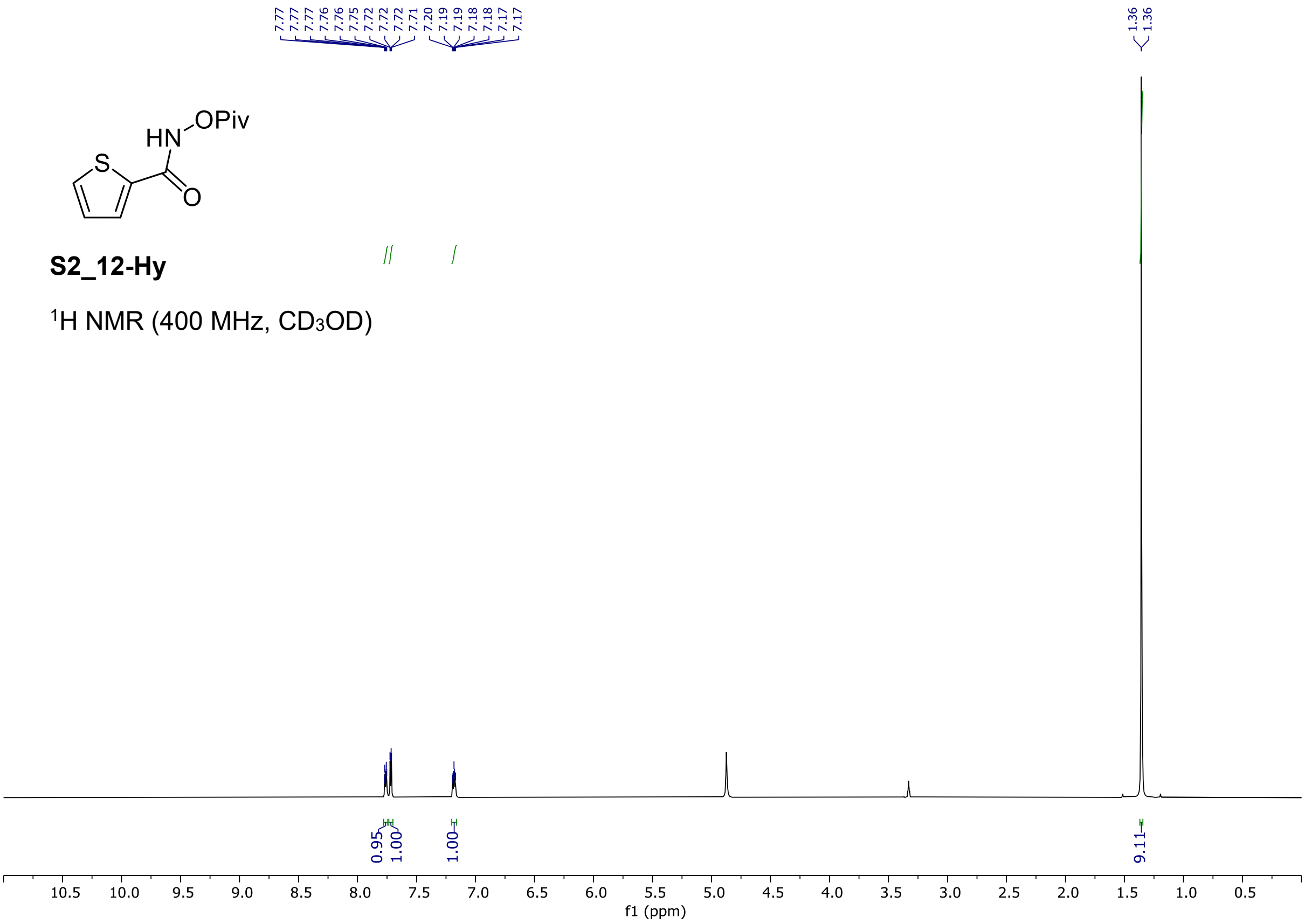

S2_12-Hy

^{1}H NMR (400 MHz, CD_3OD)

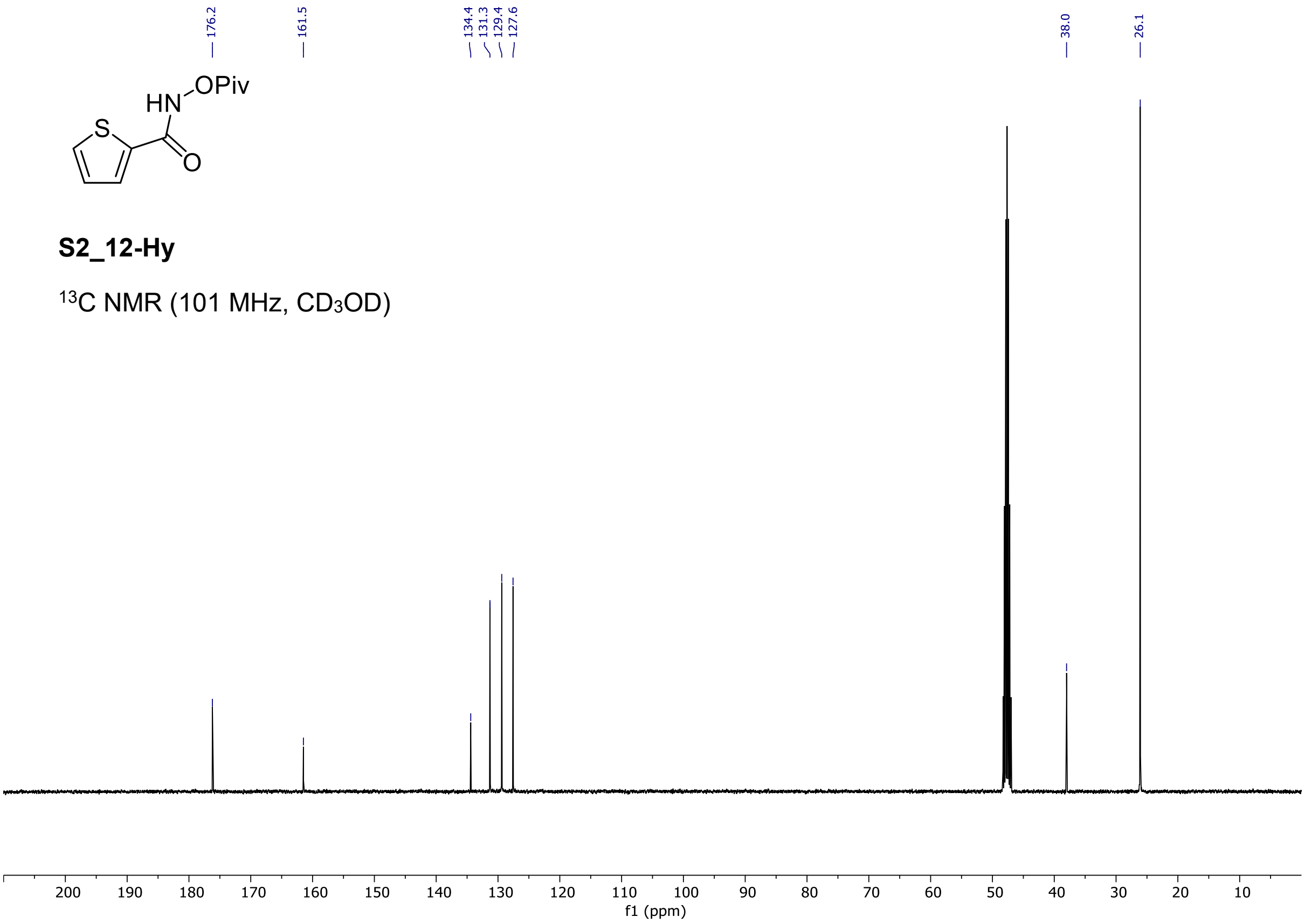

S2_12-Hy

^{13}C NMR (101 MHz, CD_3OD)

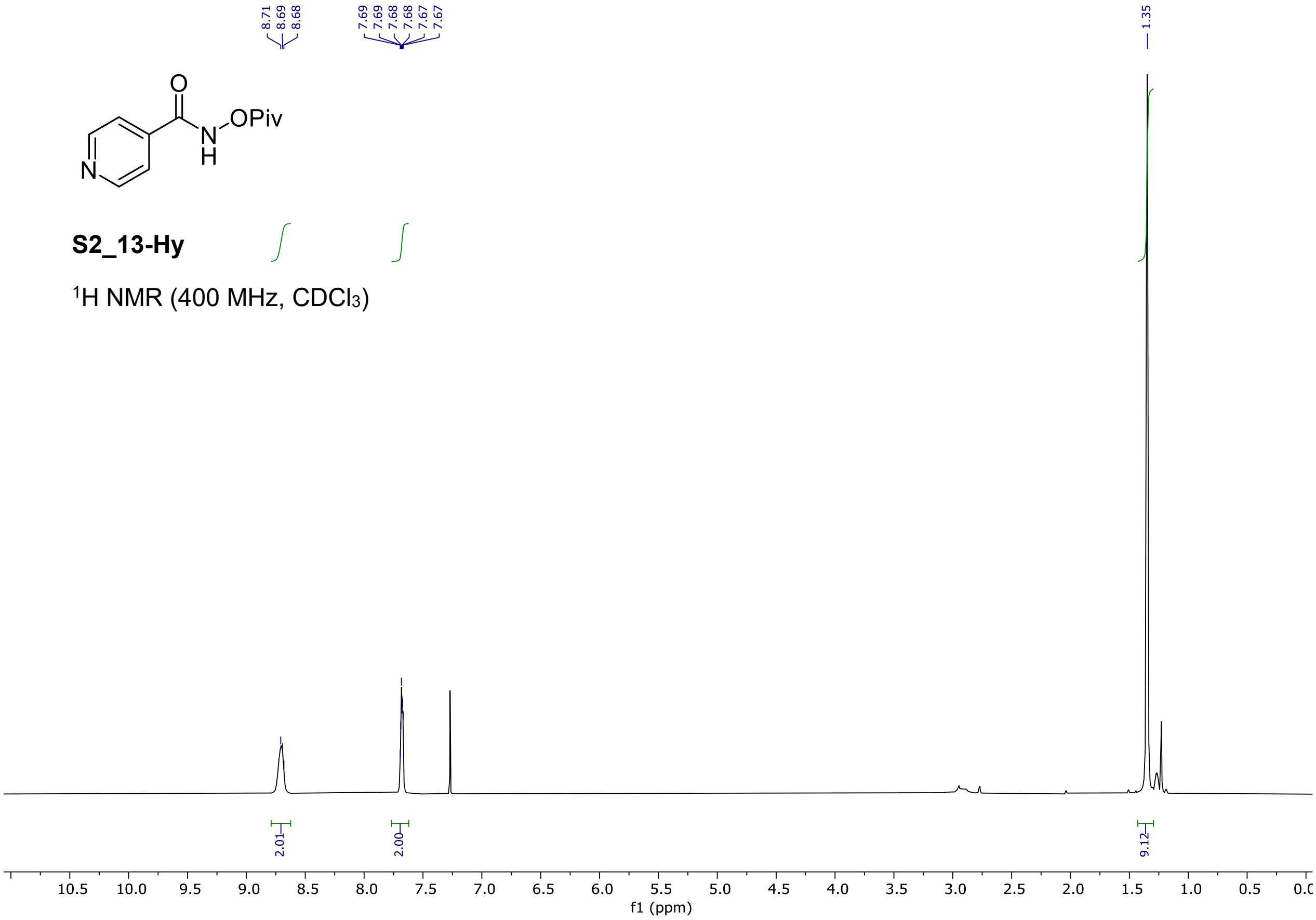

S2_13-Hy

^{1}H NMR (400 MHz, $CDCl_3$)

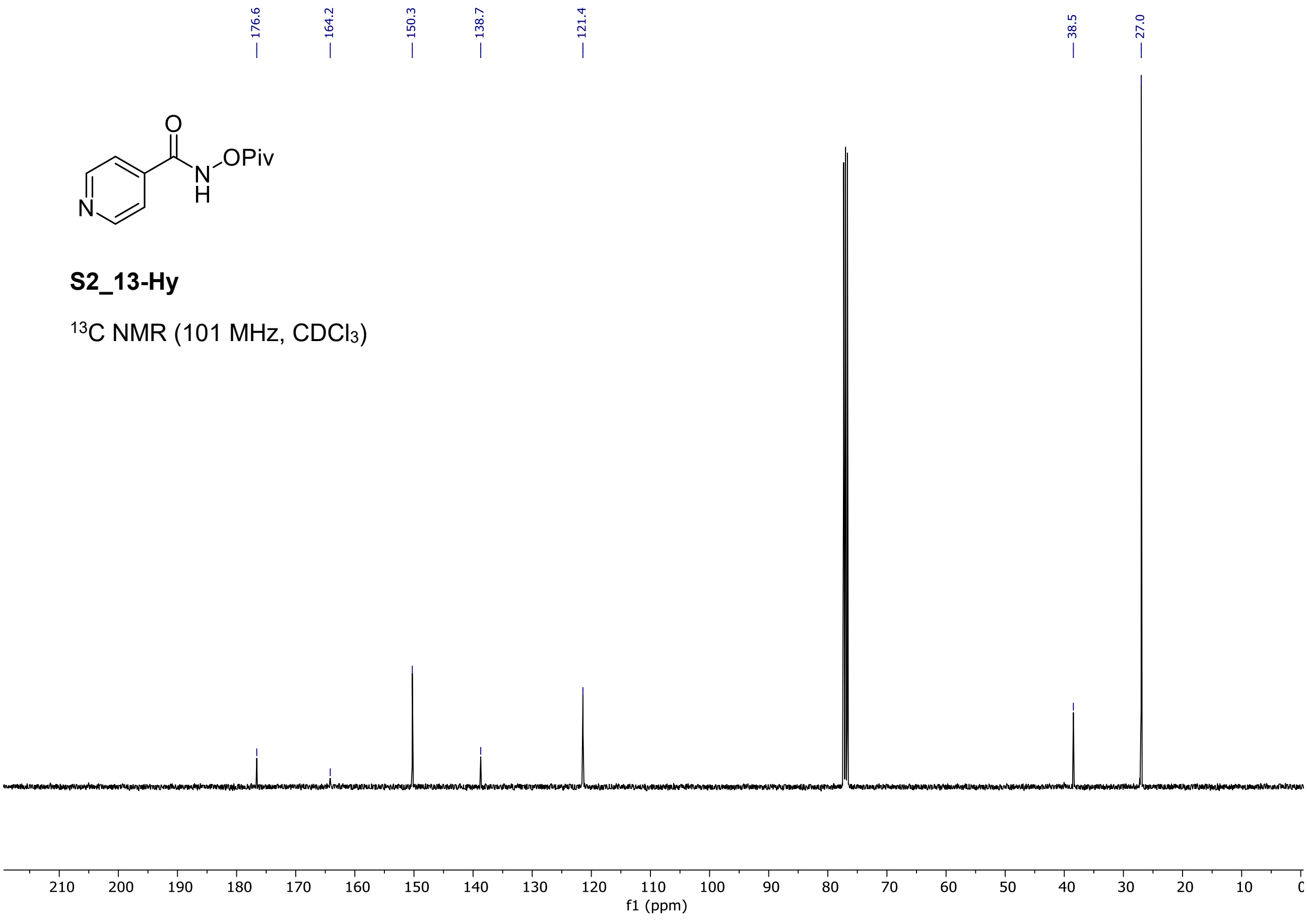

S2_13-Hy

^{13}C NMR (101 MHz, $CDCl_3$)

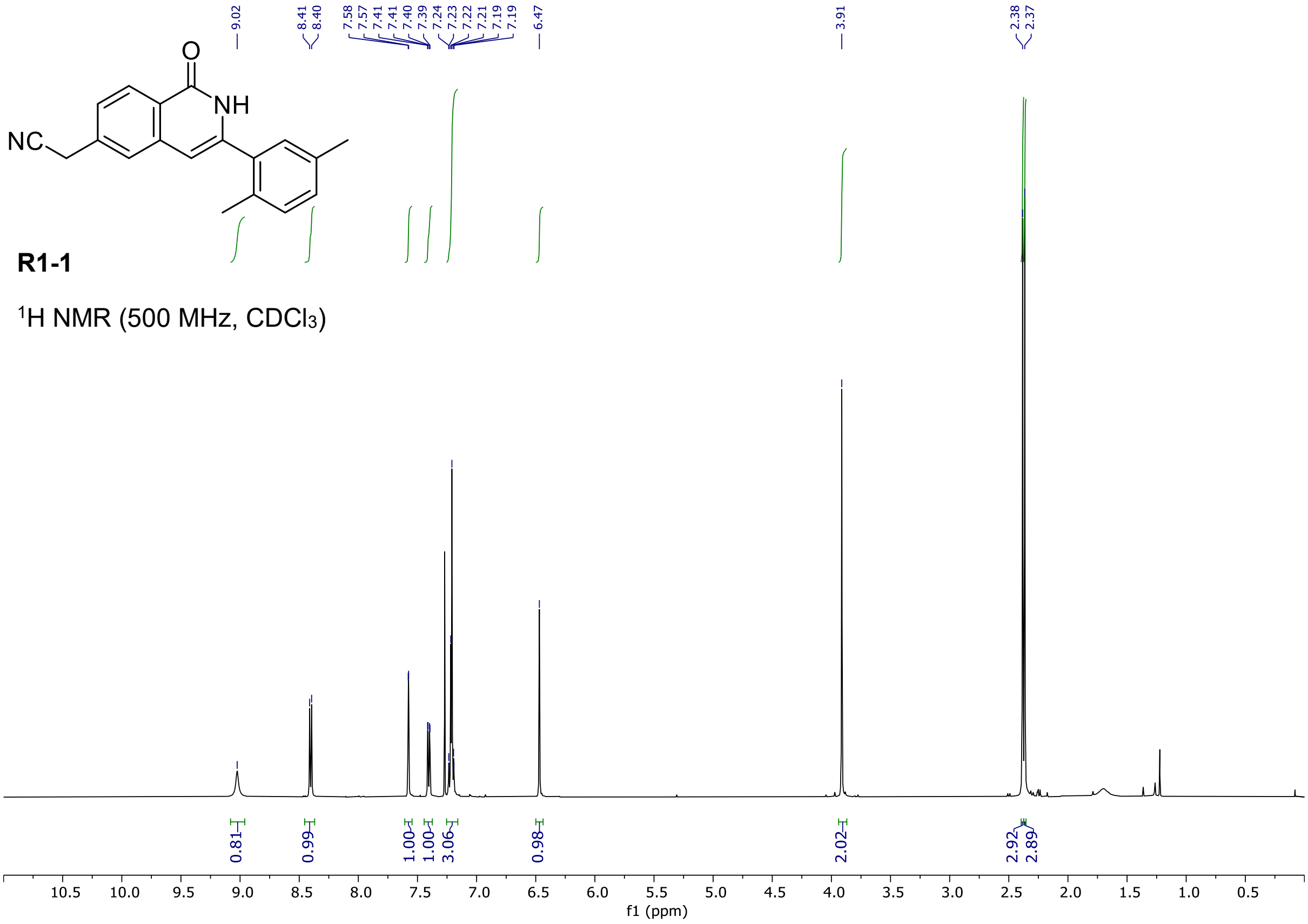
R1-1
^{1}H NMR (500 MHz, CDCl$_3$)
NC
O
NH
9.02
8.41
8.40
7.58
7.57
7.41
7.41
7.40
7.39
7.24
7.23
7.22
7.21
7.19
7.19
6.47
3.91
2.38
2.37
0.81
0.99
1.00
1.00
3.06
0.98
2.02
2.92
2.89
f1 (ppm)

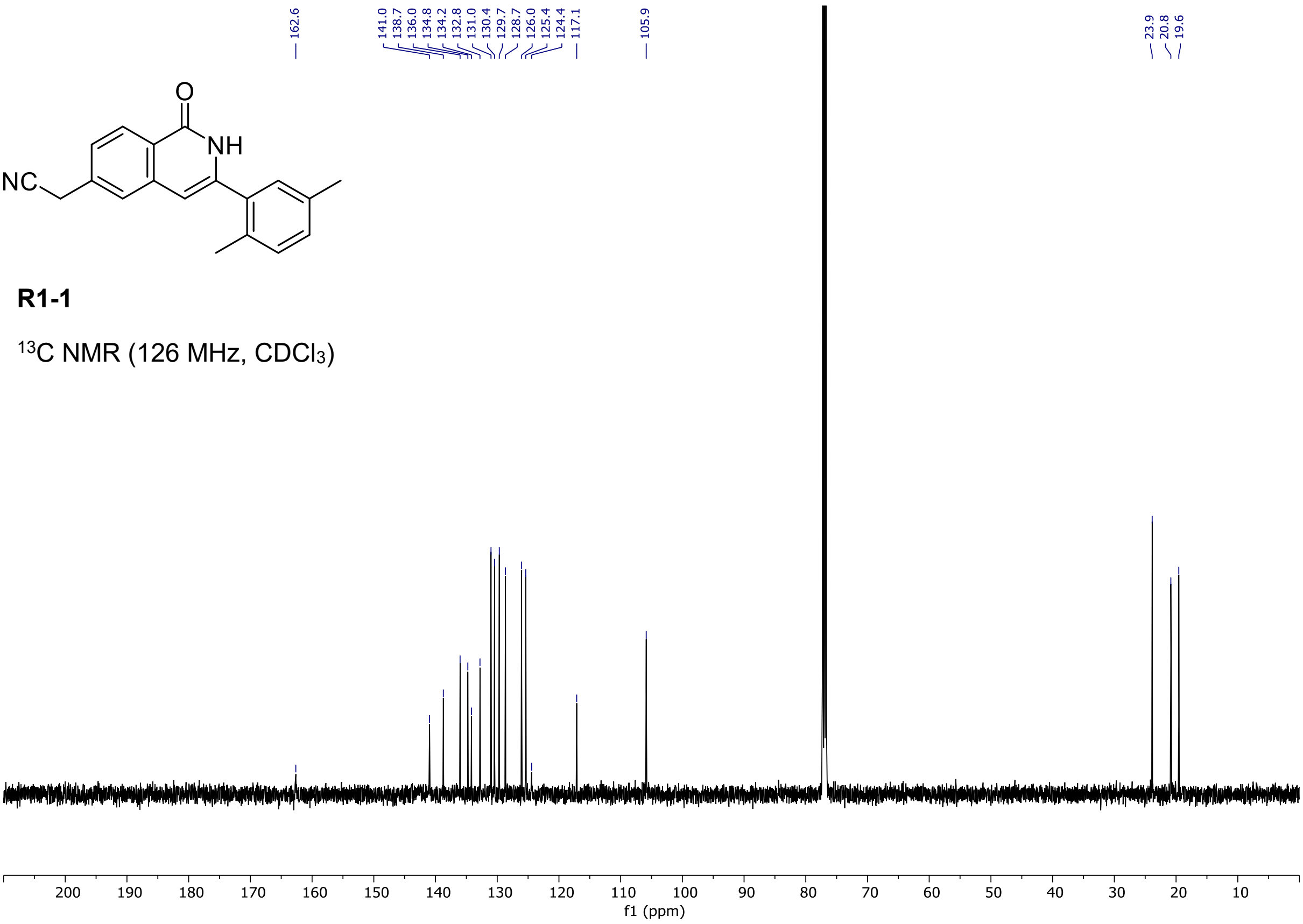
R1-1
^{13}C NMR (126 MHz, CDCl$_3$)
NC
O
NH
162.6
141.0
138.7
136.0
134.8
134.2
132.8
131.0
130.4
129.7
128.7
126.0
125.4
124.4
117.1
105.9
23.9
20.8
19.6
f1 (ppm)

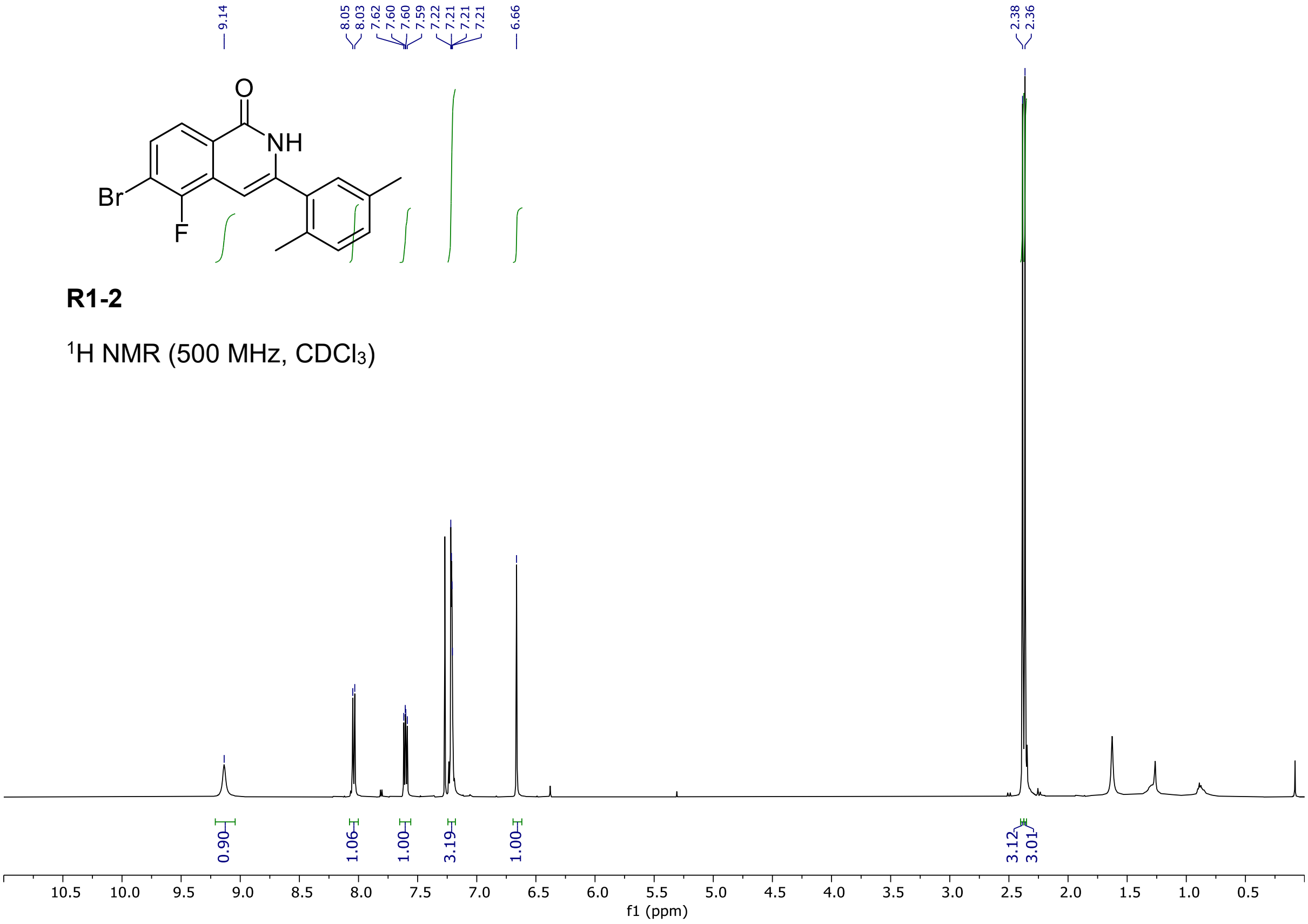
9.14
8.05
8.03
7.62
7.60
7.60
7.59
7.22
7.21
7.21
7.21
6.66
2.38
2.36
Br
F
O
NH
R1-2
1H NMR (500 MHz, CDCl3)
0.90
1.06
1.00
3.19
1.00
3.12
3.01
f1 (ppm)

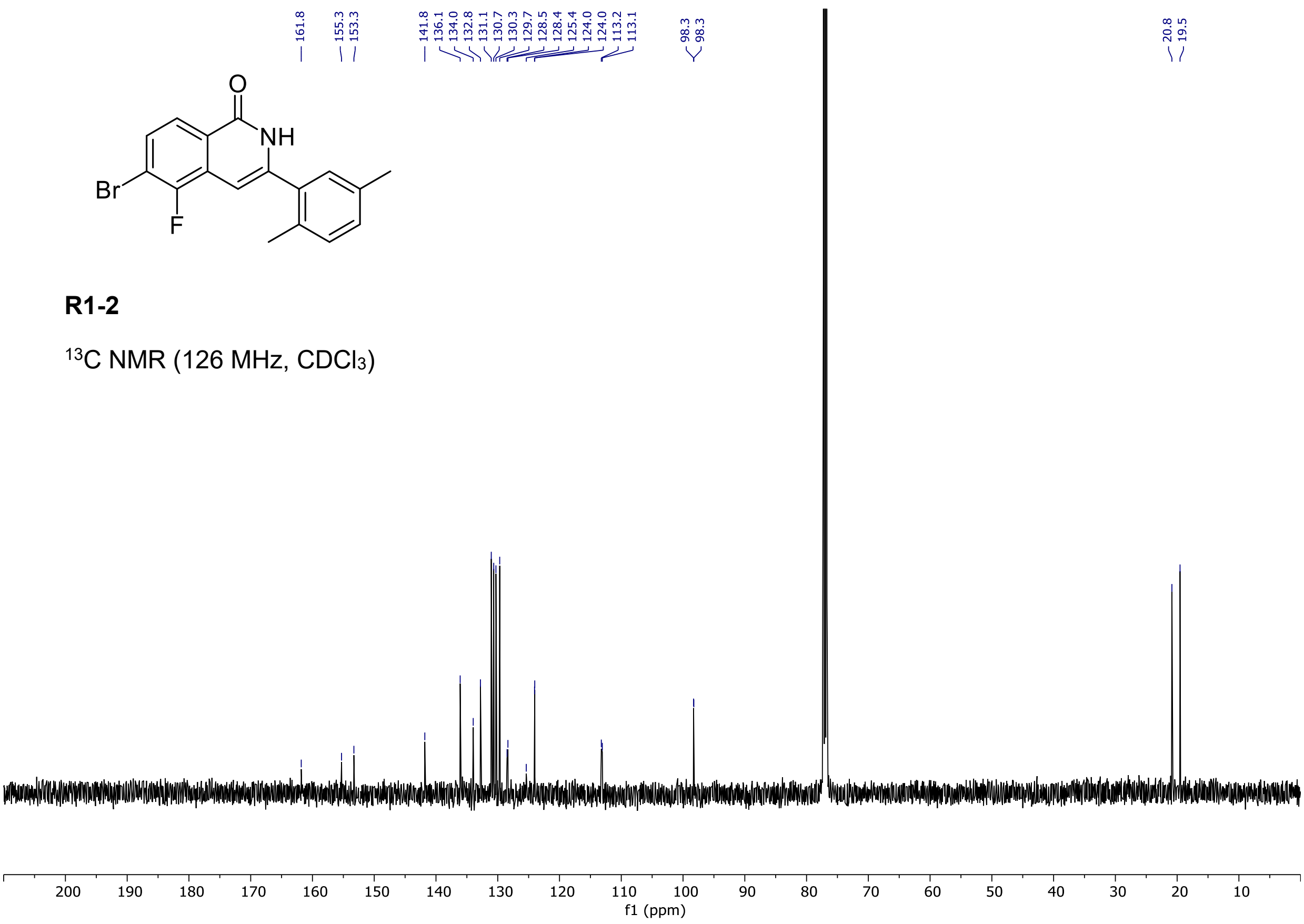
161.8
155.3
153.3
141.8
136.1
134.0
132.8
131.1
130.7
130.3
129.7
128.5
128.4
125.4
124.0
124.0
113.2
113.1
98.3
98.3
20.8
19.5
Br
F
O
NH
R1-2
13C NMR (126 MHz, CDCl3)
f1 (ppm)

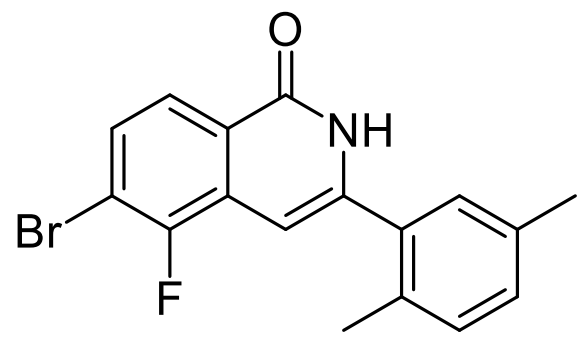

R1-2

^{19}F NMR (471 MHz, $CDCl_3$)

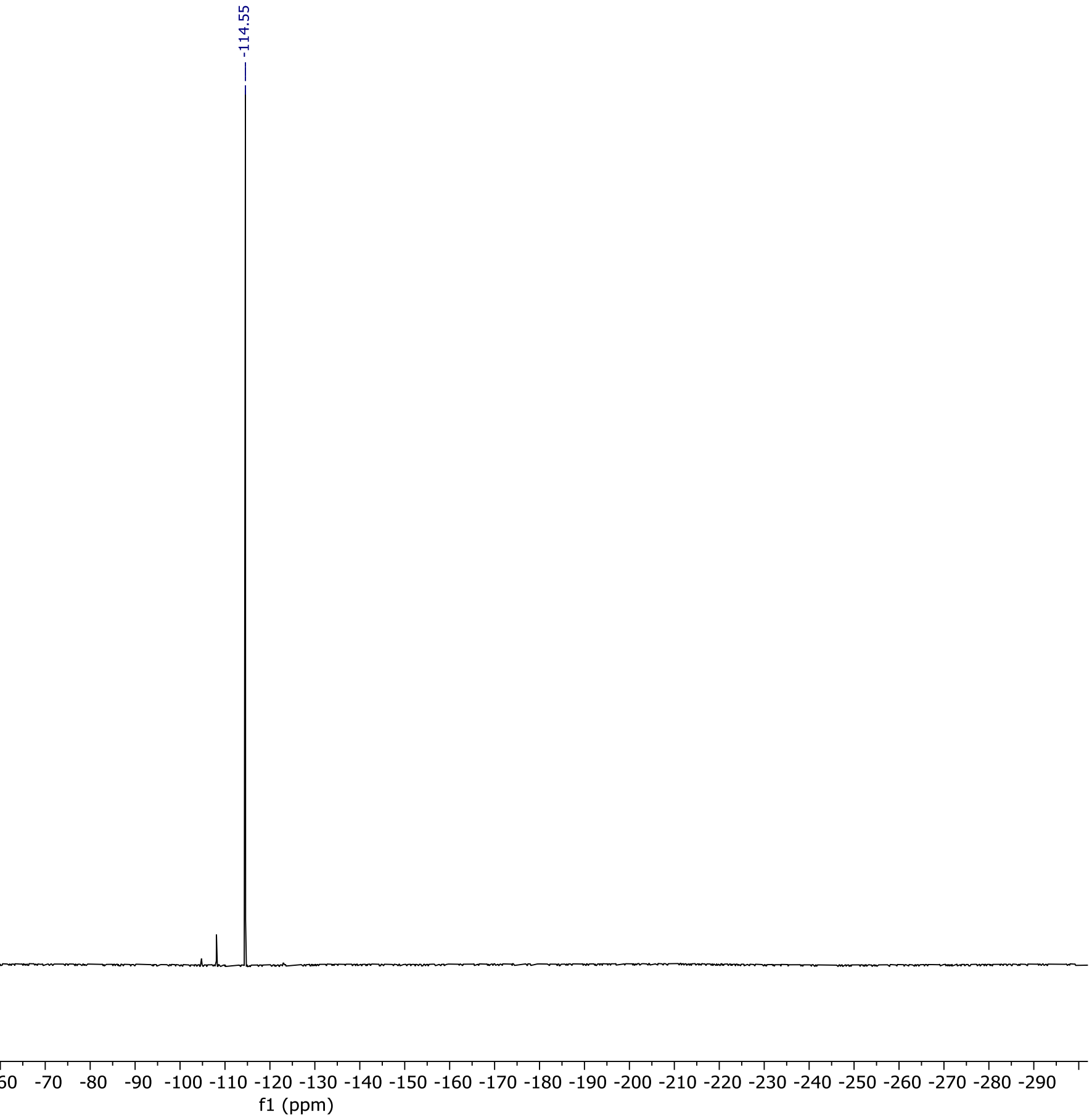

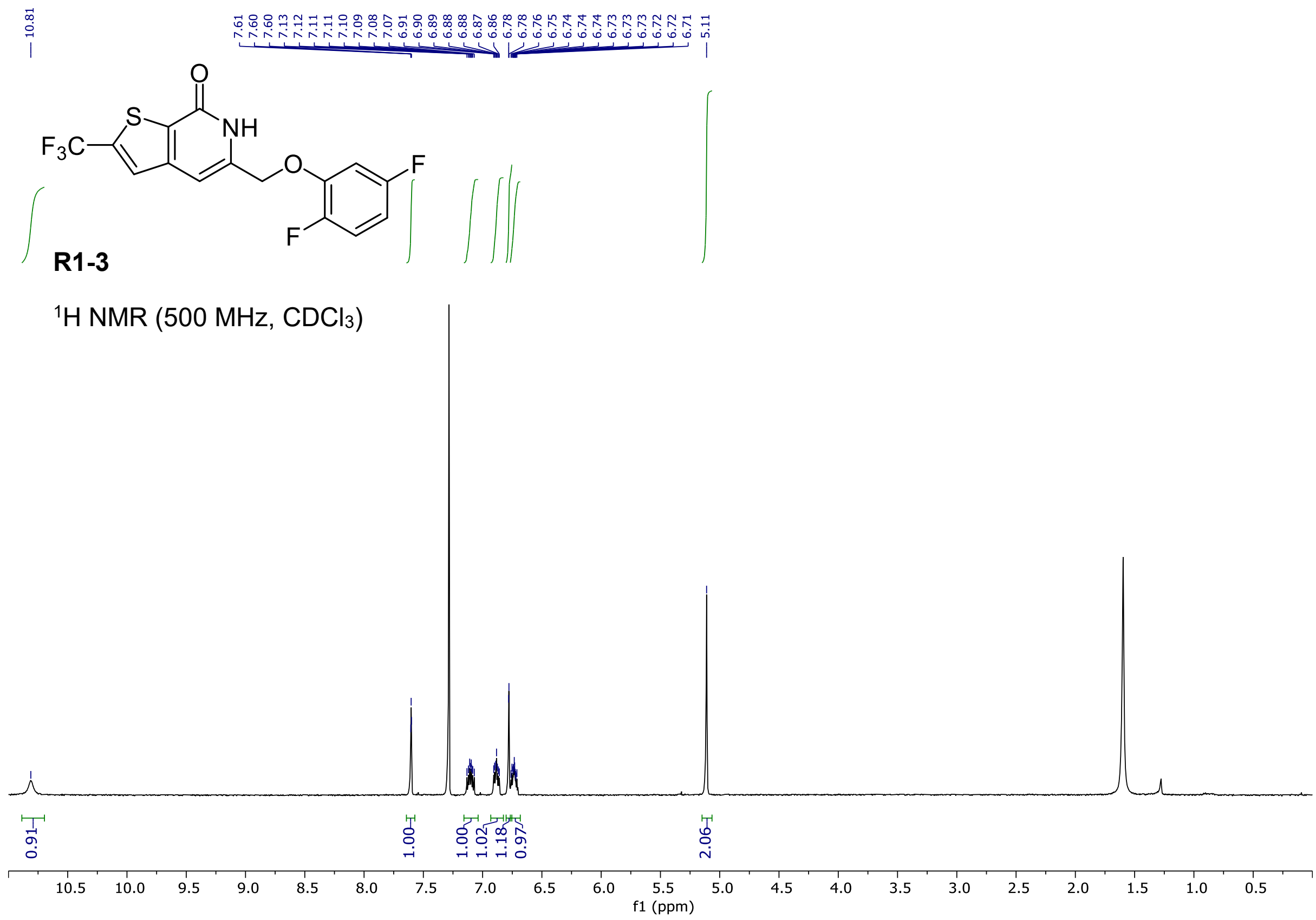
R1-3
¹H NMR (500 MHz, CDCl3)
f1 (ppm)

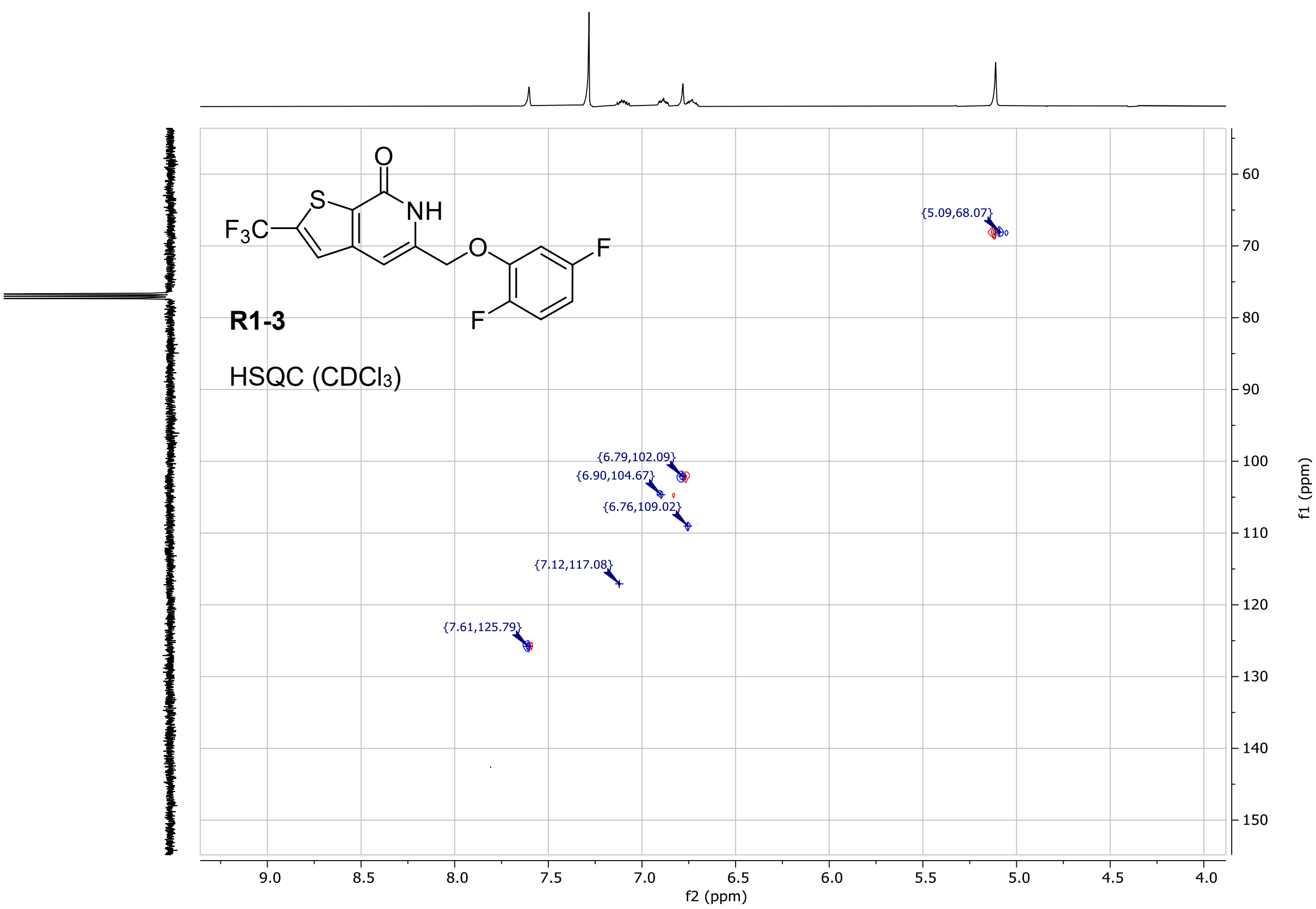
R1-3
HSQC (CDCl3)
f2 (ppm)
f1 (ppm)

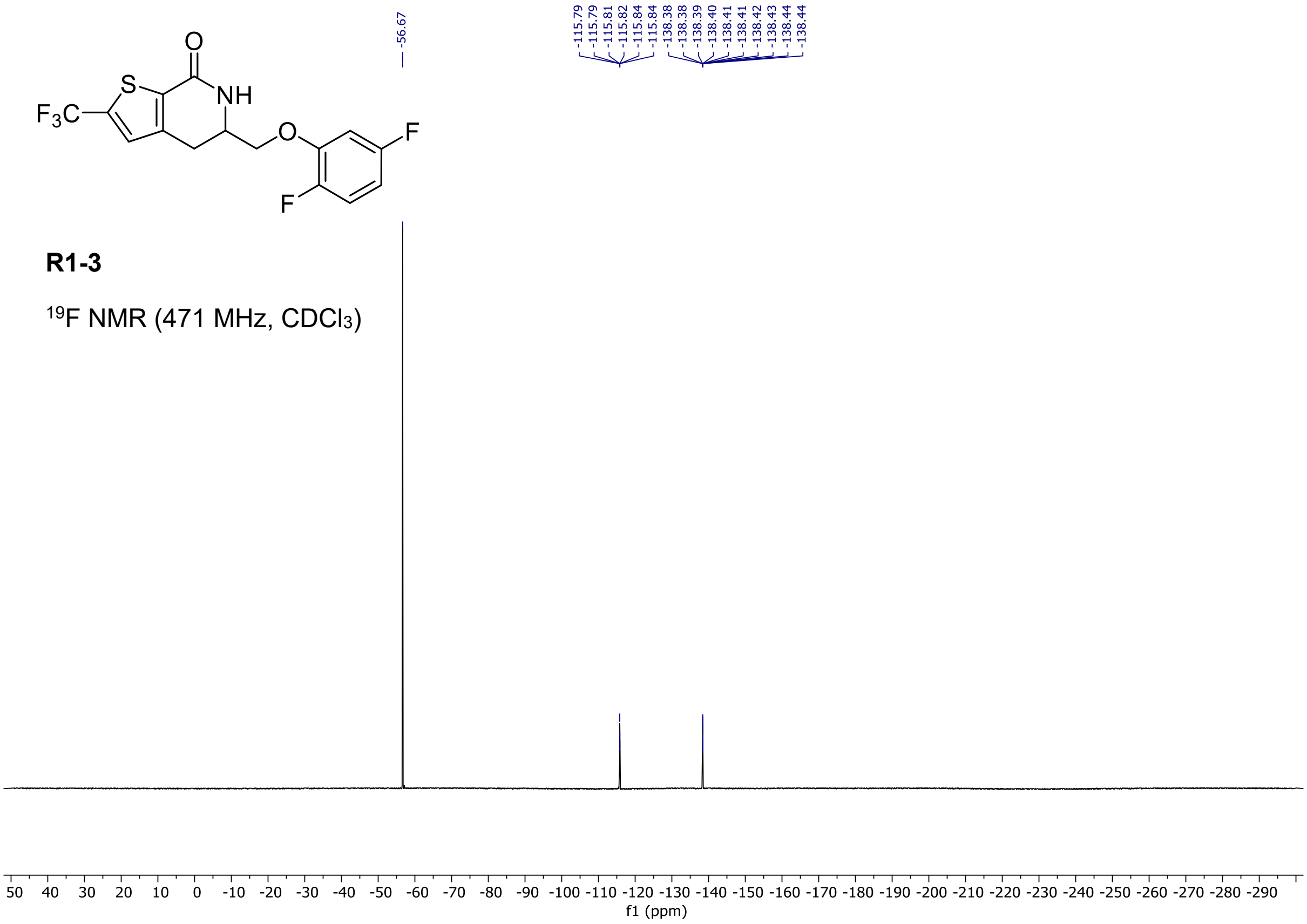

R1-3

^{19}F NMR (471 MHz, $CDCl_3$)

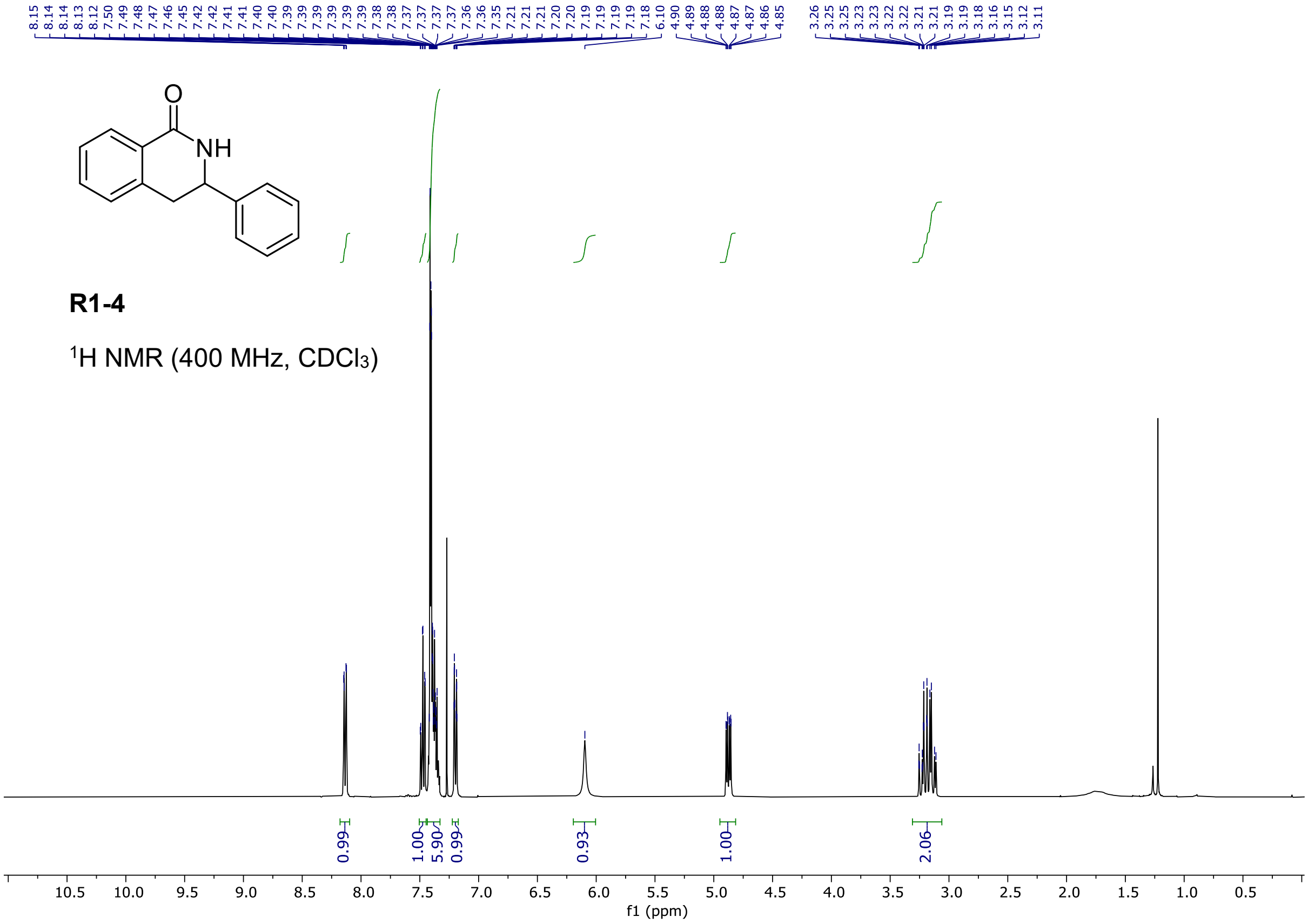

R1-4

^{1}H NMR (400 MHz, $CDCl_3$)

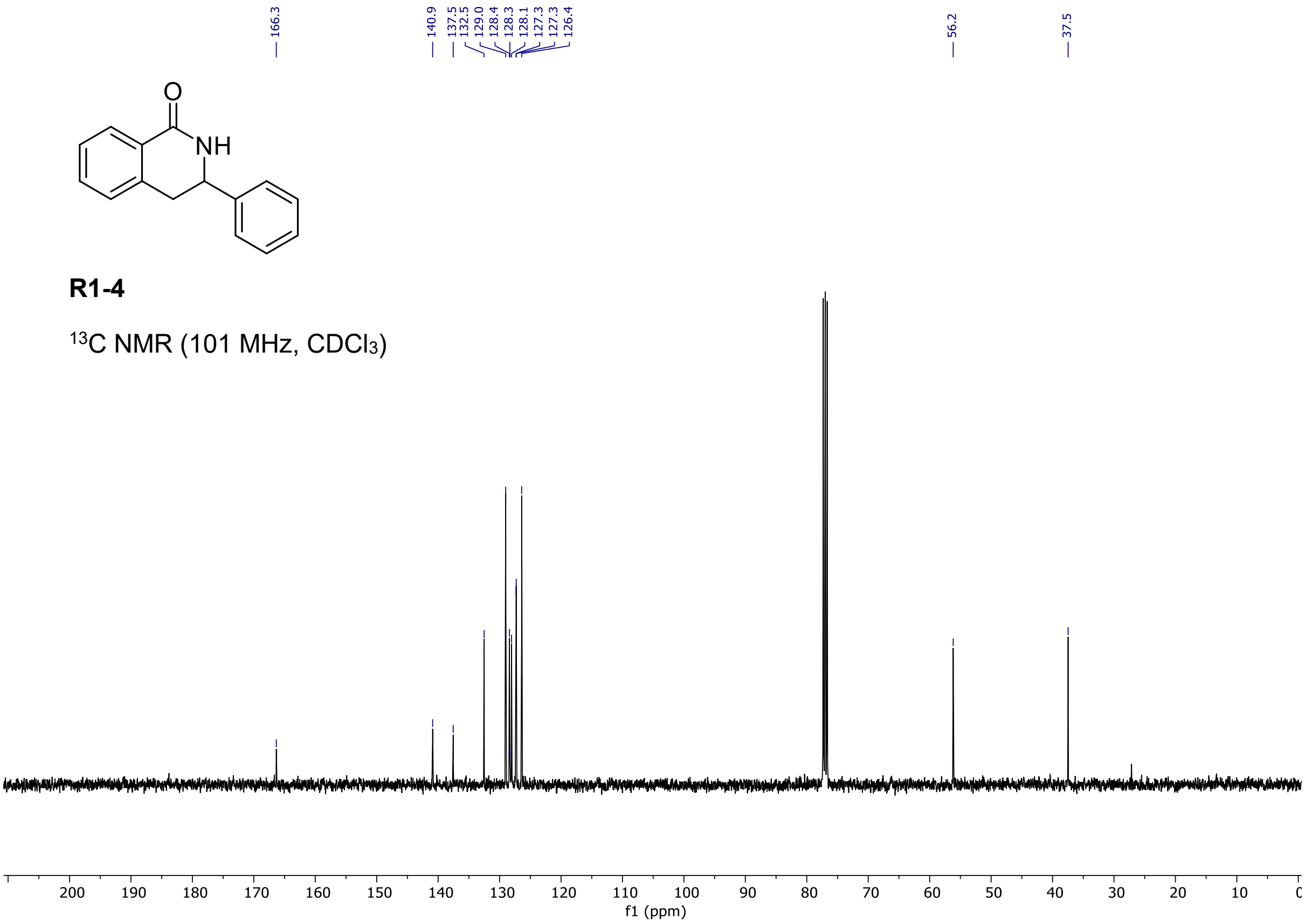

R1-4

^{13}C NMR (101 MHz, $CDCl_3$)

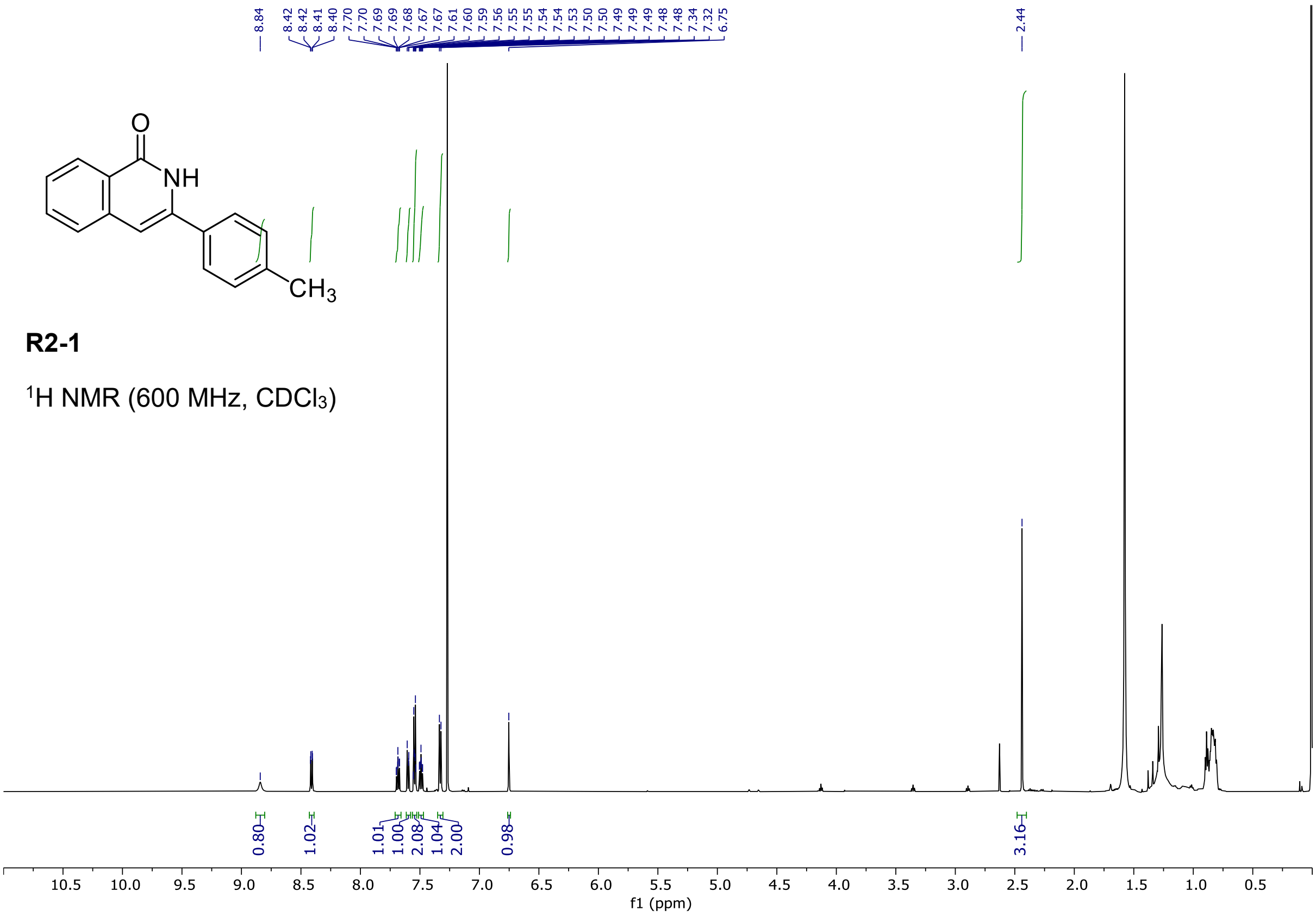
R2-1
1H NMR (600 MHz, CDCl3)
f1 (ppm)

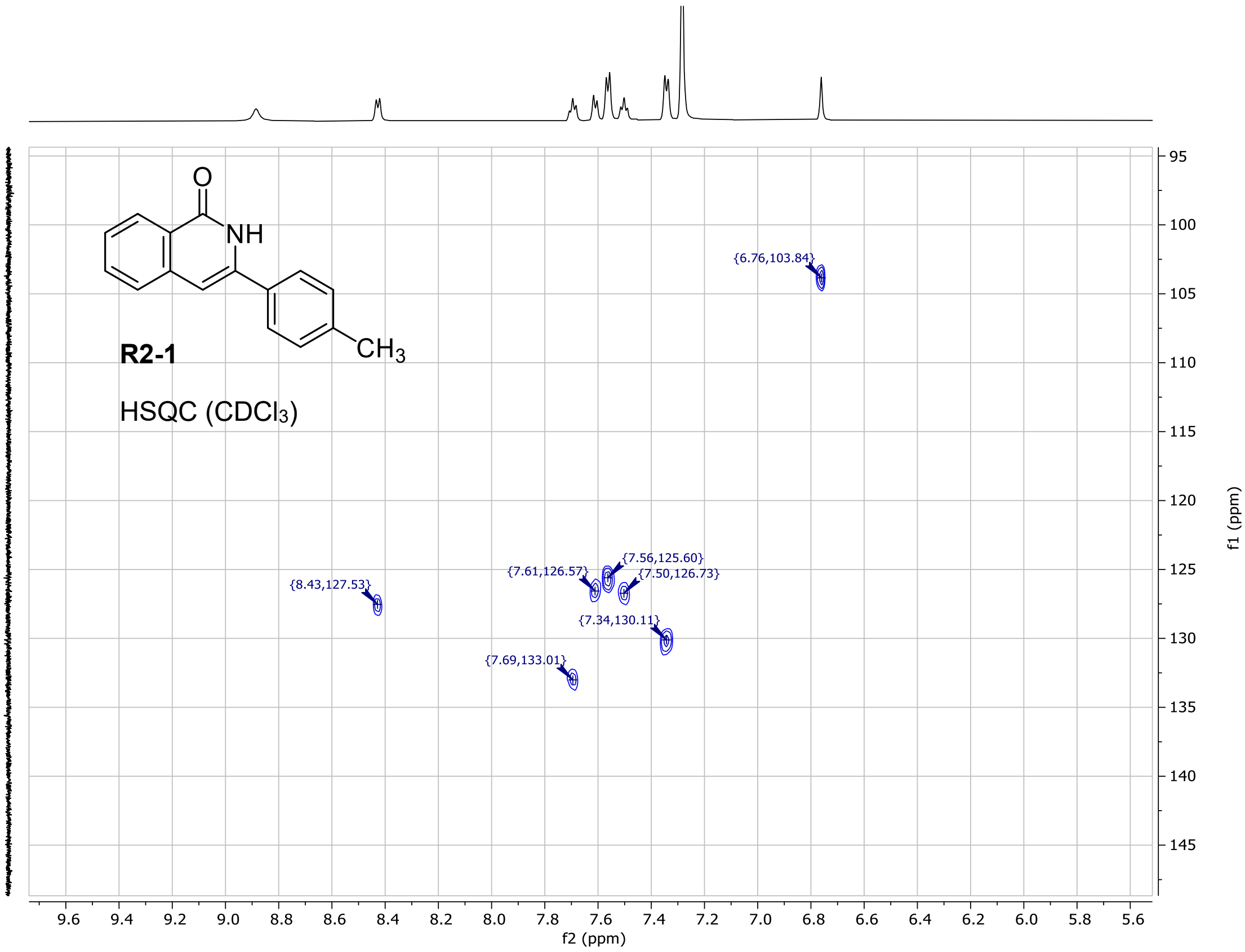
R2-1
HSQC (CDCl3)
{6.76,103.84}
{7.56,125.60}
{7.61,126.57}
{7.50,126.73}
{8.43,127.53}
{7.34,130.11}
{7.69,133.01}
f2 (ppm)
f1 (ppm)

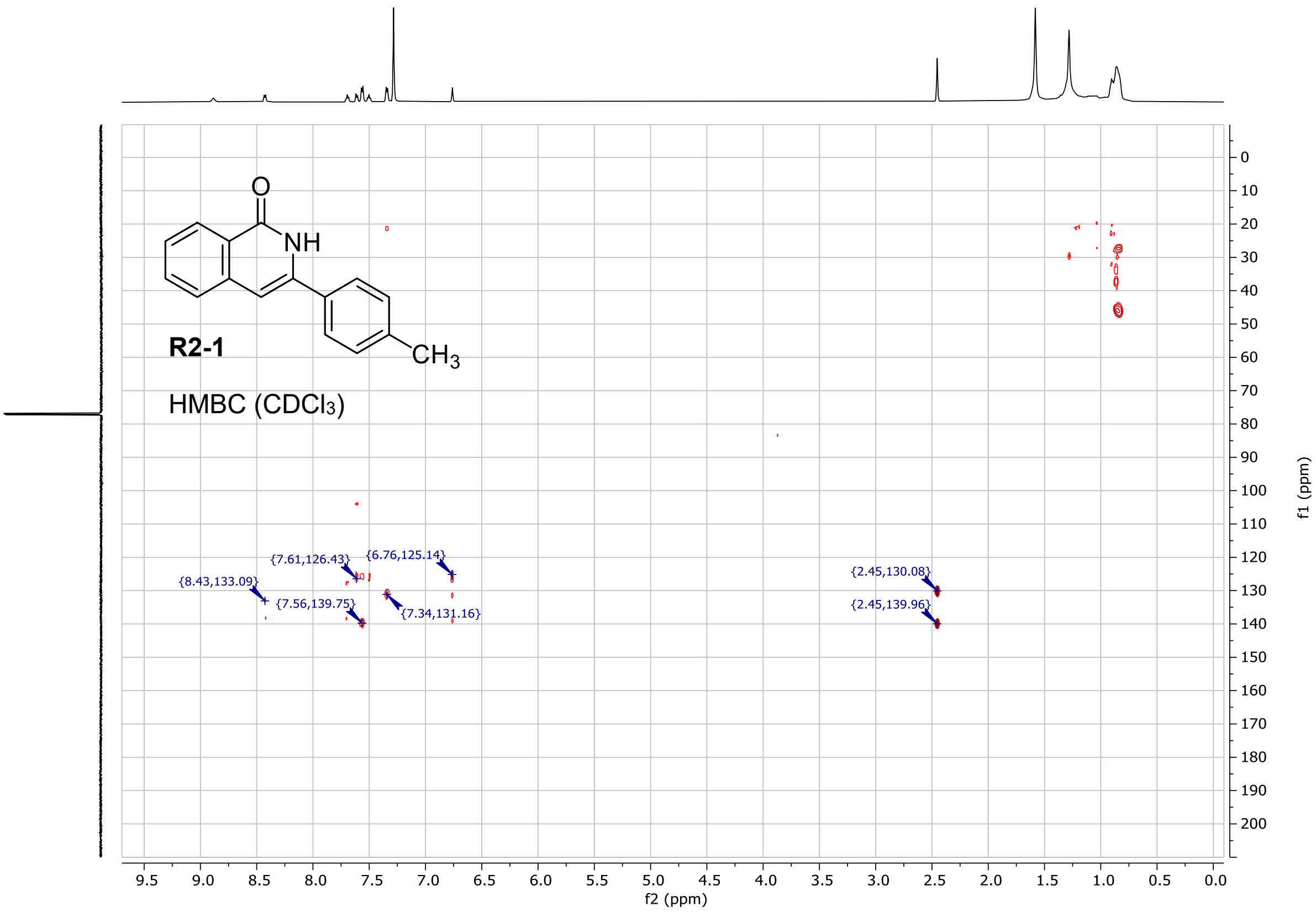
O
NH
CH3
R2-1
HMBC (CDCl3)
{7.61,126.43}
{6.76,125.14}
{8.43,133.09}
{7.56,139.75}
{7.34,131.16}
{2.45,130.08}
{2.45,139.96}
f2 (ppm)
f1 (ppm)

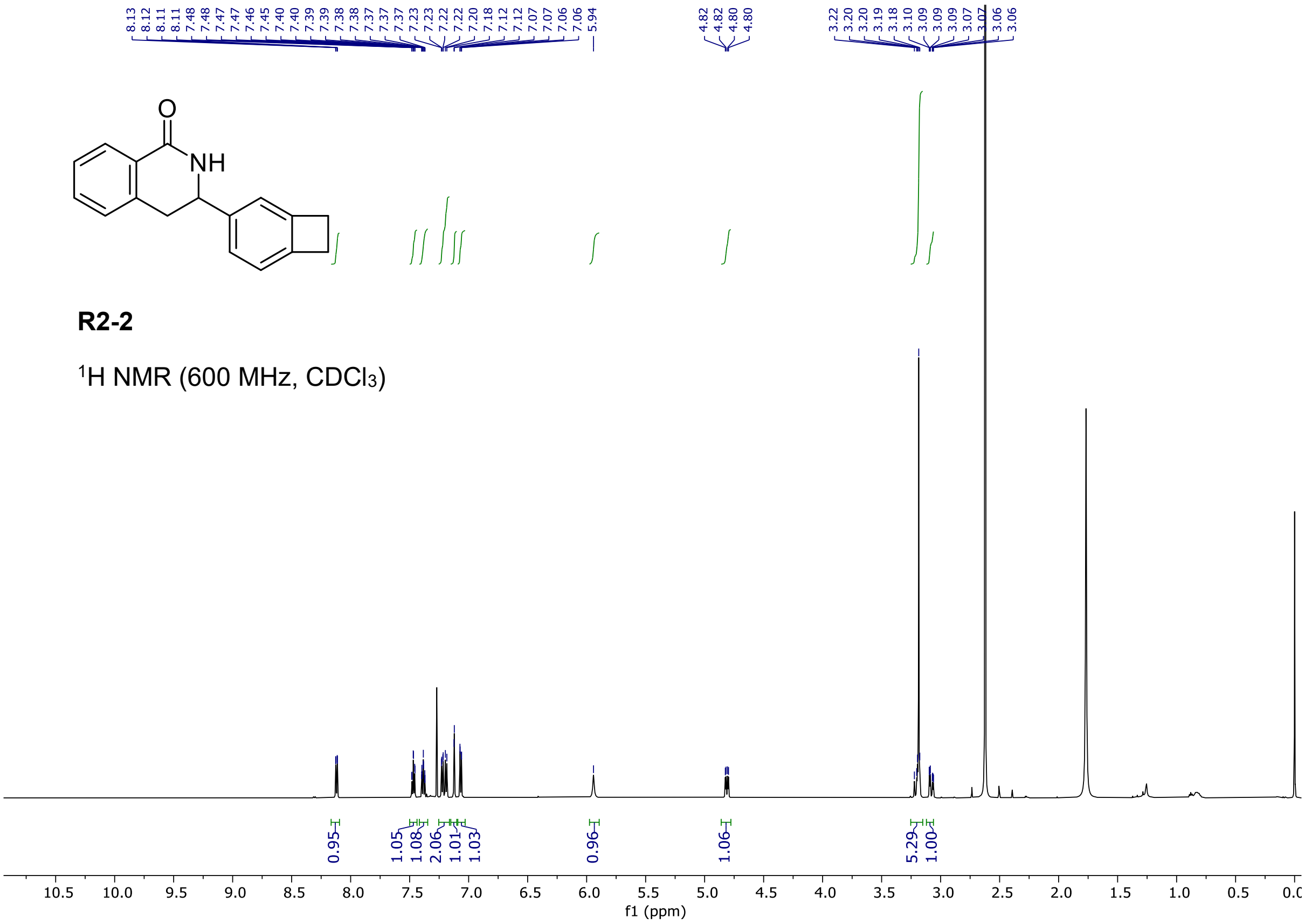

R2-2

^{1}H NMR (600 MHz, $CDCl_3$)

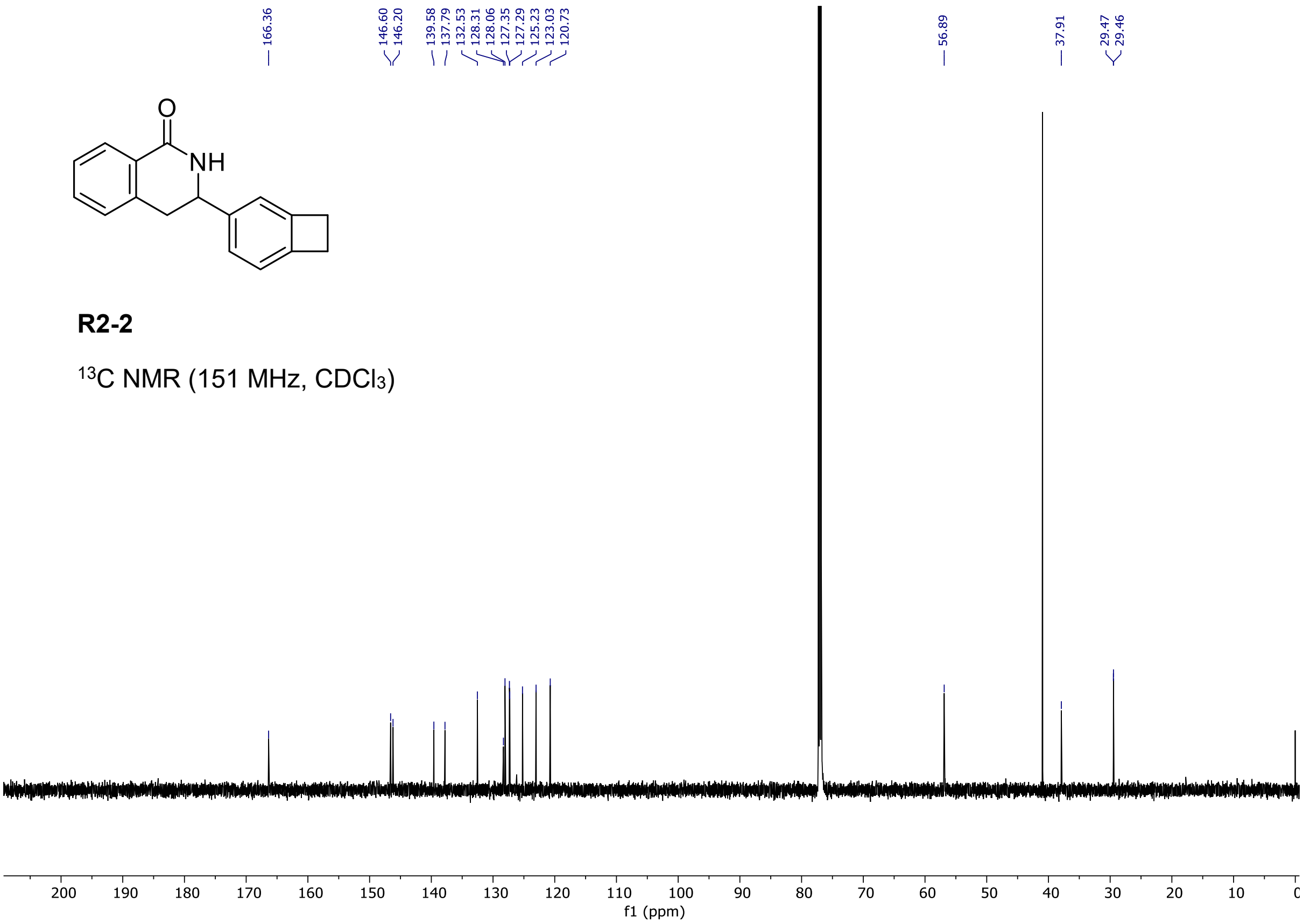

R2-2

^{13}C NMR (151 MHz, $CDCl_3$)

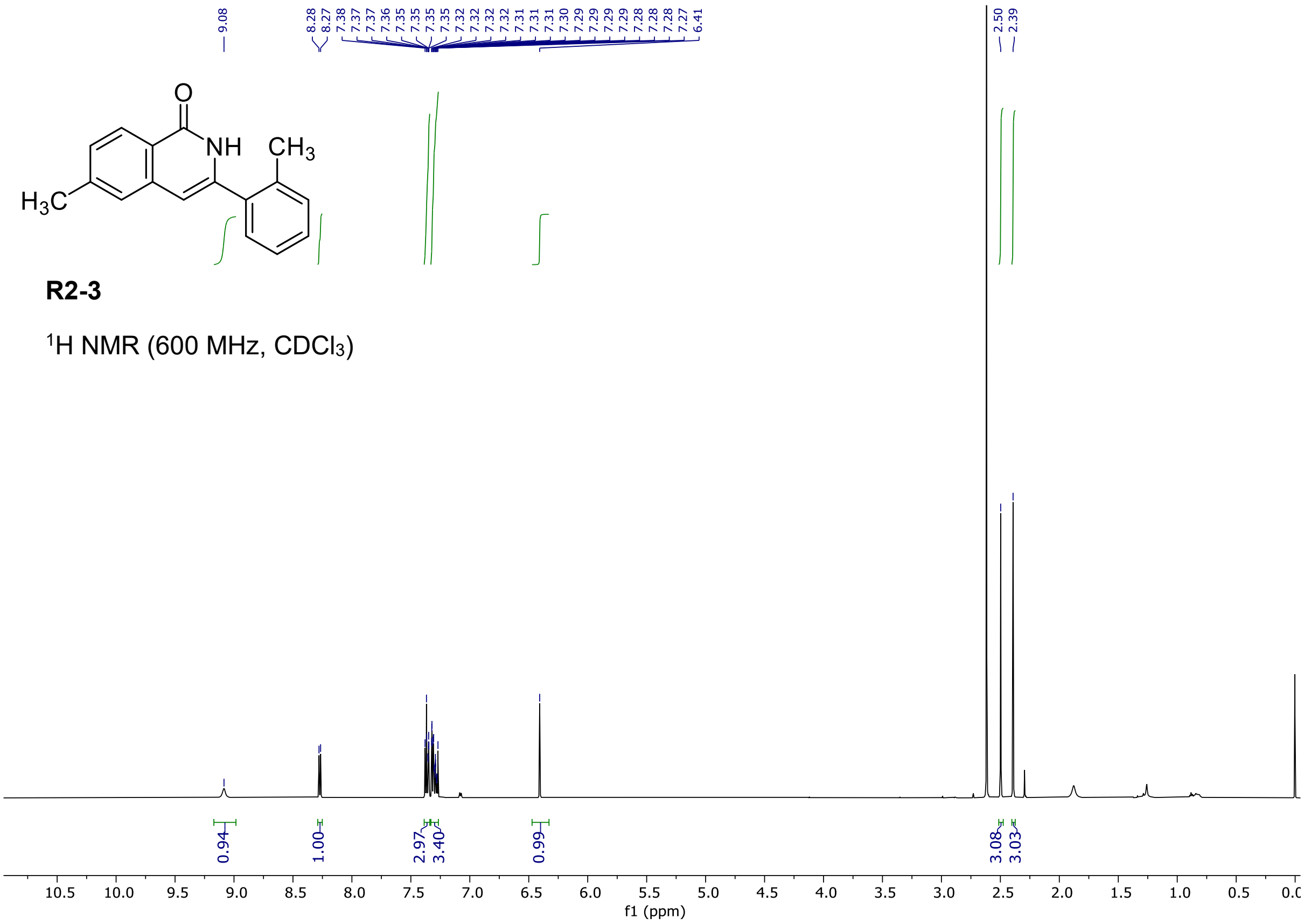

R2-3

^{1}H NMR (600 MHz, $CDCl_3$)

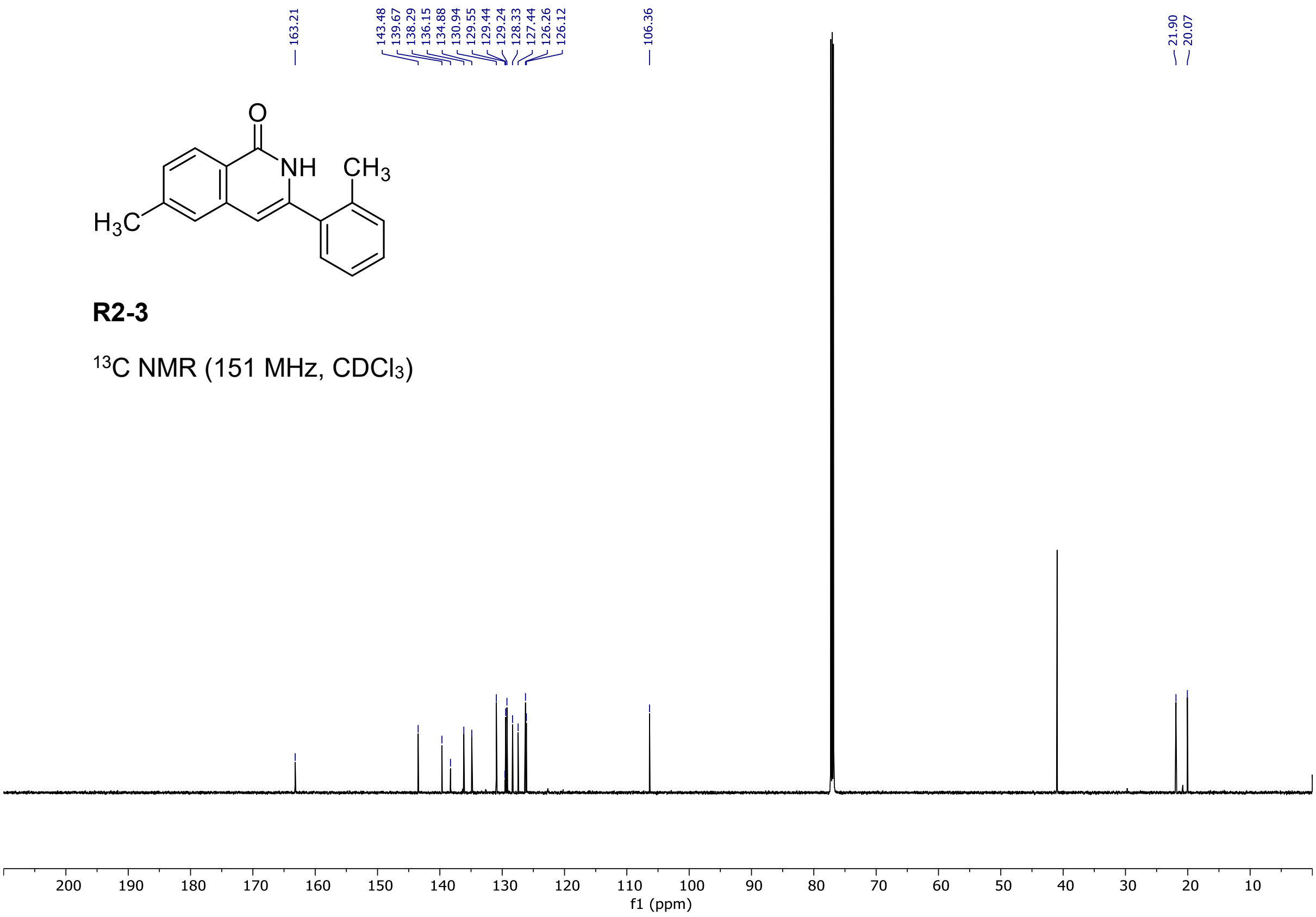

R2-3

^{13}C NMR (151 MHz, $CDCl_3$)

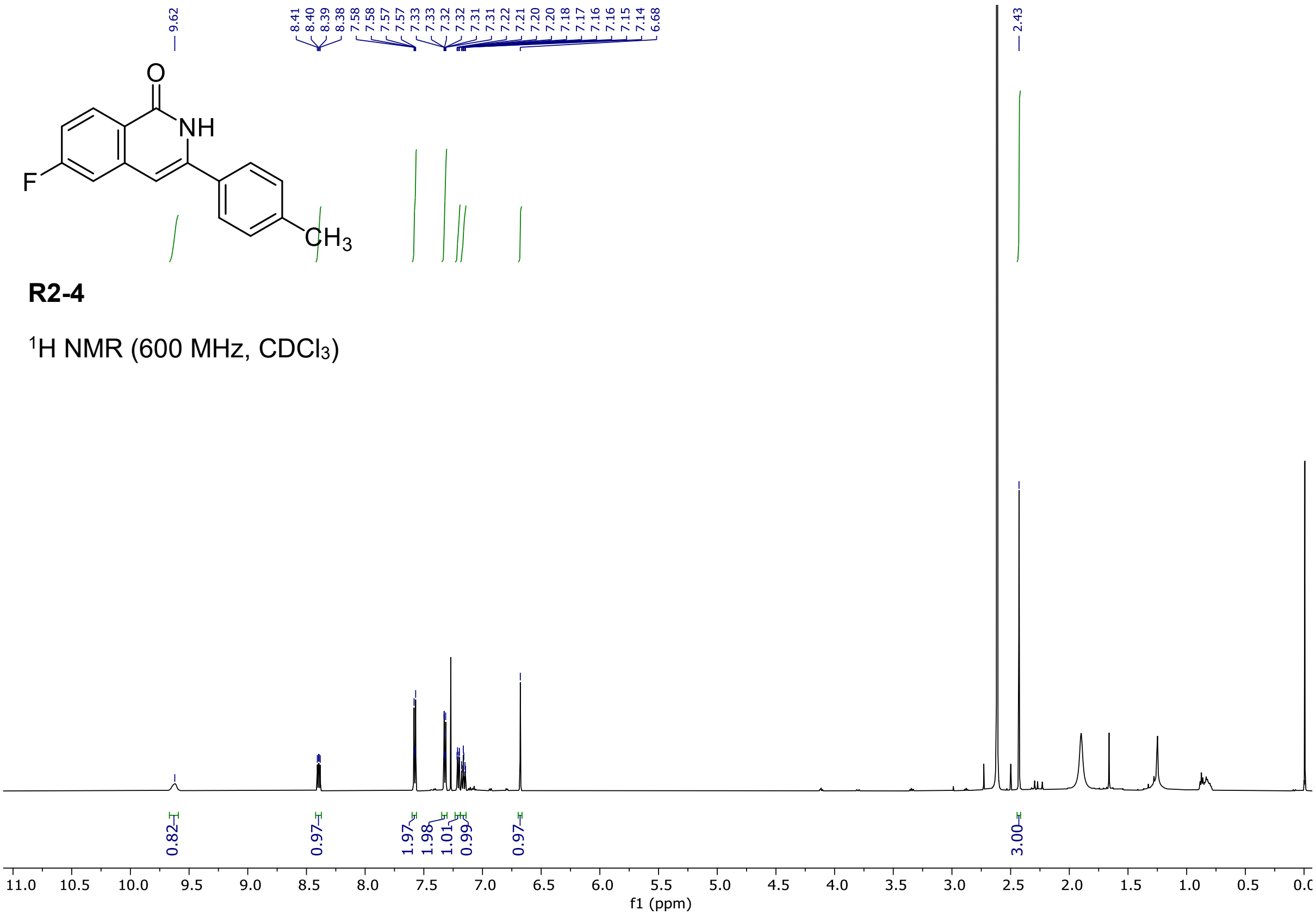

R2-4

^{1}H NMR (600 MHz, $CDCl_3$)

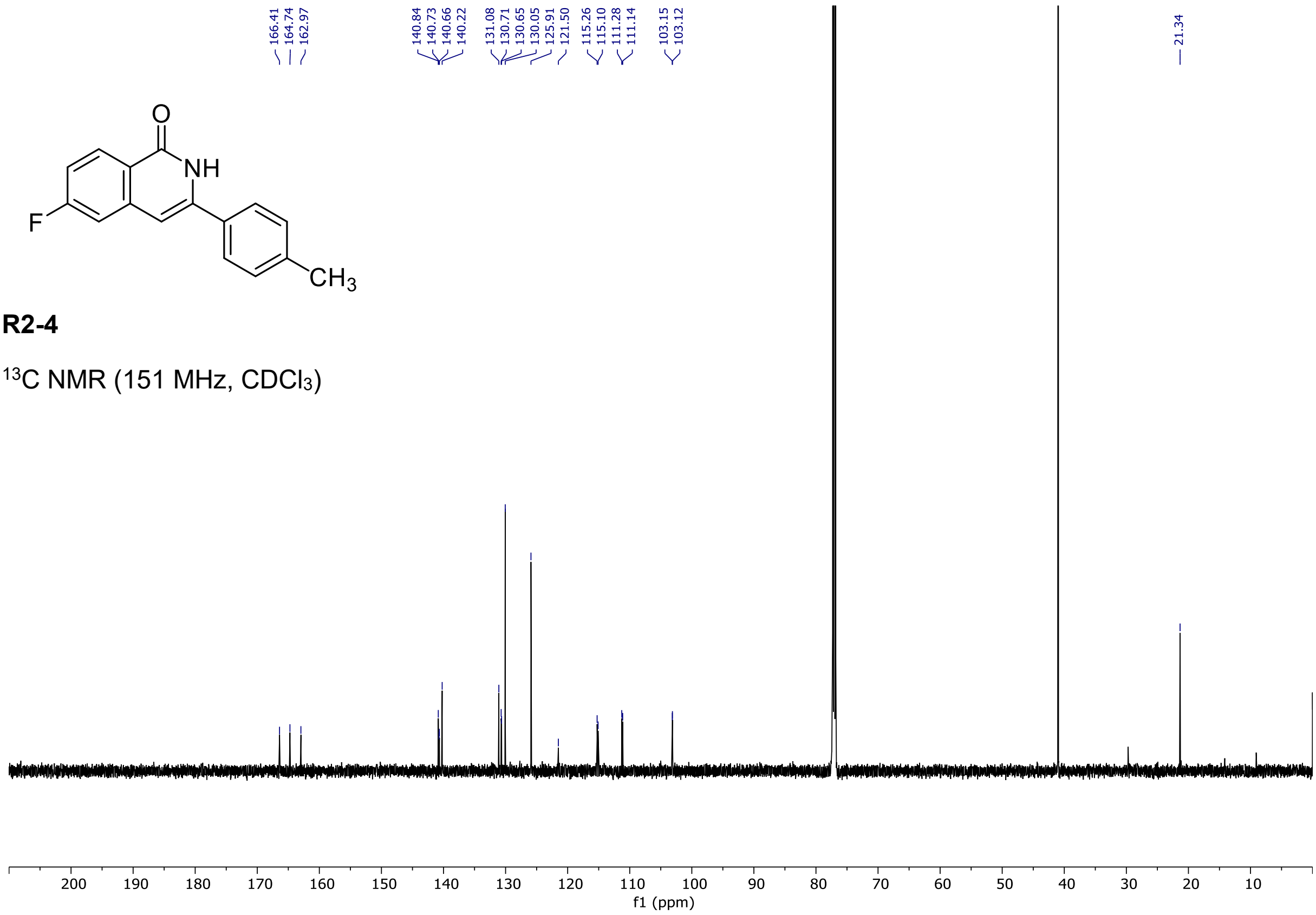

R2-4

^{13}C NMR (151 MHz, $CDCl_3$)

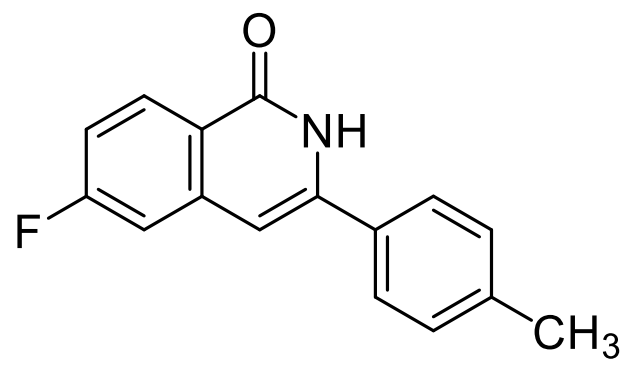

R2-4

^{19}F NMR (376 MHz, $CDCl_3$)

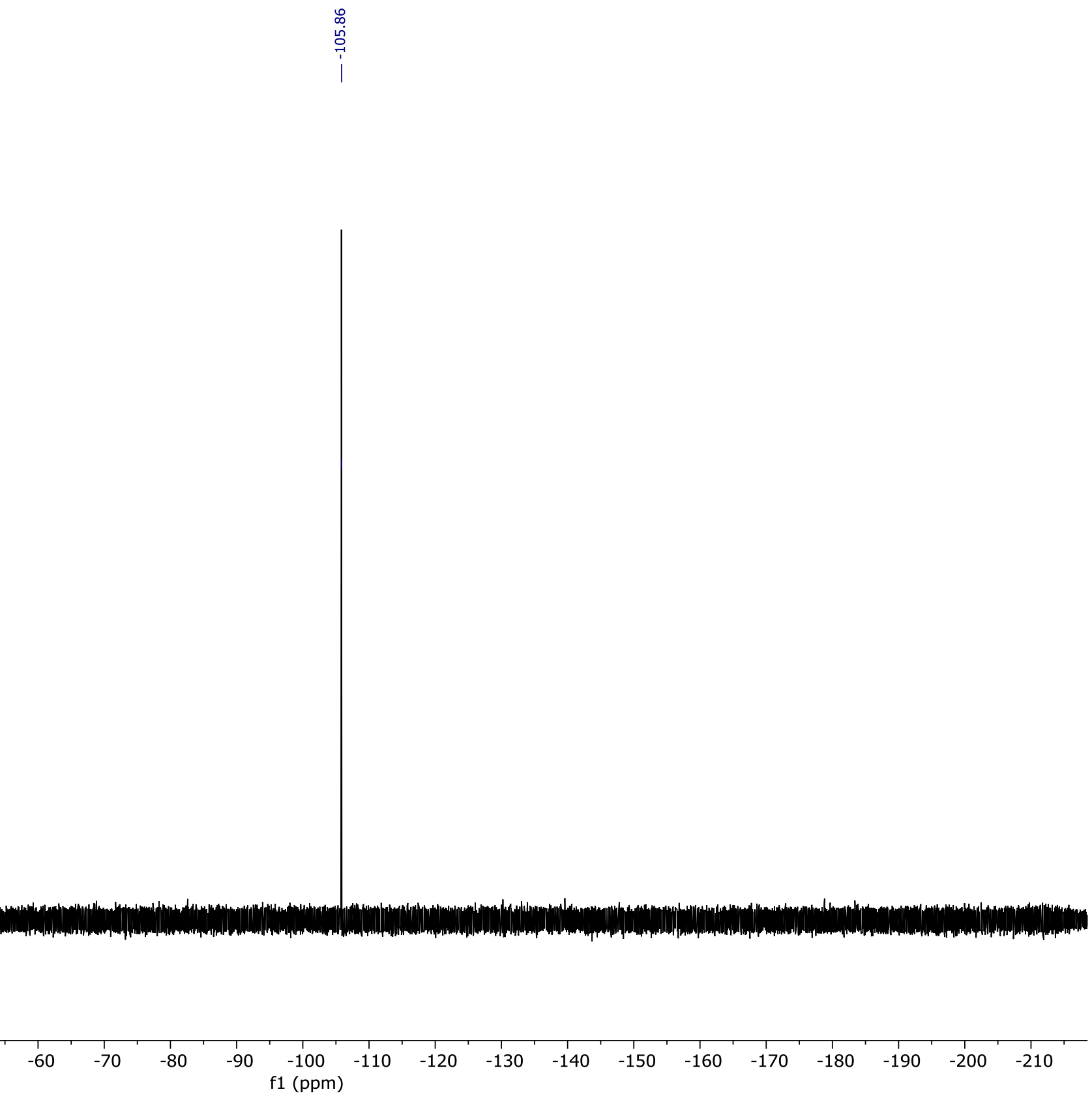

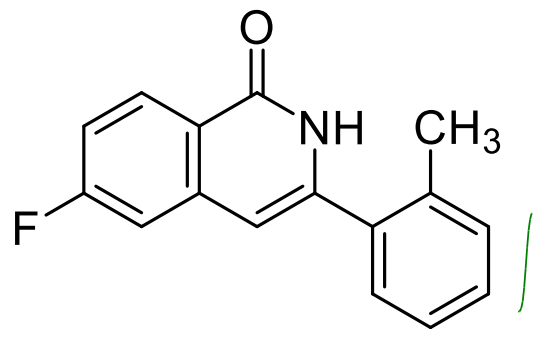

R2-5

^{1}H NMR (600 MHz, $CDCl_3$)

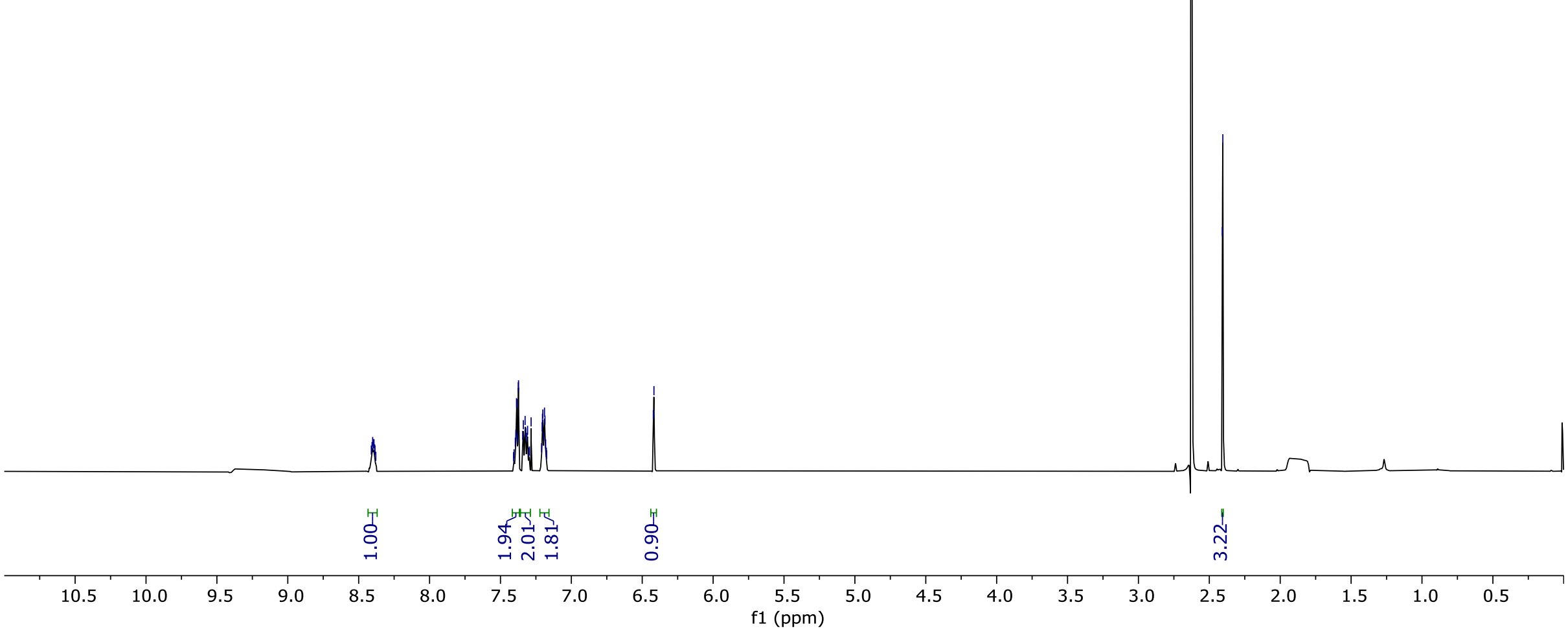

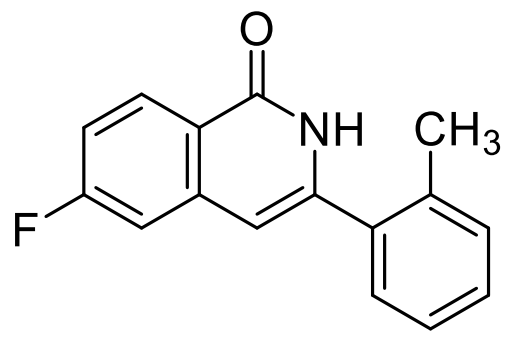

R2-5

^{13}C NMR (151 MHz, $CDCl_3$)

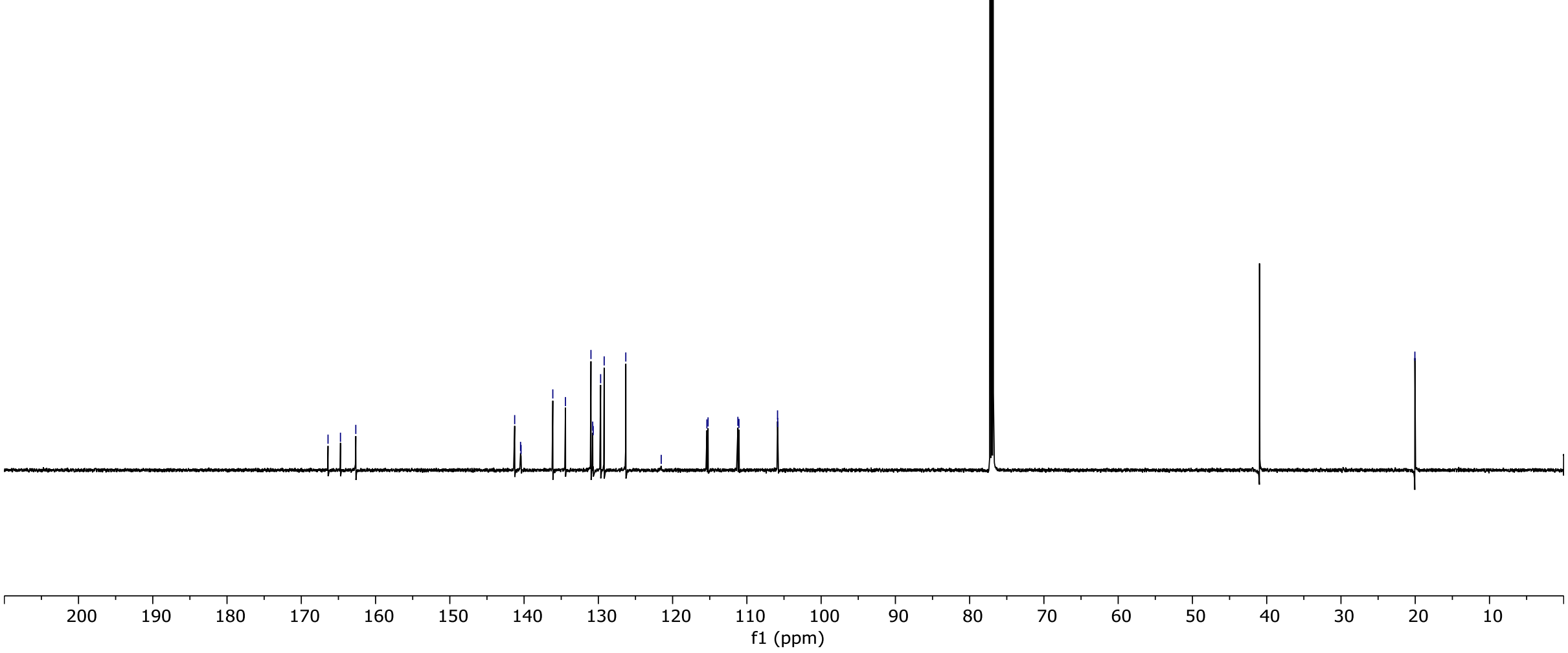

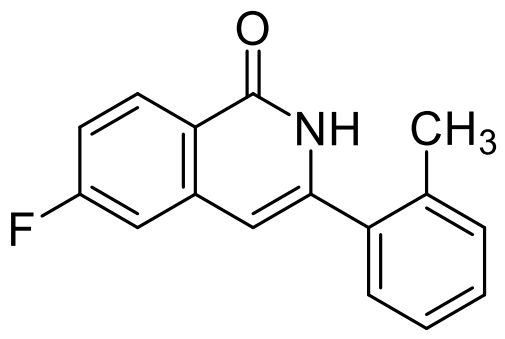

R2-5

^{19}F NMR (376 MHz, $CDCl_3$)

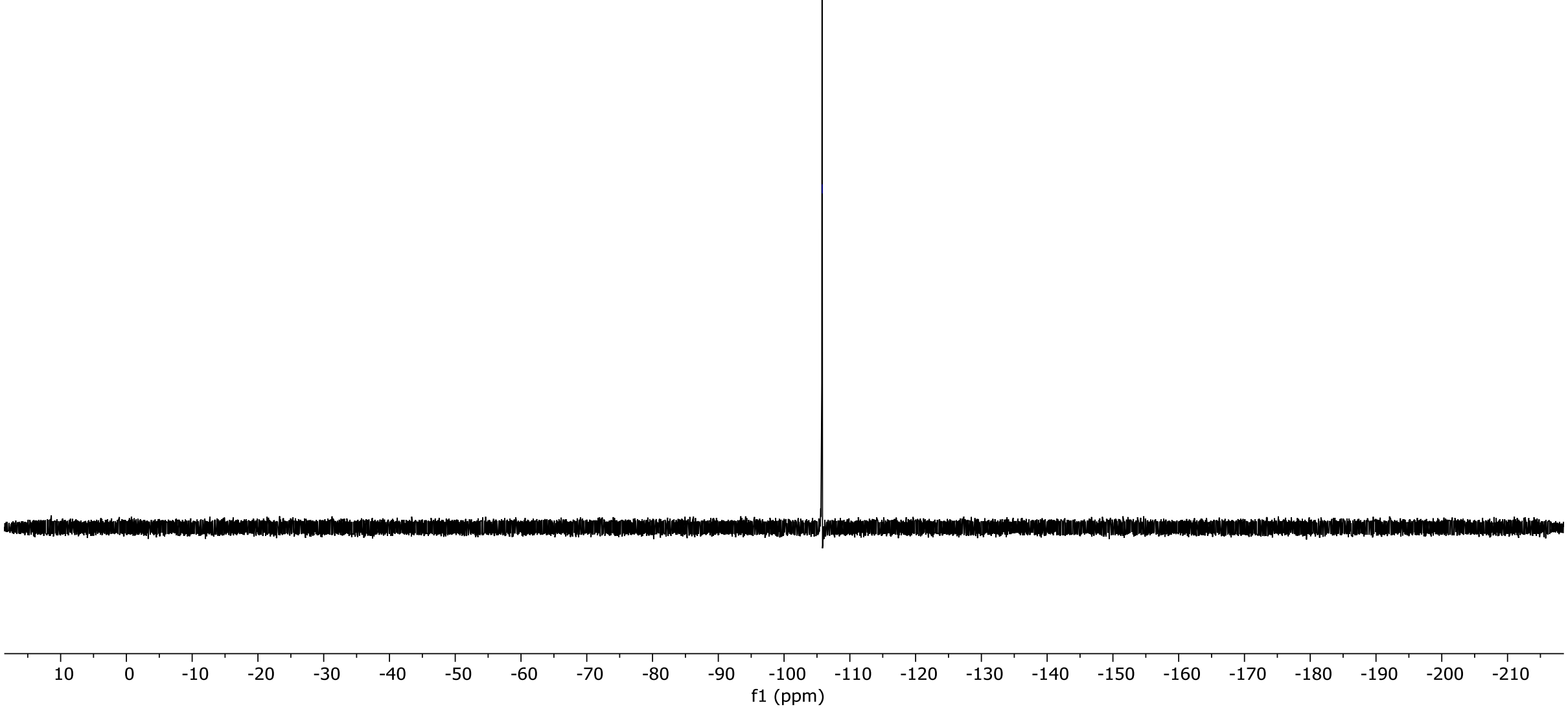

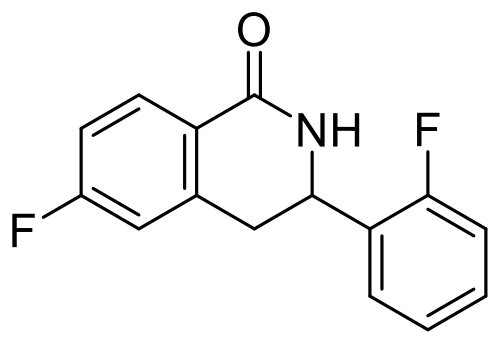

R2-6

^{1}H NMR (600 MHz, $CDCl_3$)

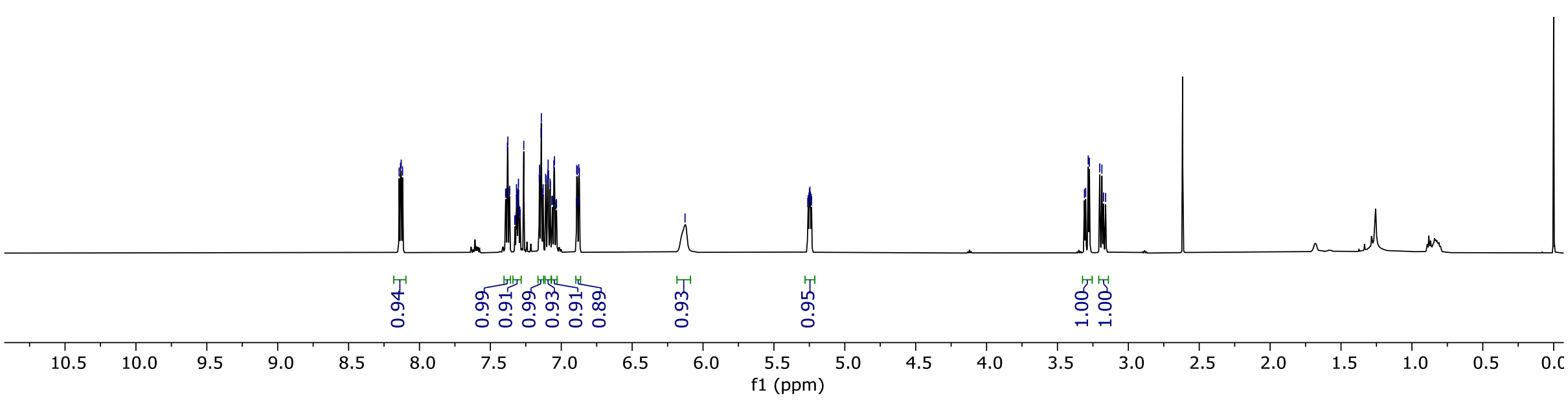

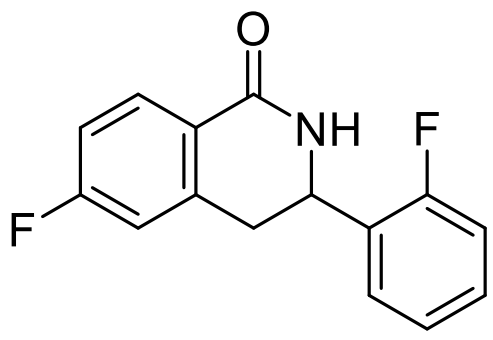

R2-6

^{13}C NMR (151 MHz, $CDCl_3$)

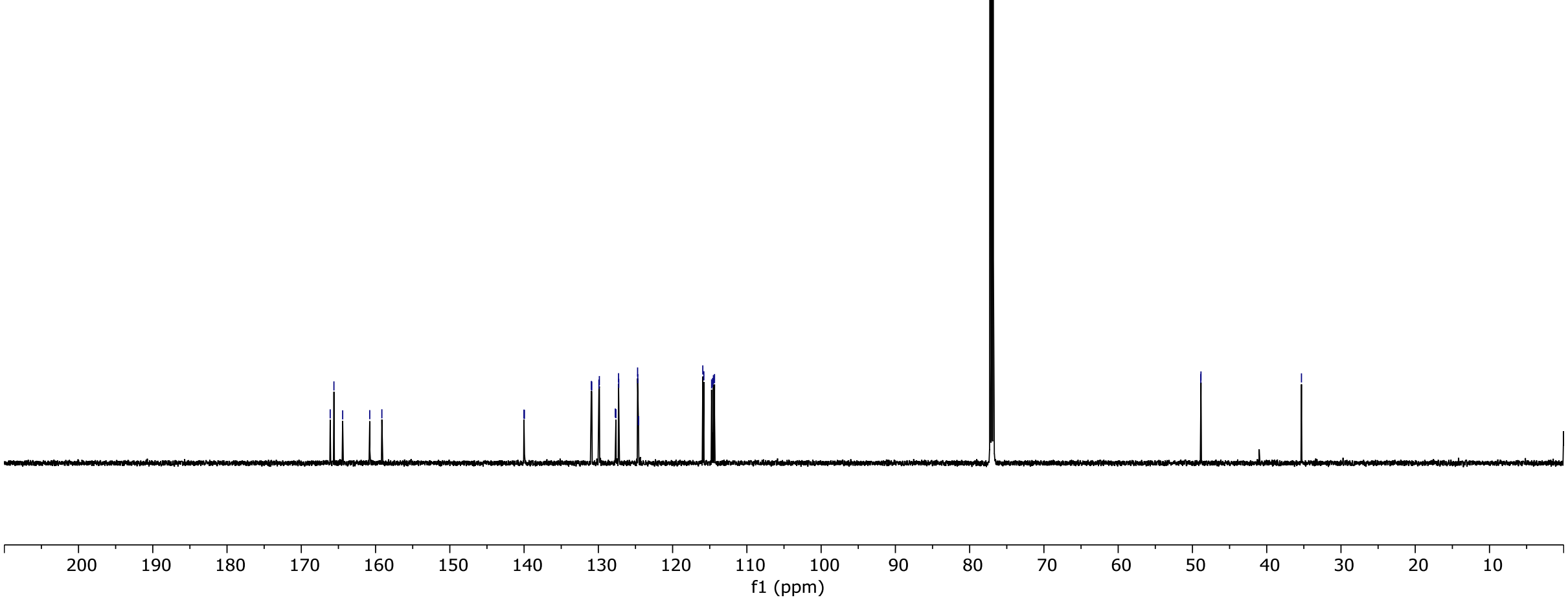

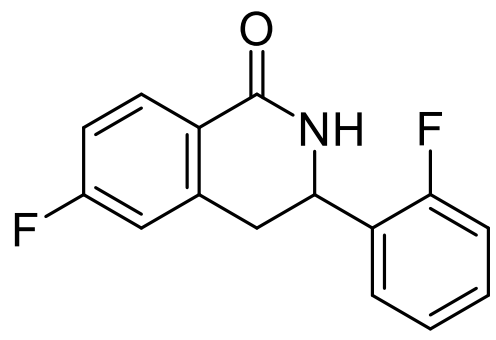

R2-6

^{19}F NMR (376 MHz, $CDCl_3$)

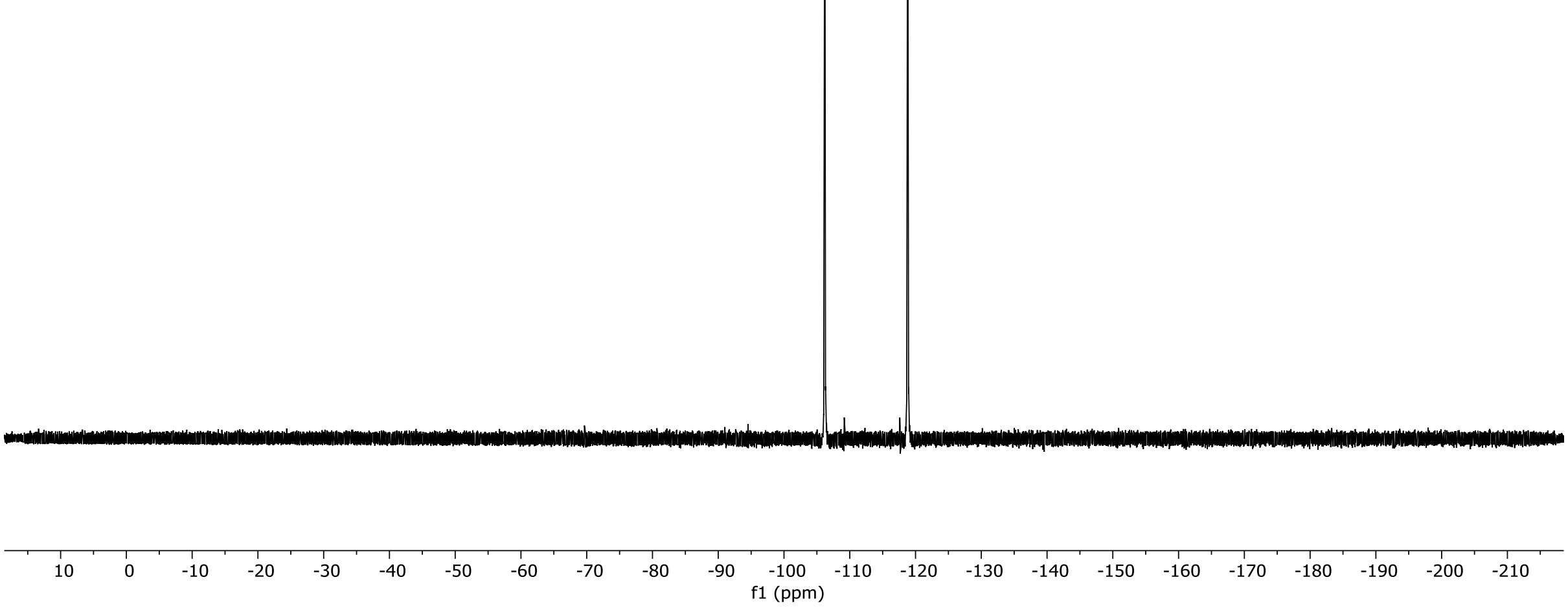

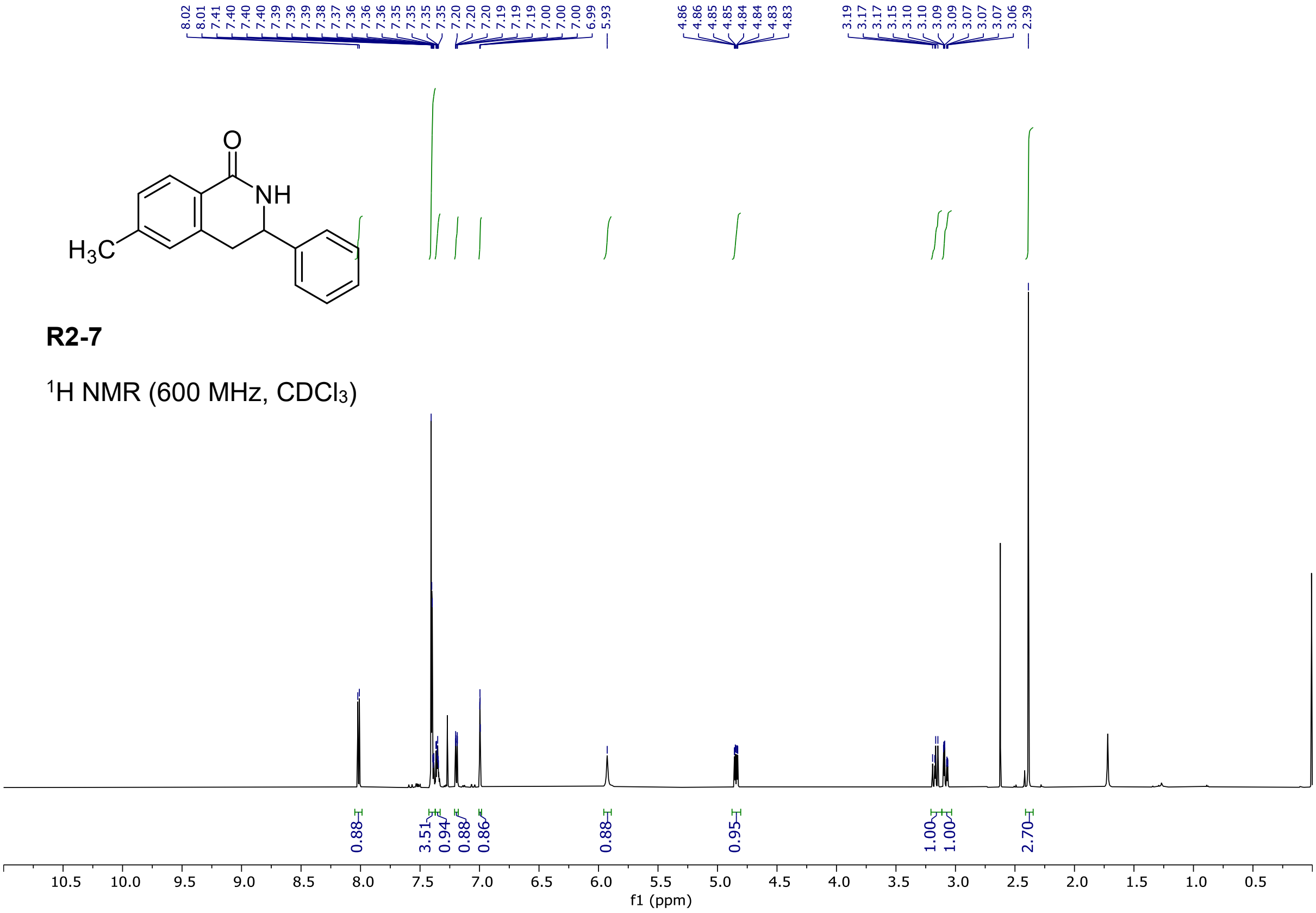

R2-7

^{1}H NMR (600 MHz, $CDCl_3$)

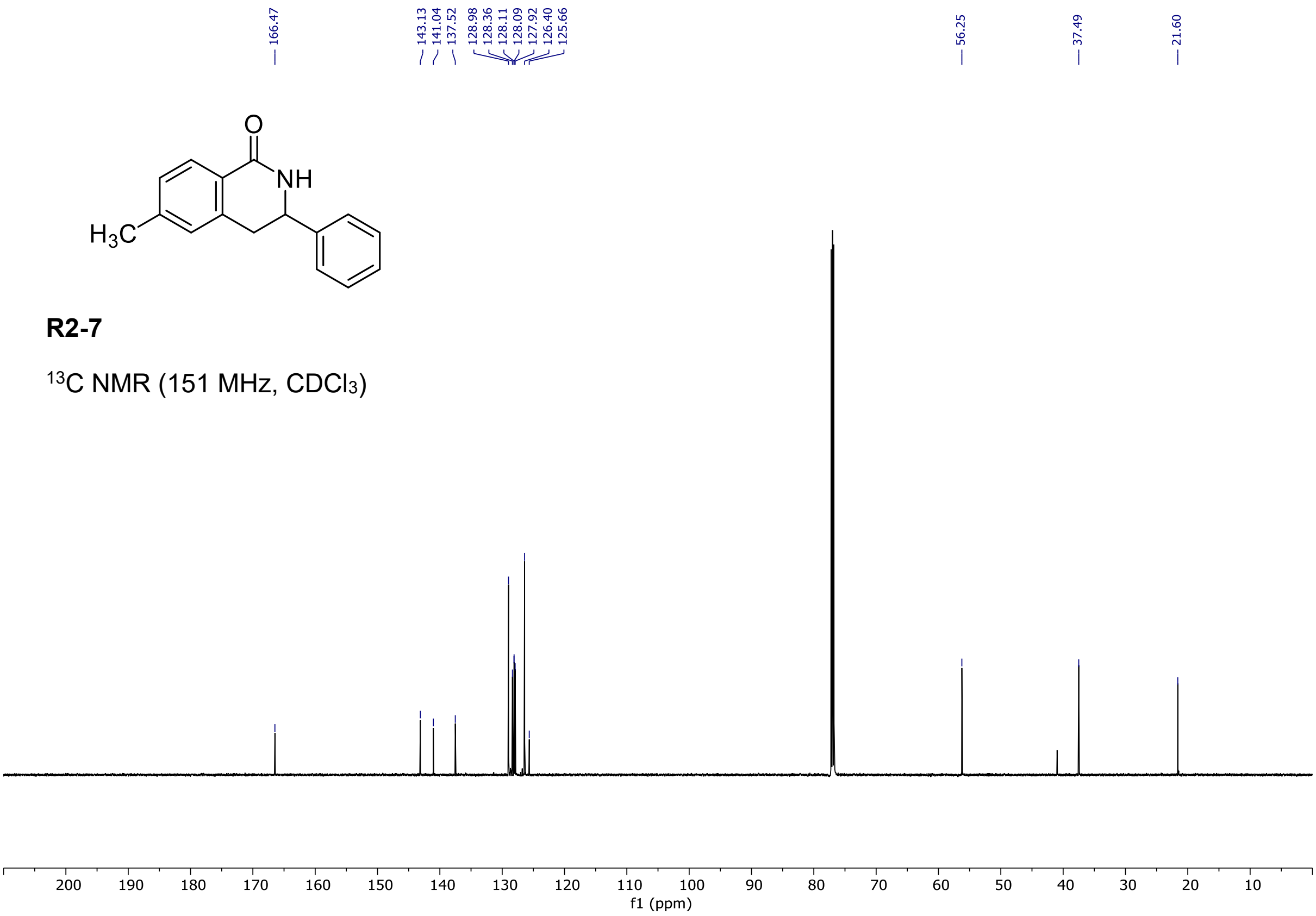

R2-7

^{13}C NMR (151 MHz, $CDCl_3$)

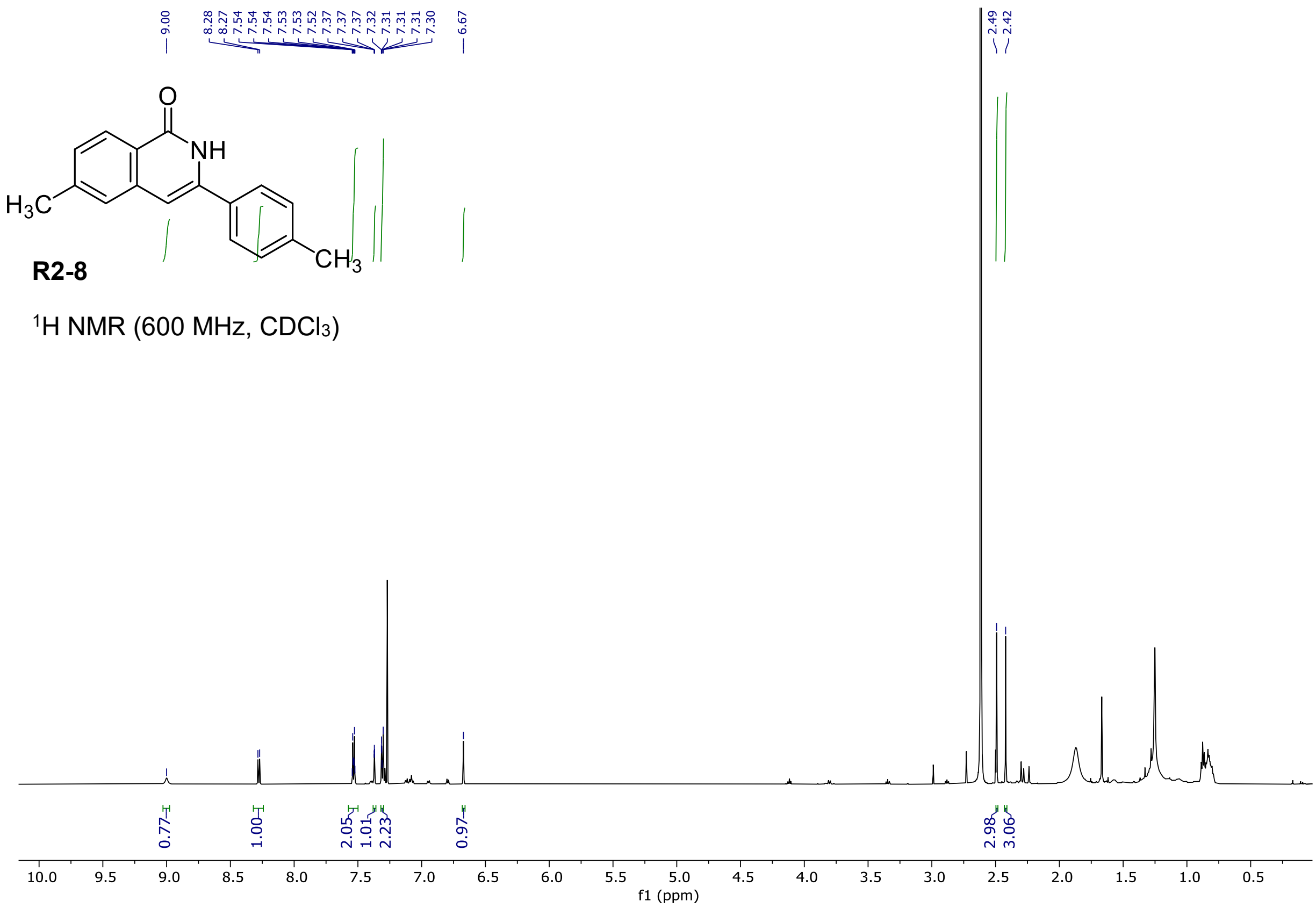

R2-8

^{1}H NMR (600 MHz, $CDCl_3$)

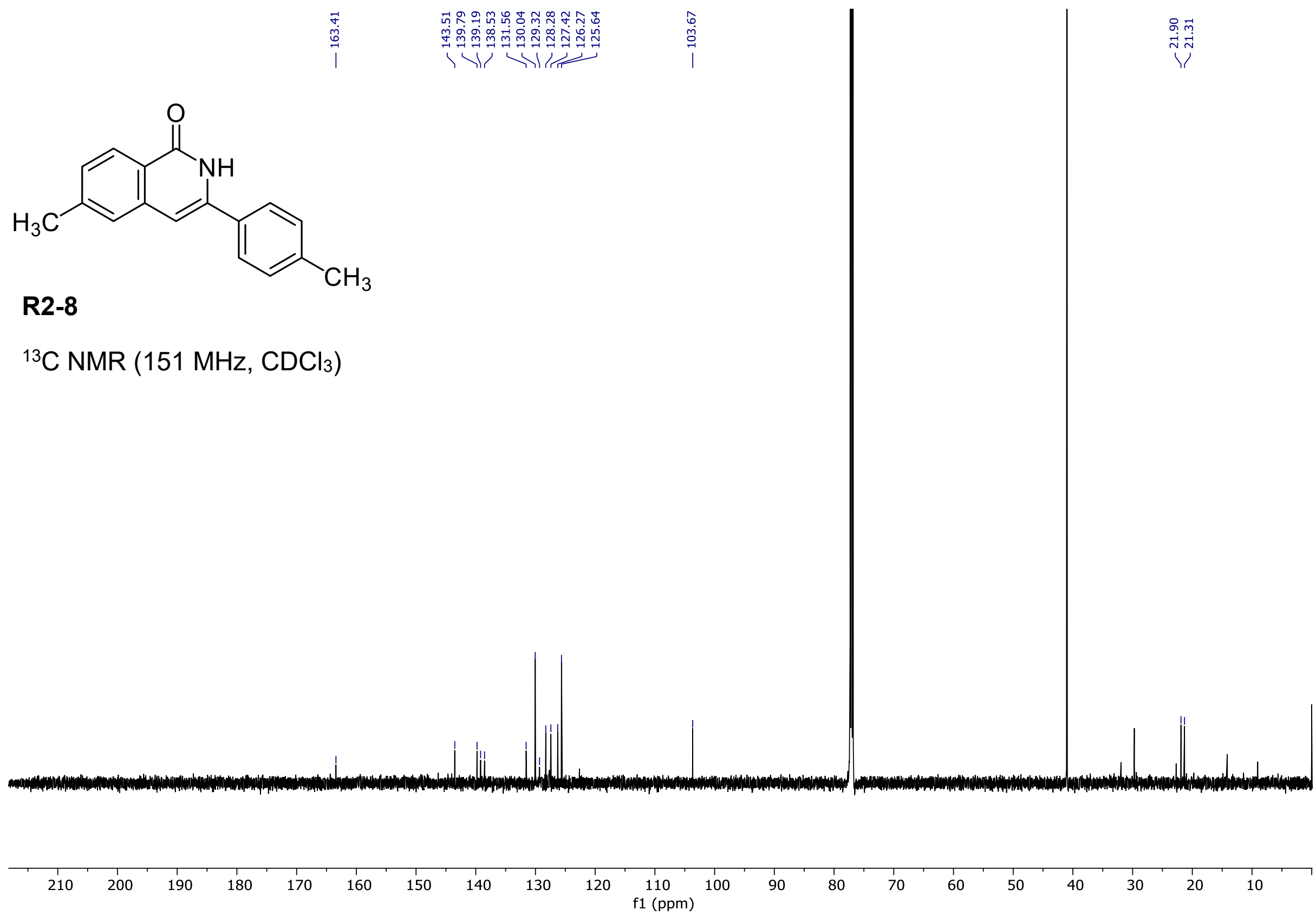

R2-8

^{13}C NMR (151 MHz, $CDCl_3$)

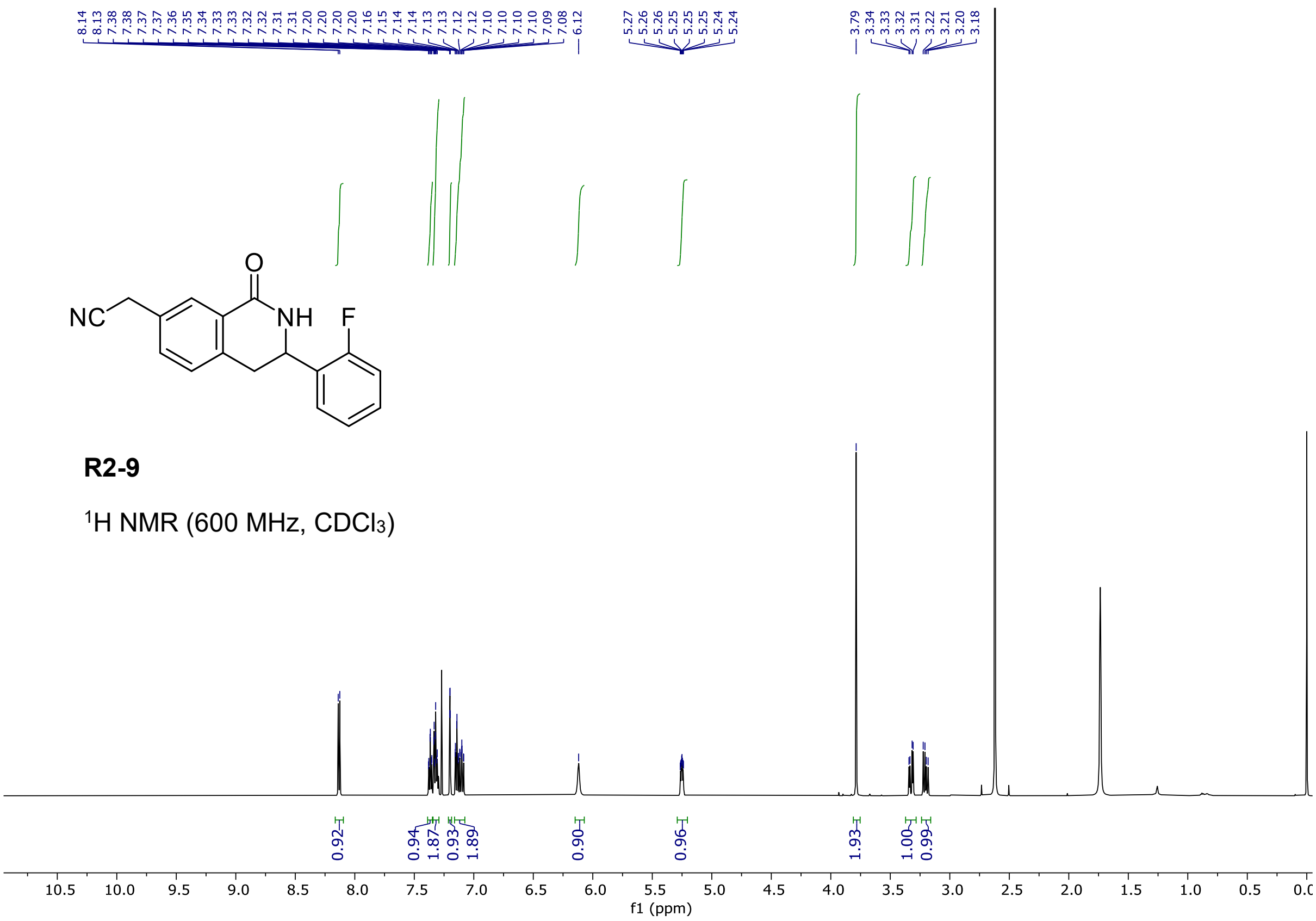

R2-9

^{1}H NMR (600 MHz, $CDCl_3$)

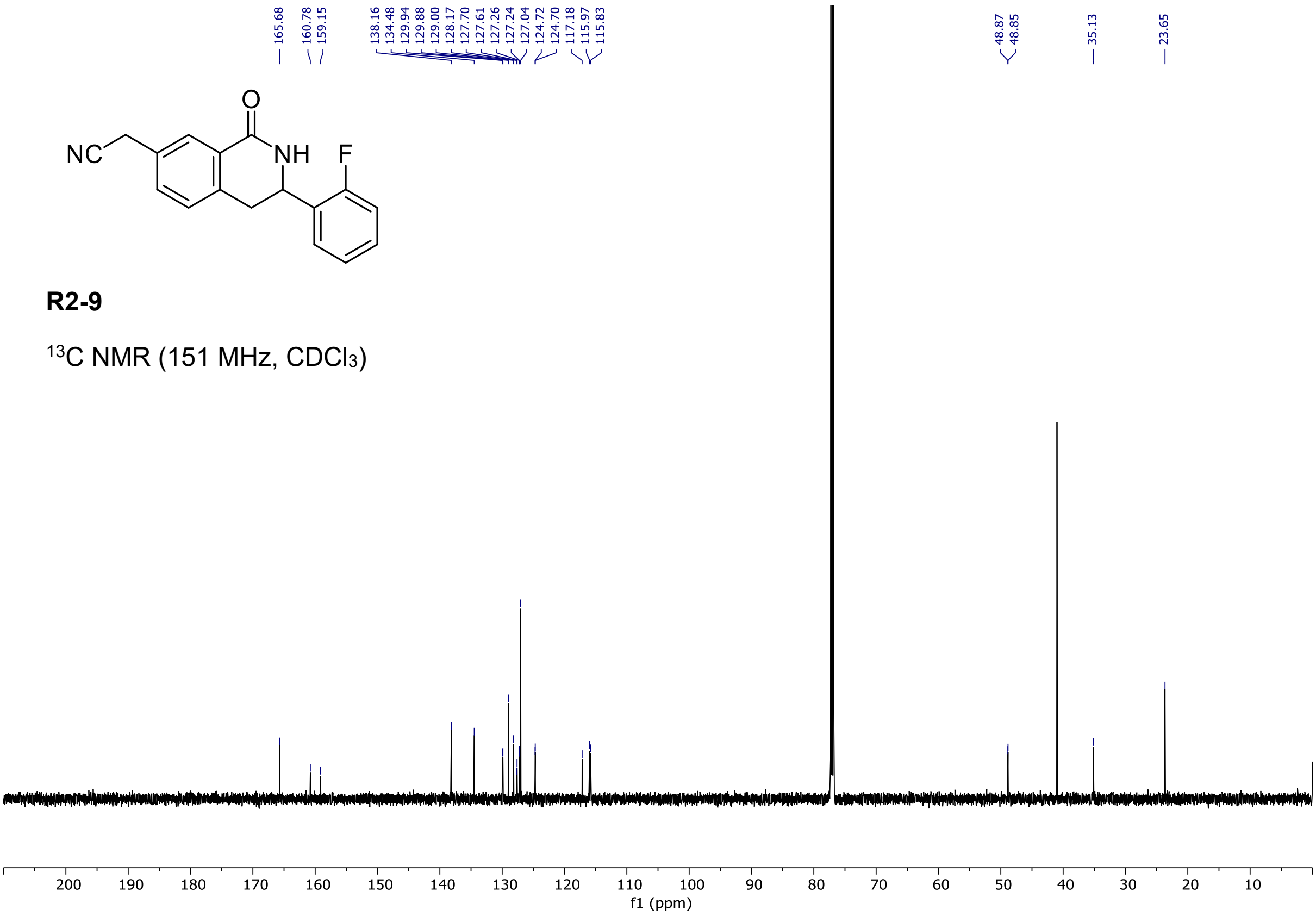

R2-9

^{13}C NMR (151 MHz, $CDCl_3$)

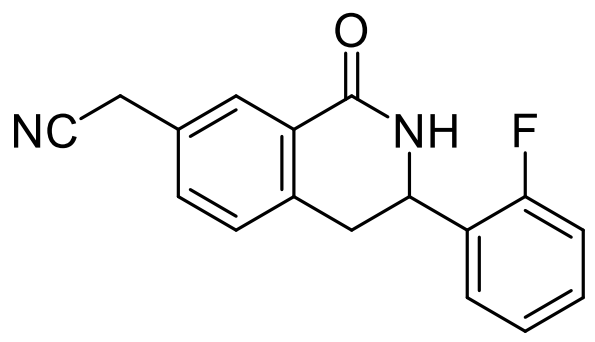

R2-9

^{19}F NMR (376 MHz, $CDCl_3$)

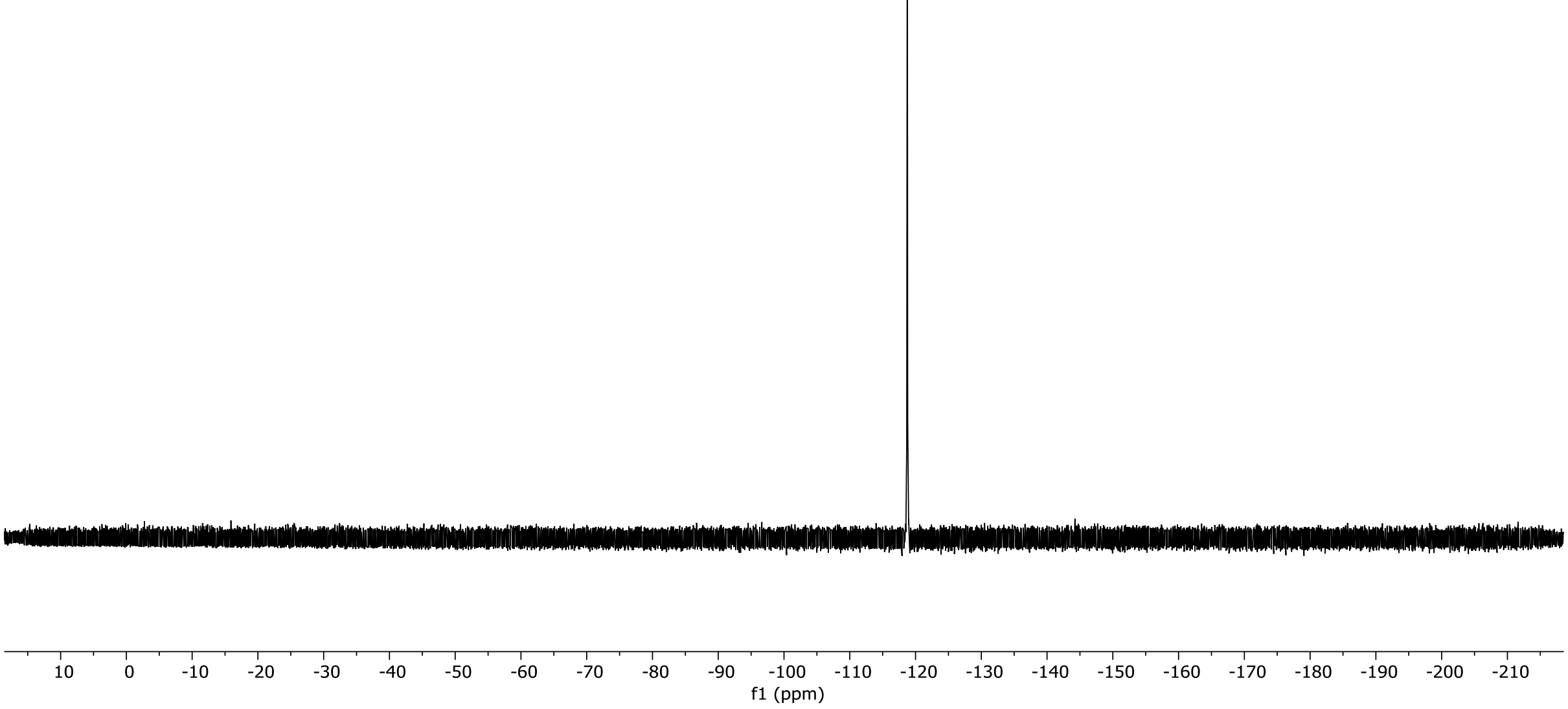

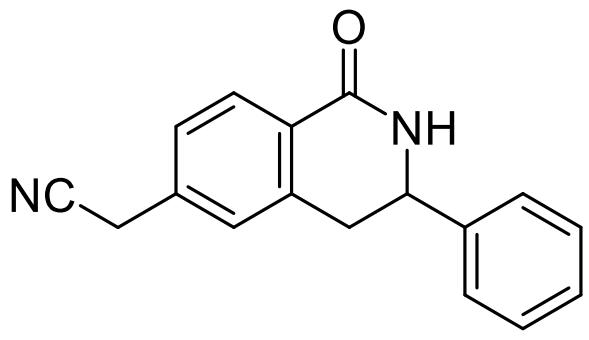

R2-10

^{1}H NMR (600 MHz, CDCl$_3$)

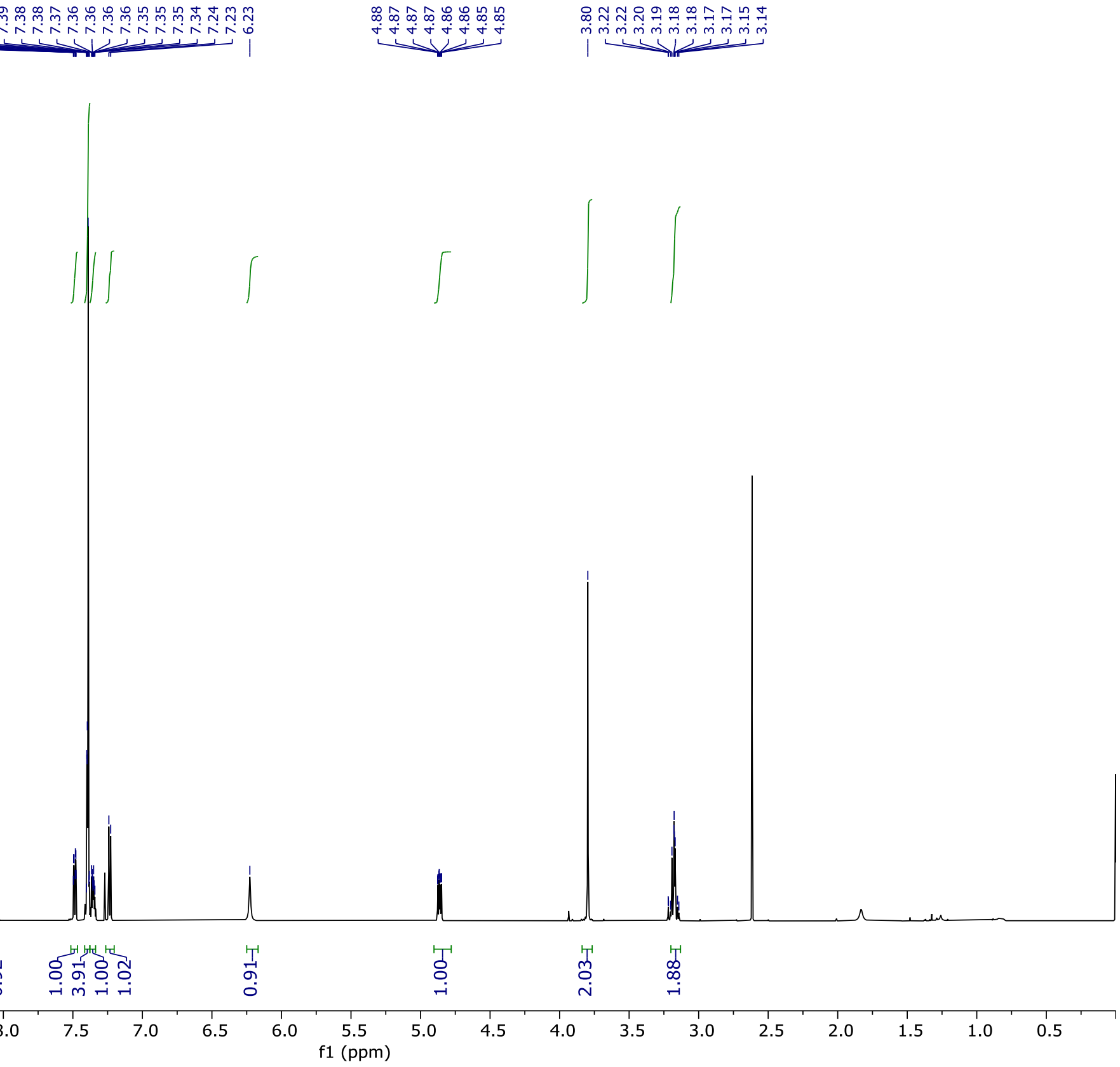

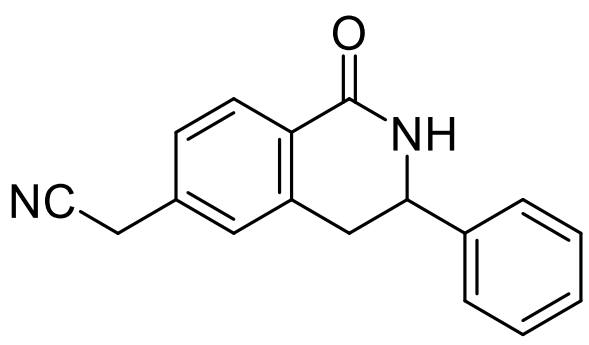

R2-10

^{13}C NMR (151 MHz, CDCl$_3$)

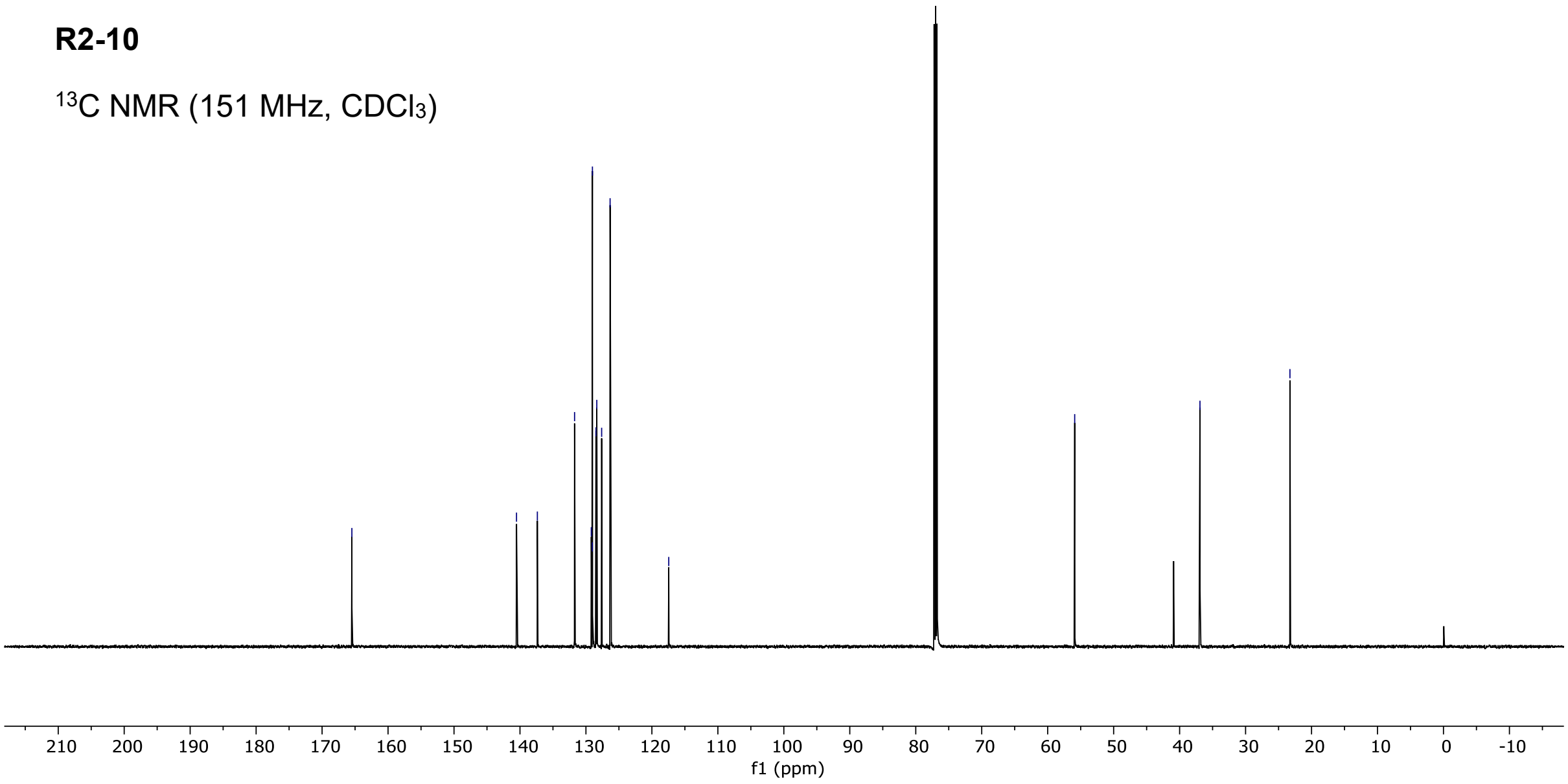

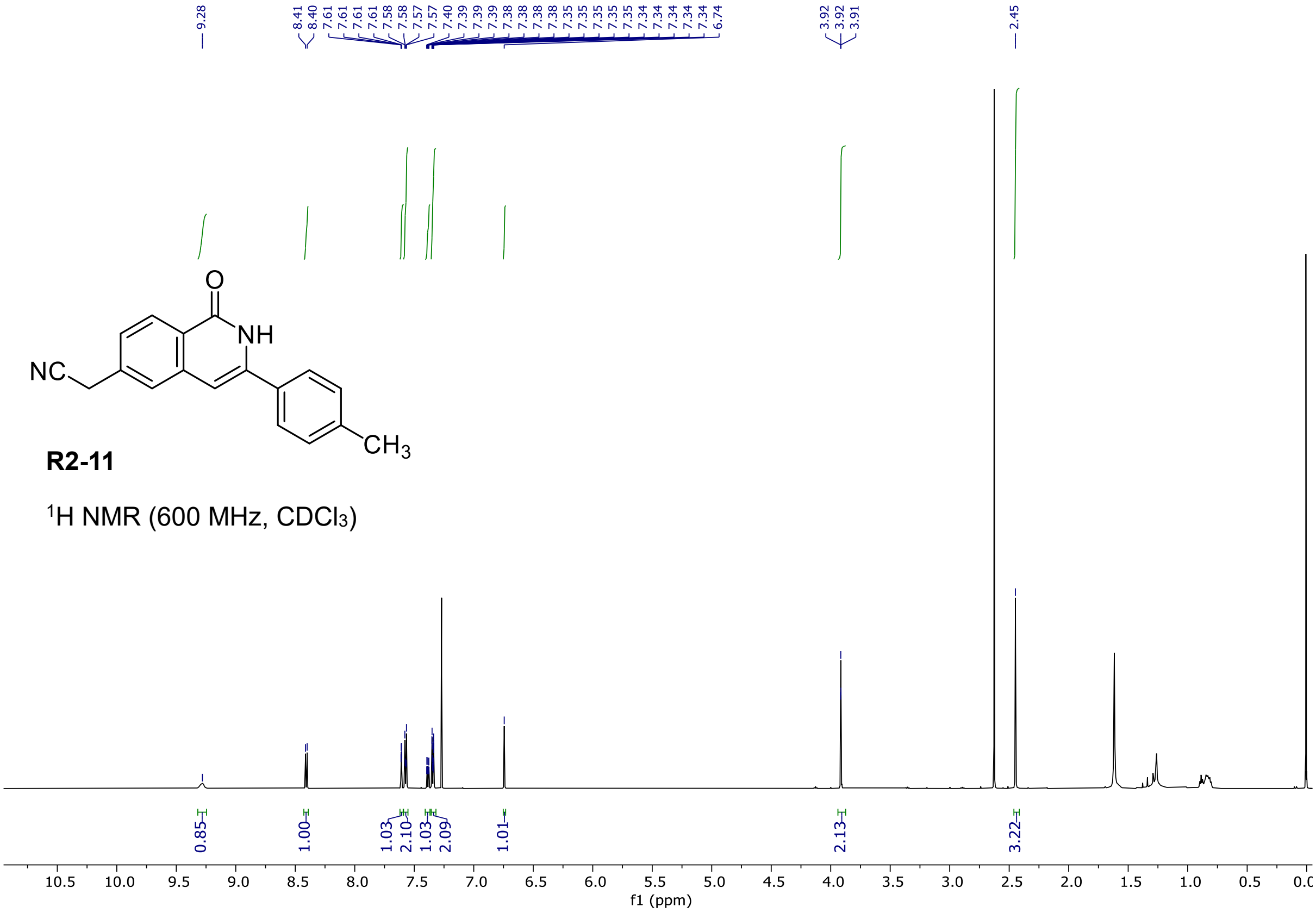
R2-11
¹H NMR (600 MHz, CDCl₃)
9.28
8.41 8.40 7.61 7.61 7.61 7.61 7.58 7.58 7.57 7.57 7.40 7.39 7.39 7.39 7.39 7.38 7.38 7.38 7.38 7.35 7.35 7.35 7.35 7.35 7.34 7.34 7.34 7.34 7.34 6.74
3.92 3.92 3.91
2.45
0.85 1.00 1.03 2.10 1.03 2.09 1.01 2.13 3.22
f1 (ppm)

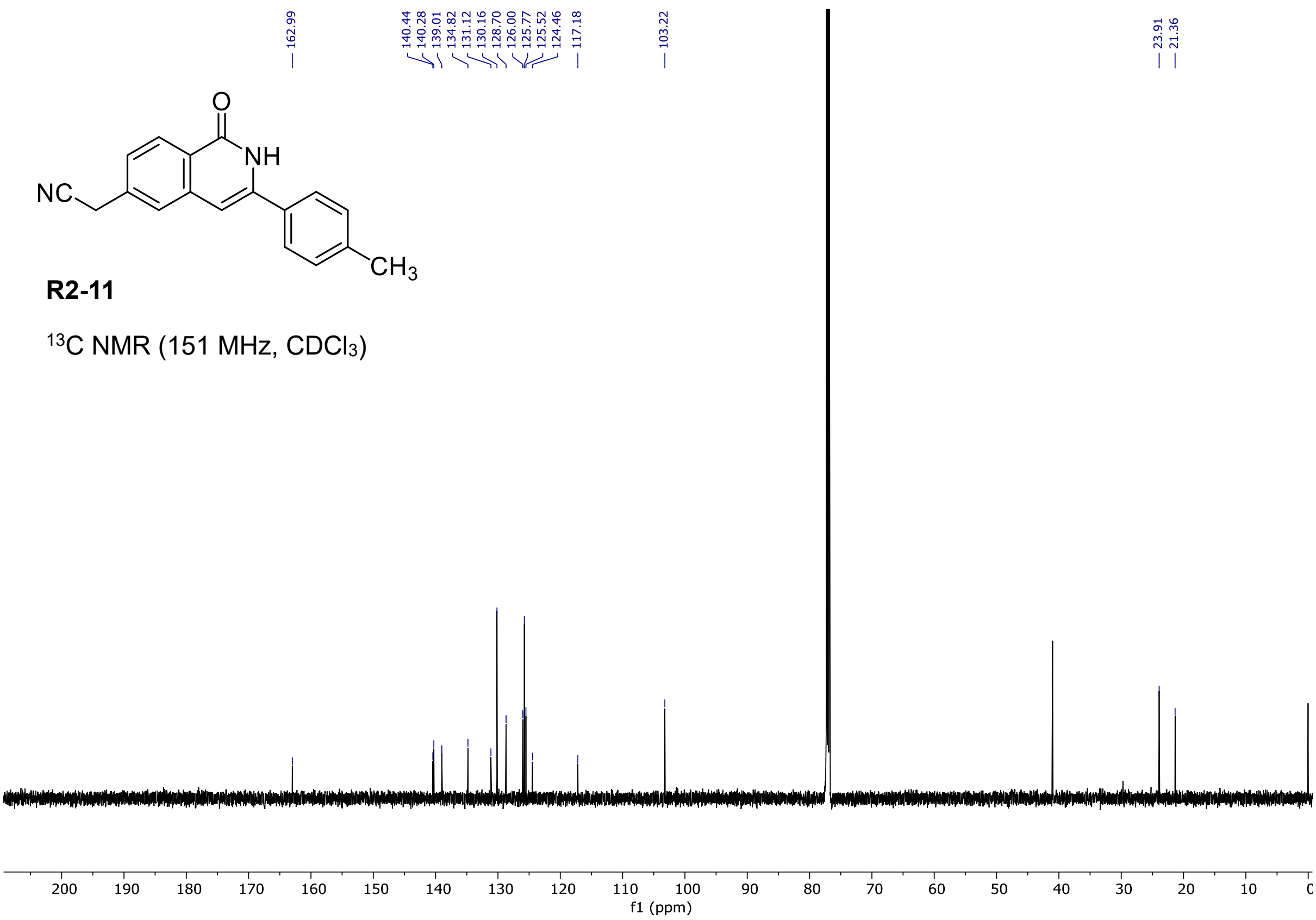
R2-11
¹³C NMR (151 MHz, CDCl₃)
162.99
140.44 140.28 139.01 134.82 131.12 130.16 128.70 126.00 125.77 125.52 124.46 117.18
103.22
23.91 21.36
f1 (ppm)

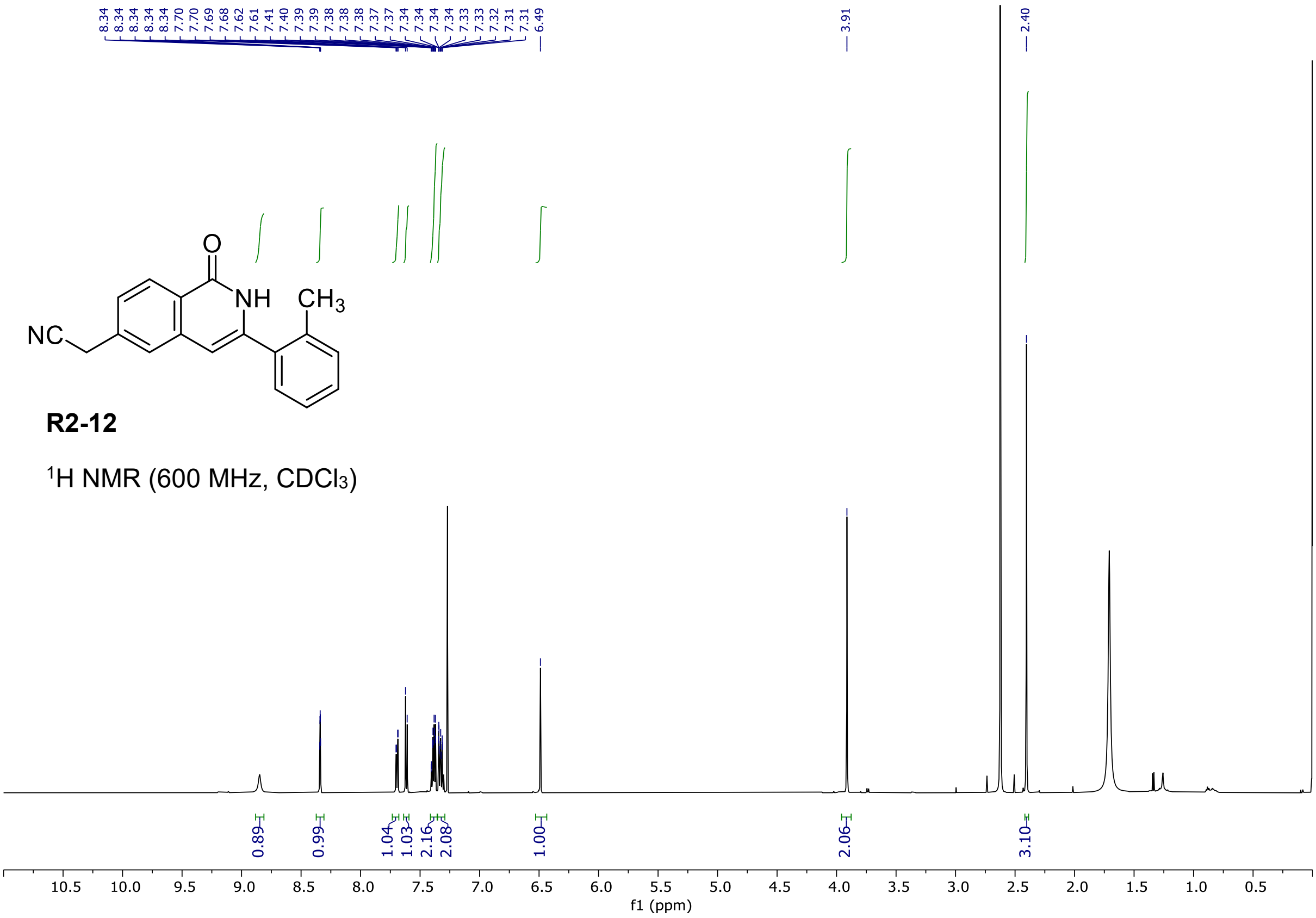
8.34
8.34
8.34
8.34
8.34
7.70
7.70
7.69
7.68
7.62
7.61
7.41
7.40
7.39
7.39
7.38
7.38
7.38
7.37
7.37
7.34
7.34
7.34
7.34
7.33
7.32
7.31
7.31
6.49
3.91
2.40
NC
O
NH
CH3
R2-12
1H NMR (600 MHz, CDCl3)
0.89
0.99
1.04
1.03
2.16
2.08
1.00
2.06
3.10
10.5
10.0
9.5
9.0
8.5
8.0
7.5
7.0
6.5
6.0
5.5
5.0
4.5
4.0
3.5
3.0
2.5
2.0
1.5
1.0
0.5
f1 (ppm)

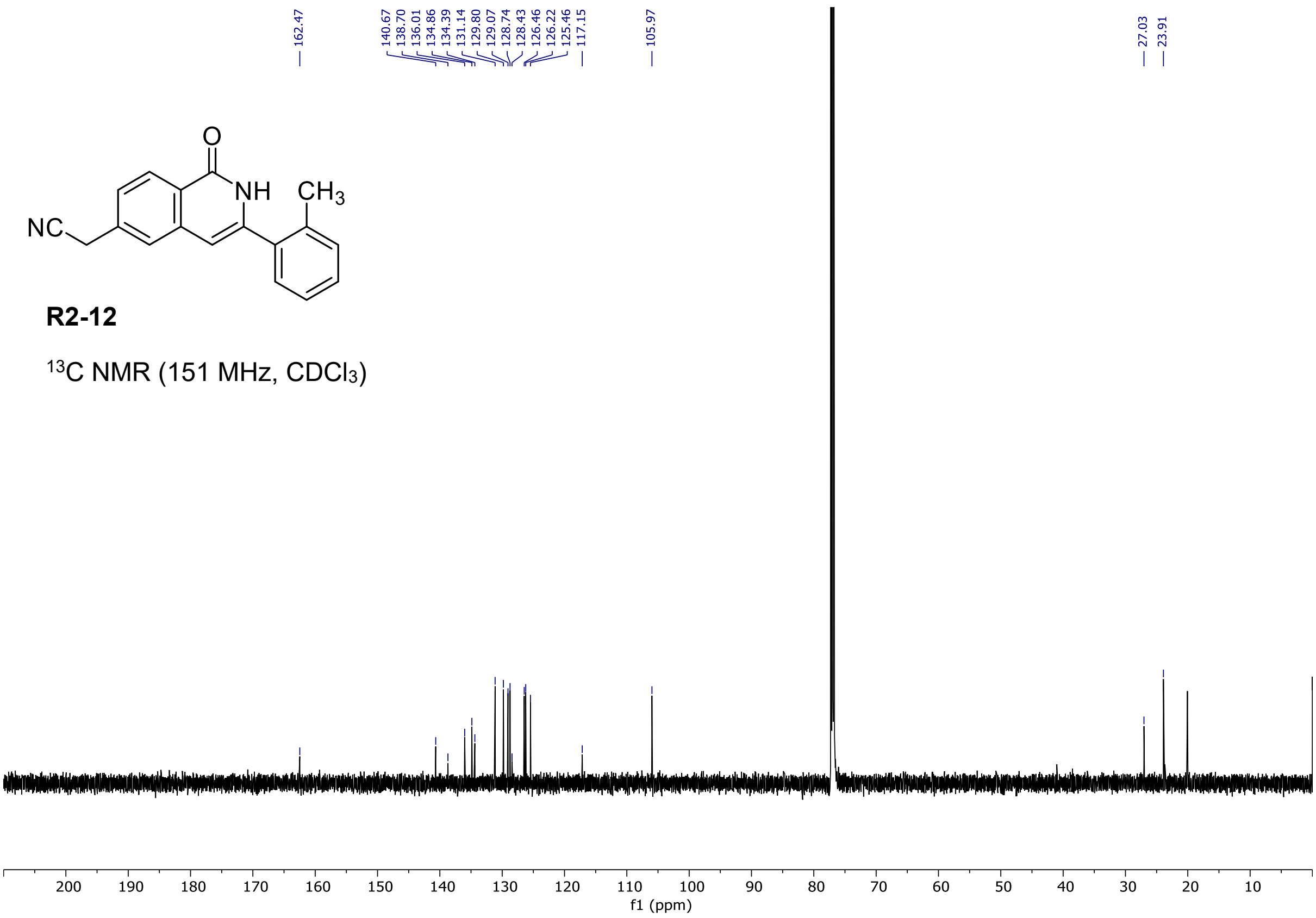
162.47
140.67
138.70
136.01
134.86
134.39
131.14
129.80
129.07
128.74
128.43
126.46
126.22
125.46
117.15
105.97
27.03
23.91
NC
O
NH
CH3
R2-12
13C NMR (151 MHz, CDCl3)
200
190
180
170
160
150
140
130
120
110
100
90
80
70
60
50
40
30
20
10
f1 (ppm)

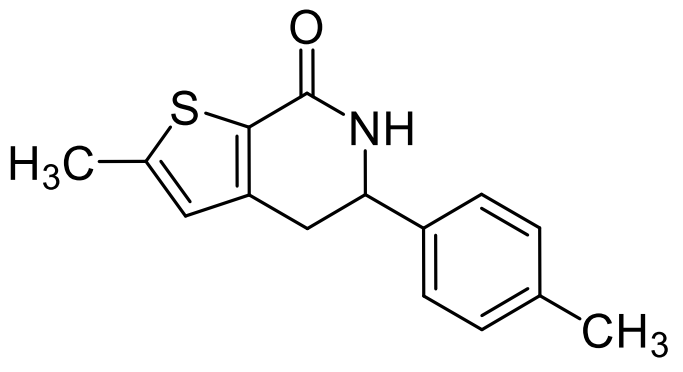

R2-14

^{1}H NMR (600 MHz, $CDCl_3$)

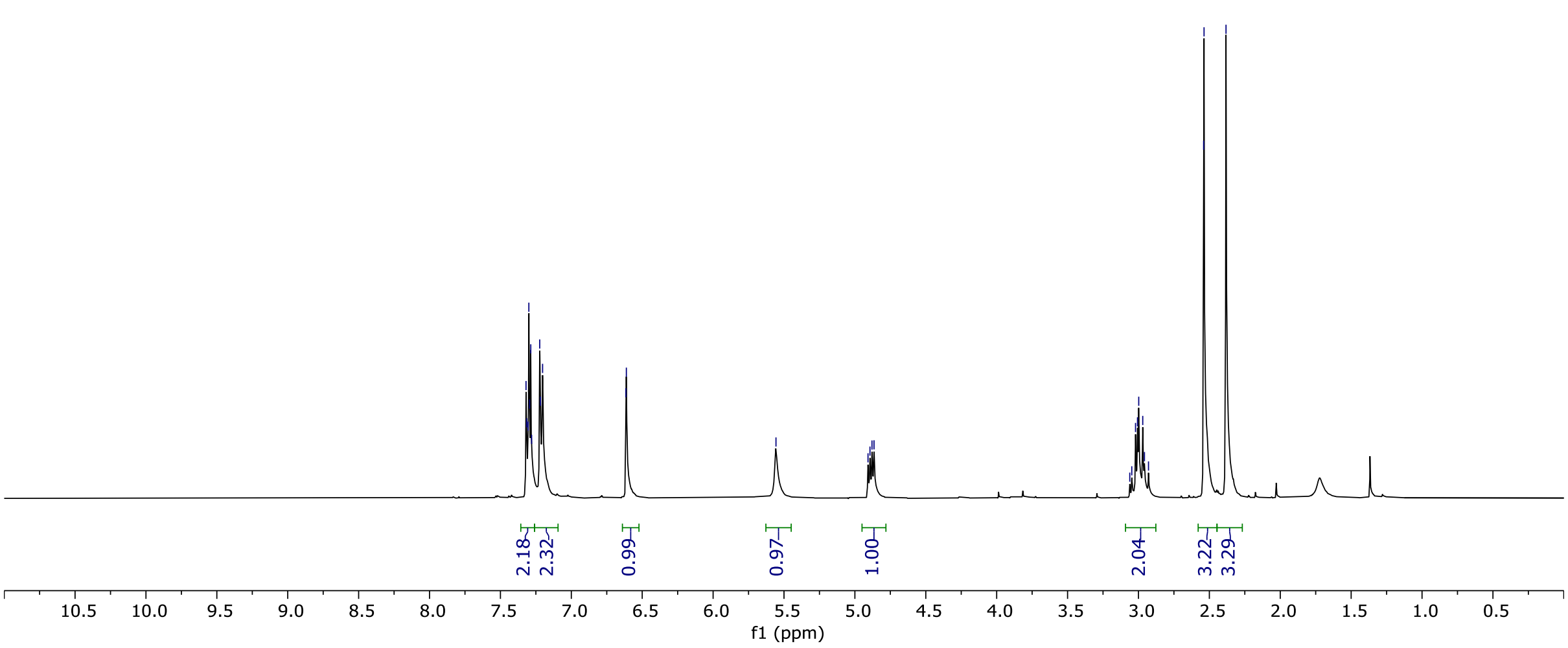

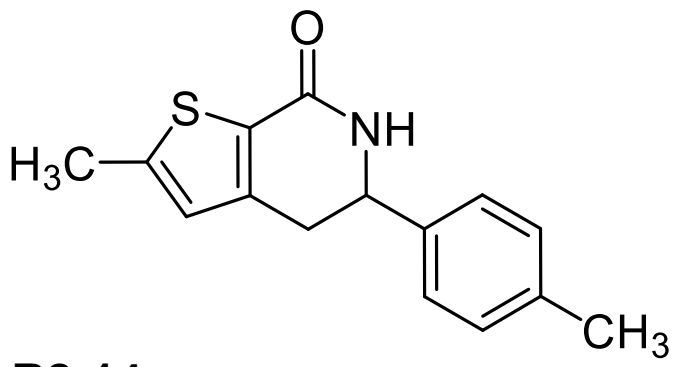

R2-14

^{13}C NMR (600 MHz, $CDCl_3$)

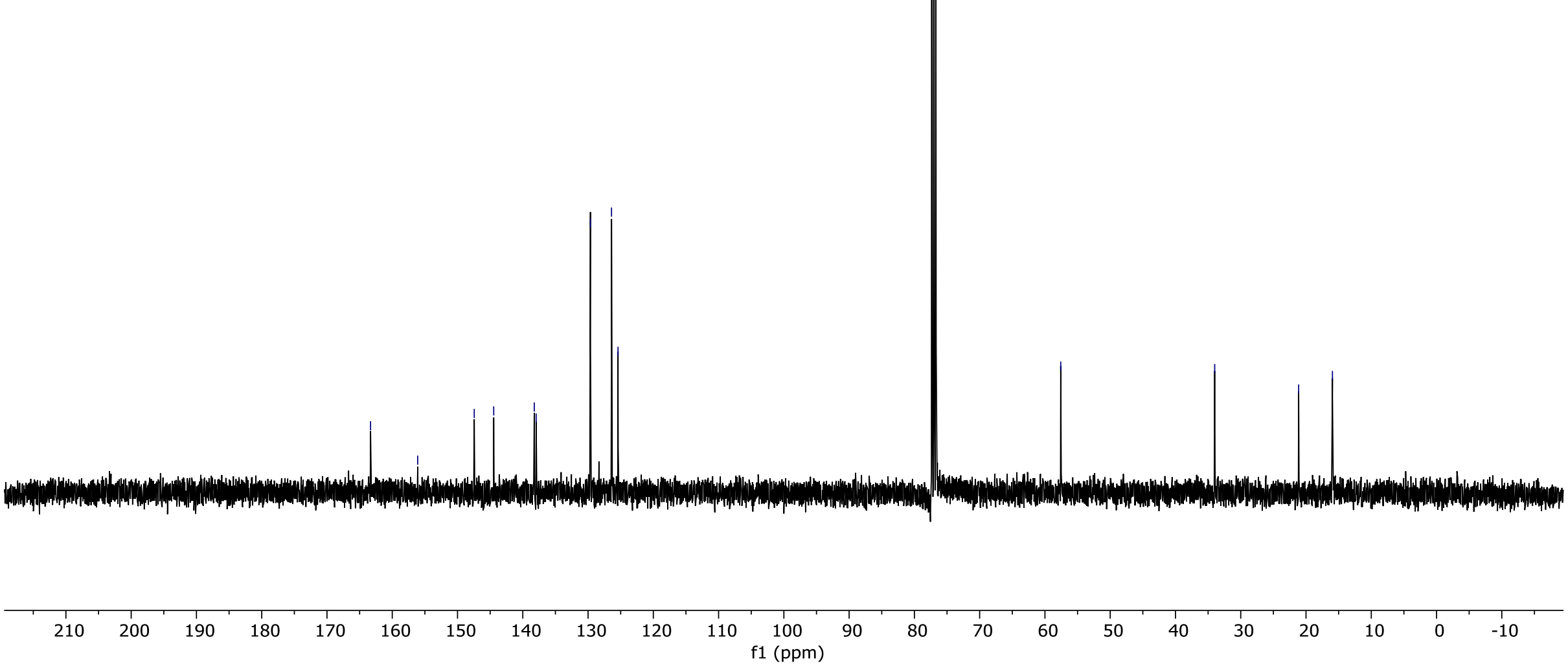

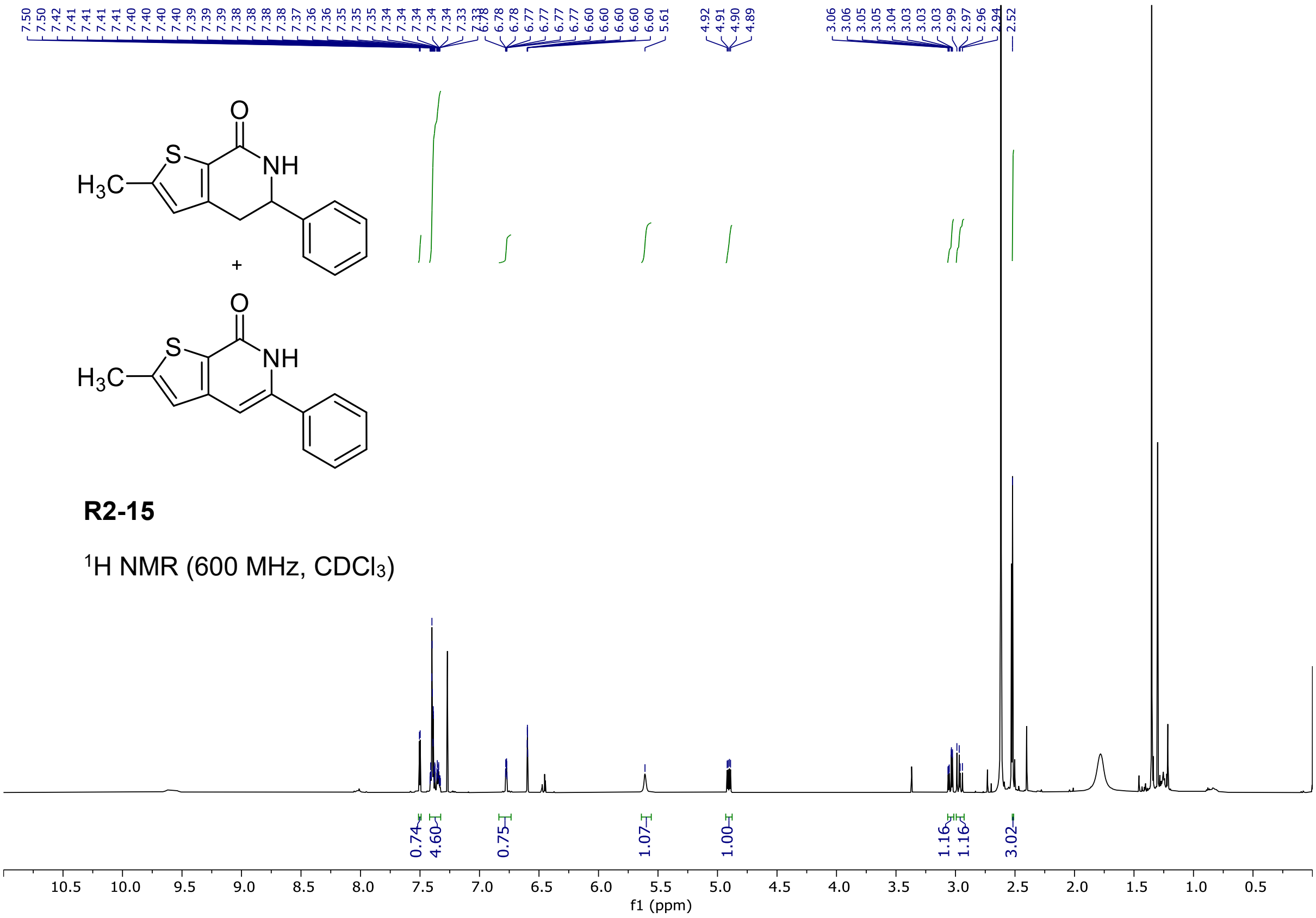

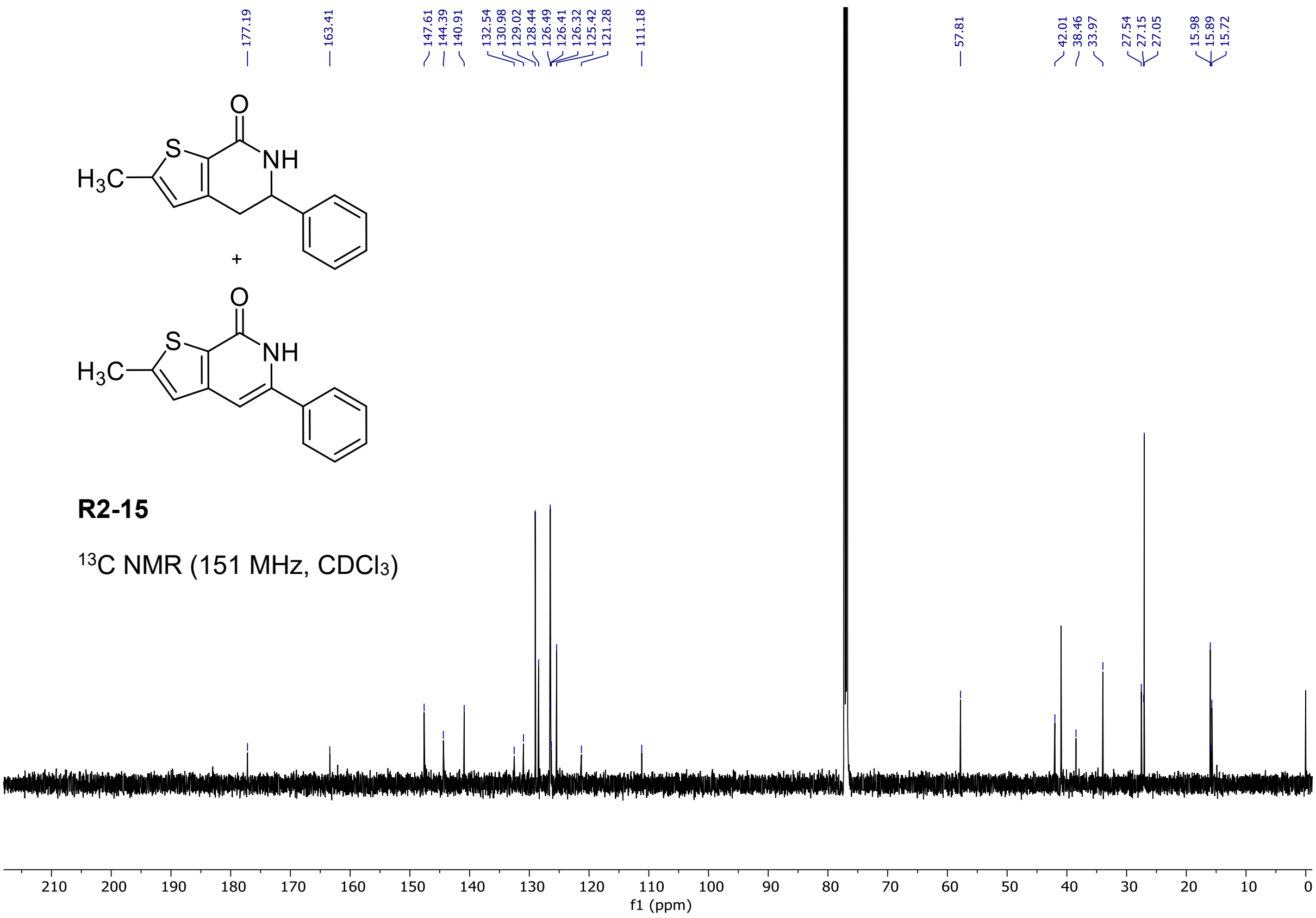

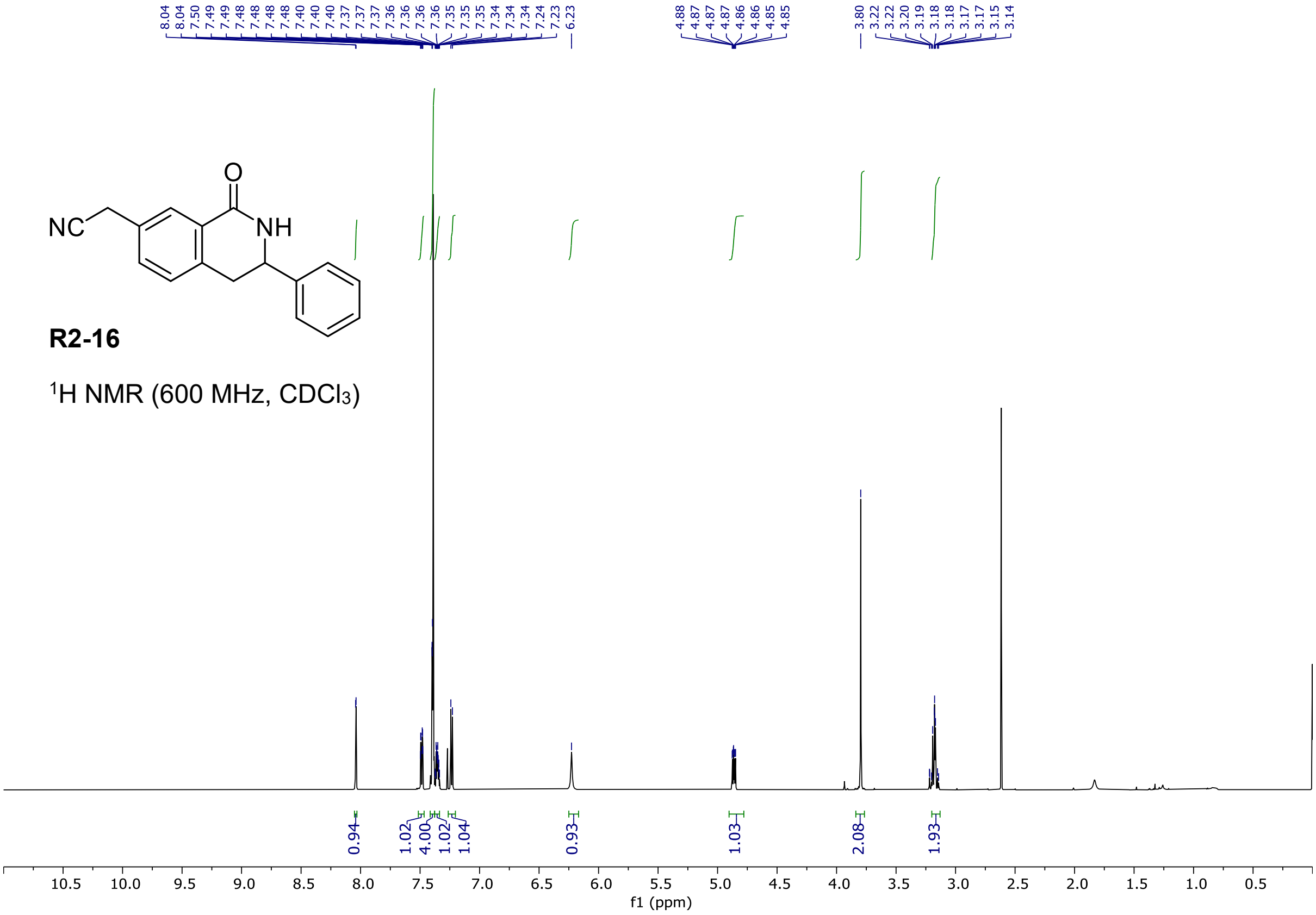
8.04 8.04 7.50 7.49 7.49 7.48 7.48 7.48 7.48 7.40 7.40 7.40 7.37 7.37 7.37 7.36 7.36 7.36 7.36 7.35 7.35 7.35 7.34 7.34 7.34 7.24 7.23 6.23
4.88 4.87 4.87 4.87 4.86 4.86 4.85 4.85
3.80 3.22 3.22 3.20 3.19 3.18 3.18 3.17 3.17 3.15 3.14
O
NC
NH
R2-16
1H NMR (600 MHz, CDCl3)
0.94
1.02
4.00
1.02
1.04
0.93
1.03
2.08
1.93
10.5 10.0 9.5 9.0 8.5 8.0 7.5 7.0 6.5 6.0 5.5 5.0 4.5 4.0 3.5 3.0 2.5 2.0 1.5 1.0 0.5
f1 (ppm)

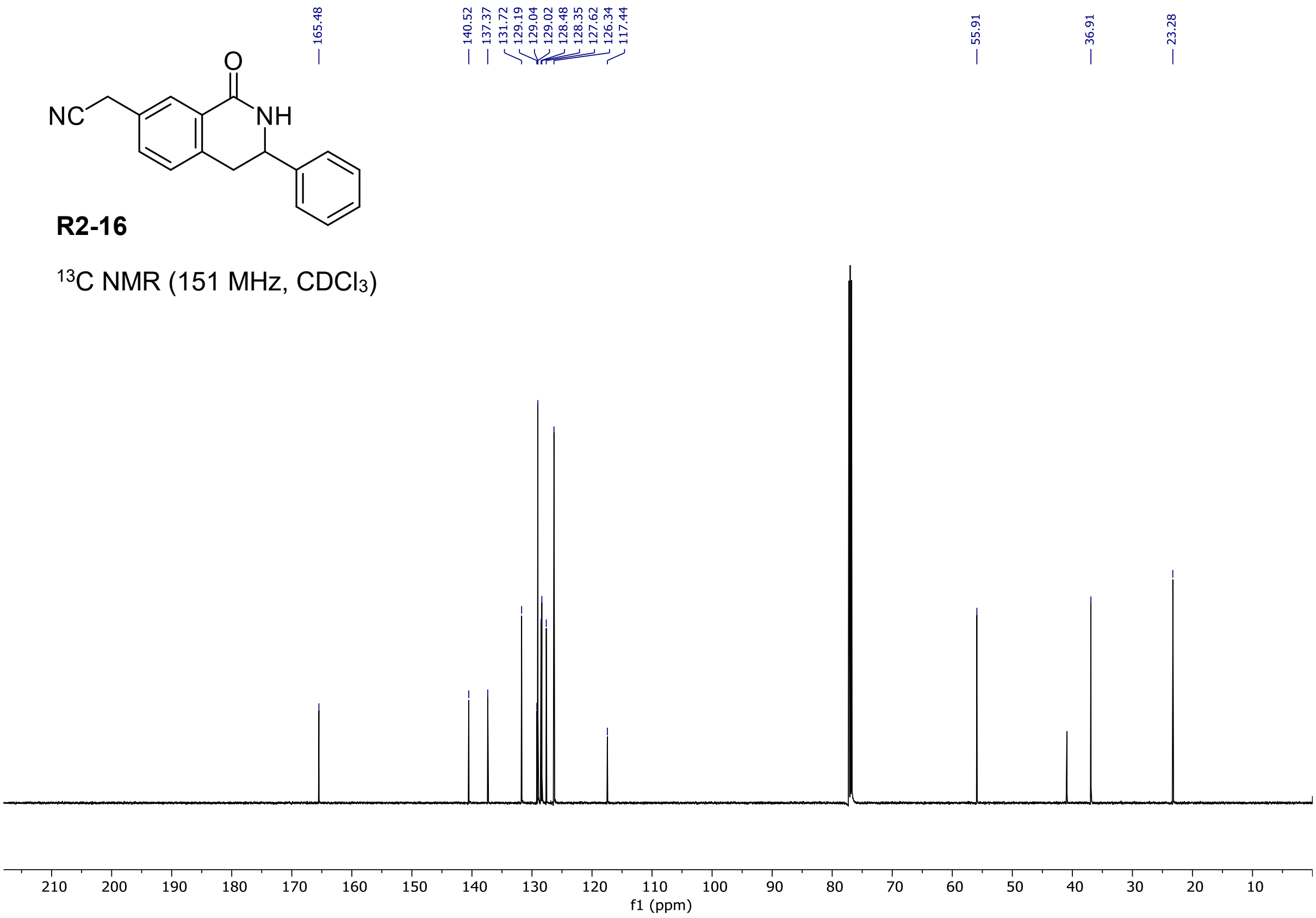
165.48
140.52 137.37 131.72 129.19 129.04 129.02 128.48 128.35 127.62 126.34 117.44
55.91
36.91
23.28
O
NC
NH
R2-16
13C NMR (151 MHz, CDCl3)
210 200 190 180 170 160 150 140 130 120 110 100 90 80 70 60 50 40 30 20 10
f1 (ppm)

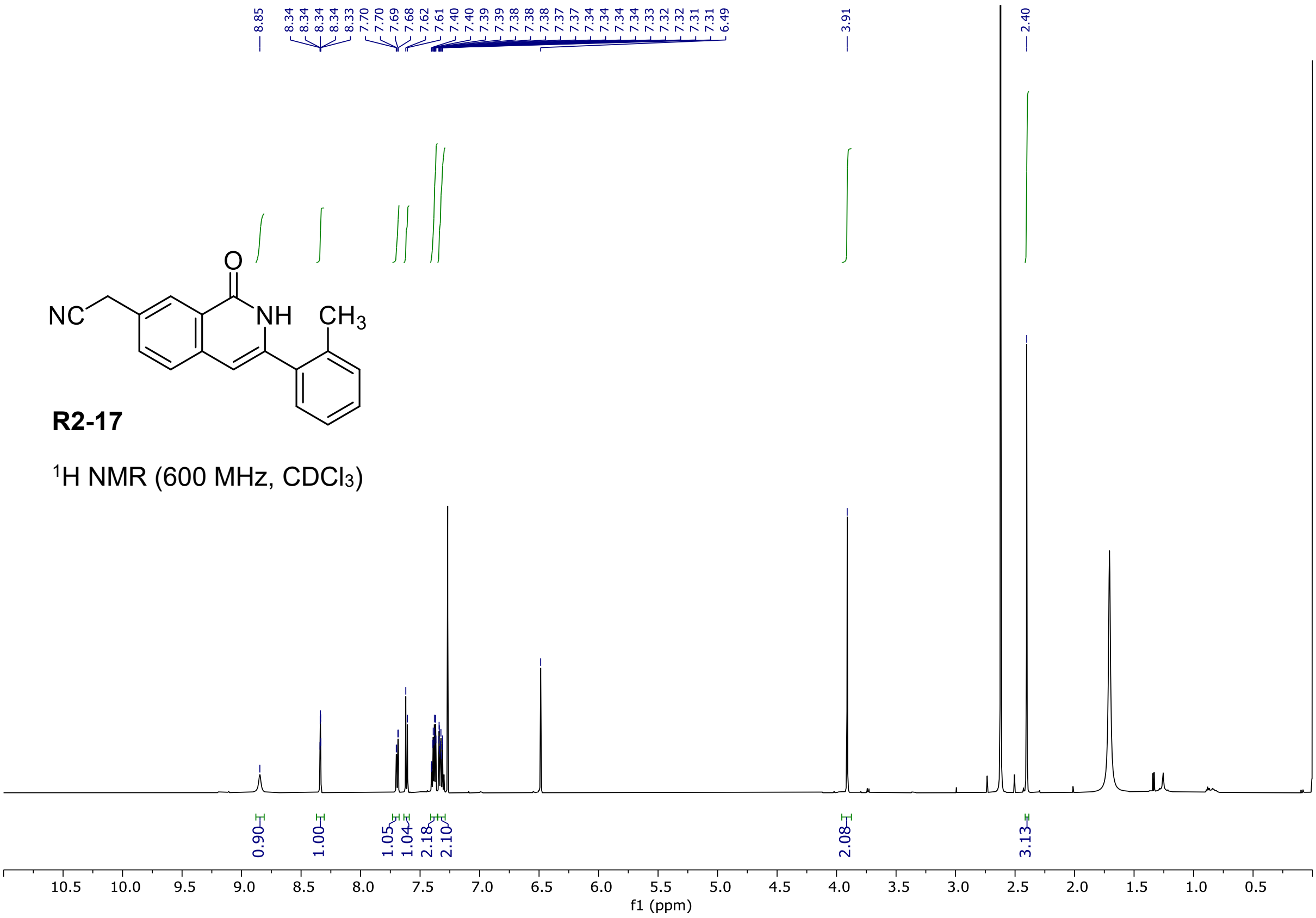

R2-17

^{1}H NMR (600 MHz, $CDCl_3$)

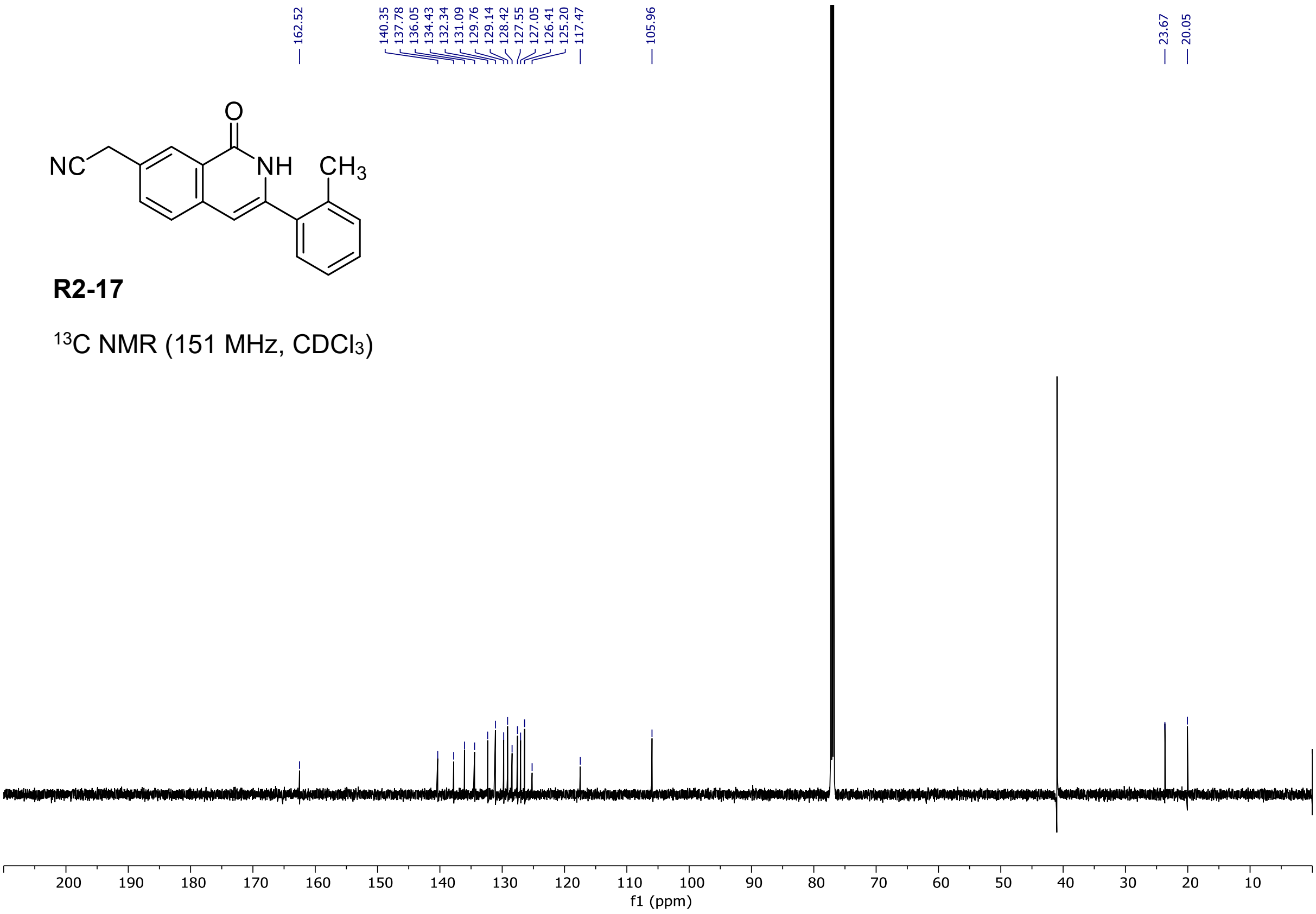

R2-17

^{13}C NMR (151 MHz, $CDCl_3$)

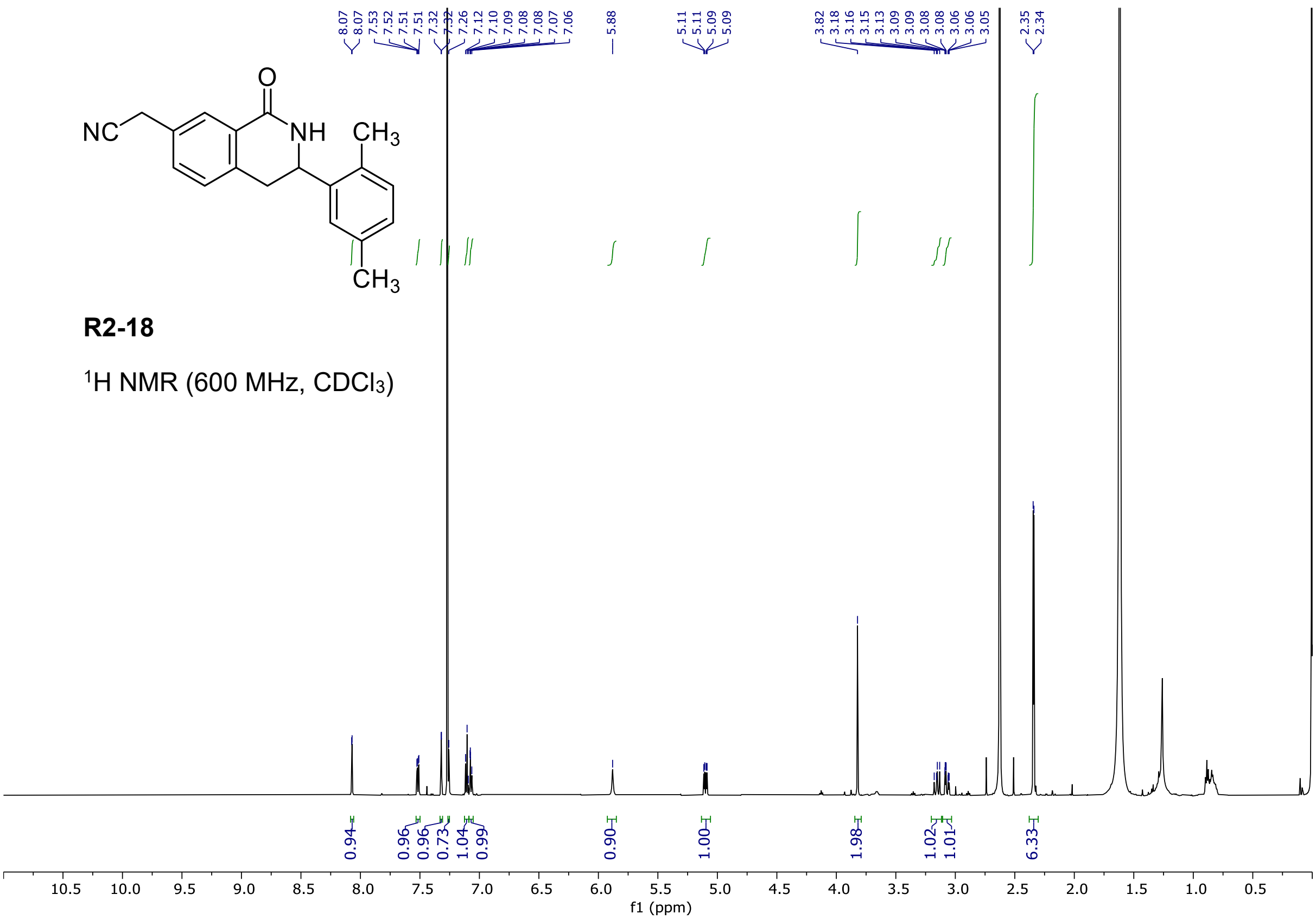
NC
O
NH
CH3
CH3
R2-18
1H NMR (600 MHz, CDCl3)
f1 (ppm)

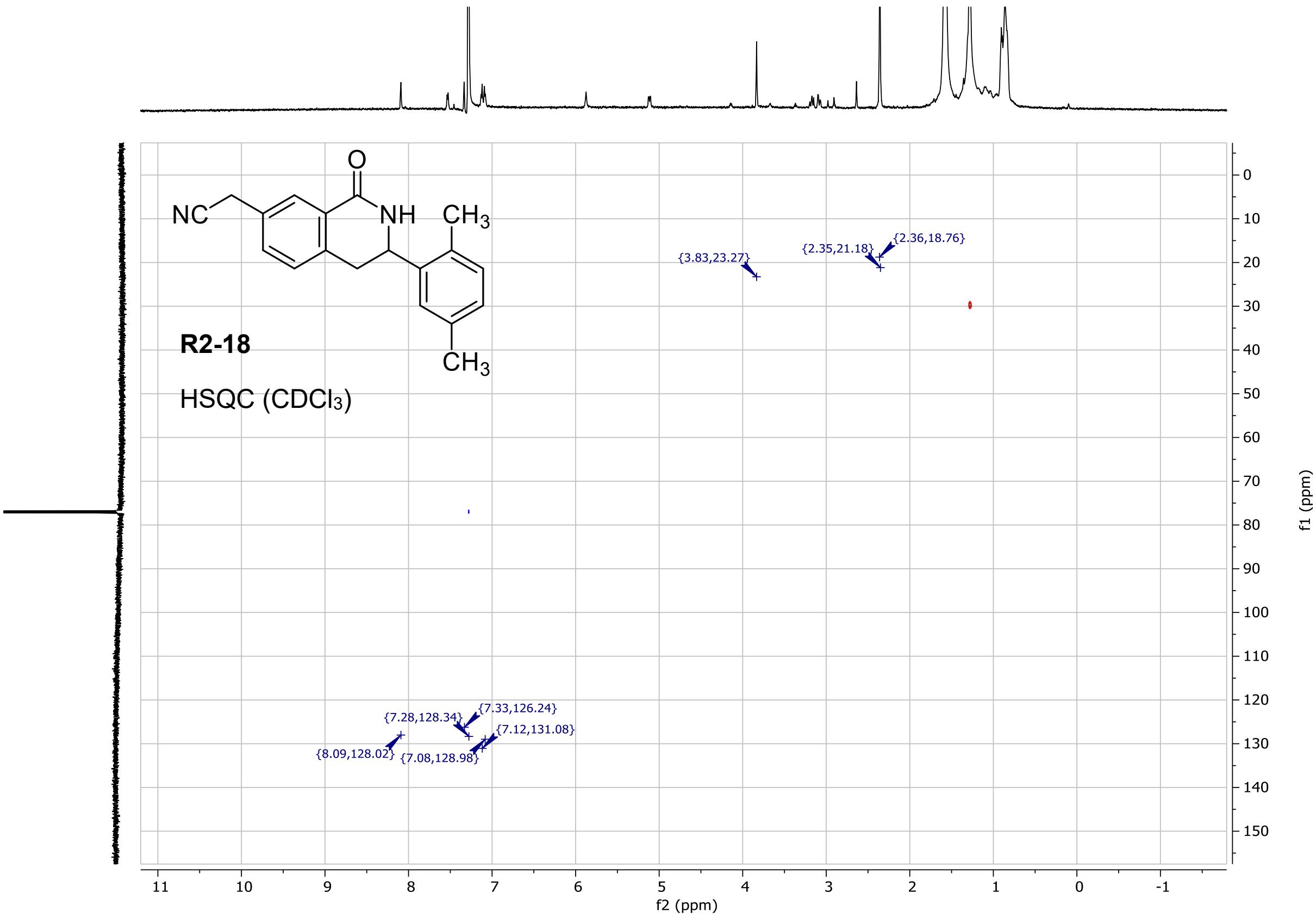
NC
O
NH
CH3
CH3
R2-18
HSQC (CDCl3)
{3.83,23.27}
{2.35,21.18}
{2.36,18.76}
{7.28,128.34}
{7.33,126.24}
{7.12,131.08}
{8.09,128.02}
{7.08,128.98}
f2 (ppm)
f1 (ppm)

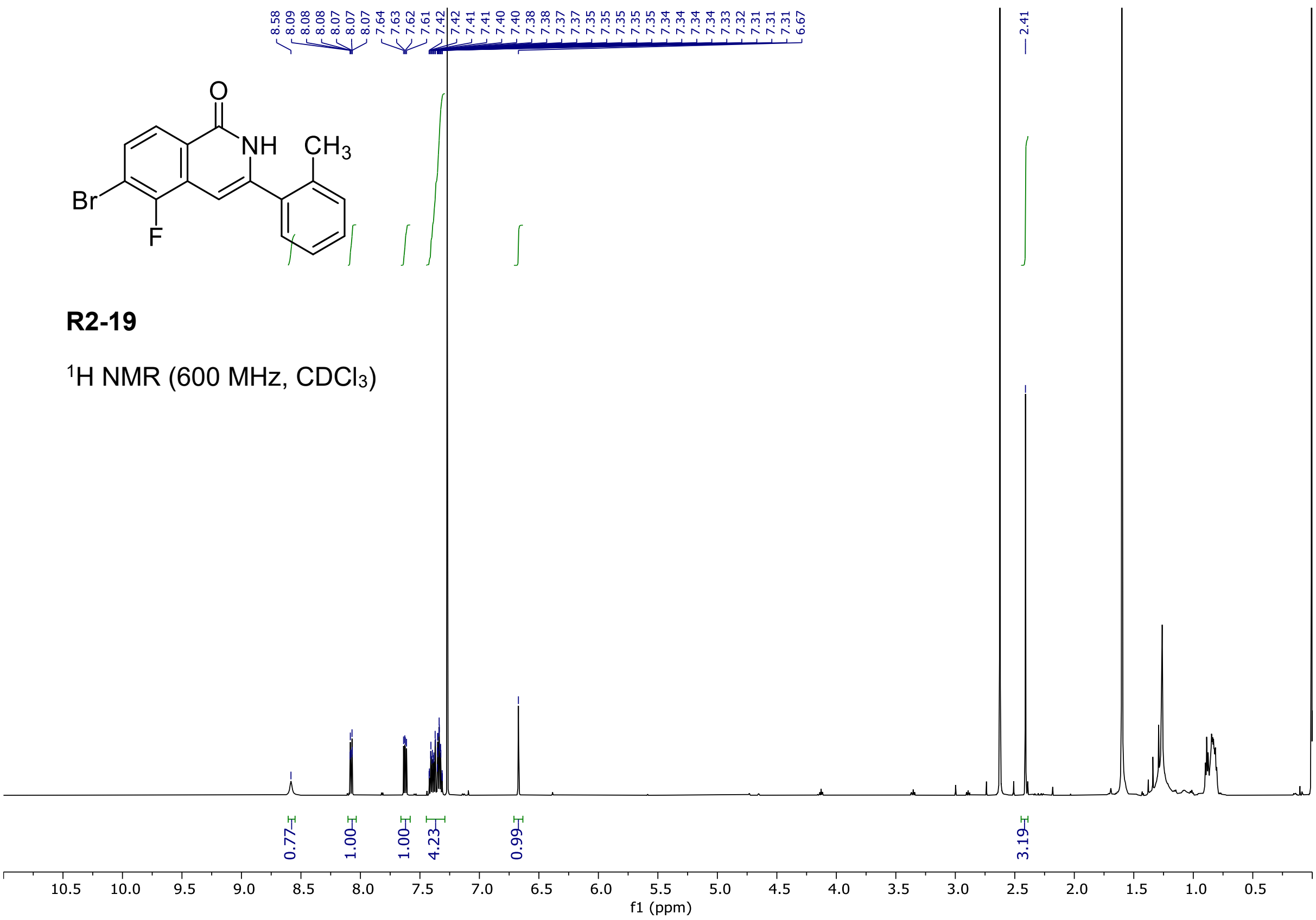

R2-19

^{1}H NMR (600 MHz, $CDCl_3$)

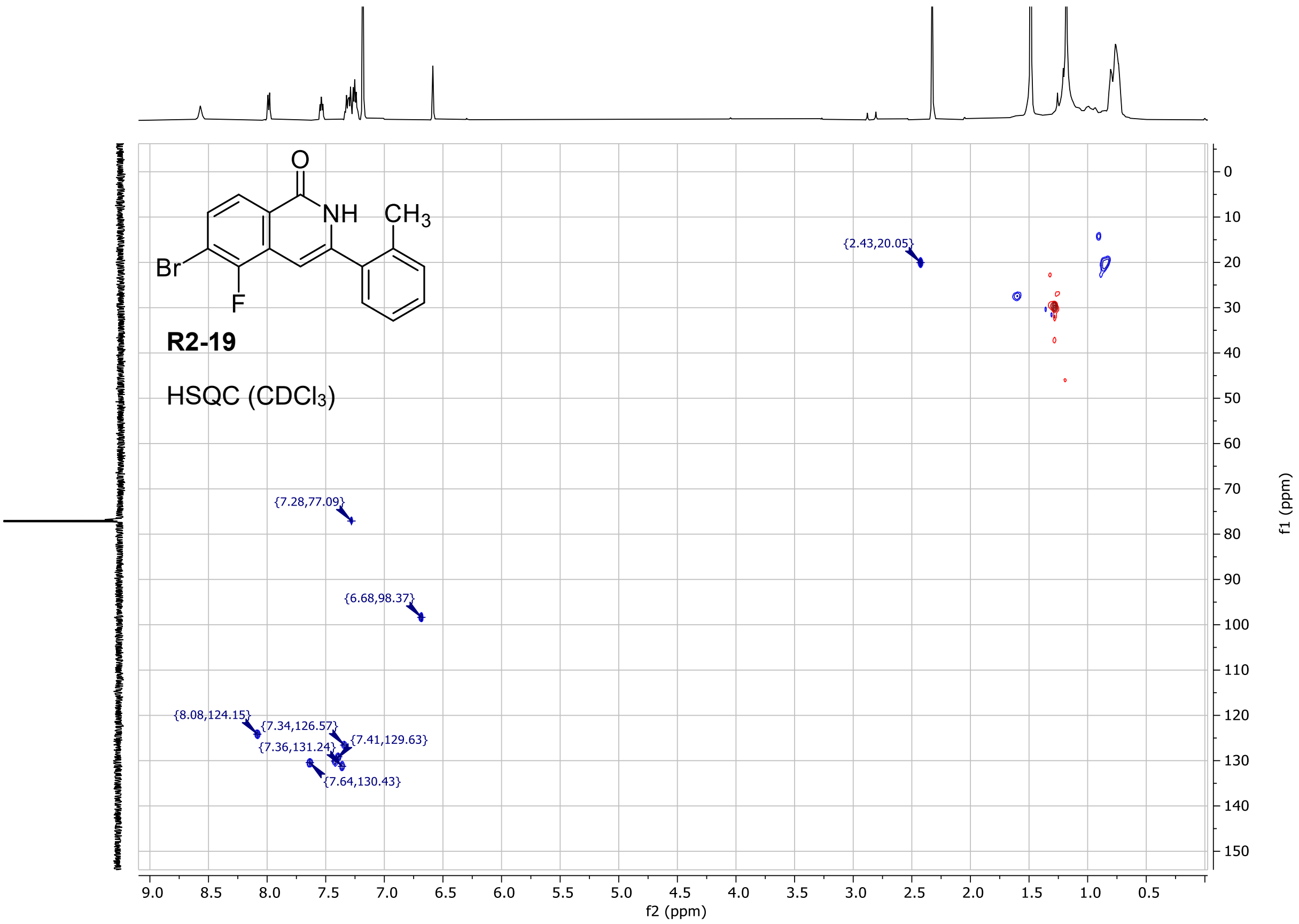

R2-19

HSQC ($CDCl_3$)

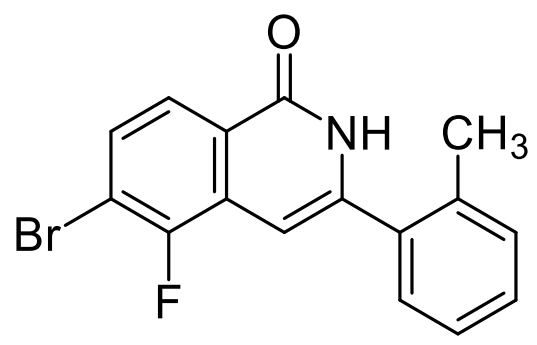

R2-19

^{19}F NMR (376 MHz, $CDCl_3$)

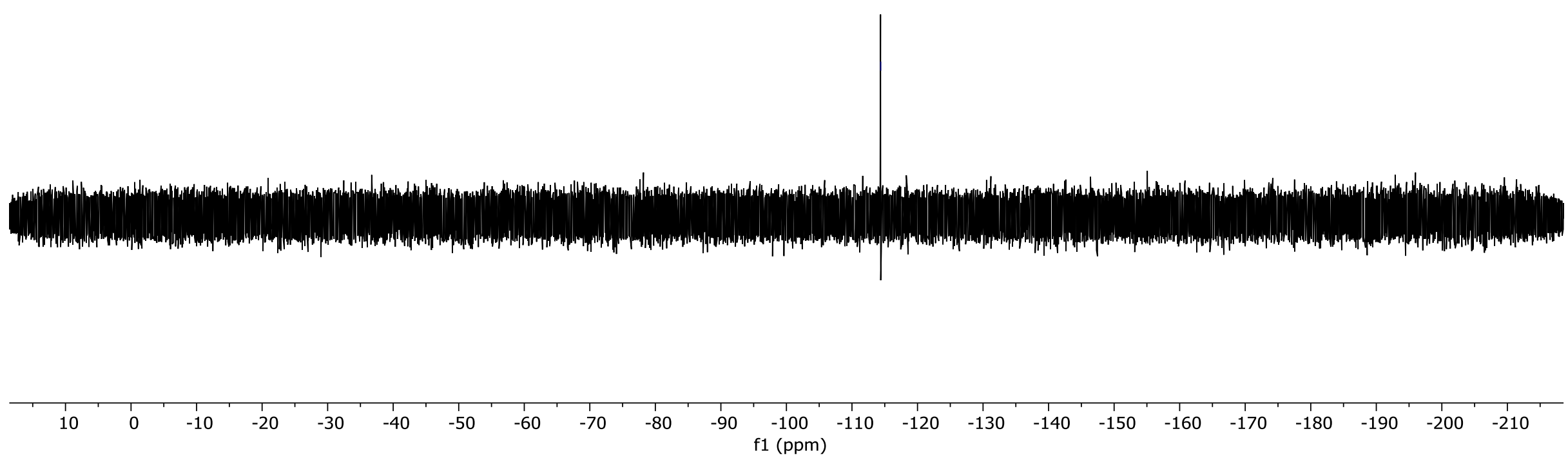

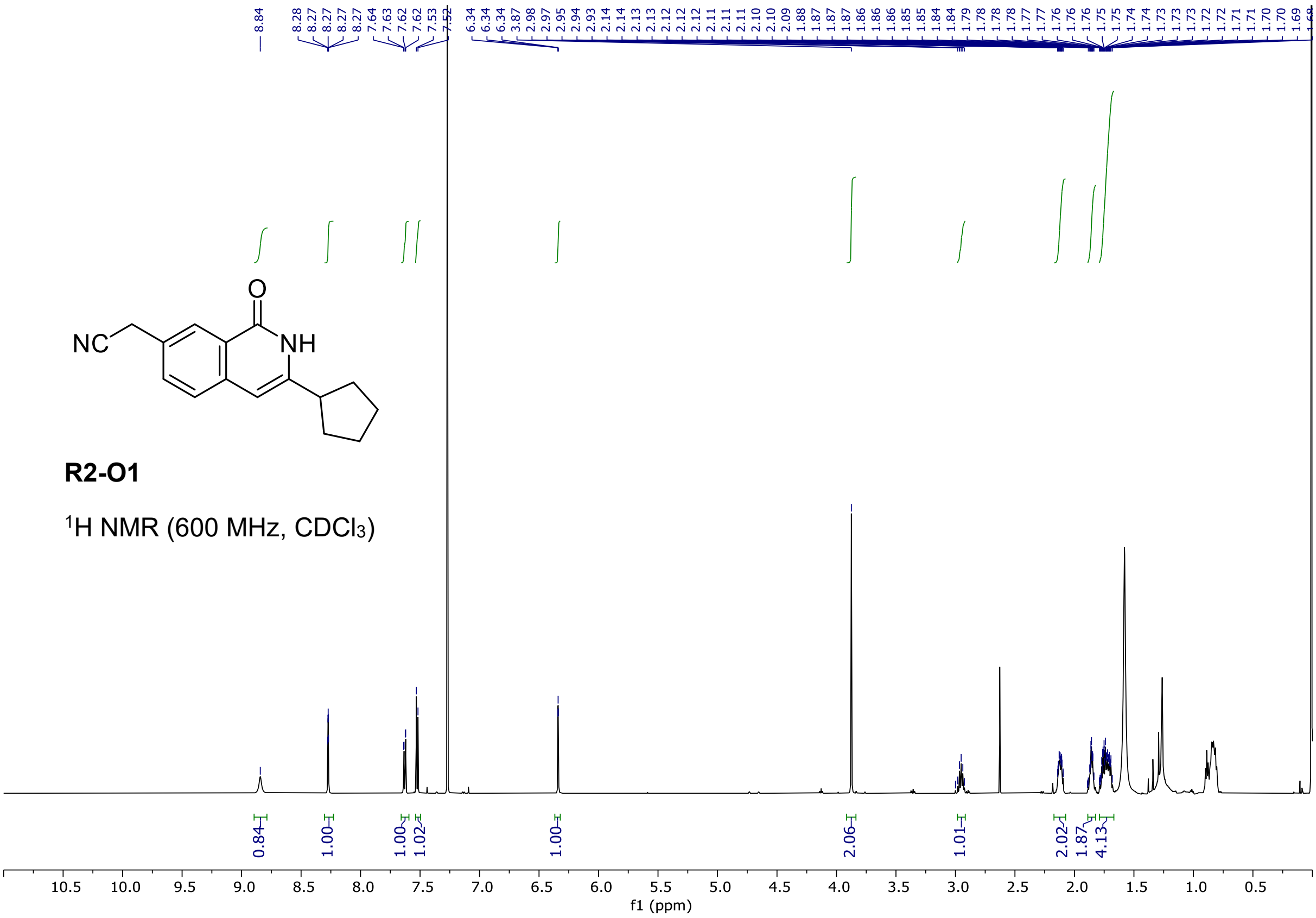
R2-O1
^{1}H NMR (600 MHz, CDCl$_3$)
f1 (ppm)

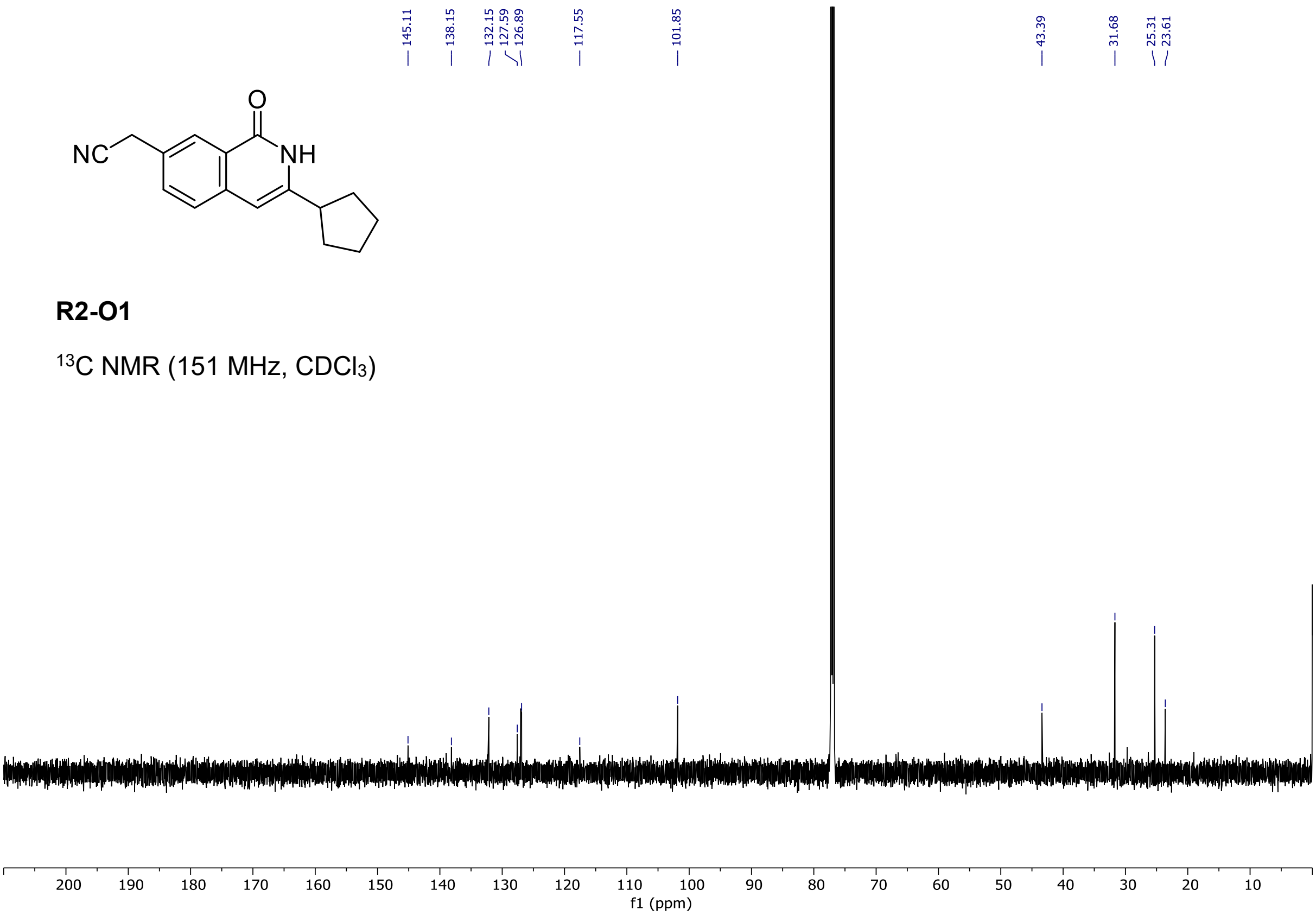
145.11
138.15
132.15
127.59
126.89
117.55
101.85
43.39
31.68
25.31
23.61
R2-O1
^{13}C NMR (151 MHz, CDCl$_3$)
f1 (ppm)

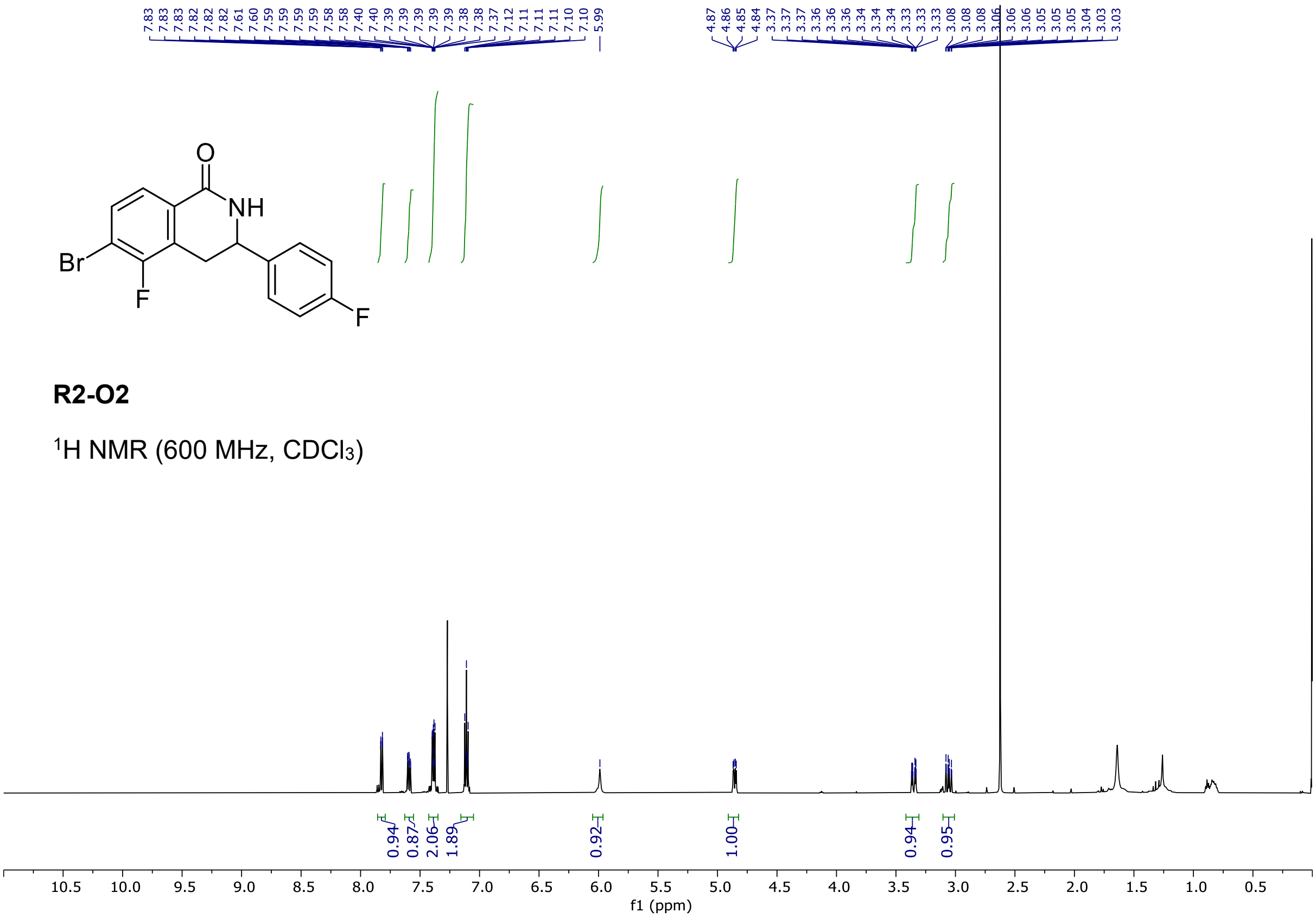

R2-O2

^{1}H NMR (600 MHz, $CDCl_3$)

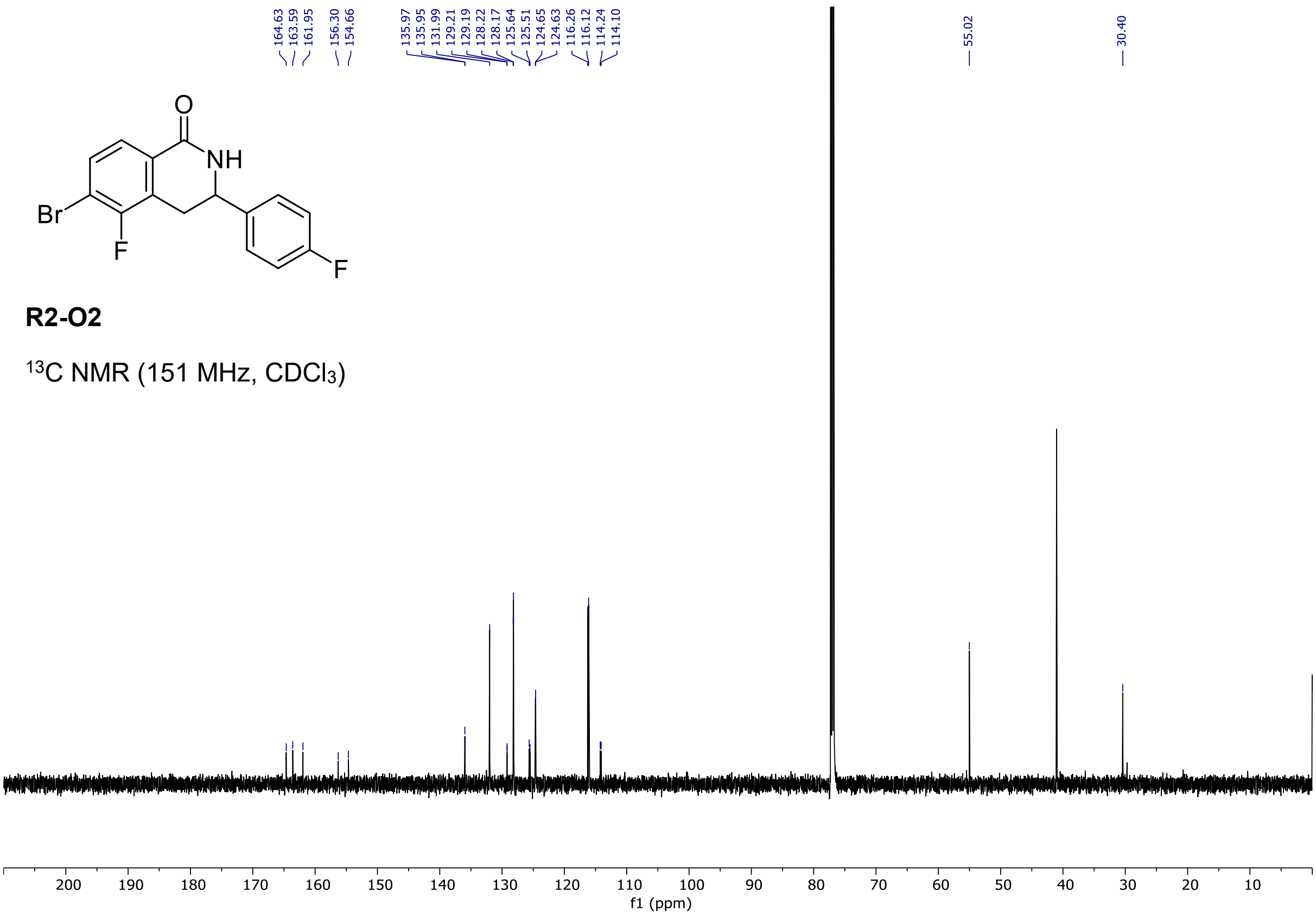

R2-O2

^{13}C NMR (151 MHz, $CDCl_3$)

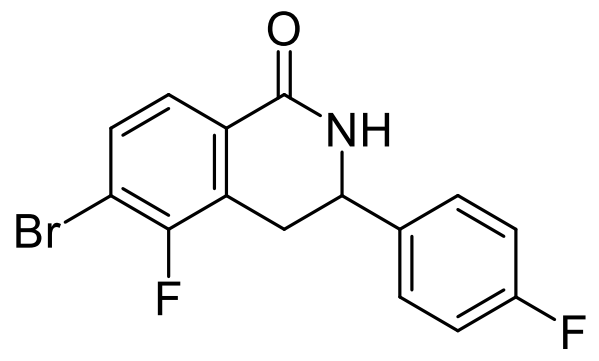

R2-O2

^{19}F NMR (376 MHz, $CDCl_3$)

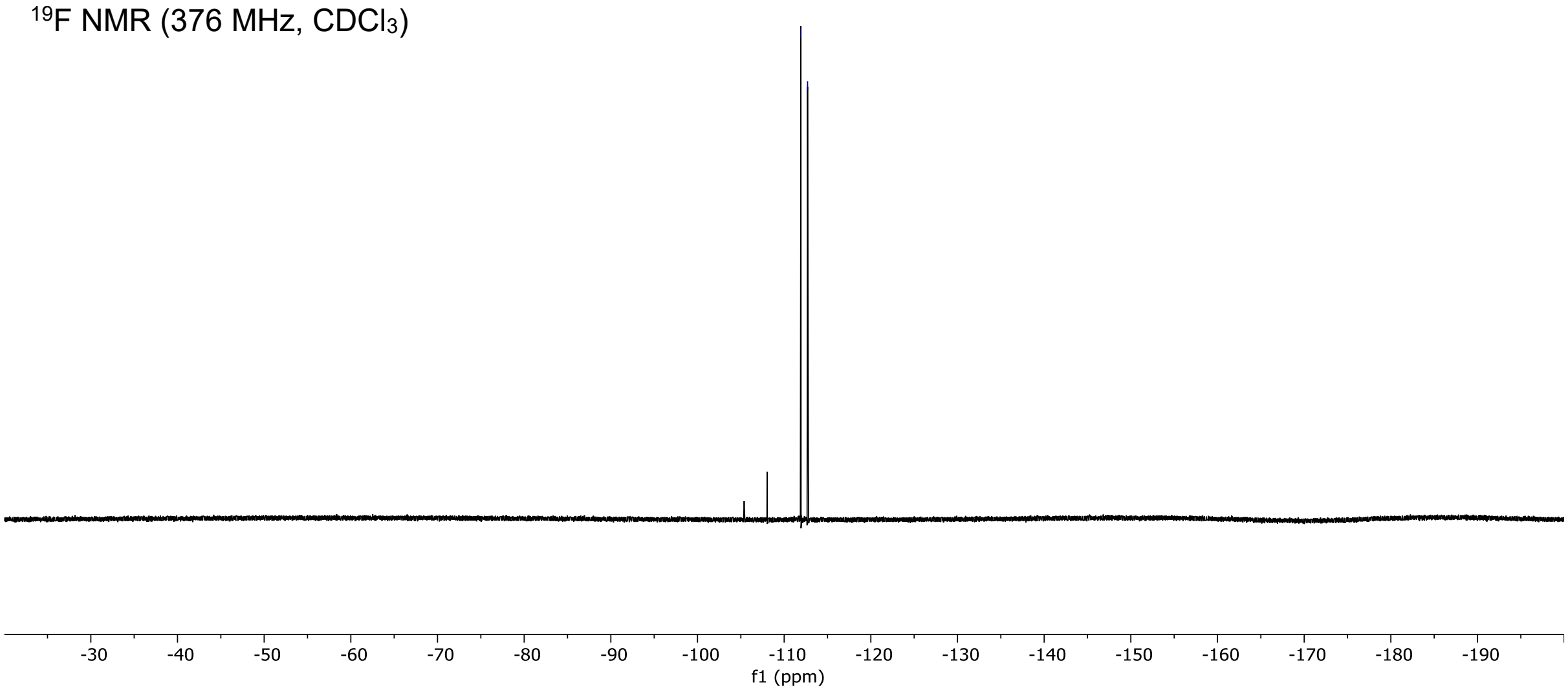

Supplement: Supplementary file 2 [file 42004_2026_2066_MOESM2_ESM.pdf]
